# An oncogenic role of Agrin in regulating focal adhesion integrity in hepatocellular carcinoma

**DOI:** 10.1038/ncomms7184

**Published:** 2015-01-29

**Authors:** Sayan Chakraborty, Manikandan Lakshmanan, Hannah L.F. Swa, Jianxiang Chen, Xiaoqian Zhang, Yan Shan Ong, Li Shen Loo, Semih Can Akıncılar, Jayantha Gunaratne, Vinay Tergaonkar, Kam M. Hui, Wanjin Hong

**Affiliations:** 1 Institute of Molecular and Cell Biology, Agency for Science, Technology and Research (A*STAR), 61, Biopolis Drive, Proteos, Singapore 138673, Singapore; 2 Laboratory of Cancer Genomics, Cellular and Molecular Research Division, National Cancer Center Singapore, 11, Hospital drive, Singapore 169610, Singapore

## Abstract

Hepatocellular carcinoma (HCC) is one of the leading causes of cancer-related deaths globally. The identity and role of cell surface molecules driving complex biological events leading to HCC progression are poorly understood, hence representing major lacunae in HCC therapies. Here, combining SILAC quantitative proteomics and biochemical approaches, we uncover a critical oncogenic role of Agrin, which is overexpressed and secreted in HCC. Agrin enhances cellular proliferation, migration and oncogenic signalling. Mechanistically, Agrin’s extracellular matrix sensor activity provides oncogenic cues to regulate Arp2/3-dependent ruffling, invadopodia formation and epithelial–mesenchymal transition through sustained focal adhesion integrity that drives liver tumorigenesis. Furthermore, Agrin signalling through Lrp4-muscle-specific tyrosine kinase (MuSK) forms a critical oncogenic axis. Importantly, antibodies targeting Agrin reduced oncogenic signalling and tumour growth *in vivo*. Together, we demonstrate that Agrin is frequently upregulated and important for oncogenic property of HCC, and is an attractive target for antibody therapy.

## Abstract (web summary)

The proteoglycan Agrin is known to be expressed in neurons and muscle and to bind ECM protein laminin. Here the authors report that Agrin promotes hepatocellular carcinoma by stimulating proliferation, decreasing focal adhesion, increasing invasiveness and promoting an epithelial-to-mesenchymal transition.

## Graphical abstract

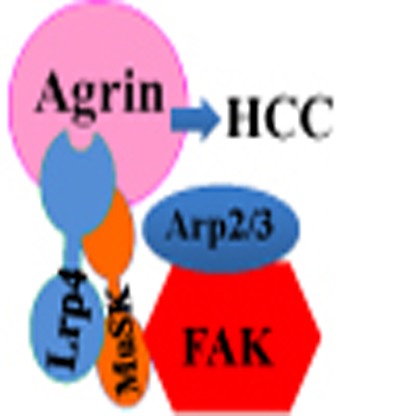

Hepatocellular carcinoma (HCC) is one of the most common malignancies having a high mortality rate worldwide^1^. The cell surface proteins defining various signalling pathways in HCC are not very well characterized^2^, and current therapies are largely restricted to targeting receptor tyrosine kinases^3^. Hence, identification of cell surface proteins of HCC is necessary to broaden the potential of targeted therapy. Although cell surface biotinylation followed by proteomic analysis of enriched proteins offers a useful strategy to screen differentially expressed targets in many cancers, a thorough investigation is lacking for HCC^4^,^5^. Here we utilized surface biotinylation to enrich plasma membrane proteins in a stable isotope labelled amino acids in culture (SILAC)^6^-based quantitative proteomic strategy in Hep3B hepatoma cell line and non-tumorigenic liver MIHA cells to identify differentially expressed proteins.

Among many proteins showing enriched expression in Hep3B versus MIHA cells, we have focused and characterized Agrin in detail as histopathological analyses indicated its accumulation in liver cirrhosis and induced HCC, cholangiocarcinomas and malignant HCC lesions without a defined role^7^,^8^,^9^. Splice variants of Agrin, a ~210-kDa proteoglycan, are expressed either as membrane protein or secreted in extracellular matrix (ECM)^10^,^11^. Neural Agrin clusters acetylcholine receptors at synaptic clefts and maintains functional neuromuscular junctions^12^,^13^,^14^. Agrin is expressed in several tissues including muscles and neurons^10^. Agrin binds to Lipoprotein-related receptor 4 (Lrp4), a member of low density lipoprotein receptor (LDLR) family and mediates muscle-specific receptor tyrosine kinase (MuSK) signalling at neuromuscular junctions^15^,^16^. Neural and muscle Agrins also bind laminin in the ECM, which is involved in cytoskeletal rearrangements and neuronal outgrowths with mechanisms remaining unclear^17^,^18^,^19^.

Integrins and focal adhesion (FA) kinases (FAKs)-associated microenvironment, membrane protrusions and degradation of ECM are not only essential for cancer cell invasion, epithelial–mesenchymal transition (EMT) and metastasis but also for promoting tumour growth^20^,^21^,^22^,^23^,^24^,^25^,^26^,^27^. However, not many ECM proteins except collagen can modulate invasiveness and metastasis^23^, and the role of Agrin in regulating oncogenesis is unknown.

Herein we report that Agrin promotes liver carcinogenesis as an ECM sensor regulating cell proliferation, motility, invasiveness, FA integrity and EMT through Lrp4-MuSK signalling axis. *In vivo*, short hairpin RNA (shRNA)-mediated Agrin depletion or function blocking antibodies suppressed oncogenic signalling and liver tumorigenesis. Collectively, our findings establish a role of Agrin upregulation in HCC and suggest it as an attractive therapeutic target.

## Results

### SILAC proteomics identifies differentially expressed proteins

**Figure 1:**
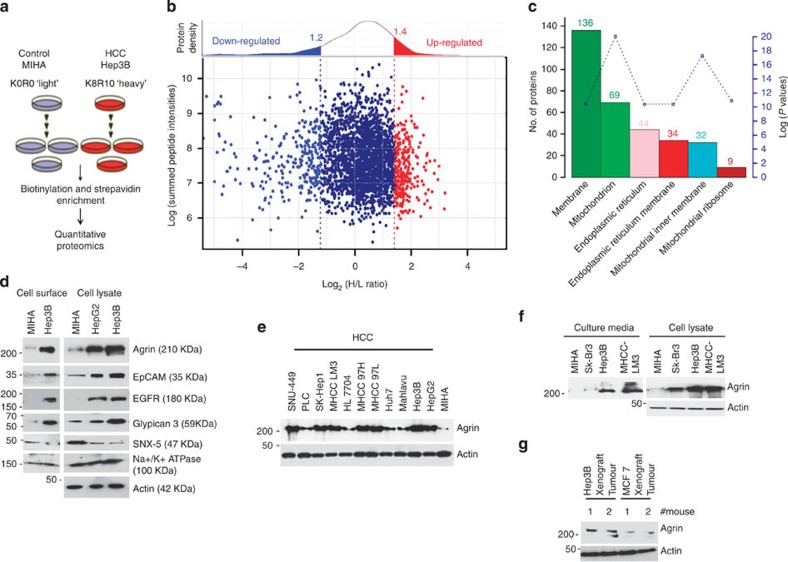
Identification of Agrin as an overexpressed cell surface protein in HCC cell lines. (**a**) Schematic workflow of SILAC mass spectrometry-based analysis of cell surface proteins, where MIHA and Hep3B cells are labelled with ‘light’ and ‘heavy’ amino acids, respectively, and vice-versa. (**b**) The ratio intensity plot representing protein fold change (SILAC ratio versus corresponding summed peptide intensity distribution) and protein density plot (upper panel). Red, blue and navy-blue cluster indicates up-, down- and unregulated proteins. (**c**) Gene ontology Cellular Component analysis of upregulated proteins in Hep3B cells. The scale bar (*y* axis right hand side) indicates the *P* values, while the numbers on top of each bar denotes the abundance of identified proteins in the particular cellular component. (**d**) Biotinylated cell surface proteins and total cell lysates from the indicated cell lines were analysed by western blot analysis using the indicated primary antibodies. (**e**) Western blot analysis of Agrin expression in non-tumorigenic MIHA and various HCC cell lines. β-Actin was used as loading control. (**f**) Conditioned medium from serum-starved (12 h) cell lines were concentrated and precipitated with trichloroacetic acid and analysed by western blot for secreted Agrin. Total cell lysates from the same cell lines were also probed for Agrin with β-actin as a loading control. (**g**) Levels of Agrin in mouse xenografts of indicated cell lines were analysed by western blot using an Agrin-specific monoclonal antibody. β-Actin served as loading control. H/L, heavy/light.

To identify potential diagnostic and/or therapeutic targets differentially expressed in HCC, biotinylated cell surface proteins enriched for plasma membrane fractions were affinity purified with streptavidin sepharose beads. Compared with non-tumorigenic liver MIHA cells, we observed a significant number of proteins enriched in Hep3B and/or HepG2 (HCC) cell lines (Supplementary Fig. 1a). The enrichment of plasma membrane fractions were confirmed by examining the distribution of a membrane marker (integrin β1) and cytosolic marker (Rho-GDI; Supplementary Fig. 1b). For quantitative evaluation, we combined surface biotinylation and extraction of cell surface proteins and identified them by SILAC-based quantitative mass spectrometry (Fig. 1a). To increase the confidence of protein quantification, only proteins with at least two ratio counts were considered for follow-up analysis. A protein density plot was generated using the ratios of those quantified proteins to determine the thresholds for clustering differentially expressed proteins. Using 10%, 90% and in-between quantile-based thresholds, the proteins were categorized as down-, up- and unregulated proteins, respectively (Fig. 1b). In this study, we focussed on proteins that are overexpressed in Hep3B cells and are potential candidates overexpressed in liver carcinoma (red cluster, Fig. 1b). As such, the upregulated protein cluster (Supplementary Table 1) was subjected to Gene Ontology Cellular Component analysis that revealed that >50% of upregulated proteins are enriched as plasma membrane proteins (Fig. 1c and Supplementary Table 2). The overexpressed proteins including epidermal growth factor receptor, glypicans and epithelial cell adhesion molecule (EpCAM) have widespread roles in numerous cancers, thereby supporting the authenticity of our SILAC screen. Proteoglycan Agrin was also identified in this screen as an overexpressed surface protein in Hep3B cells (Supplementary Tables 1 and 2).

### Validation of selected set of identified proteins

To independently confirm our mass spectrometry findings, biotinylated surface proteins in MIHA and Hep3B cells were affinity purified and the expression levels of selected candidate(s) were analysed by immunoblot analysis. Consistent with our SILAC observations, Agrin, EpCAM, epidermal growth factor receptor and glypican-3 showed increased cell surface expression in Hep3B compared with MIHA cells (Fig. 1d). Moreover, higher expression of these proteins was also evident in the whole-cell lysates of HepG2 and Hep3B cell lines (Fig. 1d). Conversely, sorting nexin-5 (SNX5), a protein indicated to be downregulated in Hep3B cell surface has reduced expression in both surface-enriched fraction and total cell lysate (Fig. 1d). The sodium/potassium ATPase (Na^+^/K^+^ ATPase), a plasma membrane protein and many other transporter proteins with unaffected SILAC ratios did not show significant change between MIHA and Hep3B cells in western blot analysis (Fig. 1d). β-Actin levels indicate similar loading of whole-cell lysates (Fig. 1d).

### Agrin is overexpressed and secreted in HCC cell lines

Among the identified molecules, we considered Agrin as an attractive target in HCC owing to its accumulation in cirrhotic liver and HCC but with little known roles^7^,^8^,^9^. Compared with MIHA cells, Agrin was well expressed in a panel of HCC cell lines with relatively higher levels in metastatic MHCC-97H, MHCC-LM3, Sk-HEP-1 and SNU-449 cells (Fig. 1e). Since secreted neural Agrin aggregates acetylcholine receptors, we next examined whether it is secreted in cancer cell lines and hence can potentially act as a biomarker. Supernatants of HCC cell lines (MHCC-LM3 and Hep3B), a breast cancer cell line SkBr-3 and control MIHA cells were tested for Agrin secretion. Indeed, secreted Agrin was high in HCC cell culture supernatants, low in SkBr-3 and hardly detectable in MIHA cells (Fig. 1f).

*In vivo,* mouse xenografts also showed a higher expression of Agrin in the liver (Hep3B) tumours compared with MCF7 cell breast carcinoma (Fig. 1g). These results show an elevated expression and secretion of Agrin in HCC cell lines and Hep3B xenografts.

### Lipid raft-enriched Agrin is constitutively internalized

Reported lipid raft localization of neural Agrin prompted us to examine the exact membrane localization of Agrin in HCC cell lines^28^. Indeed, the bulk of cell surface-bound Agrin is localized to caveolin-1- and flotillin-1-enriched lipid raft membranes, while a subpopulation of it was associated with endosomal and/or high-density fractions marked by Rab5 and CD-71, respectively (Supplementary Fig. 2a). Analysis of membrane and soluble fractions also revealed robust Agrin levels in Hep3B compared with MIHA cells (Supplementary Fig. 2b). The soluble Agrin may represent those loosely associated with endosomal membranes and/or secreted. To test constitutive Agrin internalization, an Agrin antibody internalization assay was performed. At 4 °C, Agrin antibody at cell surfaces was colocalized with cholera toxin-B (CTxB), which binds monosialogangliosides in lipid raft membranes (Supplementary Fig. 2c,d, first panel). After 5 min incubation at 37 °C, Agrin antibody was co-internalized with CTxB coupled with coherent signal intensity overlaps projected towards intracellular compartments (Supplementary Fig. 2c,d, second panel) and were in internal organellar compartments by 30 and 40 min (Supplementary Fig. 2c,d, third and fourth panels, respectively). Similarly, internalized Agrin antibody was observed in EEA-1-positive early endosomes from 5 min till the observed 30 min (Supplementary Fig. 2e,f), suggesting that secreted and cell surface Agrin is dynamically endocytosed, consistent with its identification by surface biotinylation and SILAC as a surface-enriched protein in HCC cells.

### Critical role of Agrin in cellular proliferation

**Figure 2:**
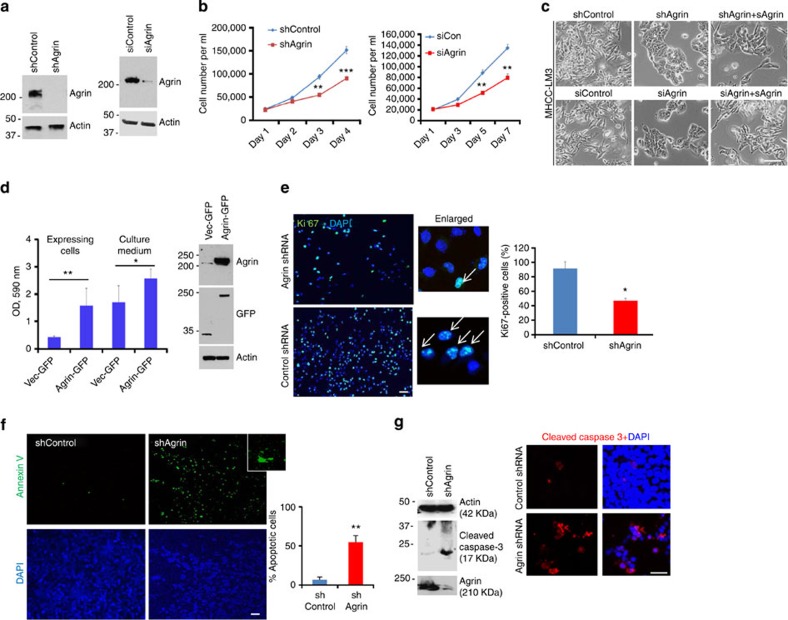
Agrin knockdown affects cell growth and apoptosis. (**a**) Western blot in MHCC-LM3 cells transduced with control and shAgrin expressing lentiviruses or transfected with control or siRNA against Agrin. β-Actin was used as a loading control. (**b**) Cellular proliferation of control and Agrin knockdown MHCC-LM3 cells when passaged in the indicated days. Error bars represent s.d. of means of three independent experiments performed in triplicates (**P value*=*0.002 and ***P=0.0004, respectively, Students *t*-test). (**c**) Bright-field microscopy images of control and Agrin-depleted MHCC-LM3 cells either untreated or treated with soluble recombinant Agrin (20 μg ml^−1^) for 1 day. Scale bar, 10 μm (**d**) MIHA cells expressing either control or Agrin-GFP were subjected to cell proliferation assay after 72 h post transfection (first two bars). Conditioned media from the above transfected cells were incubated with naive MIHA cells for 1 day and subsequently analysed for cell proliferation assay (third and fourth bars). Error bars represent s.d. of means for three independent experiments performed in triplicates (**P value*=*0.003,*P value*=*0.02, Student’s *t*-test). Western blot analysis of Agrin, GFP expression and β-actin (as loading control) are shown. (**e**) Control shRNA and shAgrin-transduced MHCC-LM3 cells were immunostained with Ki67 and counterstained with DAPI. Representative confocal images are shown. Arrows in the enlarged panel indicate actively proliferating cell nuclei. Scale bar, 10 μm. Quantitative plot depicting the percentage Ki67-positive cells in at least five different microscopic fields and error bars represent s.d. of means (*P value*=*0.01, Student’s *t*-test). (**f**) shControl or shAgrin MHCC-LM3 cells were analysed using Annexin V apoptosis staining kit. Representative images co-stained with DAPI are shown and enlarged area inset depicts a typical Annexin V-stained apoptotic cell. Quantitative plot depicting the percentage of apoptotic cells in at least five different microscopic fields is shown. Scale bar, 10 μm. Error bar represents s.d. (**P value=0.005, Student’s *t*-test). (**g**) Western blot analysis of cleaved caspase-3 in shControl and shAgrin MHCC-LM3 cells. The blots were stripped and probed for Agrin and β-actin. Immunofluorescence analysis for cleaved caspase-3 (red) and DAPI (blue) in control and Agrin shRNA-transduced MHCC-LM3 cells. Scale bar, 10 μm.

To characterize the functional role of Agrin in HCC, we depleted Agrin either by stably transducing with Agrin shRNA-expressing lentiviruses or transfecting a short interfering RNA (siRNA) pool targeting Agrin in highly metastatic MHCC-LM3 and Hep3B cells. Like Hep3B cells, cell surface expression of Agrin in MHCC-LM3 cells was significantly higher than MIHA cells (Supplementary Fig. 3). Agrin knockdown was very efficient in HCC cell lines (Fig. 2a and Supplementary Fig. 4a). Compared with the control cells, Agrin depletion significantly reduced cellular proliferation rates by 42% (Fig. 2b and Supplementary Fig. 4a) and converted the elongated control cells to a cobblestone shape with enhanced cell apposition, similar to MIHA cells (Fig. 2c and Supplementary Fig. 4b). This morphological change was substantially reversed by addition of soluble recombinant Agrin (Fig. 2c). In addition, MIHA cells overexpressing Agrin-green fluorescent protein (GFP) showed enhanced proliferation than those expressing vector alone (Fig. 2d). Interestingly, culture media from Agrin overexpressing cells also promoted growth when incubated with naive MIHA cells (Fig. 2d), supporting the notion that secreted Agrin is the functional form. Significant reduction (~41%) of proliferation marker Ki67 labelling in Agrin knockdown cells is consistent with reduced proliferation observed upon Agrin depletion in HCC cells (Fig. 2e and Supplementary Fig. 4c).

We next examined whether growth arrest due to Agrin depletion is also associated with increased apoptosis in addition to decreased proliferation. Compared with control cells, we observed >50% apoptosis in Agrin knockdown cells, as indicated by Annexin V staining (Fig. 2f). Similarly cleaved caspase-3 (a late-phase apoptosis marker) was elevated in Agrin-depleted cells (Fig. 2g and Supplementary Fig. 4d). Hence, suppressing Agrin expression reduced cell proliferation and induced apoptosis in HCC cell lines.

### Agrin functions in cellular motility and invasion

**Figure 3:**
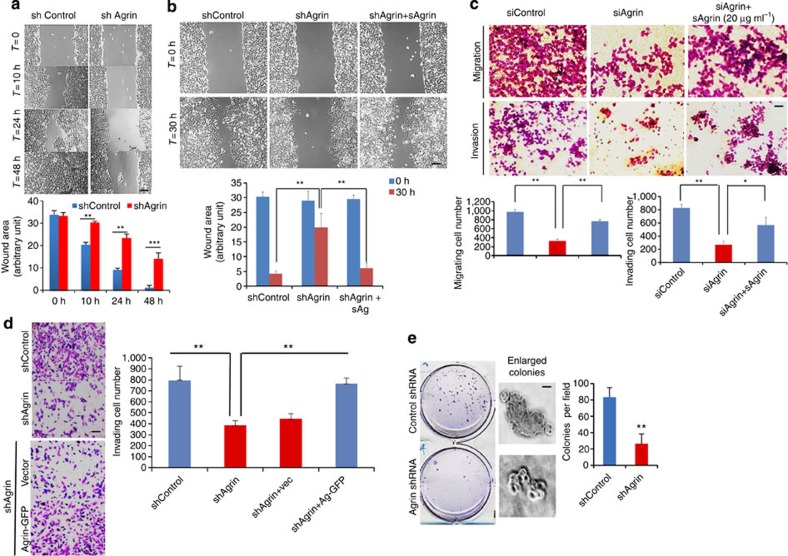
Agrin regulates cancer cell migration, invasion and colony formation. (**a**) Wound-healing assay performed in shControl or shAgrin MHCC-LM3 cells. Representative images are shown at indicated time points and results are quantified using ImageJ software (magnification × 20). The area left unhealed was measured by ImageJ software in arbitrary units and error bars represent s.d for three independent experiments performed in triplicates (**P value*=*0.003, ***P value*<*0.0003, respectively, Student’s *t*-test). Scale bars, 50 μm. (**b**) Wound-healing assay performed in control, Agrin depleted and Agrin-depleted cells treated with soluble recombinant Agrin (20 μg ml^−1^) for 12 h before wound scratch. Representative images are shown at indicated time points and results are quantified as in **a**. Error bars represent s.d. (**P value<0.005, Student’s *t*-test). Scale bar, 50 μm. (**c**) Control or Agrin knockdown MHCC-LM3 cells were either untreated or treated with soluble recombinant Agrin (20 μg ml^−1^) for 12 h and subjected to transwell migration (without Matrigel) and invasion (with Matrigel) assays. Migratory or invasive cells are imaged in a bright-field microscope under × 20 magnification. Results are also quantified using ImageJ software and error bars represent s.d. of means for three independent experiments performed in triplicates (**P value*=*0.001, *P value*=*0.02, respectively, Student’s *t*-test). Scale bar, 50 μm. (**d**) Control or Agrin knockdown MHCC-LM3 cells either expressing GFP-vector or Agrin-GFP were subjected to a Matrigel invasion assay. Cells invading Matrigel were imaged in a bright-field microscope under × 20 magnification. Results were also quantified as in **c**. Error bars indicate s.d. of means of three independent biological replicates performed in triplicates (**P values=0.005 and 0.001, respectively, Student’s *t*-test). Scale bar, 50 μm. (**e**) Control or Agrin knockdown MHCC-LM3 cells were subjected to a soft agar clonogenic assay. Representative pictures for colony growth are shown. Typical cell colonies observed by bright-field microscopy at day 10 of growth in soft agar is also shown. Quantification of the number of colonies is shown. Error bars indicate s.d of mean of three independent experiments performed in triplicates (**P value*=*0.004, Student’s *t*-test). Scale bars, 50 μm.

We next investigated whether morphological and proliferative changes in Agrin-depleted cells affected migratory and invasive behaviour. Agrin depletion severely reduced the migration of MHCC-LM3 cells in a wound-healing assay (Fig. 3a). While shControl cells almost recovered the wound by 48 h, shAgrin cells had significant wound area unhealed (Fig. 3a). Interestingly, soluble recombinant Agrin significantly rescued the migration of Agrin-depleted cells 30 h post-wound scratch (Fig. 3b). Agrin-depleted cells also exhibited significantly lower (>50% reduction) infiltration rates in transwell migration and invasion assays than control cells (Fig. 3c,d and Supplementary Fig. 4e). Again, these migration and invasion defects in knockdown cells were significantly rescued either by supplementation of soluble Agrin (Fig. 3c) or expressing full-length rat Agrin-GFP (Fig. 3d). These results cumulatively suggest that the observed phenotype in Agrin knockdown cells is specifically attributed to reduced Agrin levels. To further demonstrate a role of Agrin in liver tumorigenesis, anchorage-independent growth of control and Agrin knockdown cells was assessed. As expected, Agrin-depleted cells significantly reduced the colony formation by ~65% compared with control cells (Fig. 3e). Compared with well-developed colonies observed in control cells, shAgrin cell colonies were fewer and relatively smaller in size (Fig. 3e, enlarged panel). Therefore, these results suggest that Agrin is critical for oncogenic property of liver cancer cells.

### Agrin controls membrane ruffling and invadopodia

**Figure 4:**
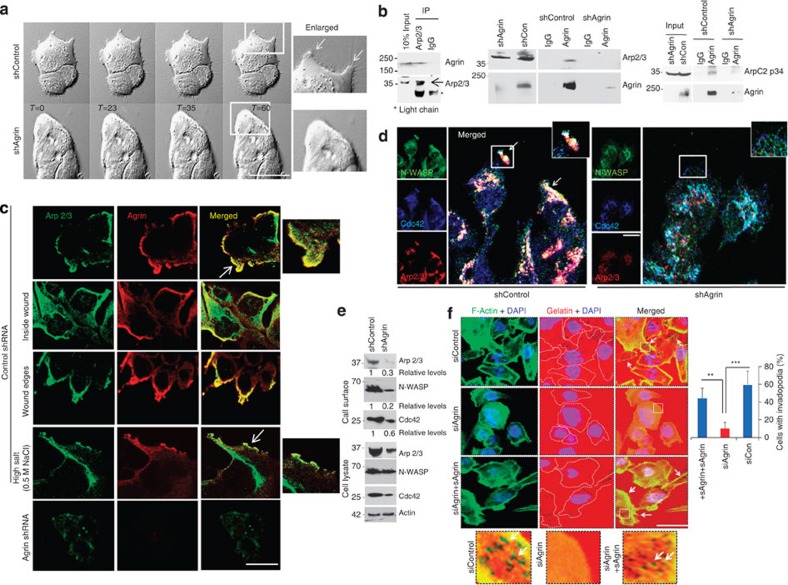
Agrin regulates ruffling and invadopodia in HCC cell lines. (**a**) Representative live-cell time-lapse differential interference contrast images of control and Agrin knockdown cells at indicated time frames (in seconds) are shown. Scale bar, 10 μm. Boxed area is represented as enlarged panels. Arrows indicate membrane ruffles/protrusions. (**b**) MHCC-LM3 cells IP using Arp2/3 subunit 1B (left panel) or Agrin antibodies (middle and rightmost panels) and IgG were analysed by western blotting for Agrin, Arp2/3 subunits 1B or ArpC2p34. The blot was stripped and re-probed for Arp2/3 or Agrin. Thirty micrograms (10%) of whole-cell lysate were used as input control. Lysates from Agrin knockdown cells (right panel) were used as negative control. (**c**) Immunofluorescence analysis of control and Agrin knockdown MHCC-LM3 cells 12 h post scratch assay using goat Arp2/3 antibody and mouse Agrin antibody. Representative confocal images are shown. Panels (ii and iii) represent images of cells within the wound area and edges of wound, respectively. For panel (iv), cells were washed two times with high salt wash buffer (0.5 M NaCl) for 10 min at RT before fixation and immunostaining. Enlarged Z sections were performed at the leading edge of membrane ruffles marked by white arrows. Scale bar, 10 μm. (**d**) Cells were processed similarly as in **c** and immunostained with mouse anti-WASP, rabbit anti-Cdc42 and goat anti-Arp2/3. Boxed area is represented as enlarged panel inset. Scale bar, 10 μm. (**e**) Biotinylated surface proteins from control or Agrin knockdown MHCC-LM3 cells were analysed by a western blot for indicated proteins. Total cell lysate was also western blotted and β-actin served as a loading control. (**f**) Control and Agrin siRNA-transfected cells either untreated or treated with soluble Agrin (20 μg ml^−1^) for 12 h were cultured on Cy3-Gelatin for another 12 h, fixed and then stained with F-actin and DAPI. Representative confocal images are shown and invadopodia was quantified using ImageJ. Boxed areas are represented as enlarged lower panels. Arrows indicate F-actin-enriched degraded gelatin areas. Scale bar, 10 μm. At least five different fields were quantified and error bars indicate the s.d. of means of at least three independent experiments (***P value*=*0.0005, **P value*=*0.004, respectively, Student’s *t* test).

Loss of cellular invasion upon Agrin depletion associated with altered membrane ruffling and/or invadopodia are hallmarks of invasive cancer cells^29^,^30^. Dorsal ruffles and protrusions observed in control cells were markedly reduced upon Agrin depletion (Fig. 4a, and Supplementary Movies 1 and 2). Since these invasive structures are largely governed by association of actin-related Arp2/3, N-WASP and Cdc42 complex near cell surface^31^, we deciphered whether Agrin is involved in associating Arp2/3 subunits to the inner leaflet of cell membrane to regulate ruffling. Co-immunoprecipitation (co-IP) with either Arp2/3 (subunits 1B and ArpC2p34) or Agrin antibody revealed a specific interaction of Agrin with Arp2/3 complex (Fig. 4b). As Arp2/3 and Agrin are distributed on the inside and outside of the plasma membrane, we interpret their co-IP as indirect interaction most likely mediated by transmembrane protein complexes. Agrin and Arp2/3 subunit 1B colocalized at the leading edge of membrane ruffles in a group of migratory cells 12 h post a wound scratch assay (Fig. 4c, panel i). Agrin similarly colocalized with Arp2/3 within dense cells and towards the edge of the wound, ruling out the possibility of cell density or wound stress mediated colocalization between the two proteins (Fig. 4c, panels ii,iii). High salt wash that removes peripherally associated proteins, partially affected the colocalization at the leading edge of membrane ruffles (Fig. 4c, panel iv). In contrast, Agrin-depleted cells had greatly reduced Arp2/3 at leading edges (Fig. 4c, panel v). Further, Arp2/3, N-WASP and Cdc42 colocalized at leading edges of migrating control cells that were greatly hampered by Agrin depletion (Fig. 4d). In addition, association with the inner leaflet of cell membrane and total protein levels of Arp2/3, N-WASP and Cdc42 were also reduced by 70%, 80% and 40%, respectively, upon Agrin depletion (Fig. 4e).

As invadopodia are distinct from membrane ruffles in being protrusive matrix-degrading structures on the ventral surface of cancer cells enriched with F-actin and cortactin in addition to the above-mentioned markers^32^,^33^, we examined Agrin’s role in invadopodia formation by a gelatin-invadopodia assay simulating degradation of ECM. F-Actin-enriched invadopodia visualized by gelatin-degrading activity were abundant on the ventral surfaces of control cells (Fig. 4f, top panels). Significantly, invadopodia were greatly reduced in Agrin-depleted cells (Fig. 4f, middle panels). Addition of soluble Agrin restored the invadopodia formation in knockdown cells (Fig. 4f, bottom panels). Moreover, Agrin-cortactin clusters that localized to invadopodia and degraded matrix sites were diminished upon Agrin depletion (Supplementary Fig. 5a,b). Together, these results suggest that Agrin governs Arp2/3 complex-dependent ruffling and invadopodia formation in HCC cells.

### Agrin affects EMT and integrin-FA signalling

**Figure 5:**
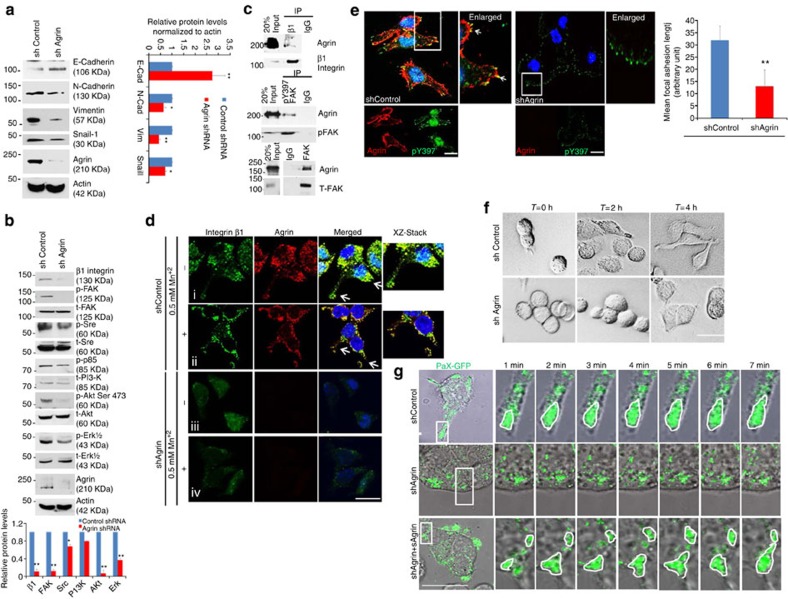
Agrin as an ECM sensor for EMT and FA integrity. (**a**,**b**) Total lysates of control or Agrin shRNA MHCC-LM3-transduced cells were analysed by western blots using antibodies against the indicated proteins. Quantification of proteins normalized to actin (**a**) or respective total protein levels (**b**) are depicted graphically. Error bars represent s.d. (*P value<0.04, **P value<0.002, Student’s *t*-test). (**c**) MHCC-LM3 cell lysates were IP with either rabbit IgG or anti-integrin β1 (upper panel), anti-FAK pY397 (middle panel) or anti-total FAK (T-FAK) antibody (lower panel) and western blotted for Agrin. The blot was stripped and re-probed for integrin β1 (upper panel), pFAK (middle panel) or T-FAK (lower panel). Sixty micrograms (20%) of whole-cell lysate were used as input control. (**d**) Control or Agrin-depleted cells were either mock or pre-treated with 0.5 mM MnCl_2_ solution in complete media for 30 min at 37 °C to activate FAs, washed and processed for immunofluorescence using rabbit integrin β1 and mouse Agrin antibodies. Representative images co-stained with TO-PRO3 are shown. White arrows indicate the enlarged area of cells where Z section was performed. Scale bar, 10 μm. (**e**) Control or Agrin-depleted MHCC-LM3 cells were immunostained using mouse anti-Agrin and rabbit anti-FAK (pY397) antibodies. Representative confocal images co-stained with TO-PRO3 are shown. Arrows indicate colocalization and boxed areas are represented as enlarged panels. FA length was analysed by ImageJ software and at least 10 different fields were chosen for analysis and data represents mean±s.d. (**P value*=*0.005, Student’s *t*-test). Scale bar: 10 μm. (**f**) Control or Agrin-depleted MHCC-LM3 cells plated on fibronectin (10 μg ml^−1^)-coated dishes. Morphological changes analysed by time-lapse differential interference contrast microscopy live-cell imaging at indicated time points. Scale bar, 10 μm. (**g**) Paxillin-GFP expressing control, Agrin-depleted MHCC-LM3 cells alone or supplemented with 10 μg ml^−1^ sAgrin for 1 day were plated on fibronectin-coated dishes. Three hours post plating, cells were imaged live for 7 min at an interval for 1 min. Representative time-lapse series of boxed region is enlarged and shown. Scale bar, 10 μm.

Enhanced cohesiveness of Agrin-depleted cells, in the context that vimentin is one of the proteins enriched in HCC cancer cells that was recovered in our SILAC analysis likely due to interaction with other surface proteins (Supplementary Table 1), suggests that Agrin may be involved in maintaining mesenchymal property of HCC cells. Consistently, along with reduced mRNA levels of Arp2/3, reduction of vimentin mRNA was also observed in Agrin-depleted cells (Supplementary Fig. 6a). Therefore, we tested the hypothesis that Agrin might have a role in vimentin’s association with plasma membrane. As shown, co-IP with an antibody against vimentin co-recovered Agrin from Hep3B cells but not in MIHA cells (Supplementary Fig. 6b). Agrin depletion increased epithelial marker E-cadherin by 2.7 folds but reduced mesenchymal markers N-cadherin, vimentin and Snail-1 by 37%, 44% and 30%, respectively (Fig. 5a). Elevated E-cadherin levels coupled with reduced vimentin in Agrin-depleted cells was reversed by addition of soluble Agrin (Supplementary Fig. 6c,d), suggesting that secreted Agrin alters EMT markers and regulates their association with inner cell membrane (Supplementary Fig. 6e). Similar results were also observed upon siRNA-mediated Agrin depletion with no effect on glypican-3 (Supplementary Fig. 6f). These lines of evidence support a role of Agrin in maintaining mesenchymal characteristics of HCC cells.

Mechanistic investigation of Agrin’s role in modulating the integrin-FAK pathway instrumental in recruiting mesenchymal markers to cell surface and regulating membrane protrusions^34^,^35^ revealed that Agrin depletion markedly reduced FA proteins integrin β1 and pY397 FAK by 85% and 88%, respectively (Fig. 5b). Addition of soluble Agrin restored pY397 FAK level (Supplementary Fig. 6d). In contrast, Agrin depletion had lesser inhibition on pSrc and pPI3-K signalling (Fig. 5b). Notably, Agrin knockdown also reduced pAkt and pErk1/2 signalling by 87% and 61%, respectively (Fig. 5b and Supplementary Fig. 6f), likely accounting for reduced cell proliferation. Furthermore, an interaction between Agrin and integrin β1 was observed (Fig. 5c upper panel and Supplementary Fig. 7a). Agrin was co-IP with activated (pY397) and total FAK (Fig. 5c middle and lower panels, and Supplementary Fig. 7b). In addition to significant colocalization observed without stimulation, activation of integrin and FAs by Mn^+2^ led to an increased Agrin clustering in FAs (Fig. 5d, panels i and ii). In contrast, integrin β1 appeared dispersed and not restricted to FA in Agrin-depleted cells (Fig. 5d, panel iii). Even Mn^+2^ stimulation in these cells failed to activate the integrin–adhesion complexes (Fig. 5d, panel iv), suggesting that Agrin associates with FAs and its depletion affects FA integrity.

### Agrin as ECM sensor regulating FAs and cell attachment

To address how Agrin provides ECM cues to regulate FAs, we first examined its effect on FA integrity. Control cells exhibited Agrin and phospho-Focal adhesion kinase (pFAK) (pY397) colocalization while Agrin-depleted cells had reduced pFAK and FA lengths similar to disrupted FAs induced by myosin IIA bleb inhibitor blebbistatin^35^ (Fig. 5e and Supplementary Fig. 8a). In elongated appearing control cells, pFAK colocalized with other FA components p-Paxillin (Tyr118) and vinculin at activated FAs (Supplementary Fig. 8b,c, left panels i). Agrin-depleted cells were circular and widespread with disrupted FAs (Supplementary Fig. 8b,c, left panels ii). Since defect in cell morphology correlates with poor FA alignment and loss of invasiveness^36^, we further investigated whether Agrin depletion induced FA disruption affected cell attachment. Immediately, post plating on fibronectin-coated dishes, control and Agrin knockdown cells appeared rounded (Fig. 5f, *T*=0 h panel). After 2 h, control cells started to elongate, whereas Agrin-depleted cells remained circular (Fig. 5f, *T*=2 h panel). At 4 h post plating, both control and Agrin-depleted cells attached; the former typically elongated with aligned FA activity, whereas the latter widespread with poor FA activity (Fig. 5f, *T*=4 h panel and Supplementary Fig. 8d). The cell elongation ratio was 3.53 and 1.54 μm for control and Agrin knockdown cells, respectively (Supplementary Fig. 8d). Furthermore, at 3 h post plating, control cells expressing Paxillin-GFP had well-defined FAs that appeared fragmented upon Agrin depletion (Fig. 5g, and Supplementary). This FA defect was substantially rescued by exogenous Agrin treatment (Fig. 5g and Supplementary Movie 5), confirming the specific role of Agrin in controlling FA integrity.

### Agrin promotes FAK dependant ECM degradation

Since the defined pFAK-cortactin cluster length, colocalization in migrating cells 16 h post scratch assay and association as analysed by co-IP were hampered upon Agrin depletion (Supplementary Fig. 9a,b), we explored whether Agrin, FAK or their dual involvement regulates invadopodia formation and matrix degradation. Consistently, some activated FA components (pY397FAK and p-Paxillin) localized near or within areas of degraded matrix in control cells as reported before^37^,^38^ (Supplementary Fig. 9c–e). Interestingly, Agrin depletion affected FA activity and prevented matrix degradation (Supplementary Fig. 9c–e). Although FAK knockdown reduced invadopodia and matrix degradation by ~50%, Agrin and FAK double depletion had significantly greater inhibition (up to 82%; Supplementary Fig. 10a,b). Moreover, MIHA cells with low endogenous Agrin levels had negligible invadopodia and matrix degradation despite the presence of pFAK (Supplementary Fig. 10c). Interestingly, supplementing Agrin increased invadopodia by ~50% with pFAK being observed within some degraded areas. These matrix degradation were substantially reduced in FAK-depleted cells treated with Agrin (Supplementary Fig. 10c), suggesting that Agrin functions through FAK-dependent ECM degradation.

### Cumulative impact of FAs and Agrin on tumour cell growth

The role of FAK in promoting tumour growth is reported in various cancers such that FAK inhibitors have been proposed as anti-tumorigenic agents^24^,^25^,^26^,^27^. To establish that Agrin-mediated regulation of FAs affects tumour cell growth, we used FAK inhibitor PF-562271 that reduced pFAK activity and spheroid culture diameter in a dose-responsive manner (Supplementary Fig. 11a,b). Although inhibitory effects of 0.8 μM FAK inhibitor alone in spheroid and soft agar growth were slightly lesser than that of Agrin depletion, PF-562271 treatment in Agrin-depleted cells cumulatively had significantly greater anti-growth effects (Supplementary Fig. 11b,c), suggesting the importance of Agrin-FAK synergism in *in vitro* tumorigenesis.

### Exogenous Agrin rescues defects of Agrin knockdown cells

**Figure 6:**
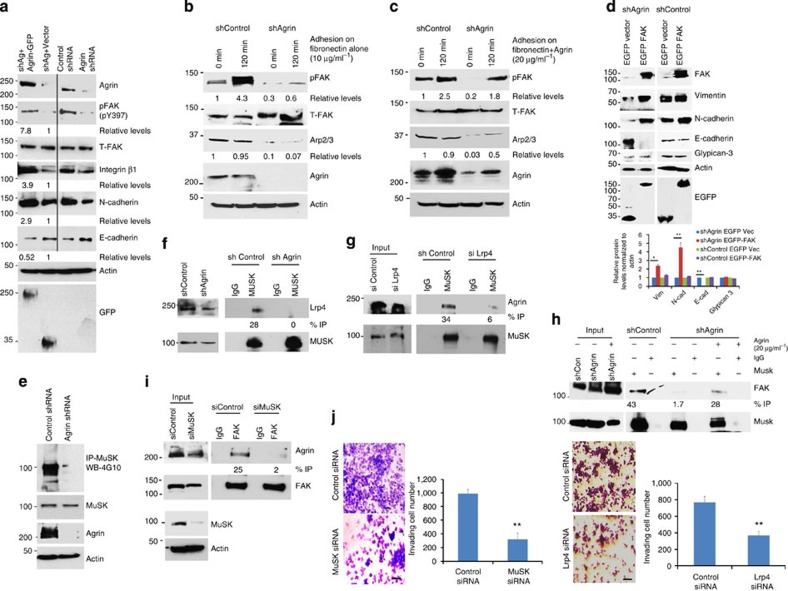
Oncogenic property of Agrin-Lrp4-MuSK axis is primarily mediated by FAK. (**a**) Control and Agrin-depleted MHCC-LM3 cells transfected with GFP-vector or full-length Agrin-GFP and total cell lysates were western blotted for indicated proteins. (**b**,**c**) Serum-starved control or Agrin-depleted MHCC-LM3 cells were trypsinized and plated on fibronectin alone (**b**) or fibronectin and 20 μg ml^−1^ recombinant Agrin (**c**)-coated surface. Total lysates were analysed by western blots for indicated proteins. β-Actin served as loading control. (**d**) Control or Agrin-depleted MHCC-LM3 cells transfected with EGFP-vector or EGFP-FAK and total cell lysates were Western blotted for the indicated proteins. Quantification of proteins normalized to actin is depicted. Error bars represent s.d. (*P value<0.03, **P value<0.002, Student’s *t*-test). (**e**) Control or Agrin-depleted MHCC-LM3 cells were IP with MuSK antibody and western blotted using tyrosine-specific 4G10 antibody. The blot was re-probed for MuSK, Agrin and β-actin (as loading control). (**f**) Control or Agrin-depleted MHCC-LM3 cells were IP with rabbit MuSK or control IgG antibodies and Western blotted for Lrp4. The blot was stripped and re-probed for MuSK. Thirty micrograms (10%) cell lysates were used as input controls. (**g**) Control or Lrp4-depleted MHCC-LM3 cells were IP as in **f**, western blotted for Agrin. The blot was stripped and re-probed as in **f**. (**h**) Cells same as in **f** were either untreated or treated with 20 μg ml^−1^ recombinant Agrin for 12 h and IP with rabbit IgG or MuSK, and western blotted for FAK. The blot was stripped re-probed for MuSK expression. Thirty micrograms (10%) of lysate was used as input control. (**i**) Control or MuSK-depleted cells were IP with FAK antibody and western blotted for Agrin. Thirty micrograms (10%) of protein lysate was used as input control. The blot was stripped re-probed for FAK expression. Lower panels represent MuSK knockdown and β-actin as loading control. (**j**) Control, MuSK and Lrp4 knockdown MHCC-LM3 cells were subjected to a Matrigel invasion assay. Representative images under × 10 magnification are shown and results were quantified using ImageJ software. Error bars represent s.d. (**P values*<*0.005 and 0.004, respectively, Student’s *t*-test). Scale bars, 50 μm.

We next rationalized that restoring Agrin should rescue the effects caused by Agrin shRNAs if these phenotypes are primarily attributable to reduced levels of Agrin. Hence, we expressed full-length rat Agrin-GFP (resistant to the shRNA targeting human Agrin) in Agrin knockdown MHCC-LM3 cells, as depicted by Agrin and GFP western blots, respectively (Fig. 6a, first panel and last panel). Compared with vector control, Agrin rescued cells exhibited 7.8 and 3.9 folds increase in activated FAK (pY397) and integrin-β1 levels, respectively, while un-transfected control and Agrin-depleted cells showed reduced pFAK and integrin levels as shown earlier (Fig. 6a). Agrin restoration increased N-cadherin by 2.9 folds and reduced E-cadherin by 48% (Fig. 6a). Compared with control cells, we also observed reduced FAK activity (by 86%) and Arp2/3 levels (by 93%) 2 h post attachment on fibronectin-coated plates upon Agrin depletion (Fig. 6b). Importantly, supplementing Agrin with fibronectin in ECM rescued adhesion defects by increasing pFAK and Arp2/3 levels by 72% and 55%, respectively (Fig. 6c). Moreover, actin polymerization activity by Arp2/3 complex reduced by 44% upon Agrin depletion but was substantially rescued by soluble Agrin (Supplementary Fig. 12). These results cumulatively validate the role of Agrin as an ECM sensor regulating FA and Arp2/3-dependent cell attachment.

### FAK overexpression suppresses Agrin-depleted phenotype

Recent studies indicate that FAK is critical for HCC progression^22^. If the key function of Agrin is to maintain and sustain FAK activity in HCC cells, then the phenotype caused by Agrin knockdown is primarily due to decreased FAK activity. If this is indeed the case, then we should predict that overexpression of active FAK alone could suppress, at least in part, the phenotype caused by Agrin depletion. Accordingly, EGFP-FAKpY397 expression in Agrin-depleted cells restored the mesenchymal markers vimentin and N-cadherin by 2.4 and 4.4 folds, respectively, and decreased E-cadherin by 95% (Fig. 6d, shAg panel). Respective FAK and enhanced-green fluorescent protein (EGFP) expression levels are also shown (Fig. 6d). However, FAK expression in Agrin knockdown cells did not affect glypican-3, suggesting the specificity of FAK to rescue Agrin-depletion-mediated effects (Fig. 6d). We also did not observe any major effects of FAK overexpression in control shRNA-transduced cells (Fig. 6d, shControl panel). Together, these observations reveal a synergistic dependence of Agrin on FAK activity to drive EMT programme and strongly suggest that FAK is a major mediator of Agrin function.

### Agrin–Lrp4–MuSK complex as an oncogenic signalling axis

Since neuronal Agrin containing splice variants within Z exons preferentially activate Lrp4-MuSK signalling^15^,^16^, it is important to investigate whether the observed roles of Agrin in HCC is mediated through Lrp4-MuSK or occurred independently by alternative pathways. Reverse transcription PCR analysis with primers flanking Z exons revealed high expression of Agrin Z transcripts in multiple HCC cell lines (Supplementary Fig. 13a,b). Moreover, Lrp4 and MuSK are also widely expressed in a panel of HCC cell lines (Supplementary Fig. 14a). MuSK and Lrp4 knockdown in MHCC-LM3 cells decreased proliferation and induced morphological changes similar to those observed upon Agrin depletion (Supplementary Fig. 14b,c). As Agrin depletion strongly reduced tyrosine phosphorylation of MuSK (Fig. 6e), we investigated the role of Agrin–Lrp4–MuSK complex in HCC. Interestingly, the co-IP of MuSK and Lrp4 in control cells was compromised in Agrin-depleted cells (Fig. 6f), suggesting that interaction between MuSK and Lrp4 occurs in an Agrin-dependent manner as reported previously^39^. Although Agrin does not directly interact with MuSK^39^,^40^,^41^, its association with Lrp4 stimulated the presence of MuSK in the Agrin–Lrp4 complex, which was decreased by 83% upon Lrp4 depletion (Fig. 6g), indicating that the formation of Agrin–Lrp4–MuSK complex is due to Lrp4-dependent MuSK activation by Agrin. We next deciphered whether Agrin–Lrp4–MuSK complex indirectly signals to FAs. Indeed, IP with MuSK antibody co-recovered FAK, indicating an indirect presence of FAK in the Agrin–Lrp4–MuSK complex, which was reduced by 96% upon Agrin depletion but was partly restored by addition of soluble Agrin (Fig. 6h). In addition, MuSK depletion decreased the interaction of FAK with Agrin by 92% (Fig. 6i). MuSK and Lrp4 depletion also significantly reduced cellular invasion rates by 68% and 52% compared with control cells, respectively (Fig. 6j). Importantly, overexpression of Lrp4 and MuSK in HCC tumours (8 out of 11 HCC patients; Supplementary Fig. 14d) is consistent with the notion that Agrin functions through the Lrp4–MuSK complex to activate FAK in driving the oncogenic programme of HCC cells.

### Agrin’s role in *in vivo* tumorigenesis

**Figure 7:**
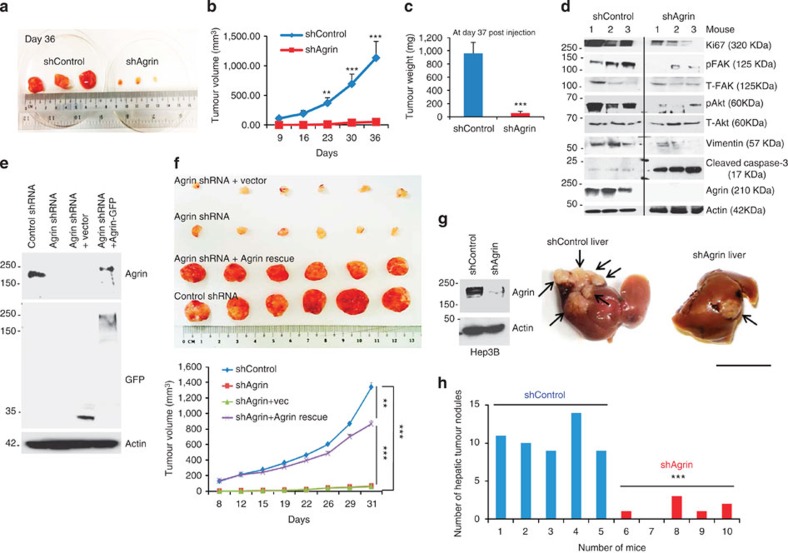
Agrin regulates oncogenic signalling, tumour growth in xenograft and orthotopic HCC models. (**a**) Control or Agrin shRNA-expressing MHCC-LM3 cells (1 × 10^7^ cells per ml) were injected subcutaneously in athymic nude mice to establish tumours. Representative images showing respective xenograft tumours at day 36 post subcutaneous injection (*n*=3 per group). (**b**,**c**) Tumour sizes measured and depicted as tumour volume (**b**) or tumour weight (**c**). Error bars represent s.e.m. (**P values=0.005, ***P values=0.0005, respectively, Student’s *t*-test). (**d**) Western blot analysis in mouse xenografts from **a** showing the expression of indicated proteins. (**e**) Agrin knockdown MHCC-LM3 cells were stably transfected with rat full-length Agrin-GFP or vector-GFP control. Western blot showing the expression of Agrin/Agrin-GFP, GFP expression and β-actin (as a loading control). (**f**) Cells (5 × 10^6^) derived from **e** were subcutaneously injected in nude mice as in **a** to establish tumours. Representative tumours and their volume are depicted graphically; *n*=6 (for each group). Error bar represents s.e.m. (**P value=0.002, ***P value<0.0001, respectively, Student’s *t*-test). (**g**) Western blot analysis for Agrin expression in control shRNA and Agrin shRNA-transduced Hep3B cells with β-actin as a loading control (left panel). Cells (1 × 10^6^) were then intrahepatically inoculated in mice. Representative image of hepatic nodules 6 weeks post-hepatic injection (right panel), *n*=5 (per group). Black arrows indicate hepatic nodules. Scale bar, 10 mm. (**h**) The hepatic tumour nodules (>1 mm in size) in control and Agrin-depleted Hep3B cell injected livers are quantified (***P value=0.0002, Student’s *t*-test).

Having mechanistically deciphered the oncogenic properties of Agrin *in vitro*, we next examined whether silencing Agrin affects tumour development *in vivo*. While mice injected subcutaneously with control MHCC-LM3 cells developed solid tumours >1,000 mm^3^ in volume and weight (at day 36), Agrin-depleted xenografts were significantly smaller in volume (~57 mm^3^) and weight (Fig. 7a–c). Western blot analysis revealed reduced levels of Ki67, pY397FAK, pAkt and vimentin but increased levels of cleaved caspase-3 in Agrin-depleted tumours (Fig. 7d). To validate that the *in vivo* impact is truly due to Agrin silencing, we restored Agrin in knockdown cells by stably expressing a full-length rat Agrin-GFP construct (Fig. 7e). Agrin-GFP rescued cells have significantly increased *in vivo* tumour growth (Fig. 7f). Furthermore, the *in vivo* role of Agrin was also investigated in an orthotopic HCC tumour model as tissue microenvironment has a crucial role in carcinogenesis. Six weeks post intrahepatic inoculation, mice injected with Agrin-depleted Hep3B cells developed significantly lesser hepatic nodules than control cells (Fig. 7g,h). Cumulatively, these results confirm the oncogenic potential of Agrin *in vivo*.

### Agrin antibodies reduce signaling and tumour growth

**Figure 8:**
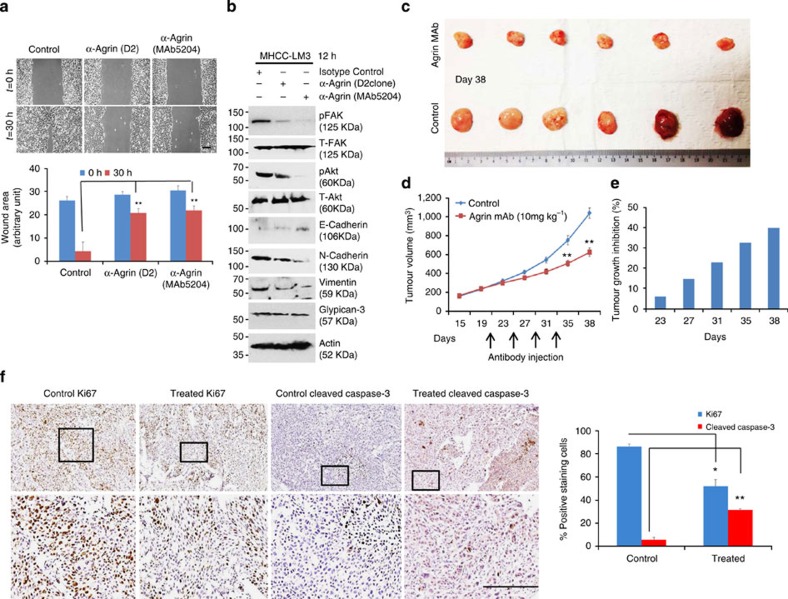
Agrin functional blocking antibodies reduce tumorigenesis. (**a**) MHCC-LM3 cells were pre-treated with indicated Agrin antibodies (10 μg ml^−1^) for 12 h and then a wound-healing assay was performed in the presence of respective antibodies. Representative bright-field images are shown at indicated time points post scratching. Results are quantified using ImageJ software and error bars represent s.d. of mean area unhealed over time (in arbitrary unit) for three independent experiments performed in triplicates (**P value=0.004, Student’s *t*-test). Scale bar, 50 μm. (**b**) MHCC-LM3 cells were pre-treated with Agrin antibodies (10 μg ml^−1^) for 12 h and lysates were subjected to western blot analysis for the indicated proteins. (**c**,**d**) Athymic nude mice were injected subcutaneously with MHCC-LM3 cells (1 × 10^7^ cells per ml) to develop tumours. After the tumours reached a size of ~200 mm^3^, MAb5204 antibody (dose 10 mg kg^−1^) against Agrin (*n*=6 mice) or PBS control (*n*=6 mice) was injected four times at an interval of 4 days. Tumour sizes shown after mice were killed at day 38. Mean tumour volume is represented in **d**. Error bars represent s.e.m. (**P values<0.001, Student’s *t*-test). (**e**) Tumour growth inhibition depicted with respect to PBS control on the days subsequently after antibody injection. (**f**) Immunohistochemical analyses of antibody-treated or PBS-treated control tumours stained for proliferation marker (Ki67) and apoptosis marker (cleaved caspase-3). Representative bright-field images are shown (*n*=3 mice per group). Boxed areas are represented as enlarged panels. At least six different control or antibody-treated tumour sections were analysed and quantified using LEICA image software for Ki67 and cleaved caspase-3 staining positivity. The percentage of positive staining cells are plotted and error bars represent s.d. of mean values of at least six different sections (*P value=0.02, **P value*=*0.003, respectively, Student’s *t*-test). Scale bar, 100 μm.

The necessity of Agrin in tumorigenesis may represent it as a suitable therapeutic target in HCC. Therefore, we characterized two Agrin targeting antibodies (D2 antibody and MAb5204 antibody, respectively). Their antigenic epitopes are mapped within the 20-kDa C-terminal fragment of rat Agrin (Supplementary Fig. 15a) and recognize full-length protein and C-terminal fragment (Supplementary Fig. 15b). Agrin antibodies strongly inhibited Agrin-induced MuSK phosphorylation in differentiated muscle C2C12 and MHCC-LM3 cells, suggesting their functional blocking activities (Supplementary Fig. 15c,d). As such, both antibodies significantly reduced cell migration by ~77% as compared with isotype control (Fig. 8a). Agrin antibodies strongly suppressed pY397FAK, pAKT (Ser473), N-cadherin and vimentin expression while upregulating E-cadherin without affecting glypican-3 levels (Fig. 8b). Since MAb5204 showed greater *in vitro* effects, we examined its inhibitory role on tumour growth in a xenograft model. MAb5204 antibody treatment reduced the growth of pre-established tumours compared with PBS controls with ~40% tumour growth inhibition (Fig. 8c–e). Consistently, treated tumours displayed reduced proliferation (Ki67) by 42% and increased apoptosis (cleaved caspase-3) by 87% (Fig. 8f), suggesting that MAb5204 antibody treatment reduces tumour growth.

### Agrin is frequently overexpressed in HCC patients

**Figure 9:**
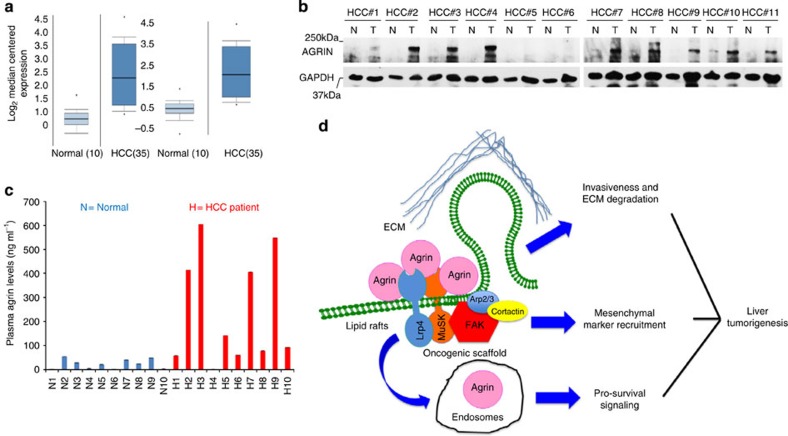
Agrin is frequently overexpressed in HCC. (**a**) Gene expression analysis of Agrin using microarray (Oncomine) on normal liver versus HCC patient data sets. Pre-processed expression levels are Log_2_ normalized and median centred (*P* values=6.22e-6 and 1.22e-7, respectively). Error bars represent s.e.m. (**b**) Western blot analysis for Agrin in a cohort of liver cancer patients in Singapore. GAPDH was used as a loading control. (**c**) Detection of circulating Agrin in the plasma samples of healthy normal individuals and HCC patients by Agrin ELISA. Data represented as mean±s.d. (**d**) A working model describing the role of Agrin in HCC. Overexpression of secreted and cell surface Agrin triggers elevated binding to its receptors Lrp4 and promotes the formation of the Agrin–Lrp4–MuSK signalling complex, which activates FAs (FAK activation), Arp2/3 associated components and cortactin generating ruffling and invadopodia. This ECM sensor activity of Agrin is critical for sustaining FAK activity, cell motility, invasiveness, matrix degradation and subsequent mesenchymal marker recruitment to cell membrane. Internalized Agrin and its complex may also mediate signalling at the endosomal compartments. Cumulatively, these Agrin-mediated events are essential for hepatic tumorigenesis.

To establish a clinical relevance of Agrin’s role in HCC, we analysed HCC patient microarray data sets^42^,^43^. Consistent with our findings, we observed a 3.5- and 3.9-fold upregulation of Agrin (AGRN) mRNA in HCC patients (compared with normal liver tissues) in two independent data sets (Fig. 9a). Western blot analysis of matched normal (non-tumour; N) and HCC (liver-tumour; T) specimens revealed a significant upregulation of Agrin (9 out of 11 HCC patients) in cancer (Fig. 9b and Supplementary Table 3). Similarly compared with normal liver tissues, Agrin overexpression was also observed across various stages of HCC (Supplementary Fig. 16a,b and Supplementary Table 3). Furthermore, circulatory plasma Agrin levels are higher in HCC patients when compared with normal individuals (Fig. 9c and Supplementary Table 3). These results confirm Agrin overexpression in tumour tissues and increased levels of circulating Agrin in HCC.

## Discussion

Multiple lines of evidence presented here identify a unique ECM sensor activity of Agrin in providing oncogenic cues that promote liver tumorigenesis. We demonstrate Agrin’s role in *in vitro* and *in vivo* liver tumorigenesis by stimulating cellular motility, FA integrity and EMT. We also validated the frequent upregulation of Agrin in clinical HCC specimens. Since cell surface proteins are the best known therapeutic targets in most cancers, our results suggest that targeting Agrin may be a potential approach to suppress HCC.

Proteomic screens offer unbiased approach to identify candidates in many cancer types^44^. Since quantitative membrane proteomics is rare in liver cancer, our SILAC analysis identifying differentially expressed cell surface proteins was designed accordingly. Based on our data, Agrin appears to be secreted and enriched in the plasma membrane (especially the lipid rafts) from where it can be actively endocytosed. As Agrin forms a complex with Lrp4-MuSK in HCC cells, it is likely that the endocytosis also occurs as the complex. Such endocytic events are often associated with signal amplification in many cancers^45^.

The EMT programme is activated when invading tumour cells breach through the underlying basement membrane and ECM^46^. However, how the basement membrane components regulate EMT is unknown. Interestingly, Agrin is reported as a basement membrane protein^7^,^28^. Several lines of evidence including changes in cell morphology, reversal of EMT characteristics and loss of invasiveness and clonogenecity upon Agrin knockdown are suggestive of Agrin’s role in tumorigenesis. Conversely, Agrin rescue experiments by either expressing exogenous protein or addition of soluble Agrin restored migration, invasion and mesenchymal characteristics.

The molecular mechanism of Agrin’s action was investigated. First, it was revealed that Agrin coupled to Arp2/3 regulates invadopodia formation required for ECM degradation and invasion. Second, Agrin activates the integrin-FAK pathway and maintains FA integrity. Agrin silencing or antibody treatment hampers FA integrity while Agrin re-expression restores FAs in knockdown cells. Since FAK controls mesenchymal characteristics bestowing adhesion and invasiveness in cancer cells^34^, Agrin-FAK axis probably drives EMT programme in HCC, corroborated by our FAK complementation evidence in Agrin-deprived cells. As integrin-FAK signalling also directs proliferation for metastatic cancer cells^47^, it can be speculated that Agrin provides stimulatory signals to augment and sustain FAK activity and proliferation during tumour growth and extravasation. Instances where Agrin-depleted cells displayed poor FA activity, less invasiveness and synergistic inhibition of the Agrin-FAK pathway having robust growth inhibitory effects shed light to the above hypothesis. Elevated Agrin expression leads to enhanced binding and signalling through Lrp4 and MuSK. Anti-proliferative effects, loss of FA interactions and invasiveness upon Lrp4-MuSK depletion in the presence of Agrin strongly support that Agrin–Lrp4–MuSK complex may indirectly mediate signalling of Agrin to FAs and form a critical oncogenic axis (a working model presented in Fig. 9d). Moreover, reduced activity of Arp2/3 complex driving actin-induced membrane protrusions, FA alignment and migration observed upon Agrin depletion can be significantly reversed by Agrin supplementation in the ECM. Therefore, Agrin serves as the unique link via Lrp4-MuSK between extracellular surface and FAK signalling critical for regulating tumorigenesis.

On the clinical perspective, elevated Agrin levels in HCC patient microarray data sets, tumour tissues and circulation of HCC patients cumulatively emphasize a strong role of Agrin in liver cancer. Agrin depletion hampered oncogenic signalling and tumour growth in xenograft and orthotopic models that is rescued by Agrin re-expression or addition of soluble Agrin, suggesting the tumour initiating and maintenance properties of Agrin. Low Agrin expression in normal livers and the observation that Agrin antibodies reduced pre-established tumour growth offer the possibility of Agrin as a potential therapeutic target by antibody therapy. Moreover, the increased plasma Agrin levels may also be exploited to develop diagnostic strategy for HCC in the future.

In conclusion, we reveal Agrin as an important factor activating and coordinating cellular adhesion, migration and invasiveness of HCC cancer cells. Although the role(s) of surface proteoglycans in promoting liver tumorigenesis are unclear, therapeutically targeting proteoglycans is gaining immense potential in HCC^48^. Therefore, targeting Agrin may augment additional HCC therapeutic strategies in the future. Moreover, our identification of Agrin’s biological role sheds light on the broad cellular mechanisms as to how proteoglycans may regulate HCC.

## Methods

### Cell lines

The human HCC cell lines Hep3B, HepG2 and Huh-7 cells were purchased from American Type Cell Culture (Manassas, VA) and cultured in recommended media. The immortalized non-tumorigenic human hepatocyte cell line MIHA was kindly gifted by Dr J.M. Luk^49^. All other HCC cell lines were routinely maintained in Dr Kam-Man Hui laboratory (National Cancer Center, Singapore). Cells were cultured as per standard conditions described previously^50^. C2C12 mouse myoblasts were cultured in DMEM supplemented with 10% fetal bovine serum (FBS) and were differentiated in DMEM containing 2% horse serum for another 3 days^51^.

### Patient data analysis

The collection of tumour and adjacent normal liver tissues and plasma from HCC patients were approved by the SingHealth Institutional Review Board (CIRB Ref:2007/447/B). All the tissues studied were provided by The SingHealth Tissue Repository. Written informed consent was obtained from all participating patients. Clinical and histopathological data provided to the researchers were rendered anonymous. Normal liver and HCC tissue microarray slides were obtained from US Biomax, Inc. (catalogue no. BC03116). The disease information of patients are provided in Supplementary Table 3.

### Antibodies and reagents

Agrin (D2) and rabbit (H-300), Lrp4, Cdc42, N-WASP (western blot (WB) dilution—1:100) and anti-GFP (WB dilution—1:1,000) antibodies were obtained from Santa Cruz Biotechnology Inc., Santa Cruz, CA), Antibodies against Na+/K+ ATPase, Rab5, integrin β1, cleaved caspase-3, total FAK, cortactin, pSrc, total Src, pPI3-K, total PI3-K, pAkt, total Akt, pERK1/2 and total ERK1/2 were obtained from Cell Signaling Technology (dilution for WB—1:1,000). Antibodies against pFAK pY397, caveolin-1, Ki67, E-cadherin, N-cadherin and Vimentin were purchased from BD Biosciences, San Jose, CA (WB dilution—1:500). PF-562271, glypican-3, flotillin-1, Arp2/3 subunit 1B, pFAK (pY397), Snail-1 and MuSK antibodies were obtained from Abcam (dilution for WB—1:500). Phospho-tyrosine monoclonal antibody 4G10, Agrin monoclonal antibody MAb5204, p34Arc, GFP and EGFP antibodies were obtained from Millipore, Billerica, MA (WB dilutions—1:1,000). Agrin polyclonal rabbit antibody, OKT-9 hybridoma cells producing anti-CD-71 antibody and fibronectin antibody were obtained from Sigma, St Louis, MO. Recombinant rat Agrin protein residues was obtained from R&D systems and My Biosource, Inc, San Diego, CA. Recombinant CTxB, Alexa 488 and 555 conjugated secondary antibodies were from purchased Molecular Probes, Invitrogen, Carlsbad, CA.

### *In vivo* tumorigenesis

Xenograft tumorigenesis: Six- to eight-week-old female nude mice were inoculated subcutaneously in the left and right hind flanks with 1 × 10^7^ cells per ml suspended in Matrigel (1:1). Tumour development was monitored over a period of 30–36 days before the mice were killed for further analysis. Tumour volume (mm^3^) is calculated by the formula *V*=*L* × *B*^2^/2, where *V*=volume of tumour, *L*=length of tumour and *B*=breadth of tumour measured in mm scale. Intrahepatic tumorigenesis was performed as follows^52^: a midline abdominal incision was made to expose the lateral liver lobes. Approximately, 1 × 10^6^ cells were directly delivered into liver parenchyma. Six weeks later, mice were killed and hepatic nodules >1 mm in diameter were counted. All animal care and handling were in compliance with Institutional Animal Care and Use Committee at Institute of Molecular and Cell Biology, A*STAR, Singapore.

### Generation of knockdown and rescue cells

A lentiviral pool encoding shAgrin (catalogue no. sc-29652-V) and scramble shRNA control (catalogue no. sc-108080) were purchased from Santa Cruz Biotechnology Inc. Viral transduction was performed as per manufacturer’s protocol. Agrin shRNAs comprises a pool of three different shRNA hairpins specifically targeting human Agrin. Their sense sequences are 5′-CAGGAGAAUGUCUUCAAGATT-3′, 5′-CGACGUGUGCUGUGAAGAATT-3′ and 5′-CGACCUCUUCCGGAAUUCATT-3′, respectively. Briefly, 30 μl of viral particles was used to infect MHCC-LM3 cells overnight along with 0.5 μg ml^−1^ polybrene in complete media. Next day, medium was changed and cells were re-infected for 2 h and monitored for 2 more days before selection in media containing puromycin (2 μg ml^−1^) for at least two doubling times. After 3 days, knockdown was verified by a western blot analysis using Agrin-specific antibody. Agrin rescue in previously knocked down MHCC-LM3 cells were performed by nucleofection of a full-length rat Agrin-GFP construct that is not targeted by human shRNA sequences as per standard protocol. Forty-eight hours post nucleofection, GFP cells were selected using 400 μg ml^−1^ neomycin (G418) for 10 days. A western blot confirming stable Agrin-GFP expression was performed.

### siRNAs plasmids and transfections

For siRNA-mediated knockdown, ONTARGET *plus* SMARTpool of siRNA against human Agrin (no. L-031716-00-0050), human Lrp4 (no. L-027194-02-0020), human PTK2 (no. L003164-0020) and scramble control (no. D-001210-01-20) were obtained from Dharmacon (Thermo Fisher Scientific). siRNA targeting MuSK (no. AM16704) and ArpC2/p34 (no. AM16708) were obtained from Ambion Life Technologies. Cells were transfected using RNA-iMAX Lipofectamine 2000 (Invitrogen). A western blot analysis verifying target protein knockdown was performed after 72 h post transfection. Full-length rat Agrin-GFP, C-terminal and N-terminal Agrin-GFP were kindly gifted by Dr Matthew P. Daniels, National Institutes of Health, Bethesda, MD, USA^53^. EGFP-FAK and EGFP control vectors were obtained from Addgene.

### Biotinylation and cell surface protein extraction

Cell surface proteins were biotinylated and extracted using the Pierce Cell Surface Protein Isolation kit (Pierce, IL) as per manufacturer’s protocol. Briefly, cells labelled with 0.25 mg ml^−1^ cleavable biotinylation reagent (Sulfo-NHS-SS-Biotin) for 30 min at 4 °C and quenched by adding 2 ml of quenching solution. Post washing, cells were harvested in lysis buffer (Pierce), sonicated in ice for 30 min followed by centrifugation at 10,000*g* for 2 min. Biotin-labelled proteins were affinity purified with streptavidin Agarose and eluted with SDS–polyacrylamide gel electrophoresis (SDS–PAGE) sample buffer (62.5 mM Tris-HCl, pH 6.8, 1% SDS, 10% glycerol and 50 mM dithiothreitol). Flow-through fractions were also analysed.

### SILAC-based mass spectrometry analysis

Hep3B and MIHA cells were cultured in DMEM for SILAC (Thermo Fisher Scientific) supplemented with 10% dialysed FBS either containing normal isotopes of L-lysine-(^12^C_6_^14^N_2_) (K^0^) and L-arginine-(^12^C_6_^14^N_4_) (R^0^) (K^0^R^0^-‘light’) or stable isotope L-lysine-(^13^C_6_^15^N_2_) (K^8^) and L-arginine-(^13^C_6_^15^N_4_) (R^10^) (R^10^K^8^-‘heavy’) for at least six doublings to ensure efficient incorporation of labelled amino acids. Cells were subsequently biotinylated and cell surface proteins were extracted as described above, and equal amount of cell lysates from light (K^0^R^0^) and heavy (R^10^K^8^) were mixed and separated on a 10% SDS–PAGE. Extracted peptides were subjected to LC-Orbitrap MS analysis (Fig. 1a). Equal amounts of protein lysates from heavy- and light biotin-labelled cell lysates were mixed, and 80 μg proteins were separated on a one-dimensional gradient (4–12%) Nu-Page Novex Bis-Tris gel (Invitrogen) and digested with trypsin described previously^54^. The samples were analysed on an Orbitrap XL (Thermo Fisher Scientific) full-scan mass spectra. Identification and quantification of peptides were performed using mascot version 2.2 (Matrix Science, London, U.K.) as described previously^54^.

### Western blot analysis

Cells were washed once with ice-cold PBS, lysed in cold lysis buffer (150 mM NaCl, 50 mM Tris-HCl pH 7.3, 0.25 mM EDTA pH 8.0, 1% sodium deoxycholate, 1% Triton X-100, 0.2% sodium fluoride and 0.1% sodium orthovanadate supplemented with protease inhibitor cocktail (Roche Applied Biosciences). Cell lysate was centrifuged at 13,000 r.p.m. for 30 min, boiled in 2 × sample buffer, separated on either 7.5, 10 or 4–20% gradient SDS–PAGE and blotted onto nitrocellulose membrane. The membrane was blocked in 5% skimmed milk in PBS containing 0.1% Tween-20, probed with primary antibody followed by appropriate secondary antibody conjugated with horseradish peroxidase (Pierce) and immunoreactive bands were visualized by enhanced chemiluminescence super signal pico (Pierce). Bands were quantified using ImageJ software. For phospho-proteins, bands were normalized to their respective total protein levels, while for other panels bands were normalized to a loading control (β-actin). The respective values in arbitrary units are either shown in the panels as ‘relative levels’ or plotted graphically as fold change. The uncropped scanned full gels are shown in Supplementary Fig. 17.

### Immunoprecipitation

Cells were lysed in lysis buffer (20 mM Tris-HCl, pH 7.5, 150 mM NaCl, 1% Triton X-100 and 1 mM PMSF with complete EDTA-free protease inhibitor mixture (Roche)). The lysate was incubated on ice for 30 min and cleared by centrifugation at 13,000 r.p.m. for 30 min at 4 °C. IP was performed at 4 °C with 5 μg of antibody in the presence of either protein A or protein G-Sepharose 4 Fast Flow (GE Healthcare) for 4 h at 4 °C in a rocker. The Sepharose bound proteins was then washed three times with cell lysis buffer and three times with cold PBS. Bound proteins were eluted with 2 × Laemmli sample buffer, resolved by SDS–PAGE for subsequent Western blotting. Quantification of IP protein band was normalized to that of bait protein and represented as percentage pulldown (% IP).

### Reverse transcription PCR analysis

Total RNA was isolated from indicated cell lines using Qiagen RNeasy minikit as per the manufacturer’s protocol. An amount of 10 μg RNA aliquots were then reverse transcribed using High Capacity cDNA reverse transcription kit, Applied Biosystems Inc., as per standard protocols. cDNA was used as template for PCR, and PCR products were separated on a 1.5% agarose gel. Agrin Z exon flanking primers: forward 5′-ACACCGTCCTCAACCTGAAG-3′ and reverse 5′-CCAGGTTGTAGCTCAGTTGC-3′. Expected product size=453 bp. Vimentin primers: forward 5′-TCCAGCAGCTTCCTGTAGGT-3′ reverse 5′-CCCTCACCTGTGAAGTGGAT-3′, Arp2 forward 5′-GTAATGTTTGAAACTTACCAG-3′, reverse 5′-CGATACCAAGGAATACCGAC-3′; Arp3 forward 5′-AGAGATTTGAAAAGAACTGTAG-3′ reverse 5-′ACTGGTCCAACACTCTTGTC-3′, GAPDH: forward 5′-GAGCCACATCGCTCAGAC-3′ reverse 5′-CTTCTCATGGTTCACACCC-3′, respectively.

### Agrin ELISA

Secreted Agrin were determined in normal and HCC patient plasma using an Agrin ELISA kit (MyBiosource, Inc.). Briefly, 50 μl of provided Agrin standard or diluted plasma were added to 96-well plates coated with Agrin antibodies. A volume of 100 μl of secondary antibody conjugated to horseradish peroxide were then added to the wells and incubated for 1 h at 37 °C. Post washing, 100 μl of substrate was added to each well followed by 50 μl stop solution. Absorbance was measured at 450 nm wavelength. Agrin levels were calculated from a standard curve. Each patient samples were analysed in quadruplex. Data represented as mean±s.d.

### Cytosolic soluble and membrane fractionation

Cells were harvested in homogenization buffer (20 mM Tris-HCl, pH 7.5, 150 mM NaCl and 1 mM PMSF with complete EDTA-free protease inhibitor mixture (Roche)). The cell suspension homogenized through a 29-G needle 10 times on ice was briefly centrifuged at 2,000 r.p.m. for 5 min at 4 °C. The clarified lysate was then centrifuged at 80,000 r.p.m. using the TLA120.1 rotor (Beckman Coulter) for 1 h at 4 °C. Cytosol was collected and the membrane pellet was washed once with cytosol buffer and then centrifuged at 80,000 r.p.m. for 30 min at 4 °C once more. The membrane pellet was dissolved in lysis buffer and centrifuged at 80,000 r.p.m. for 1 h at 4 °C for the third time. The solubilized membrane proteins were then collected.

### Lipid raft/caveolae membrane isolation

Lipid raft/caveolae extraction was performed as per the instructions of lipid raft/caveolae isolation kit (Sigma) and previously published protocols^55^. Briefly, confluent cell lines were lysed in 0.5 M sodium bicarbonate lysis buffer (pH 11.0) containing protease inhibitor cocktail (Roche) for 15 min on rocker. Cell lysate were homogenized in a Dounce homogenizer (10 strokes) and sonicated for 10 s at 4 °C. A discontinuous gradient (0–35%) of optiprep and lysis buffer was made, and 2 ml of cell lysate and optiprep (35%) was placed at the bottom of a centrifuge tube with each layer pre-ceding on top. The tubes were centrifuged at 40,000 r.p.m. for 4 h using a Beckmann SWI-55 rotor. A volume of 1 ml fractions were collected from the top and analysed by Dot and western blots.

### Agrin internalization assay

Hep3B cells were seeded in eight-well chamber slides (Nalge Nunc, Inc.). Confluent cells (70–80%) were treated with 3 μg ml^−1^ Agrin antibody (Santa Cruz Biotechnology, Inc.) and 0.5 μg ml^−1^ CTxB subunit conjugated with Alexa 488 (green) in chilled complete medium for 1 h at 4 °C on ice. Subsequently, the antibody was washed away and cells were replenished with complete media for indicated time points at 37 °C. Cells were fixed after indicated time points with 4% paraformaldehyde for 15 min at room temperature (RT) and processed for immunofluorescence analysis using appropriate secondary Alexa Fluor-labelled antibodies (Molecular Probes).

### Immunofluorescence confocal microscopy

Cells grown either on coverslips (Thermo Fisher Scientific) or in eight-well chamber slides (Nalge Nunc, Inc.) were washed two times with PBS supplemented with 1 mM CaCl_2_ and 1 mM MgCl_2_ (PBSCM). They were fixed with 4% paraformaldehyde in PBSCM for 15 min at RT, washed five times at 5-min intervals using PBSCM and permeabilized with 0.1% Triton X-100 in PBSCM for 5 min at RT. The cells were then immunostained with appropriate primary antibodies diluted in fluorescence dilution buffer (PBSCM with 5% FBS and 2% bovine serum albumin) for 1 h at RT and washed five times with 0.1% Triton X-100 PBSCM at 5-min intervals. Secondary antibodies conjugated to either Alexa 488 (green) or Alexa 595 (red) were diluted appropriately in fluorescence dilution buffer and incubated at RT for 1 h. The coverslips were washed again with 0.1% Triton X-100 PBSCM five times for 5-min each and were mounted on microscopic slides with Vectashield mounting medium containing 4,6-diamidino-2-phenylindole (DAPI; Vector Laboratories). Confocal microscopy was performed with either an Axioplan II microscope (Carl Zeiss, Inc.) equipped with Zeiss confocal scanning optics or Olympus Fluoview 1000 confocal microscope (Olympus).

### Immunohistochemistry

Paraffin-embedded tissue sections were deparaffinized in Bond Dewax solution, rehydrated through 100% ethanol to 1 × Bond Wash solution, antigenic epitope retrieval performed at pH 6.0 and blocked in 10% goat serum for 30 min. Slides were then incubated with primary antibodies in Bond diluent solution for 45 min, washed three times, polymerized for 10 min and washed again for at least four times. Nuclei were counterstained with haematoxylin for 5 min, rinsed in deionized water and mounted in mounting medium. Slides were visualized under a bright-field microscope using Leica imaging software.

### Live-cell imaging

Control or Agrin shRNA-tranduced MHCC-LM3 cells were cultured in glass bottom Petri dishes (MatTek Corporation) for 12–16 h before imaging live at 37 °C using the differential interference contrast filter in a confocal microscope (Olympus). For paxillin-GFP imaging, cells were trypsinized and plated as in adhesion assay and imaged 3 h post plating. Each image shown in panels corresponds to a 1-min interval. Data were analysed using the Fluoview software.

### Wound-healing assay

Cell migration was assessed by wound-healing assays. Briefly, confluent MHCC-LM3 cells either transduced with control shRNA or Agrin shRNA plated on six-well plates were wounded by manual scratching with a 200-μl pipette tip. Subsequently, cells were washed with PBS and incubated at 37 °C in complete media. At the indicated time points, phase contrast images at specific wound sites were taken.

### Anchorage-independent growth in soft agar

A volume of 1.5 ml of 0.5% agar (electrograde ultra-pure; Invitrogen, Carlsbad, CA) supplemented with DMEM, 10% FBS, was plated in six-well culture dishes as bottom agar. Three thousand cells were mixed with 1.5 ml of 0.35% agar supplemented with DMEM, 10% FBS, and plated on top of the bottom agar. A volume of 1 ml of media was added on top of solidified agar layers and colonies were allowed to grow in incubator at 37 °C for 10–12 days. Images of cell colonies were observed in an inverted microscope.

### Three-dimensional sphere culture

A volume of 45 μl of growth factor reduced Matrigel (BD Biosciences) was added to eight-well chamber slides and solidified in 37 °C incubator for 15 min. MHCC-LM3 cells were trypsinized and resuspended in 400 μl culture media containing 2% Matrigel to have 5,000 cells per well. The cells were grown in a 5% CO_2_ humidified incubator for 10–12 days and nourished with medium every 3–4 days. Microscopic observation was performed to screen spheres.

### Transwell migration and invasion assays

Cell invasion was determined using the 24-well chambers with 8-μm pore polycarbonate membranes either uncoated (for migration) or pre-coated with Matrigel Basement Membrane Matrix (for invasion; BD Biosciences). The chambers were rehydrated in serum-free medium. Complete medium with 10% FBS (700 μl) served as chemoattractant in the bottom chamber and 1 × 10^4^ cells per ml cells were incubated for 24 h at 37 °C, 5% CO_2_. At the end of incubation period, cells invading membrane or Matrigel were washed and stained with 0.05% crystal violet solution and imaged in a bright-field microscope.

### Fibronectin adhesion assay

Briefly, culture plates were coated with 10 μg ml^−1^ fibronectin for 1 h at 37 °C. Control or Agrin knockdown cells (2 × 10^3^ cells ml^−1^) were trypsinized and plated on coated dishes. Adhered cells were analysed either by live-cell imaging for morphological changes at indicated time points or lysates were collected for western blot analysis.

### Actin polymerization assay

Actin polymerization assay was performed according to actin polymerization biochem kit (catalogue no. BK003), Cytoskeleton, Inc. based on enhanced pyrene actin fluorescence principle. Briefly, 200 μl of 0.4 mg ml^−1^ globular actin (G-actin) in actin buffer with ATP was added to Corning 96-well plate reader, shaken for 5 s and baseline fluorescence was read at 435 nm for 3 min for every min interval. A volume of 20 μl of cell lysate (20 μg protein) and 20 μl of actin polymerization buffer (10 × stock) were added and plate was read constantly for 1 h with an interval of 1 min. Means of triplicate data was expressed as arbitrary fluorescence units.

### Gelatin degradation invadopodia assay

Invadopodia formation was assayed as per manufacturer’s protocol using invadopodia assay kit (catalogue. no. ECM671), Millipore. Briefly, coverslides were coated with 0.2% w/v poly-L-lysine for 20 min at RT, washed three times with sterile PBS, followed by 15 min fixation with 0.5% glutaraldehyde at RT. After washing, slides were layered with Cy3-labelled gelatin and unlabelled gelatin in a ratio of 1:5 for 30 min at RT. Labelled gelatin matrix was sterilized with 70% ethanol for 30 min. MHCC-LM3 (2 × 10^3^) cells were layered onto labelled gelatin matrix and cultured for 12–16 h under standard tissue culture conditions. Cells were then fixed with 3.7% paraformaldehyde (PFA) for 15 min at RT and processed for immunofluorescence. Cells with degraded matrix were imaged in a confocal microscope and quantified using ImageJ software.

### Microarray data mining and gene expression analysis

We analysed microarray data sets containing normal and HCC patient samples (Oncomine)^42^,^43^. Since all of them were assayed on Affymetrics human-UG133 platform, the mean expression of Agrin using AGRN 212285_s_at as query was determined. Microarray data are pre-processed by Oncomine in a standardized way by log_2_ transformation and scaling the median value per microarray to 0.

### Statistical analysis

All experiments were performed in triplicates and repeated three times. For *in vitro* experiments, bar charts and graph represent mean values, and error bar indicates s.d. For *in vivo* experiments, error bars represent s.e.m. A paired ‘two-tailed’ Student’s *t*-test was performed using GraphPad Prism software, and data were considered significant when *P value <0.05, **P value <0.005 and ***P value<0.0005, respectively.

## Author contributions

S.C. and W.H. conceived and designed the project; S.C., H.L.F.S. and J.G. performed quantitative proteomics analysis with inputs from J.G.; M.L., S.C. and S.C.A. performed *in vivo* experiments with inputs from W.H. and V.T.; S.C. along with J.C., X.Z., Y.S.O. and L.S.L. performed other experiments; K.M.H. provided HCC patient tissues and plasma samples; and S.C. analysed data and wrote the paper with inputs from W.H.

## Additional information

**How to cite this article:** Chakraborty, S. *et al.* An oncogenic role of Agrin in regulating focal adhesion integrity in hepatocellular carcinoma. *Nat. Commun.* 6:6184 doi: 10.1038/ncomms7184 (2015).

## Supplementary Material

Supplementary Figures and Tables Supplementary Figures 1-17 and Supplementary Tables 1-3

Supplementary Movie 1 Invadopodia and membrane protrusion imaging in control shRNA transduced MHCC-LM3 cells

Supplementary Movie 2 Invadopodia and membrane protrusion imaging in Agrin shRNA transduced MHCC-LM3 cells

Supplementary Movie 3 Focal adhesion imaging in control MHCC-LM3 cells expressing paxillinGFP plated on fibronectin. Cells were imaged live 3h post plating for 7 min with an interval of 1 min. Black arrows indicate focal adhesions

Supplementary Movie 4 Focal adhesion imaging in Agrin depleted MHCC-LM3 cells plated same as for movie 3

Supplementary Movie 5 Live cell imaging of Agrin depleted cells expressing paxillin-GFP treated with 10μg/ml soluble Agrin for 1 day. Black arrows indicate focal adhesions

## References

[b1] Parkin D. M., Bray F., Ferlay J. & Pisani P. Global cancer statistics, 2002. CA Cancer J. Clin. 55, 74–108 (2005). 15761078 10.3322/canjclin.55.2.74

[b2] Whittaker S., Marais R. & Zhu A. X. The role of signaling pathways in the development and treatment of hepatocellular carcinoma. Oncogene 29, 4989–5005 (2010). 20639898 10.1038/onc.2010.236

[b3] Huynh H. *et al.* Targeting receptor tyrosine kinase pathways in hepatocellular carcinoma. Anticancer Agents Med. Chem. 11, 560–575 (2011). 21554207 10.2174/187152011796011055

[b4] Kischel P. *et al.* Cell membrane proteomic analysis identifies proteins differentially expressed in osteotropic human breast cancer cells. Neoplasia 10, 1014–1020 (2008). 18714363 10.1593/neo.08570 PMC2517647

[b5] Boersema P. J., Geiger T., Wisniewski J. R. & Mann M. Quantification of the N-glycosylated secretome by super-SILAC during breast cancer progression and in human blood samples. Mol. Cell. Proteomics. 12, 158–171 (2013). 23090970 10.1074/mcp.M112.023614 PMC3536897

[b6] Harsha H. C., Molina H. & Pandey A. Quantitative proteomics using stable isotope labeling with amino acids in cell culture. Nat. Protoc. 3, 505–516 (2008). 18323819 10.1038/nprot.2008.2

[b7] Tatrai P. *et al.* Agrin, a novel basement membrane component in human and rat liver, accumulates in cirrhosis and hepatocellular carcinoma. Lab. Invest. 86, 1149–1160 (2006). 16983329 10.1038/labinvest.3700475

[b8] Somoracz A. *et al.* Agrin immunohistochemistry facilitates the determination of primary versus metastatic origin of liver carcinomas. Hum. Pathol. 41, 1310–1319 (2010). 20471664 10.1016/j.humpath.2009.10.029

[b9] Tatrai P. *et al.* Agrin and CD34 immunohistochemistry for the discrimination of benign versus malignant hepatocellular lesions. Am. J. Surg. Pathol. 33, 874–885 (2009). 19194276 10.1097/PAS.0b013e318194b3ea

[b10] Burgess R. W., Skarnes W. C. & Sanes J. R. Agrin isoforms with distinct amino termini: differential expression, localization, and function. J. Cell Biol. 151, 41–52 (2000). 11018052 10.1083/jcb.151.1.41 PMC2189804

[b11] Neumann F. R. *et al.* An alternative amino-terminus expressed in the central nervous system converts agrin to a type II transmembrane protein. Mol. Cell. Neurosci. 17, 208–225 (2001). 11161480 10.1006/mcne.2000.0932

[b12] McMahan U. J. The agrin hypothesis. Cold Spring Harb. Symp. Quant. Biol. 55, 407–418 (1990). 1966767 10.1101/sqb.1990.055.01.041

[b13] Ferns M., Deiner M. & Hall Z. Agrin-induced acetylcholine receptor clustering in mammalian muscle requires tyrosine phosphorylation. J. Cell Biol. 132, 937–944 (1996). 8603924 10.1083/jcb.132.5.937 PMC2120739

[b14] Denzer A. J., Hauser D. M., Gesemann M. & Ruegg M. A. Synaptic differentiation: the role of agrin in the formation and maintenance of the neuromuscular junction. Cell Tissue Res. 290, 357–365 (1997). 9321698 10.1007/s004410050941

[b15] Kim N. *et al.* Lrp4 is a receptor for Agrin and forms a complex with MuSK. Cell 135, 334–342 (2008). 18848351 10.1016/j.cell.2008.10.002 PMC2933840

[b16] Zhang B. *et al.* LRP4 serves as a coreceptor of agrin. Neuron 60, 285–297 (2008). 18957220 10.1016/j.neuron.2008.10.006 PMC2743173

[b17] Lin L. *et al.* Induction of filopodia-like protrusions by transmembrane agrin: role of agrin glycosaminoglycan chains and Rho-family GTPases. Exp. Cell Res. 316, 2260–2277 (2010). 20471381 10.1016/j.yexcr.2010.05.006 PMC2902627

[b18] Uhm C. S., Neuhuber B., Lowe B., Crocker V. & Daniels M. P. Synapse-forming axons and recombinant agrin induce microprocess formation on myotubes. J. Neurosci. 21, 9678–9689 (2001). 11739577 10.1523/JNEUROSCI.21-24-09678.2001 PMC6763053

[b19] Denzer A. J., Brandenberger R., Gesemann M., Chiquet M. & Ruegg M. A. Agrin binds to the nerve-muscle basal lamina via laminin. J. Cell Biol. 137, 671–683 (1997). 9151673 10.1083/jcb.137.3.671 PMC2139873

[b20] Quail D. F. & Joyce J. A. Microenvironmental regulation of tumor progression and metastasis. Nat. Med. 19, 1423–1437 (2013). 24202395 10.1038/nm.3394 PMC3954707

[b21] Frisch S. M., Vuori K., Ruoslahti E. & Chan-Hui P. Y. Control of adhesion-dependent cell survival by focal adhesion kinase. J. Cell Biol. 134, 793–799 (1996). 8707856 10.1083/jcb.134.3.793 PMC2120934

[b22] Cheng N., Li Y. & Han Z. G. Argonaute2 promotes tumor metastasis by way of up-regulating focal adhesion kinase expression in hepatocellular carcinoma. Hepatology 57, 1906–1918 (2013). 23258480 10.1002/hep.26202

[b23] Shintani Y., Hollingsworth M. A., Wheelock M. J. & Johnson K. R. Collagen I promotes metastasis in pancreatic cancer by activating c-Jun NH(2)-terminal kinase 1 and up-regulating N-cadherin expression. Cancer Res. 66, 11745–11753 (2006). 17178870 10.1158/0008-5472.CAN-06-2322

[b24] Bagi C. M. *et al.* Sunitinib and PF-562,271 (FAK/Pyk2 inhibitor) effectively block growth and recovery of human hepatocellular carcinoma in a rat xenograft model. Cancer Biol. Ther. 8, 856–865 (2009). 19458500 10.4161/cbt.8.9.8246

[b25] Stokes J. B. *et al.* Inhibition of focal adhesion kinase by PF-562,271 inhibits the growth and metastasis of pancreatic cancer concomitant with altering the tumor microenvironment. Mol. Cancer Ther. 10, 2135–2145 (2011). 21903606 10.1158/1535-7163.MCT-11-0261 PMC3213273

[b26] Tang H. *et al.* Loss of Scar/WAVE complex promotes N-WASP- and FAK-dependent invasion. Curr. Biol. 23, 107–117 (2013). 23273897 10.1016/j.cub.2012.11.059

[b27] Tancioni I. *et al.* FAK Inhibition disrupts a beta5 integrin signaling axis controlling anchorage-independent ovarian carcinoma growth. Mol. Cancer Ther. 13, 2050–2061 (2014). 24899686 10.1158/1535-7163.MCT-13-1063 PMC4126870

[b28] Bezakova G. & Ruegg M. A. New insights into the roles of agrin. Nat. Rev. Mol. Cell. Biol. 4, 295–308 (2003). 12671652 10.1038/nrm1074

[b29] Eckert M. A. *et al.* Twist1-induced invadopodia formation promotes tumor metastasis. Cancer Cell 19, 372–386 (2011). 21397860 10.1016/j.ccr.2011.01.036 PMC3072410

[b30] Tarone G., Cirillo D., Giancotti F. G., Comoglio P. M. & Marchisio P. C. Rous sarcoma virus-transformed fibroblasts adhere primarily at discrete protrusions of the ventral membrane called podosomes. Exp. Cell Res. 159, 141–157 (1985). 2411576 10.1016/s0014-4827(85)80044-6

[b31] Linder S. The matrix corroded: podosomes and invadopodia in extracellular matrix degradation. Trends Cell. Biol. 17, 107–117 (2007). 17275303 10.1016/j.tcb.2007.01.002

[b32] Buccione R., Orth J. D. & McNiven M. A. Foot and mouth: podosomes, invadopodia and circular dorsal ruffles. Nat. Rev. Mol. Cell. Biol. 5, 647–657 (2004). 15366708 10.1038/nrm1436

[b33] Murphy D. A. & Courtneidge S. A. The ‘ins’ and ‘outs’ of podosomes and invadopodia: characteristics, formation and function. Nat. Rev. Mol. Cell. Biol. 12, 413–426 (2011). 21697900 10.1038/nrm3141 PMC3423958

[b34] Bhattacharya R. *et al.* Recruitment of vimentin to the cell surface by beta3 integrin and plectin mediates adhesion strength. J. Cell. Sci. 122, 1390–1400 (2009). 19366731 10.1242/jcs.043042 PMC2721003

[b35] Kuo J. C., Han X., Hsiao C. T., Yates J. R. 3rd & Waterman C. M. Analysis of the myosin-II-responsive focal adhesion proteome reveals a role for beta-Pix in negative regulation of focal adhesion maturation. Nat. Cell. Biol. 13, 383–393 (2011). 21423176 10.1038/ncb2216 PMC3279191

[b36] Wu C. *et al.* Arp2/3 is critical for lamellipodia and response to extracellular matrix cues but is dispensable for chemotaxis. Cell 148, 973–987 (2012). 22385962 10.1016/j.cell.2011.12.034 PMC3707508

[b37] Alexander N. R. *et al.* Extracellular matrix rigidity promotes invadopodia activity. Curr. Biol. 18, 1295–1299 (2008). 18718759 10.1016/j.cub.2008.07.090 PMC2555969

[b38] Pignatelli J., Tumbarello D. A., Schmidt R. P. & Turner C. E. Hic-5 promotes invadopodia formation and invasion during TGF-beta-induced epithelial-mesenchymal transition. J. Cell Biol. 197, 421–437 (2012). 22529104 10.1083/jcb.201108143 PMC3341156

[b39] Zhang W., Coldefy A. S., Hubbard S. R. & Burden S. J. Agrin binds to the N-terminal region of Lrp4 protein and stimulates association between Lrp4 and the first immunoglobulin-like domain in muscle-specific kinase (MuSK). J. Biol. Chem. 286, 40624–40630 (2011). 21969364 10.1074/jbc.M111.279307 PMC3220470

[b40] Burden S. J. SnapShot: neuromuscular junction. Cell 144, 826–826 e1 (2011). 21376240 10.1016/j.cell.2011.02.037

[b41] Burden S. J., Yumoto N. & Zhang W. The role of MuSK in synapse formation and neuromuscular disease. Cold Spring Harb. Perspect. Biol. 5, a009167 (2013). 23637281 10.1101/cshperspect.a009167 PMC3632064

[b42] Roessler S. *et al.* A unique metastasis gene signature enables prediction of tumor relapse in early-stage hepatocellular carcinoma patients. Cancer Res. 70, 10202–10212 (2010). 21159642 10.1158/0008-5472.CAN-10-2607 PMC3064515

[b43] Wurmbach E. *et al.* Genome-wide molecular profiles of HCV-induced dysplasia and hepatocellular carcinoma. Hepatology 45, 938–947 (2007). 17393520 10.1002/hep.21622

[b44] Eustace B. K. *et al.* Functional proteomic screens reveal an essential extracellular role for hsp90 alpha in cancer cell invasiveness. Nat. Cell. Biol. 6, 507–514 (2004). 15146192 10.1038/ncb1131

[b45] Casaletto J. B. & McClatchey A. I. Spatial regulation of receptor tyrosine kinases in development and cancer. Nat. Rev. Cancer. 12, 387–400 (2012). 22622641 10.1038/nrc3277 PMC3767127

[b46] Kalluri R. & Weinberg R. A. The basics of epithelial-mesenchymal transition. J. Clin. Invest. 119, 1420–1428 (2009). 19487818 10.1172/JCI39104 PMC2689101

[b47] Shibue T. & Weinberg R. A. Integrin beta1-focal adhesion kinase signaling directs the proliferation of metastatic cancer cells disseminated in the lungs. Proc. Natl Acad. Sci. USA 106, 10290–10295 (2009). 19502425 10.1073/pnas.0904227106 PMC2700942

[b48] Feng M. *et al.* Therapeutically targeting glypican-3 via a conformation-specific single-domain antibody in hepatocellular carcinoma. Proc. Natl Acad. Sci. USA 110, E1083–E1091 (2013). 23471984 10.1073/pnas.1217868110 PMC3607002

[b49] Xu M. Z. *et al.* AXL receptor kinase is a mediator of YAP-dependent oncogenic functions in hepatocellular carcinoma. Oncogene 30, 1229–1240 (2011). 21076472 10.1038/onc.2010.504 PMC3330262

[b50] Hui K. M. Human hepatocellular carcinoma: expression profiles-based molecular interpretations and clinical applications. Cancer Lett. 286, 96–102 (2009). 19095350 10.1016/j.canlet.2008.11.005

[b51] Choi H. Y. *et al.* APP interacts with LRP4 and agrin to coordinate the development of the neuromuscular junction in mice. Elife 2, e00220 (2013). 23986861 10.7554/eLife.00220 PMC3748711

[b52] Chen L. *et al.* Recoding RNA editing of AZIN1 predisposes to hepatocellular carcinoma. Nat. Med. 19, 209–216 (2013). 23291631 10.1038/nm.3043 PMC3783260

[b53] Neuhuber B. & Daniels M. P. Targeting of recombinant agrin to axonal growth cones. Mol. Cell. Neurosci. 24, 1180–1196 (2003). 14697677 10.1016/j.mcn.2003.08.008

[b54] Swa H. L., Blackstock W. P., Lim L. H. & Gunaratne J. Quantitative proteomics profiling of murine mammary gland cells unravels impact of annexin-1 on DNA damage response, cell adhesion, and migration. Mol. Cell. Proteomics 11, 381–393 (2012). 22511458 10.1074/mcp.M111.011205 PMC3412969

[b55] Chakraborty S., ValiyaVeettil M., Sadagopan S., Paudel N. & Chandran B. c-Cbl-mediated selective virus-receptor translocations into lipid rafts regulate productive Kaposi’s sarcoma-associated herpesvirus infection in endothelial cells. J. Virol. 85, 12410–12430 (2011). 21937638 10.1128/JVI.05953-11 PMC3209366

