# Supplementary material for: An oncogenic role of Agrin in regulating focal adhesion integrity in hepatocellular carcinoma
Source: Nat Commun. 2015 Jan 29;6:6184. doi: 10.1038/ncomms7184 (PMC4317502; doi:10.1038/ncomms7184)
Supplement: Supplementary Figures and Tables — Supplementary Figures 1-17 and Supplementary Tables 1-3 [file ncomms7184-s1.pdf]

Supplementary Information

Supplementary Figure 1

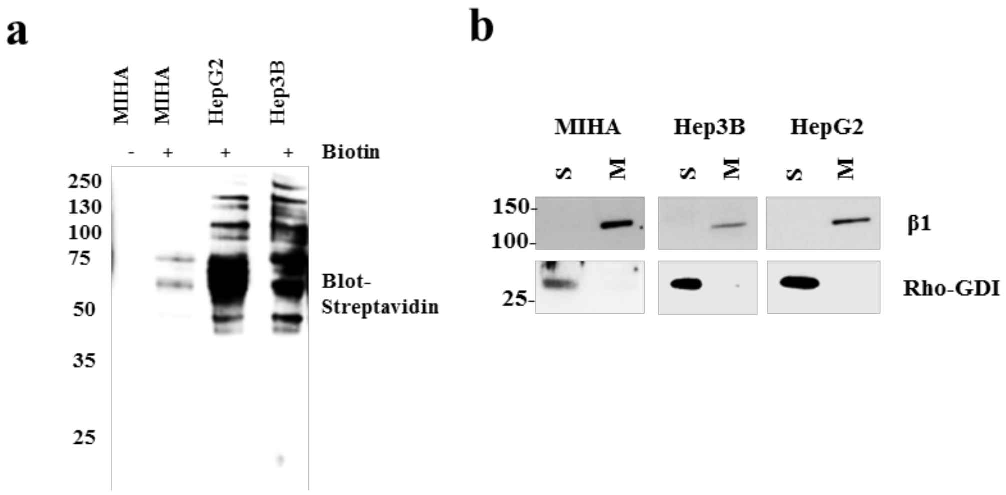

Supplementary Figure 1: Characterization of cell surface biotinylation in non-tumorigenic and tumorigenic HCC cell lines. (a) Indicated cell lines were either mock or surface biotinylated, cell membrane fractions were enriched and affinity-purified using streptavidin-A beads and analysed by a Western blot using a streptavidin antibody. **(b)** Characterization of purity of cell membrane (M) and cytosolic soluble (S) fractions. Integrin β1 and Rho-GDI are markers for membrane and soluble fractions, respectively. Molecular weight markers are indicated on the side.

Supplementary Figure 2

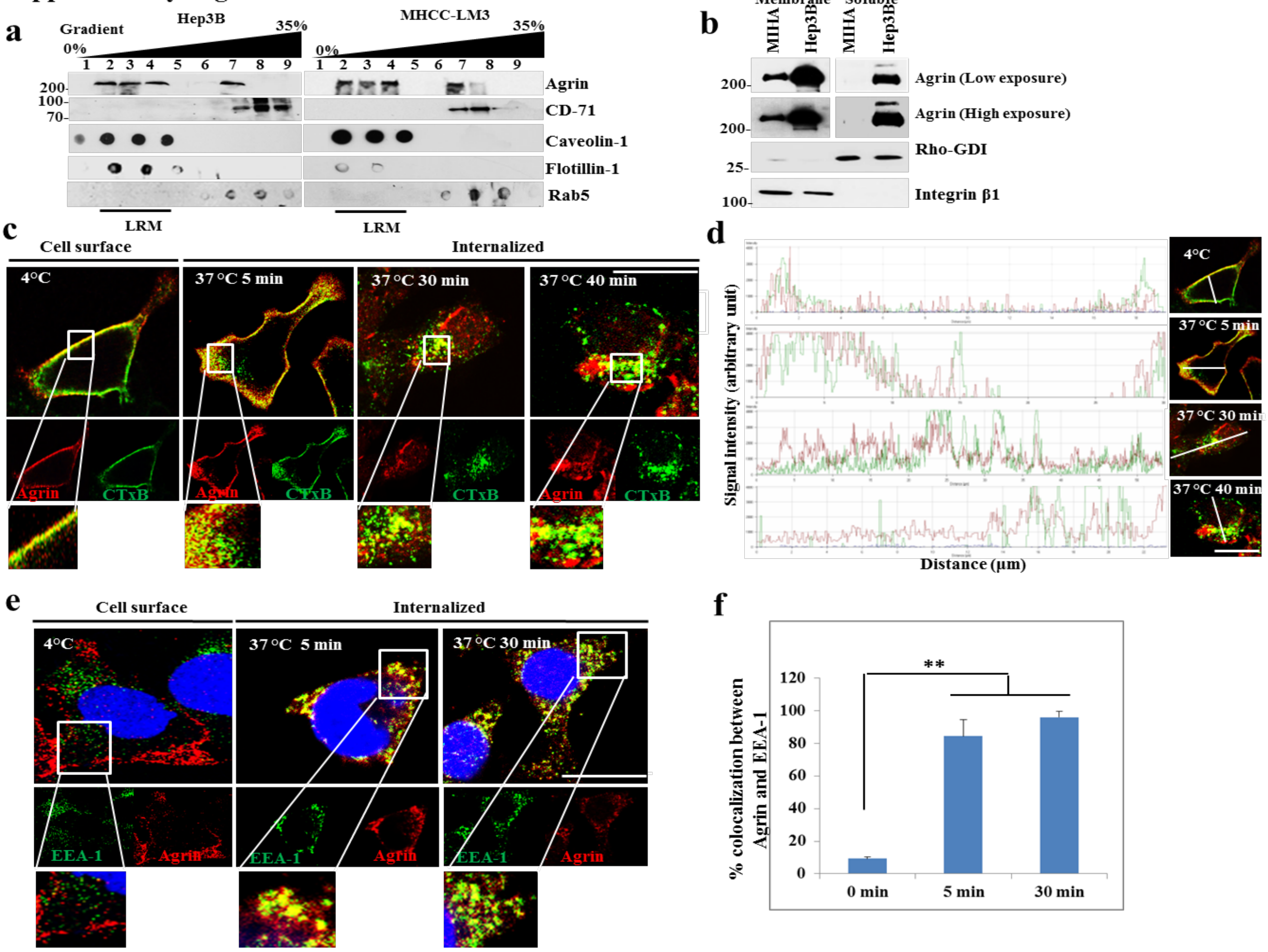

Supplementary Figure 2: Localization and constitutive internalization of Agrin in HCC cell lines. (a) Biochemical density gradient fractions from the indicated cell lines were analysed by Western blot for Agrin. Caveolin-1 and Flotillin-1 are markers for lipid raft membrane (LRM) and caveolae rich domains, CD-71 marks high density non-lipid raft domains of plasma membranes and Rab-5 marks the early endosomal vesicles containing fractions. **(b)** Membrane and soluble fractions from MIHA and Hep3B cells were isolated as described in methods section and Western blotted for Agrin. Rho-GDI and Integrin β1 served as markers for soluble and membrane fractions, respectively. **(c)** Hep3B cells were treated with Agrin antibody and recombinant Alexa 488 labeled cholera toxin B (CTxB) for 1h at 4°C, allowed to internalize at 37°C for the indicated time-points, fixed and processed for immunofluorescence microscopy analysis. Representative confocal images of internalization assay are shown. Boxed areas represent enlarged panels. Scale bar: 10μm. **(d)** Line scan signal intensity plots for the indicated cells at 4°C (cell-surface) and 37°C (internalized) are shown. Fluorescence signal intensity is represented in arbitrary units. Scale bar: 10μm. **(e)** Hep3B cells were incubated with Agrin antibody as in (c) and allowed to internalize for indicated time-points. 4% PFA fixed cells were immunostained with rabbit anti-EEA-1 antibody for 1h at room temperature (RT), followed by anti-mouse Alexa 555 and anti-rabbit Alexa 488 secondary antibodies. Representative confocal images are shown. Boxed areas are represented as enlarged panels. Scale bar: 10μm. **(f)** Quantification of internalized Agrin colocalized with EEA-1 at indicated time-points measured by the number of colocalizing cells per field. Error bars represent the s.d. of means of at least five different fields containing at least 10 cells per field (***p value*=0.002, students ‘t’ test).

Supplementary Figure 3

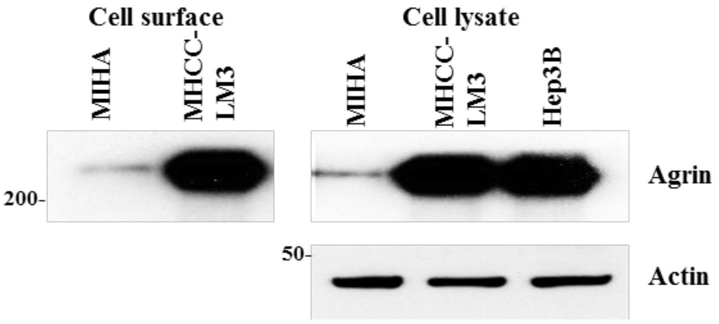

Supplementary Figure 3: Cell surface expression of Agrin in HCC cell lines. Surface biotinylated or total cell lysate from indicated cell lines were subjected to a Western blot using Agrin antibody. Actin served as loading control for total cell lysates.

Supplementary Figure 4

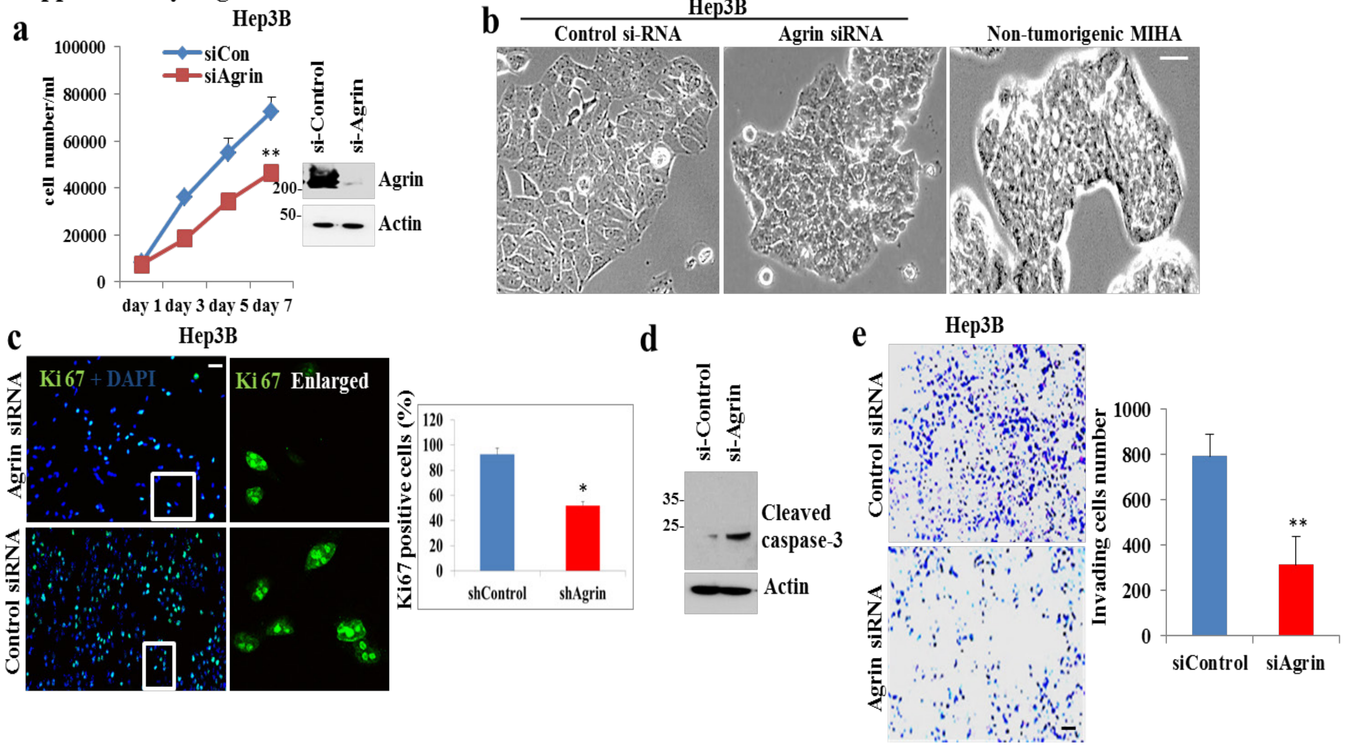

Supplementary Figure 4: Anti-tumorigenic effects of siRNA mediated Agrin knockdown in Hep3B cells. (a) Hep3B cells transfected with either control or Agrin siRNA were cultured for the indicated number of days. At each time-point, cells were trypsinized and total cell count was measured as an index of proliferation. Error bar represents s.d. of means for three independent experiments performed in triplicates (***p value*=0.002*,* students 't'test). 72 h post-transfection, total cell lysates were collected and analysed for Agrin knockdown. β-actin served as a loading control. **(b)** Morphology of control and Agrin siRNA transfected Hep3B cells 72h post-transfection. Representative bright field microscope images are shown. Non-tumorigenic MIHA cells are used as control. Scale bar: 10 µm. **(c)** Control or si-Agrin transfected Hep3B cells were immunostained with mouse Ki67 antibody followed by anti-mouse secondary antibody conjugated with Alexa fluor 488. Representative confocal images merged with DAPI are shown. Boxed region represents the enlarged panel with nuclear Ki67 staining. Scale bar: 10µm. Quantitative plot depicting the percentage Ki67 positive cells in at-least five different microscopic fields bearing at least 15-20 cells each. Error bars represent the s.d. of the means of at least five different fields (**p value*=0.03, students 't'test). **(d)** Western blot analysis for cleaved caspase-3 in control and Agrin siRNA transfected Hep3B cells. β-actin served as a loading control. **(e)** Hep3B cells were transfected with control or Agrin siRNA. 48h post-transfection, cells were subjected to a matrigel invasion assay. Invasive cells were fixed in 4% PFA after 24h, stained with 0.1% crystal violet solution and visualized in a bright-field microscope under 10X magnification. Images were quantified using ImageJ software and represented graphically. Error bar represents s.d. of the means of three biological replicates performed in triplicates (***p value*=0.002, students 't' test). Scale bar: 50µm.

Supplementary Figure 5

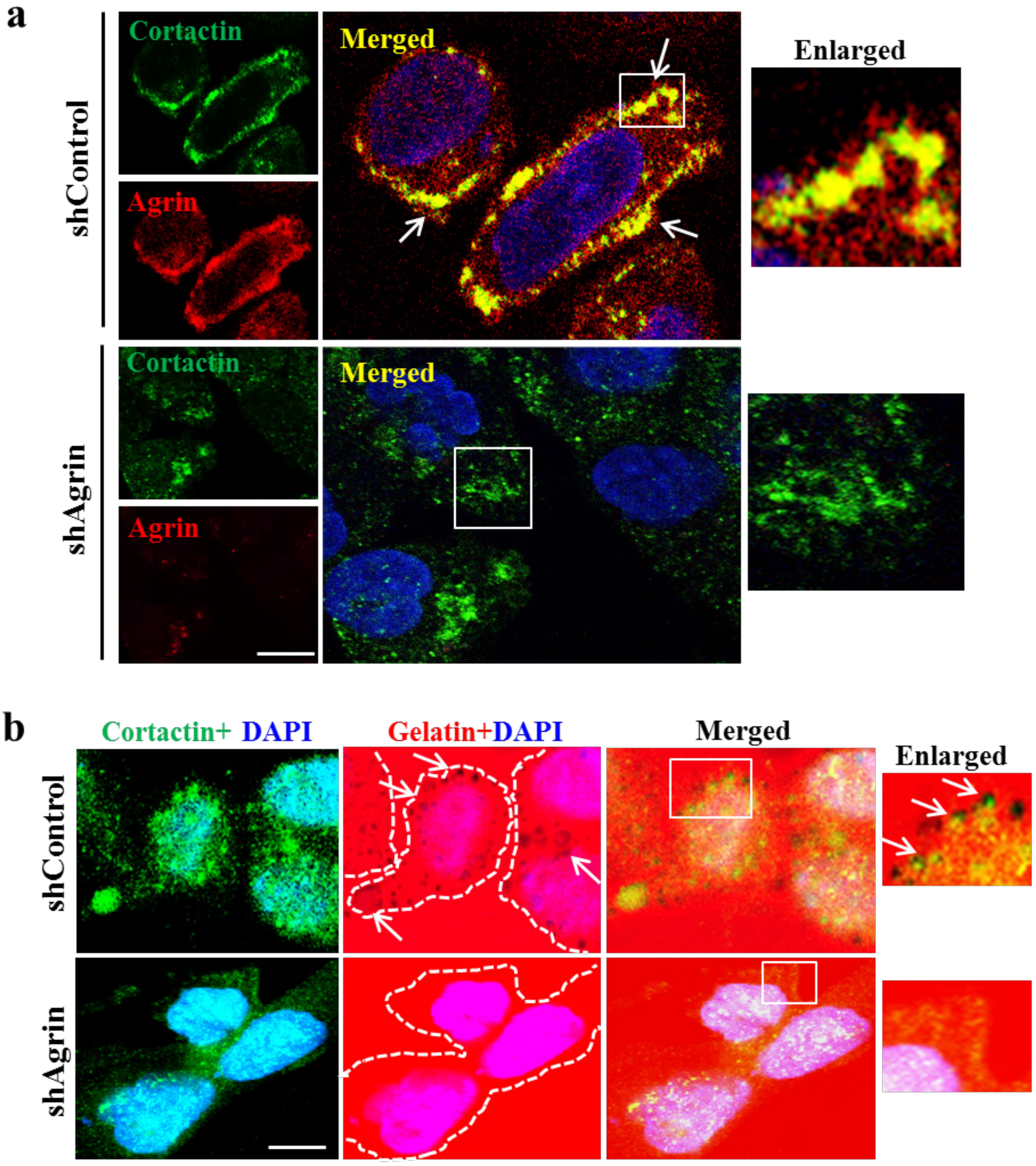

Supplementary Figure 5: Agrin clusters with invadopodia marker cortactin at invasive matrix degradation sites. (a) Control and Agrin depleted MHCC-LM3 cells were fixed, immunostained with mouse Agrin and rabbit cortactin antibodies followed by anti-mouse Alexa 555 and anti-rabbit Alexa 488 secondary antibodies. Representative confocal images are shown. Arrows point to Agrin-cortactin clusters at the cell periphery. Boxed region is represented as enlarged panels. Scale bar: 10µm. **(b)** Control and Agrin depleted MHCC-LM3 cells were cultured on Cy3-Gelatin for 12h, fixed and then stained with rabbit cortactin antibody followed by anti-rabbit Alexa 488 conjugated secondary antibodies. Representative confocal images are shown. Nuclei are stained with DAPI. Boxed areas are represented as enlarged panels. Arrows indicate cortactin enriched matrix degradation sites. Scale bar: 10µm.

Supplementary Figure 6

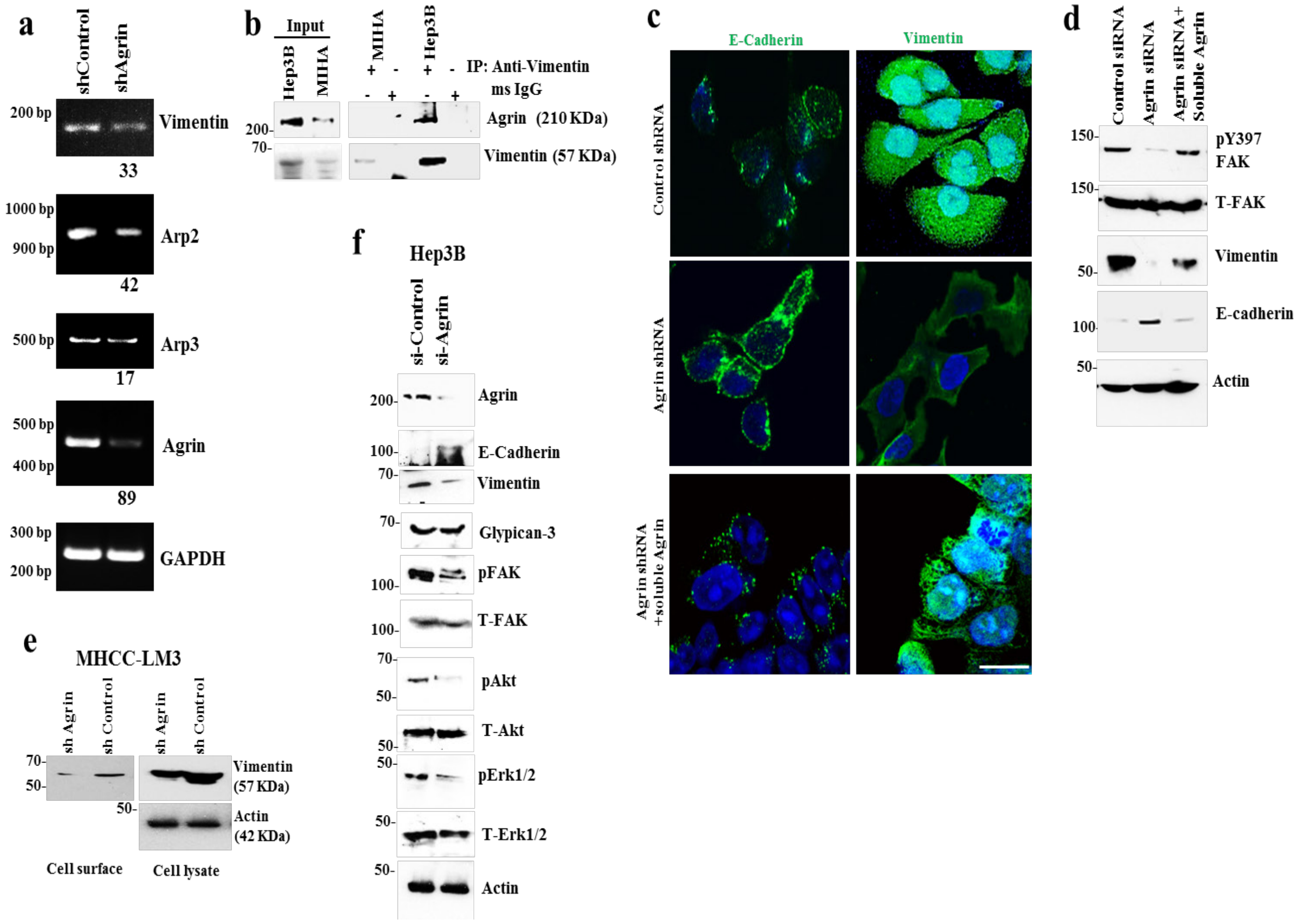

Supplementary Figure 6: Agrin regulates mesenchymal marker signaling. (a) RT-PCR analysis of indicated genes in control and Agrin depleted MHCC-LM3 cells. GAPDH mRNA levels served as endogenous control. The numbers indicate the percentage (%) reduction in expression compared to control. Transcript sizes are indicated in base pairs (bp) on the side of the gel. **(b)** Three hundred microgram protein from indicated cell lines were immunoprecipitated with either mouse IgG or vimentin antibody and analyzed by Western blot probed with an Agrin antibody. The blot were stripped and re-probed with vimentin antibody. Thirty microgram (10%) total cell lysate was used as input. **(c)** Confocal microscopy analysis of control or Agrin shRNA transduced MHCC-LM3 cells with or without soluble Agrin (20µg/ml) treatment for 12h immunostained for E-cadherin and vimentin (green) by the respective mouse antibodies followed by anti-mouse Alexa 488 secondary antibodies. The cells were co-stained with DAPI (blue) and representative confocal images are shown. Scale bar: 10µm. **(d)** Control or Agrin siRNA transfected MHCC-LM3 cells were either supplemented with or without soluble Agrin (20µg/ml) for 12h. Total cell lysates were analysed by Western blot for the indicated proteins. β-actin served as loading control. **(e)** Control or Agrin shRNA transduced MHCC-LM3 cells were surface biotinylated and analyzed by a Western blot using vimentin antibody. Corresponding total cell lysate expression is also shown. β-actin served as loading control. **(f)** Hep3B cells were transfected with either control or Agrin specific siRNA. 72h post transfection, total cell lysates were subjected to Western blot analysis for the indicated signaling proteins. β-actin served as loading control.

Supplementary Figure 7

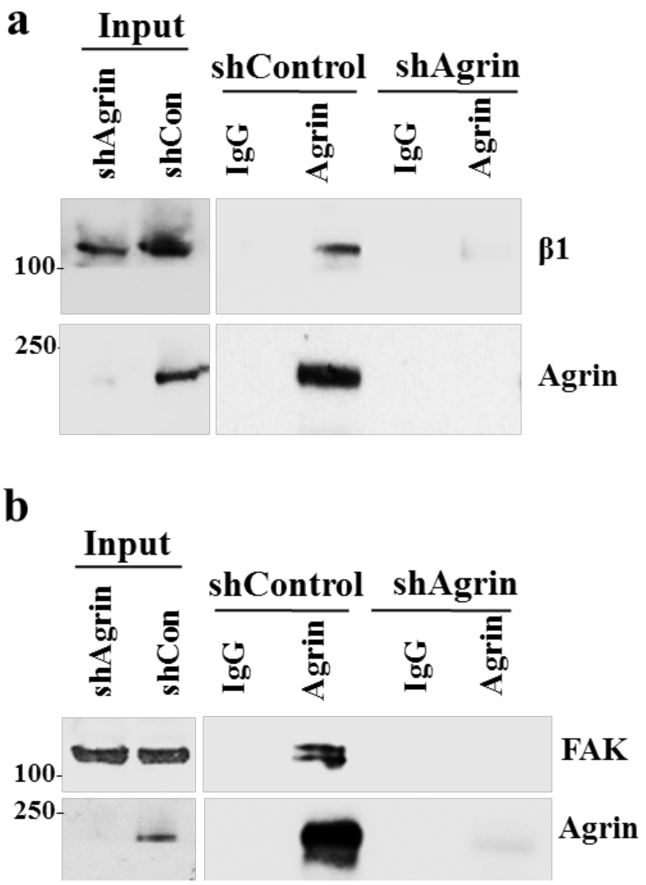

Supplementary Figure 7: Reverse co-immunoprecipitation interactions between Agrin and integrin-focal adhesion components. (a) shControl or shAgrin MHCC-LM3 cells were immunoprecipitated with IgG or Agrin antibodies and Western blotted for integrin β1. The blot was stripped and re-probed for Agrin. 30 µg (10%) total cell lysate were used as input control. **(b)** shControl or shAgrin MHCC-LM3 cells were immunoprecipitated with IgG or Agrin antibodies and Western blotted for FAK. The blot was stripped and re-probed for Agrin. 30 µg (10%) cell lysate were used as input control. Agrin depleted cell lysates were used as negative controls for IP reactions.

Supplementary Figure 8

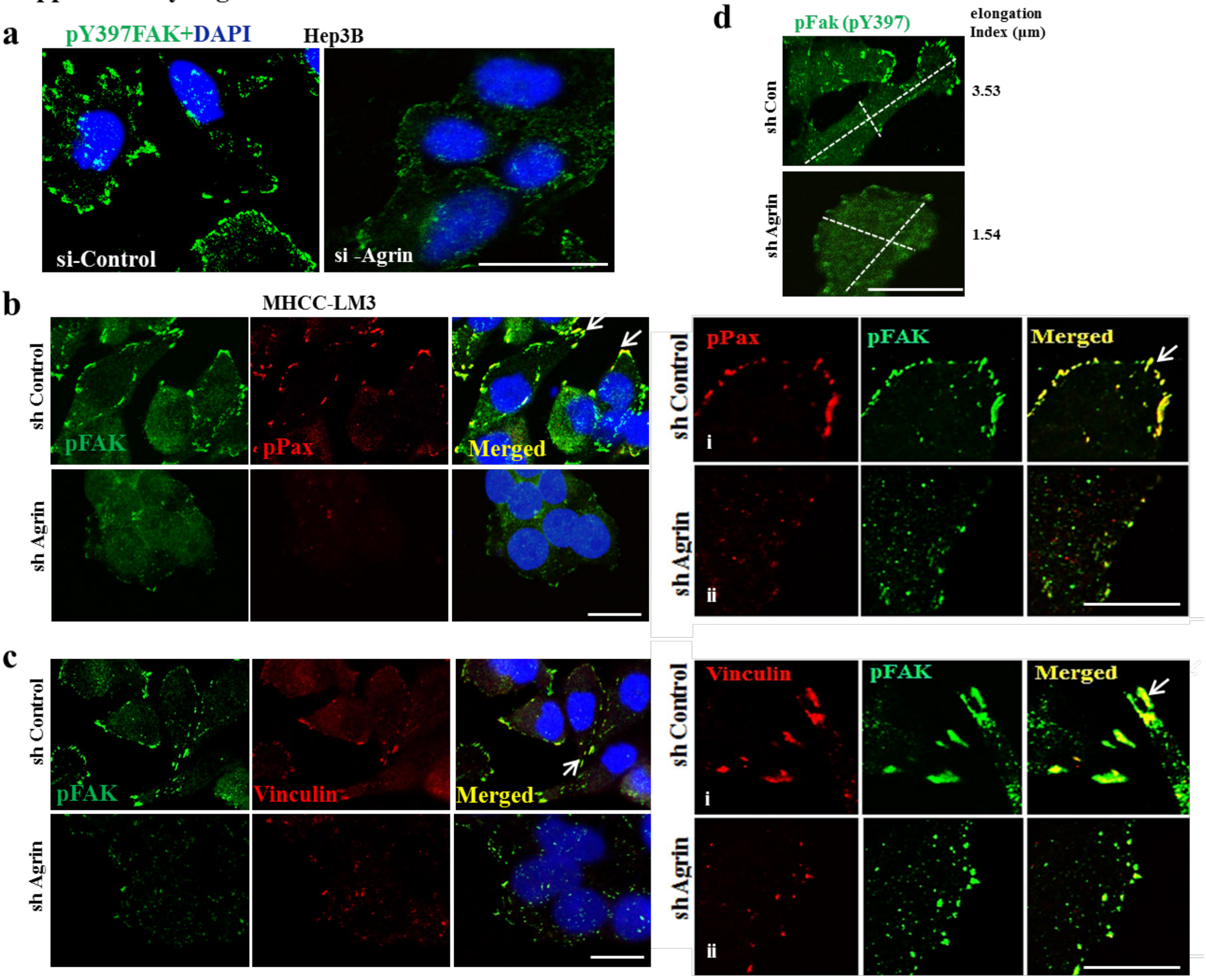

Supplementary Figure 8: Regulation of focal adhesion integrity by Agrin in HCC cell lines. (a) Confocal immunofluorescence analysis of focal adhesions in control and Agrin siRNA transfected Hep3B cells visualized by phospho-FAK (pY397) rabbit antibody followed by anti-rabbit secondary antibody conjugated with Alexa fluor 488. Scale bar: 10µm. **(b and c)** Control or Agrin shRNA MHCC-LM3 cells were immunostained with rabbit phospho-paxillin (Tyr118) (b) or rabbit vinculin (c) and mouse pFAK antibodies followed by anti-rabbit Alexa fluor 555 and mouse Alexa fluor 488 antibodies. Representative confocal images are shown. Higher magnification enlarged representative view of focal adhesions are depicted in left panels. Arrows indicate colocalization between paxillin, vinculin and pFAK at FAs. Scale bar: 10µm. **(d)** Serum starved control or Agrin knockdown MHCC-LM3 cells were trypsinized and plated on fibronectin coated plates for 2h. Post fixation, cells were stained with pFAK pY397. Cell elongation ratio calculated by signal intensity measurements for length and width of each cell in at least 5 different microscopic fields containing at least 10 cells each. Representative images are shown. Scale bar: 10µm.

Supplementary Figure 9

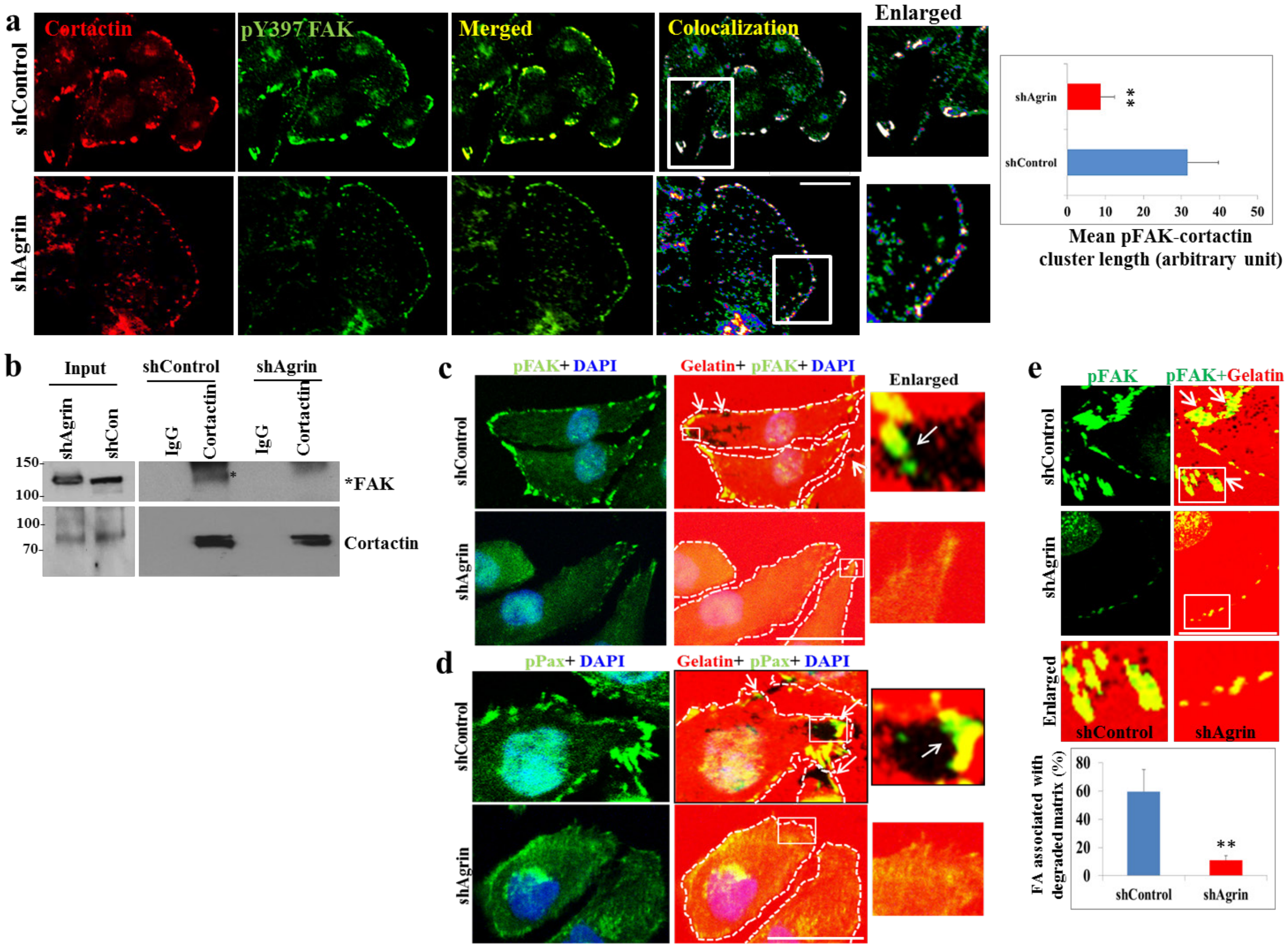

Supplementary Figure 9: Activated FAK is localized in the vicinity of invadopodia and degraded matrix. (a) Immunofluorescence analysis performed in control and Agrin knockdown MHCC-LM3 cells 12h post scratch assay using rabbit cortactin and mouse pY397FAK antibodies. Representative confocal images are shown. Colocalization ratio intensity profile is also shown and boxed areas are represented as enlarged panels. Scale bar: 10µm. Mean focal adhesion-cortactin cluster length was calculated using ImageJ software and plotted graphically and the length is represented in arbitrary units. Error bars represent the s.d. of means of atleast 15-20 cells per field (***p value*=0.0022, students 't' test). **(b)** shControl or shAgrin MHCC-LM3 cells were immunoprecipitated with rabbit IgG or cortactin antibodies and Western blotted for FAK. The blot was stripped and re-probed for cortactin. 30 µg (10%) total cell lysate were used as input control. * indicates the FAK specific band. **(c and d)** shControl and shAgrin MHCC-LM3 cells were cultured on Cy3-Gelatin for 12h before fixation and processed for immunofluorescence using pY397FAK (c) or pPaxillinTyr118 (d) antibodies. Nucleus is stained with DAPI. Arrows represent areas with degraded gelatin. Boxed areas are represented as enlarged panels. Arrows in enlarged panel indicate the presence of pFAK/pPax within degraded gelatin matrix. Scale bar: 10µm. **(e)** shControl and shAgrin MHCC-LM3 cells were cultured on Cy3-Gelatin and processed as in (c). Arrows represent activated focal adhesions (FA) at leading edges of cells in areas of matrix degradation. Scale bar: 10µm. Boxed region is represented as enlarged panels. At-least 5 different fields containing 15-20 cells each were analysed to estimate the number of cells where FA associated with degraded gelatin. Error bar represents s.d. (***p value*=0.0008, students 't' test).

Supplementary Figure 10

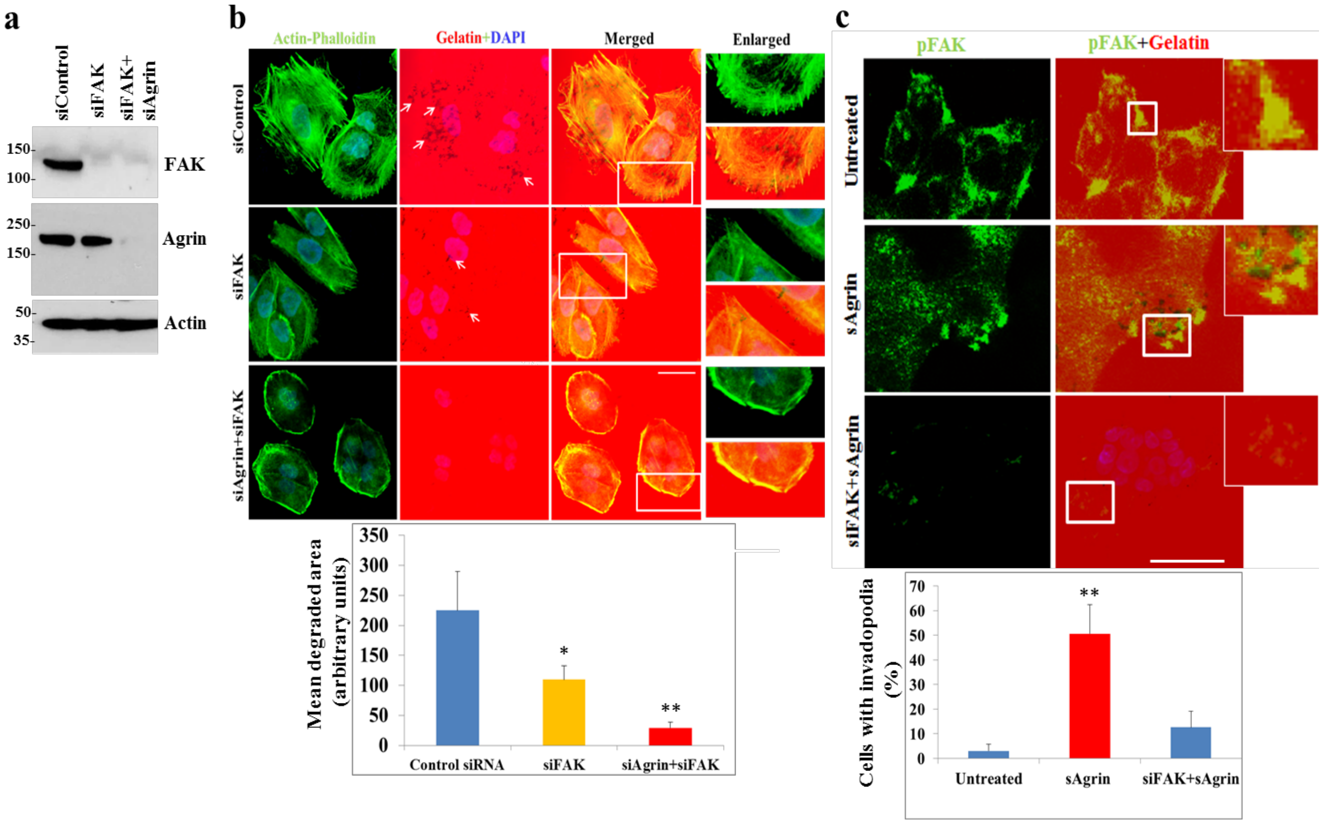

Supplementary Figure 10: Agrin promotes FAK dependant invadopodia and ECM degradation. (a) Western blot analysis for FAK and Agrin knockdown in MHCC-LM3 cells. β-actin served as loading control. **(b)** Control siRNA, FAK siRNA alone or both Agrin and FAK siRNA transfected MHCC-LM3 cells were plated on Cy3 gelatin for 18h before fixing and staining for phalloidin. Mean degraded areas were calculated by ImageJ software and represented in arbitrary units (**p value*=0.02,***p value*=0.001, students 't' test). Arrows indicate matrix degradation due to invadopodia. Boxed areas are represented as enlarged panels showing phalloidin and corresponding Cy3 gelatin merged images. Scale bar: 10µm. (c) Control or FAK siRNA transfected MIHA cells either untreated or treated with soluble Agrin (sAgrin) (10µg/ml) were grown in Cy3 gelatin for 1 day before fixation and staining with mouse pFAK antibody. Boxed region is represented as enlarged panels inset. At least 5 different fields containing 15-20 cells each were analysed to estimate the number of cells where FA associated with degraded gelatin. Scale bar: 10µm. Error bar represents s.d. (***p value*=0.002, students 't' test).

Supplementary Figure 11

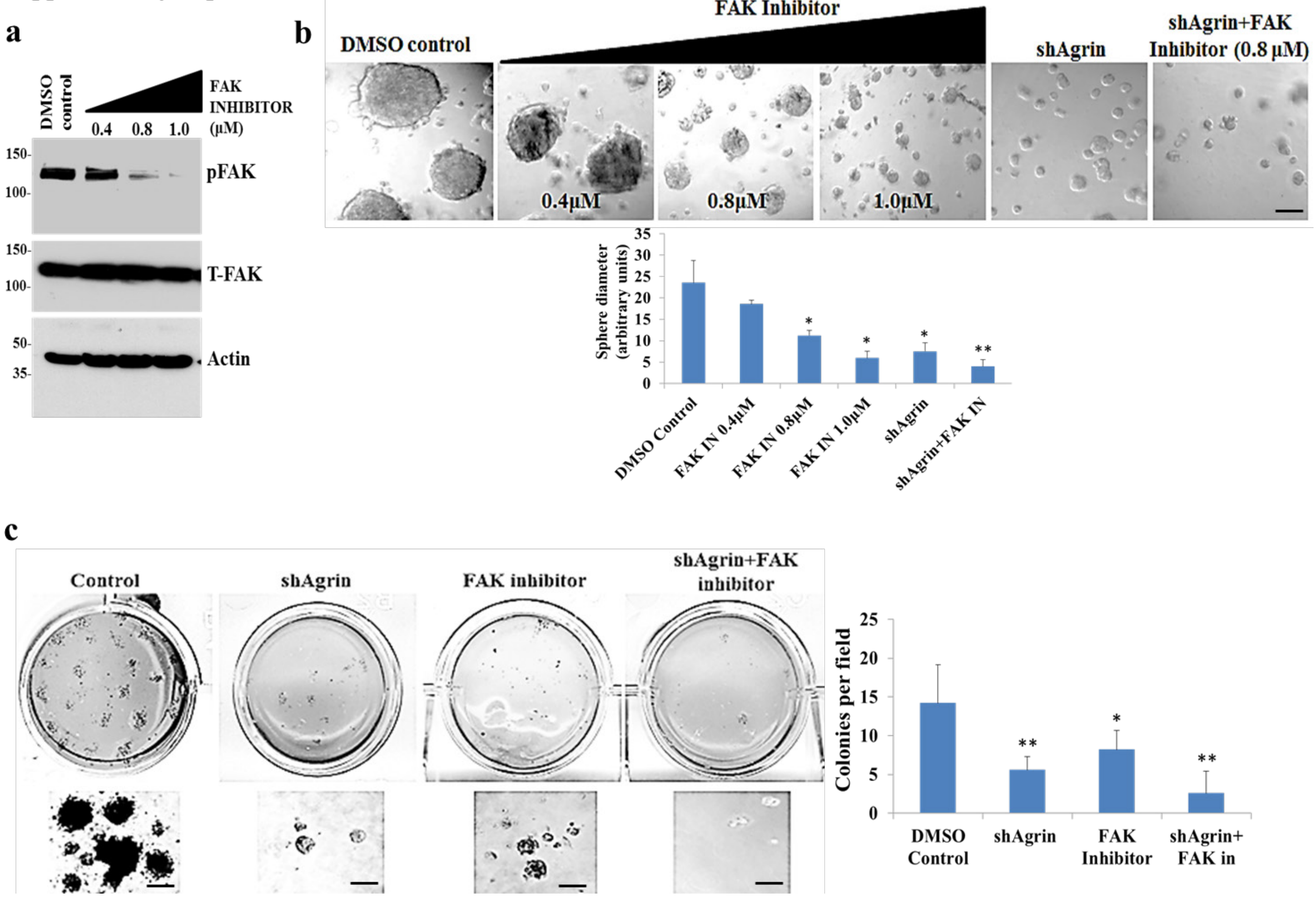

Supplementary Figure 11: Impact of focal adhesion and Agrin depletion on liver cancer cell growth. (a) Western blot analysis of pY397 FAK upon treatment of serum starved MHCC-LM3 cells with varying doses of PF-562271 for 1 day. β-actin served as loading control. **(b)** Control or Agrin depleted MHCC-LM3 cells were grown in matrigel coated chamber slides to form three dimensional spheres either in absence or presence of indicated concentrations of PF562271 (FAK inhibitor) for 10 days. Representative bright-field images of spheres are shown (Magnification 20X). At least 5-7 microscopic fields per condition were analysed and sphere diameter was calculated using ImageJ analysis and represented in arbitrary units. The experiment was performed in triplicates and error bars represent s.d. (**p value*=0.002, ***p value*=0.001, students 't' test). **(c)** Control and Agrin depleted MHCC-LM3 cells either untreated or treated with 0.8µM PF562271 were subjected to a soft agar assay. Representative images showing colonies at day 10 are shown. Inset represents bright-field images of colonies. Number of colonies in at least 10 different microscopic fields were quantitated by ImageJ software and represented graphically. Experiment was performed in triplicates and error bars represent s.d. (**p value*=0.03, ***p value*<0.002, students 't' test). Scale bar: 50 µm for panels **b** and **c**.

Supplementary Figure 12

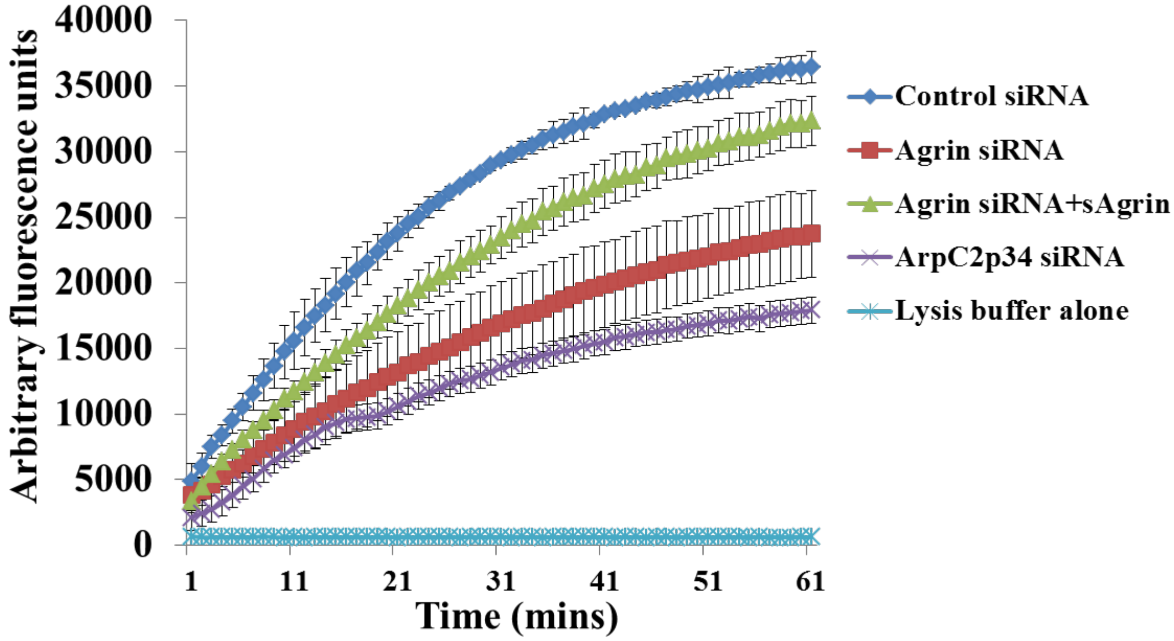

	Mean Fluorescence (Arbitrary units)	% activity
Control siRNA	26878	100
Agrin siRNA	15092	56
Agrin siRNA+sAgrin	20900	78
ArpC2 p34 siRNA	12289	45

Supplementary Figure 12: Effect of Agrin depletion on actin polymerization. 20 µg protein lysates from control, Agrin depleted MHCC-LM3 cells either untreated or treated with soluble Agrin (10µg/ml) for 1 day were added to depolymerized pyrene G-actin to induce polymerization. Fluorescence was measured at 435nm for 1 hr at an interval of 1 min. Knockdown cell lysates of ArpC2/p34 subunit of Arp2/3 complex served as a positive control while lysis buffer alone served as negative control. Mean fluorescence in arbitrary units is plotted and experiments were performed in triplicates. Error bars indicate s.d. The mean fluorescence in arbitrary units at $T_{1/2}$ (~30 min) and the relative activity expressed as percentage of control lysates are shown below (**p value*=0.03, ***p value*=0.005, ****p value*=0.0003, students 't' test).

Supplementary Figure 13

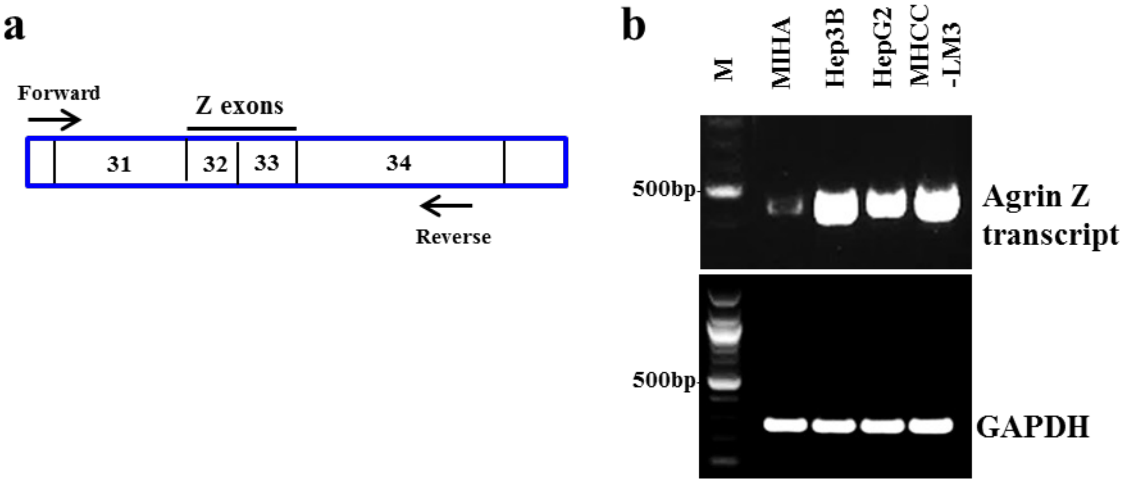

Supplementary Figure 13: Analysis of Agrin z transcript expression in HCC cell lines. (a) Schematic showing primers flanking Z exons 32-33 where alternative splicing in neuronal Agrin is prevalent. **(b)** RT-PCR analysis of expression of Z-transcript of Agrin in indicated cell lines. GAPDH mRNA is used as an endogenous control. M: marker lane showing transcript size in base pairs (bp).

Supplementary Figure 14

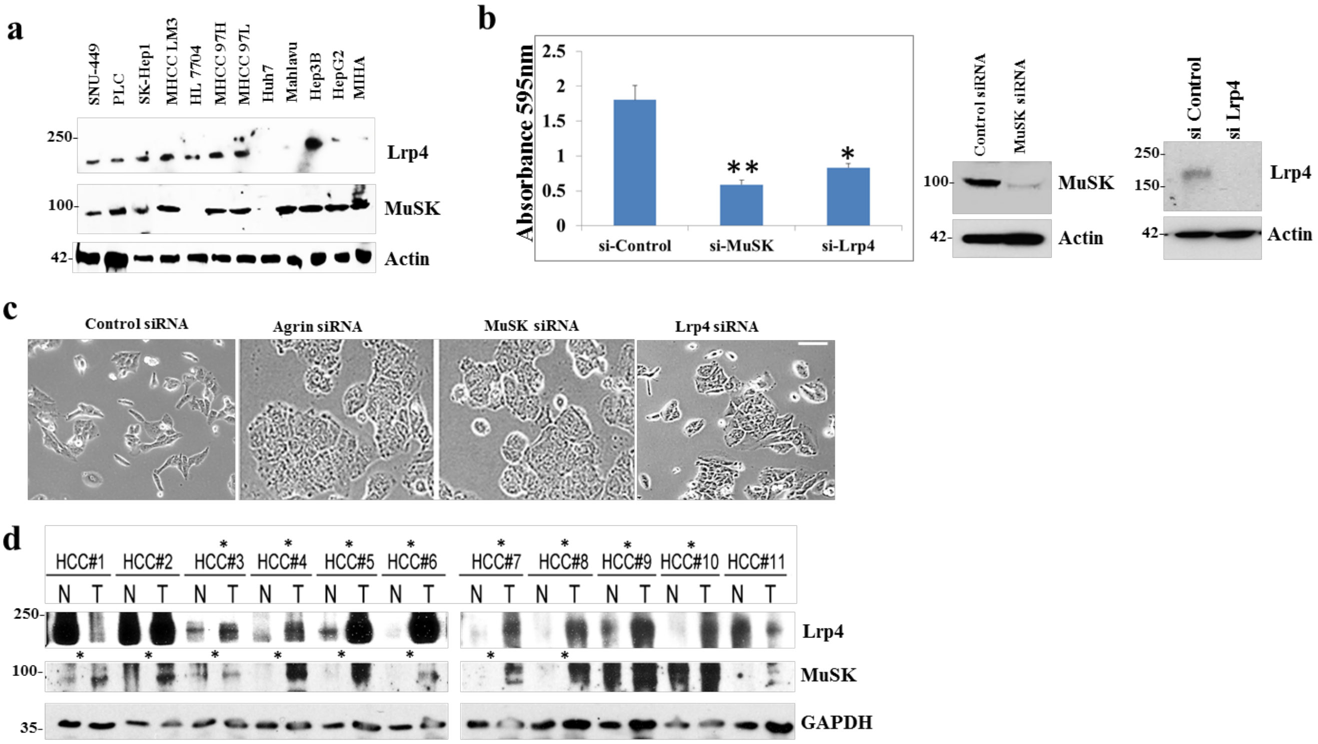

Supplementary Figure 14: Role of Lrp4-MuSK complex in controlling Agrin related events in HCC. (a) Lrp4 and MuSK expression in a panel of HCC cell lines. Actin represents loading control. **(b)** MHCC-LM3 cell proliferation upon Lrp4 and MuSK knockdown using a crystal violet assay three days post-siRNA treatment. Spectrophotometric absorbance was plotted. Statistical significance were calculated using a two-tailed student's 't' test and error bars represent the s.d. of three independent experiments performed in triplicates (***p value*=0.004 and **p value*=0.03, students 't' test). Western blot in the same transfected cells showing Lrp4 and MuSK knockdown. β-actin served as loading control. **(c)** Morphological analysis of MHCC-LM3 cells transfected with the indicated siRNAs 3-5 days post-siRNA treatment. Representative brightfield images are shown. Scale bar: 10µm. **(d)** Western blot analysis for Lrp4 and MuSK expression in a cohort of liver cancer patients in Singapore. GAPDH was used as loading control. *denotes tumor pairs with significant up-regulation of Lrp4 and/or MuSK in HCC tumors.

Supplementary Figure 15

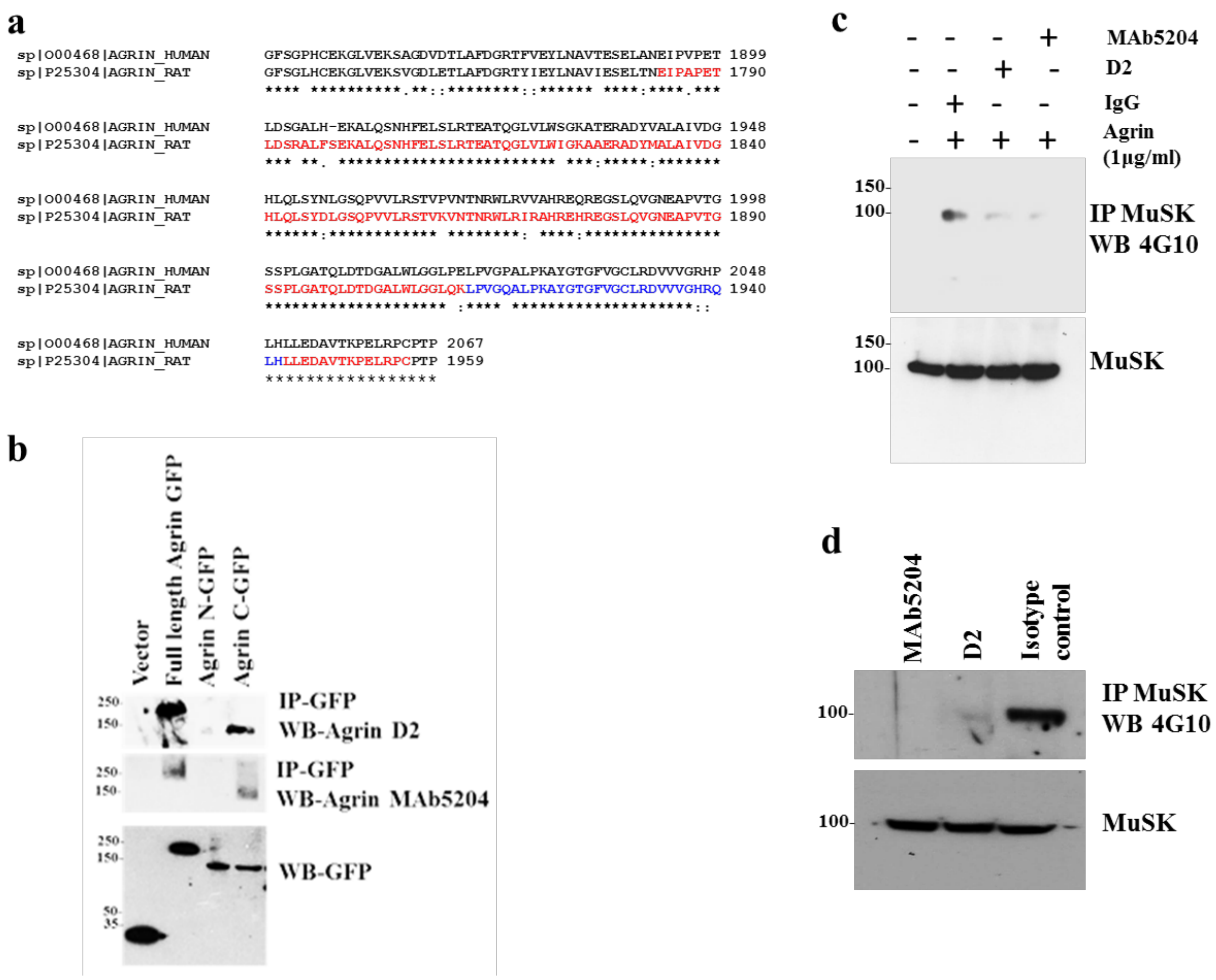

Supplementary Figure 15: Binding and function blocking properties of Agrin monoclonal antibodies. (a) Sequence alignment of human and rat Agrin C-terminal (C20) fragment illustrating the epitopes of monoclonal antibodies D2 (blue) and MAb5204 (red). Asterisks denote the conserved amino acids. **(b)** MHCC-LM3 cells were transfected with either vector control, full length Agrin-GFP, N-terminal Agrin GFP or C-terminal Agrin GFP constructs. 2 days post-transfection, total cell lysates were immunoprecipitated with a GFP monoclonal antibody and Western blotted using D2 or MAb5204 Agrin antibodies. GFP Western blot shows the expression of the constructs. **(c)** Differentiated mouse muscle C2C12 cells were pre-treated with the indicated antibodies (10µg/ml) for 12h, then either left untreated or treated with soluble Agrin (1µg/ml) for another 12h, immunoprecipitated with MuSK antibody and Western blotted using phospho-tyrosine specific 4G10 monoclonal antibody. The blot was stripped and re-probed for MuSK expression. **(d)** MHCC-LM3 cells treated with the indicated Agrin antibodies (10µg/ml) for 12h were immunoprecipitated with MuSK antibody and Western blotted using phospho-tyrosine specific 4G10 monoclonal antibody. The blot was stripped and re-probed for MuSK expression.

Supplementary Figure 16

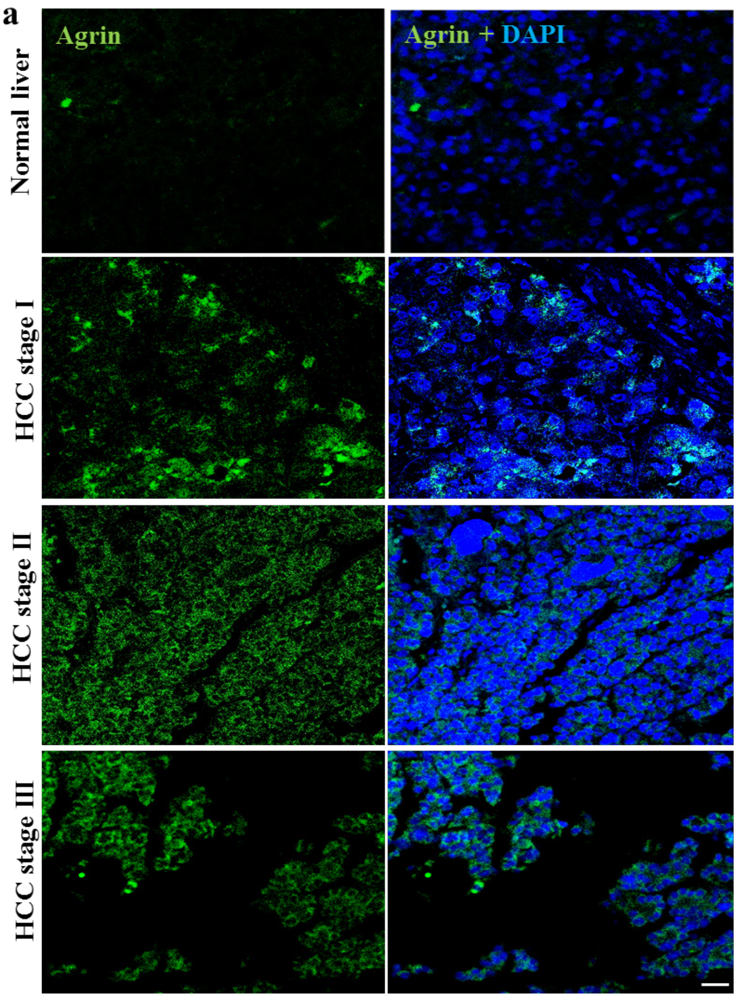

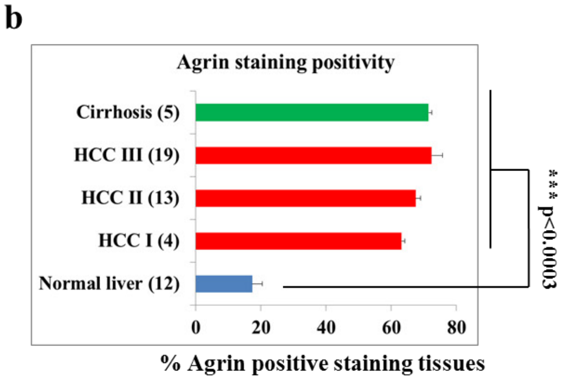

Supplementary Figure 16: Expression of Agrin in normal liver and HCC patient tissues. (a) HCC tissue microarray slides (US Biomax, cat #BC03116) containing normal liver and various HCC stage-wise sections were subjected to immunofluorescence analysis using anti-Agrin monoclonal antibody. DNA was counter-stained with DAPI. Scale bar: 10µm. **(b)** Quantitative plot depicting the proportion of Agrin staining tissue positivity in different HCC stages (****p value*<0.0003, students 't' test). The number of tumor tissues analysed for each HCC stage is indicated within parentheses. Liver cirrhosis patient tissues were used as positive control for Agrin expression.

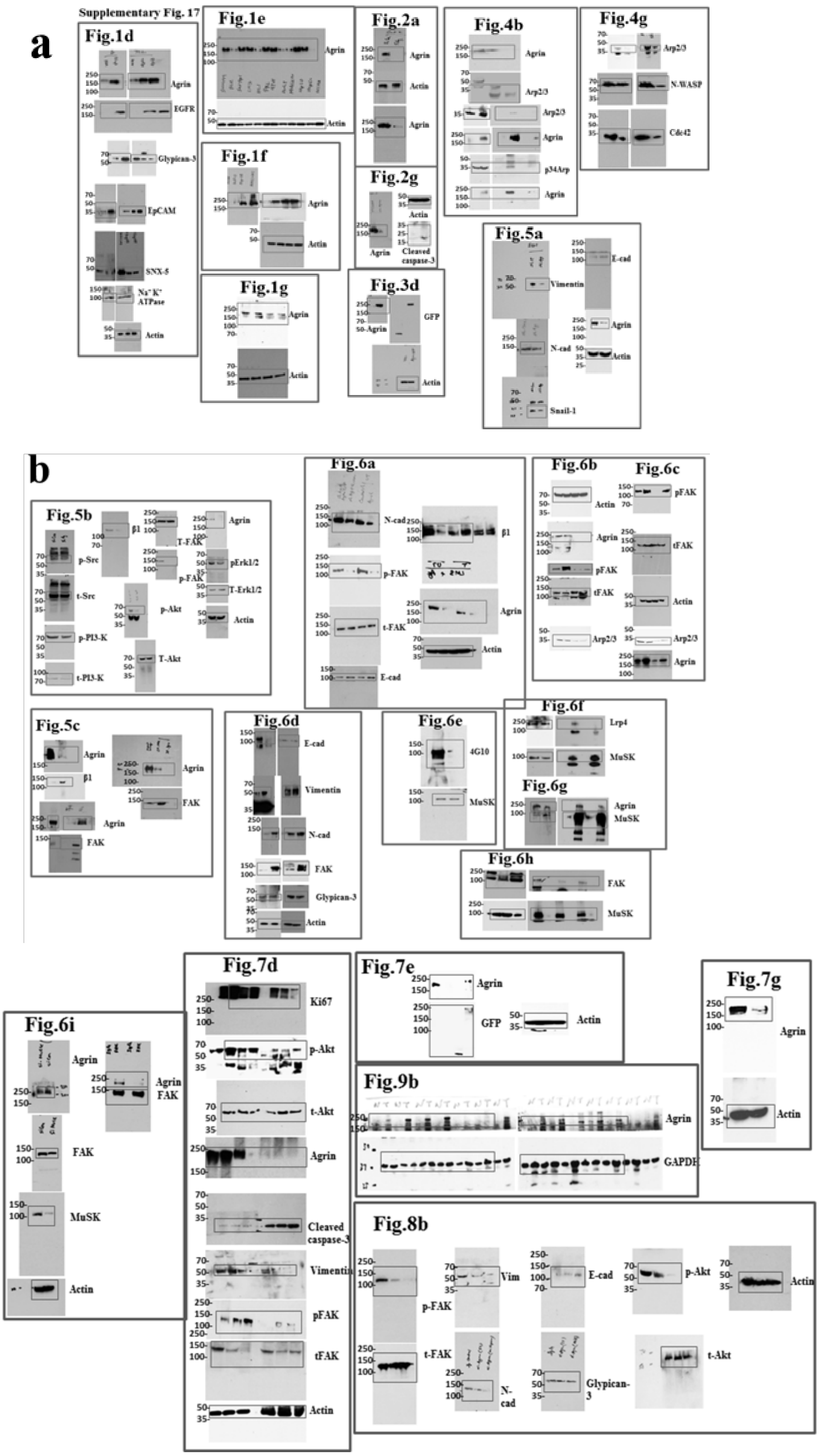

Supplementary Figure 17: Scanned images of key Western blots used in figures 1-9. (a) Scanned uncropped blots used in figures 1-5a. **(b)** Scanned uncropped blots used in figures 5b-9. Boxed region represents the area cropped and molecular weight (kDa) markers are indicated on the side.

Supplementary Table 1

Quantification software: MaxQuant v1.3.0.5

Search parameters: precursor tolerance - 7 ppm, MS/MS tolerance - 0.5 Da

Modifications: Acetyl (Protein N-term), Oxidation (M), Arginine-13C615N4 (R-full), Lysine-13C615N2 (K-full)

Database: HUMAN.fasta(Jan 2013)

Min. Ratio Count: 2

Protein IDs	Peptide counts (all	Protein names	Gene names	Sequence coverag	Mol. weight [kDa]	Sequence length	Ratio H/L L	Ratio H/L normalized L3	Ratio H/L count L3	log2(Normalized L3)	Comments
P02652	6	Apolipoprotein A-II;Truncated apolipoprotein A-II	APOA2	83	11.175	100	11.073	8.0299	7	3.005382021	Up
Q8N5G0;Q8N5G0-2;E5RH9	2;2;1	Uncharacterized protein C4orf52	C4orf52	13.1	18.402	168	15.538	7.7795	5	2.959677434	Up
Q9HCY8	7	Protein S100-A14	S100A14	80.8	11.662	104	15.79	7.1519	8	2.838326564	Up
Q9NX14-2;Q9NX14	4;4	NADH dehydrogenase [ubiquinone] 1 beta subcomplex s	NDUFB11	36.2	18.364	163	16.139	7.0436	4	2.816312982	Up
Q96T88-2;Q96T88	3;3	E3 ubiquitin-protein ligase UHRF1	UHRF1	3.2	91.115	806	9.9248	6.7086	2	2.746011726	Up
Q92804;Q92804-2;K7EPT6;I	4;4;4;1	TATA-binding protein-associated factor 2N	TAF15	7.8	61.829	592	12.365	6.341	2	2.664710377	Up
G3V556;G3V5Q3;P56378	2;2;2	6.8 kDa mitochondrial proteolipid	C14orf2;MP68	19.2	11.281	99	9.8083	6.1674	3	2.624662418	Up
Q96FQ6	7	Protein S100-A16	S100A16	75.7	11.801	103	11.838	5.9329	11	2.568737466	Up
Q96IX5	4	Up-regulated during skeletal muscle growth protein 5	USMG5	44.8	6.4575	58	12.847	5.9116	5	2.563548655	Up
P16401	6	Histone H1.5	HIST1H1B	22.1	22.58	226	15.895	5.7787	7	2.530744975	Up
Q8ND56;Q8ND56-2;B4DTG	3;3;3;2;1;1	Protein LSM14 homolog A	LSM14A	5.6	50.529	463	7.9175	5.7482	3	2.523110259	Up
Q9Y6A9;C9JBL1	2;2	Signal peptidase complex subunit 1	SPCS1	10.1	18.298	169	7.2905	5.7436	2	2.521955279	Up
Q9C002;H0YKW5	2;1	Normal mucosa of esophagus-specific gene 1 protein	NMES1;C15orf48	32.5	9.6172	83	8.9795	5.5795	2	2.480135843	Up
Q9BUP3-3;Q9BUP3;E9PI87;	8;6;3;2	Oxidoreductase HTATIP2	HTATIP2	29.3	30.13	276	10.423	5.4825	8	2.454833907	Up
Q6DD88;F5H6I7;C9JUU1;F5	12;11;9;4	Atlastin-3	ATL3	34.4	60.541	541	12.737	5.4726	14	2.452226412	Up
P56381;Q5VTU8	3;3	ATP synthase subunit epsilon, mitochondrial;ATP syntha	ATP5E;ATP5EP2	45.1	5.7797	51	9.0727	5.4595	2	2.44876883	Up
P08670;B0YJC4;B0YJC5;Q5J	40;38;20;12;5;4;4;4	Vimentin	VIM	80.7	53.651	466	12.779	5.3954	54	2.431729921	Up
Q9UBI6	3	Guanine nucleotide-binding protein G(I)/G(S)/G(O) subu	GNG12	56.9	8.0061	72	13.082	5.2571	4	2.394267178	Up
Q16799;Q16799-2;Q16799-	1;1;1;1	Reticulon-1	RTN1	1	83.617	776	16.876	5.0497	3	2.336197681	Up
P82979;H0YHG0;F8VZQ9;Q	6;5;5;5;1	SAP domain-containing ribonucleoprotein	SARNP;CIP29	31.9	23.671	210	14.714	4.8417	8	2.27551369	Up
Q6P161	4	39S ribosomal protein L54, mitochondrial	MRPL54	48.6	15.819	138	7.7206	4.7135	7	2.236798728	Up
Q14004;Q14004-2	4;4	Cyclin-dependent kinase 13	CDK13	4.2	164.92	1512	11.424	4.6948	2	2.2310637	Up
Q10472;F5GY99;K7EJV8	10;10;3	Polypeptide N-acetylgalactosaminyltransferase 1;Polype	GALNT1	22.2	64.218	559	7.3894	4.694	5	2.230817842	Up
Q96SL4	2	Glutathione peroxidase 7	GPX7	19.3	20.996	187	9.5031	4.6892	2	2.229341813	Up
Q9BW72	2	HIG1 domain family member 2A	HIGD2A	43.4	11.528	106	8.3671	4.6873	4	2.228757134	Up
Q14247;Q14247-3;Q14247-	18;16;15;7;6;4;3;3;3	Src substrate cortactin	CTTN	41.3	61.585	550	14.277	4.6427	17	2.21496406	Up
Q99805	4	Transmembrane 9 superfamily member 2	TM9SF2	9.8	75.775	663	7.4776	4.5279	5	2.178842096	Up
Q9Y6H1;Q5T1J5	4;3	Coiled-coil-helix-coiled-coil-helix domain-containing pr	CHCHD2;CHCHD2	33.8	15.512	151	15.061	4.3887	6	2.133793655	Up
Q9BTD8;K7EP90;Q9BTD8-3	2;2;2;2;2;2;1	RNA-binding protein 42	RBM42	5.8	50.413	480	8.9049	4.3885	2	2.133727907	Up
P00533;E9PFD7;Q504U8;P0	38;37;34;20;20;14;5;	Epidermal growth factor receptor	EGFR	39.6	134.28	1210	14.194	4.3722	44	2.128359397	Up
Q9P2M7;Q9P2M7-2;A6PVL	5;2;1	Cingulin	CGN	4.7	136.38	1197	6.4379	4.346	4	2.119688175	Up
O00400;H7C562	4;2	Acetyl-coenzyme A transporter 1	SLC33A1	10.6	60.908	549	9.422	4.1719	5	2.060704577	Up
Q9NZW5;B8ZZG1;C9J4Q3;C	7;7;4;2	MAGUK p55 subfamily member 6	MPP6	16.3	61.116	540	11.516	4.1658	6	2.058593577	Up
P33947;P33947-2;H7BYF7	5;4;1	ER lumen protein retaining receptor 2	KDELR2	31.6	24.422	212	6.3241	4.161	19	2.056930288	Up
P49006	2	MARCKS-related protein	MARCKSL1	14.4	19.529	195	8.1279	4.1372	2	2.048654702	Up
Q8IV08	6	Phospholipase D3	PLD3	14.7	54.705	490	10.483	4.0944	7	2.033652052	Up
Q9H0U4;E9PLD0;Q92928	10;8;7	Ras-related protein Rab-1B;Putative Ras-related protein	RAB1B;RAB1C	65.7	22.171	201	10.322	4.0635	20	2.022722894	Up
P06753-2;Q5VU66;Q5VU59	24;22;22;20;20;18;18;12;10;6;6;5;4;4		TPM3	66.1	29.032	248	8.1426	4.0189	26	2.00680068	Up
Q6UW68;K7ELQ9;K7EPR0;K	3;3;2;2;1;1	Transmembrane protein 205	TMEM205	20.6	21.198	189	7.4185	4.0102	2	2.00367419	Up
Q99442;F8WF48;F8WCJ7;F	4;4;3;1	Translocation protein SEC62	SEC62	10	45.861	399	10.229	3.9677	5	1.988302947	Up
Q7Z3K3;B7ZBY5;Q7Z3K3-3;	9;9;9;9;9;9;6;2;2;1;1;	Pogo transposable element with ZNF domain	POGZ	8.4	155.34	1410	7.02	3.9536	7	1.983166916	Up
P20337	4	Ras-related protein Rab-3B	RAB3B	26	24.758	219	6.5389	3.9127	3	1.968164498	Up
Q9BXK5;B7Z238;Q9BXK5-2	1;1;1	Bcl-2-like protein 13	BCL2L13	1.9	52.723	485	7.5822	3.9041	2	1.964990007	Up
D6RE79;Q14728;D6RA47;D	2;2;2;2	Major facilitator superfamily domain-containing protein	MFSD10	4.6	50.431	480	10.031	3.895	3	1.961623328	Up
Q9UBR2	3	Cathepsin Z	CTSZ	12.5	33.868	303	9.297	3.8823	2	1.956911605	Up
P60602;P60602-2	2;1	Reactive oxygen species modulator 1	ROMO1	31.6	8.1828	79	6.5156	3.8645	3	1.950281766	Up
Q9NRK6	7	ATP-binding cassette sub-family B member 10, mitochon	ABCB10	11	79.147	738	7.8257	3.8472	6	1.943808831	Up
P07355-2;P07355;H0YN42;	17;17;15;14;14;12;12	Annexin A2;Annexin;Putative annexin A2-like protein	ANXA2;ANXA2P2	54.1	40.411	357	11.175	3.8459	15	1.943321251	Up
Q14573	73	Inositol 1,4,5-trisphosphate receptor type 3	ITPR3	33.7	304.1	2671	8.631	3.8423	58	1.941970166	Up
P35251;P35251-2;H0Y8U4;E	5;5;1;1	Replication factor C subunit 1	RFC1	4.8	128.25	1148	7.2534	3.8314	4	1.937871652	Up
P50552;K7EM16;K7EQD0	4;2;1	Vasodilator-stimulated phosphoprotein	VASP	12.9	39.829	380	5.7555	3.814	3	1.931304844	Up
Q9NRZ7;Q9NRZ7-3;Q9NRZ	5;4;4;4;1;1;1	1-acyl-sn-glycerol-3-phosphate acyltransferase gamma	AGPAT3	12	43.38	376	6.7769	3.8017	3	1.92664469	Up
Q12955;Q12955-4;Q12955-	24;24;24;12;5;4;4;4;4	Ankyrin-3	ANK3	7.4	480.4	4377	7.5444	3.8012	19	1.926454935	Up
O43731-2;O43731	2;2	ER lumen protein retaining receptor 3	KDELR3	11.8	25.69	220	5.5502	3.7947	2	1.923985834	Up
O95197-3;O95197;O95197-	5;4;4;4;4;3;1;1;1	Reticulon-3	RTN3	28.4	25.609	236	11.176	3.7792	9	1.91808087	Up
Q12849;H0YAK1;H0Y8R1;F5	3;3;3;3;2;1	G-rich sequence factor 1	GRSF1	11.2	53.126	480	5.392	3.7684	3	1.913952109	Up
Q15293;B7Z1M1;E9PJD5;E9	11;10;4;3;3	Reticulocalbin-1	RCN1	42.9	38.89	331	11.315	3.768	10	1.913798965	Up
Q14011;D6W5Y5;B4E2X2;K	5;4;4;4;4;4;4;4;4;4;	Cold-inducible RNA-binding protein	CIRBP	38.4	18.648	172	7.5452	3.7546	10	1.908659217	Up
J3KQ66;P78509;P78509-2;P	44;44;44;44	Reelin	RELN	16.9	388.43	3460	8.1369	3.7445	36	1.90477309	Up
O96000;H3BPJ9;Q96II6;H3E	5;5;4;3	NADH dehydrogenase [ubiquinone] 1 beta subcomplex s	NDUFB10	34.3	20.776	172	8.8871	3.7399	5	1.902999695	Up
Q92793;Q09472	2;2	CREB-binding protein;Histone acetyltransferase p300	CREBBP;EP300	0.9	265.35	2442	6.7783	3.7252	2	1.897317884	Up
Q7Z7H5;Q7Z7H5-2;Q7Z7H5	6;6;5;3	Transmembrane emp24 domain-containing protein 4	TMED4	37.9	25.943	227	10.031	3.7135	5	1.892779578	Up
O95563;Q5R3B4	7;7	Brain protein 44	BRP44	57.5	14.279	127	9.0432	3.691	8	1.884011738	Up
Q15388	2	Mitochondrial import receptor subunit TOM20 homolog	TOMM20	18.6	16.298	145	9.18	3.6826	4	1.880724702	Up
Q9H089;H7C2X7;F8WFC6	3;2;1	Large subunit GTPase 1 homolog	LSG1	4.1	75.225	658	6.885	3.6523	3	1.868805273	Up
A6NMH8;E9PIF1;P60033;H	4;4;4;4;4;4;4;1;1	CD81 antigen	CD81	24.5	29.806	274	6.5828	3.6516	5	1.86852874	Up
Q6PKG0;Q6PKG0-3;E5RH5	7;7;4;2;2;2;2;2;2;2;	La-related protein 1	LARP1	7.8	123.51	1096	6.2573	3.6479	6	1.867066181	Up
O43504;E9PLX3	4;3	Hepatitis B virus X-interacting protein	HBXIP	74.7	9.6138	91	6.8665	3.6429	4	1.865087393	Up
O15397;O15397-2;F5H2I3	3;2;1	Importin-8	IPO8	3.5	119.94	1037	5.6073	3.6408	2	1.864255491	Up
Q9P2X0-2;Q9P2X0	2;2	Dolichol-phosphate mannosyltransferase subunit 3	DPM3	18	13.277	122	6.1289	3.6351	3	1.861995053	Up
O14832;B1ALH6;A8MTS8;C	5;5;3;2;2	Phytanoyl-CoA dioxygenase, peroxisomal	PHYH	14.8	38.538	338	7.8504	3.6219	3	1.856746714	Up
P78332;E9PGM9;F8WCA5;E	3;3;3;3	RNA-binding protein 6	RBM6	2.2	128.64	1123	5.9914	3.6144	3	1.853756176	Up
Q14697;F5H6X6	53;47	Neutral alpha-glucosidase AB	GANAB	71	106.87	944	10.926	3.6109	2	1.852358467	Up
P01891	14	HLA class I histocompatibility antigen, A-68 alpha chain	HLA-A	44.7	40.908	365	11.046	3.5887	2	1.843461325	Up
P61165;F5GWH5	1;1	UPF0197 transmembrane protein C11orf10	C11orf10	10.1	9.0788	79	6.2143	3.5794	5	1.839717775	Up
Q13813	190	Spectrin alpha chain, brain	SPTAN1	75.1	284.54	2472	9.0993	3.5709	335	1.836287733	Up
P55011;P55011-3;G3XAL9;E	33;32;29;1;1;1;1;1	Solute carrier family 12 member 2	SLC12A2	33.1	131.45	1212	11.04	3.5666	27	1.834549424	Up
I3L0Q9;P04062;P04062-2;J3	7;7;7;7;6;6;4	Glucosylceramidase	GBA	14.6	59.663	536	7.5444	3.5446	5	1.825622832	Up
P39060;P39060-1;P39060-2	27;27;27;17;12	Collagen alpha-1(XVIII) chain;Endostatin	COL18A1	21.5	178.19	1754	10.835	3.5406	24	1.823993864	Up
Q8NFC6	15	Biorientation of chromosomes in cell division protein 1-l	BOD1L1	7	330.46	3051	8.2046	3.5371	10	1.822567007	Up
P27487;F8WE17;F8WBB6;H	25;5;3;2	Dipeptidyl peptidase 4;Dipeptidyl peptidase 4 membran	DPP4	35.2	88.278	766	9.4071	3.5185	41	1.814960513	Up
E7EX17;P23588;B4DS13;F8	4;4;4;2;1;1;1	Eukaryotic translation initiation factor 4B	EIF4B	9.9	69.697	616	6.9968	3.5092	4	1.811142174	Up
O00468;H0Y5U1	16;2	Agrin	AGRN	9.2	214.84	2045	7.54	3.5	11	1.807354922	Up
Q6L8Q7;Q6L8Q7-2;F6T1Q0	4;4;4	2,5-phosphodiesterase 12	PDE12	9.5	67.351	609	5.298	3.4995	3	1.807148808	Up
Q99856;A6NKF2	3;1	AT-rich interactive domain-containing protein 3A	ARID3A	10.1	62.888	593	4.8408	3.4955	5	1.805498835	Up
Q5VV89;O14880;Q5VV87	4;4;2	Microsomal glutathione S-transferase 3	MGST3	47.6	18.416	166	8.7647	3.4872	4	1.802069109	Up
Q9NXB9;F6SH78;Q9NYP7;E	3;1;1;1	Elongation of very long chain fatty acids protein 2	ELOVL2	13.2	34.584	296	6.8867	3.4766	5	1.797677087	Up
Q9BYC9;B7Z746;I3L2X1	7;2;2	39S ribosomal protein L20, mitochondrial	MRPL20	41.6	17.442	149	6.7165	3.4719	4	1.795725395	Up
Q7Z6E9;Q7Z6E9-2;H3BSK8;	3;3;1;1	E3 ubiquitin-protein ligase RBBP6	RBBP6	1.7	201.56	1792	3.6755	3.4656	3	1.793105148	Up
Q5SSJ5;Q5SSJ5-2;Q5SWC8;	4;3;3;3;2;1;1;1	Heterochromatin protein 1-binding protein 3	HP1BP3	8.7	61.206	553	8.1773	3.4609	3	1.791147257	Up
Q9HA82;H0YN04;H0YNR6;	3;3;3;3;3;2;2	Ceramide synthase 4	CERS4	10.9	46.398	394	6.1409	3.4485	2	1.785968967	Up
P48681	50	Nestin	NES	38.3	177.44	1621	8.6758	3.4461	46	1.784964567	Up
C9JC84;P02679;C9JEU5;P02	5;5;5;5;2;2	Fibrinogen gamma chain	FGG	12.8	52.337	461	7.9893	3.4367	4	1.78102392	Up
Q9HD45;Q5TB53	7;6	Transmembrane 9 superfamily member 3	TM9SF3;RP11-34E	14.8	67.887	589	6.8057	3.4186	6	1.773405627	Up
O00116;B8ZZ81	20;5	Alkyldihydroxyacetonephosphate synthase, peroxisoma	AGPS	43.5	72.911	658	8.0057	3.4157	19	1.772181269	Up
A8MV58;Q16643;Q16643-2	20;20;17;15;7;5;5;3	Drebrin	DBN1	37.6	76.299	695	10.616	3.4058	15	1.767993718	Up
Q13445;K7EQ63;K7EIN4	4;2;2	Transmembrane emp24 domain-containing protein 1	TMED1	21.1	25.206	227	8.4147	3.3968	6	1.764176276	Up
P18583-9;P18583-5;P18583	14;14;14;14;14;14;14	Protein SON	SON	9.4	268.09	2466	6.9649	3.3966	10	1.764091329	Up
P20674;H3BNX8;H3BV69;H	7;6;5;4;1	Cytochrome c oxidase subunit 5A, mitochondrial	COX5A	38	16.762	150	8.0027	3.3655	8	1.750820857	Up
Q13123;E7EQZ7;D6REL4	4;1;1	Protein Red	IK	6.1	65.601	557	7.318	3.363	2	1.749748779	Up
O43676;C9JKQ2	2;1	NADH dehydrogenase [ubiquinone] 1 beta subcomplex s	NDUFB3	21.4	11.402	98	6.9748	3.3541	2	1.745925702	Up

F2Z2X0;Q4TT34;O00746;H0	3;3;3;2;2;1;1	Nucleoside diphosphate kinase;Nucleoside diphosphate	NME4	13.3	25.46	233	9.6028	3.3455	2	1.742221844	Up
F8VX04;Q9H2H9	2;2	Sodium-coupled neutral amino acid transporter 1	SLC38A1	6.8	56.195	503	6.2405	3.344	2	1.741574847	Up
O14776;O14776-2;G3V220	12;12;9	Transcription elongation regulator 1	TCERG1	11.7	123.9	1098	9.3732	3.3364	5	1.738292263	Up
Q9UGP8	3	Translocation protein SEC63 homolog	SEC63	5.5	87.996	760	8.4628	3.336	2	1.738119289	Up
Q9BQ48	4	39S ribosomal protein L34, mitochondrial	MRPL34	26.1	10.165	92	6.215	3.3343	5	1.737383915	Up
Q8N8S7;Q8N8S7-2;Q9UI08	10;10;1	Protein enabled homolog	ENAH	25.7	66.509	591	7.9748	3.3267	12	1.734091771	Up
F5H301;Q9UDY2;Q9UDY2-3	30;30;30;29;26;26;24	Tight junction protein ZO-2	TJP2	28.8	137.34	1221	9.6263	3.324	22	1.732920382	Up
P45880-1;P45880;P45880-2	6;6;6;5;3;3;1	Voltage-dependent anion-selective channel protein 2	VDAC2	23	33.371	309	7.9947	3.3175	5	1.730096466	Up
P20336;Q96E17	4;1	Ras-related protein Rab-3A	RAB3A	22.7	24.984	220	8.7035	3.3169	2	1.729835517	Up
Q27J81;Q27J81-2;H7BXE4;(	45;44;17;7	Inverted formin-2	INF2	48.8	135.62	1249	10.132	3.3093	55	1.726526083	Up
Q92930;H0YNE9;F5GY21;H(	8;7;6;4;2;2	Ras-related protein Rab-8B	RAB8B	42.5	23.584	207	10.838	3.2989	4	1.721985046	Up
Q9UKR5	4	Probable ergosterol biosynthetic protein 28	C14orf1	28.6	15.864	140	5.7872	3.296	5	1.720716243	Up
Q9Y520-7;Q9Y520;Q9Y520-	4;4;4;4;4;4;4;4	Protein PRRC2C	PRRC2C	1.2	317.08	2898	6.5273	3.2958	3	1.720628698	Up
Q9H7F0;Q9H7F0-2	9;6	Probable cation-transporting ATPase 13A3	ATP13A3	10.1	138.04	1226	7.0063	3.2864	7	1.716508087	Up
O14967;D6RAZ4	9;4	Calmegin	CLGN	18.9	70.038	610	11.404	3.2861	8	1.716376384	Up
Q9P0S9	3	Transmembrane protein 14C	TMEM14C	50	11.564	112	6.3644	3.2823	5	1.714707106	Up
Q02978;I3L1P8;F5GY65	10;10;8	Mitochondrial 2-oxoglutarate/malate carrier protein	SLC25A11	36.9	34.061	314	8.0309	3.274	11	1.711054322	Up
Q9BU76;Q9BU76-4;Q9BU76	2;2;2;1	Multiple myeloma tumor-associated protein 2	MMTAG2	8.7	29.411	263	6.0515	3.2712	2	1.709819968	Up
Q5T7F6;Q9P0B6	2;2	Coiled-coil domain-containing protein 167	CCDC167	20.2	12.728	114	7.6074	3.2683	3	1.708540416	Up
P58511	2	Protein FAM165B	FAM165B	46.6	6.8862	58	7.0757	3.26	2	1.704871964	Up
O14521;E9PIC0;E9PIG3;H3E	3;3;1;1;1;1;1;1	Succinate dehydrogenase [ubiquinone] cytochrome b sm	SDHD	27	17.043	159	5.1297	3.2578	3	1.703898038	Up
Q8WWM7;Q8WWM7-3;H3	7;6;6;6;6;6;6;6;3;3;	Ataxin-2-like protein	ATXN2L	8.2	113.37	1075	5.81	3.2475	6	1.699329526	Up
Q13136;Q13136-2;E9PJZ7;E	4;4;3;2;1;1;1;1;1;1;	Liprin-alpha-1	PPFIA1	3.7	135.78	1202	5.5101	3.2405	2	1.696216434	Up
Q09666;Q09666-2;E9PLK4;E	203;3;3;3;3;3;3;3;1	Neuroblast differentiation-associated protein AHNAK	AHNAK	61	629.09	5890	7.1783	3.2094	282	1.68230361	Up
F5H1Z9;Q05682-4;E9PGZ1;(	5;5;5;5;4;4;4;4;4	Caldesmon	CALD1	12.5	62.711	543	6.1467	3.2094	5	1.68230361	Up
O75947;O75947-2;F5H608	13;9;4	ATP synthase subunit d, mitochondrial	ATP5H	78.9	18.491	161	8.2752	3.1997	15	1.677936646	Up
Q96EH3	3	Mitochondrial assembly of ribosomal large subunit prote	MALSU1	18.4	26.17	234	4.6453	3.1962	3	1.676357687	Up
P63173;J3QL01;J3KT73;J3K!	6;5;5;3	60S ribosomal protein L38	RPL38	50	8.2178	70	5.0675	3.1896	10	1.67337551	Up
E9PDI4;O00515;H0Y901	2;2;1	Ladinin-1	LAD1	4.9	58.677	531	6.88	3.1885	3	1.672877881	Up
O75394	2	39S ribosomal protein L33, mitochondrial	MRPL33	38.5	7.6191	65	5.2744	3.1848	5	1.671202777	Up
Q9H8G2;Q9H8G2-2	1;1	Caspase activity and apoptosis inhibitor 1	CAAP1	2.2	38.367	361	7.2108	3.181	2	1.669480372	Up
Q99643;Q99643-3;Q99643-	4;4;4	Succinate dehydrogenase cytochrome b560 subunit, mito	SDHC	36.7	18.61	169	4.8089	3.1735	4	1.666074843	Up
P35221;P35221-2;G3XAM7;	51;49;45;44;30;13;11	Catenin alpha-1	CTNNA1	71.3	100.07	906	10.056	3.1682	80	1.663663412	Up
P35520-2;P35520;F5H2U1;H	6;6;5;4;2;1;1	Cystathionine beta-synthase	CBS	16.5	61.862	565	6.2137	3.1677	2	1.66343571	Up
Q32M63;Q6ZMG9	2;2	Ceramide synthase 6	LASS6;CERS6	4.8	45.793	392	6.1627	3.1657	2	1.662524544	Up
D6RF35;P02774-3;P02774;P	17;16;16;14;9;2	Vitamin D-binding protein	GC	48.1	53.02	476	8.7939	3.1617	17	1.660700483	Up
E7EW20;Q9UM54-6;Q9UM!	8;8;7;7;7;7;7;1	Unconventional myosin-VI	MYO6	7.9	148.83	1286	5.1465	3.1402	2	1.650856448	Up
Q9BYC8;B3KQX1	6;1	39S ribosomal protein L32, mitochondrial	MRPL32	37.8	21.405	188	5.7571	3.1328	8	1.64745267	Up
Q9BUN8;Q9BUN8-2;B4E1G	5;5;5;2	Derlin-1	DERL1	23.9	28.8	251	7.1073	3.1321	5	1.647130275	Up
Q9BTC0	3	Death-inducer obliterator 1	DIDO1	1.5	243.87	2240	5.0351	3.1308	3	1.64653135	Up
Q92604	7	Acyl-CoA:lysophosphatidylglycerol acyltransferase 1	LPGAT1	25.9	43.089	370	5.3471	3.1228	8	1.642840175	Up
Q5VW38;G5E994;Q5VW38-	4;4;4;2	Protein GPR107	GPR107	6.7	66.99	600	4.3921	3.116	2	1.639695233	Up
P03928	4	ATP synthase protein 8	MT-ATP8	52.9	7.9916	68	8.1927	3.1001	3	1.632314753	Up
Q9Y3R5;Q9Y3R5-2	2;2	Protein dopey-2	DOPEY2	1.1	258.23	2298	4.8482	3.091	2	1.628073654	Up
Q9BW60;B4DP24	3;3	Elongation of very long chain fatty acids protein 1	ELOVL1	12.2	32.662	279	4.2766	3.086	3	1.625738062	Up
O95202;O95202-3;O95202-	23;6;6	LETM1 and EF-hand domain-containing protein 1, mitoch	LETM1	45.5	83.353	739	8.4379	3.076	27	1.621055503	Up
P62875	2	DNA-directed RNA polymerases I, II, and III subunit RPAE	POLR2L	29.9	7.645	67	3.3599	3.069	5	1.617768646	Up
P67936;P67936-2;K7ENT6;K	18;15;11;10;6;5;4	Tropomyosin alpha-4 chain	TPM4	54	28.521	248	6.8781	3.0631	11	1.614992467	Up
P13073;H3BPG0;H3BNV9;H	8;6;5;5;4;1	Cytochrome c oxidase subunit 4 isoform 1, mitochondrial	COX4I1	42	19.576	169	8.8831	3.0602	13	1.613625944	Up
E1P660;J3QRS9;O43670;O4	3;3;3;3;2;2;1;1;1;1;1	Zinc finger protein 207	ZNF207	9.3	52.693	494	7.2012	3.06	3	1.613531653	Up
P21589;P21589-2;Q96B60;H	8;8;5;1;1	5-nucleotidase	NT5E	25.1	63.367	574	7.3262	3.0521	11	1.609802232	Up
P39748;F5H1Y3;I3L3E9	2;1;1	Flap endonuclease 1	FEN1	8.2	42.592	380	8.9764	3.0419	2	1.604972726	Up
Q8IZE3;Q8IZE3-2	4;4	Protein-associating with the carboxyl-terminal domain o	SCYL3	7.5	82.857	742	4.5051	3.0393	3	1.603739086	Up
Q9NS69	3	Mitochondrial import receptor subunit TOM22 homolog	TOMM22	31	15.521	142	7.2676	3.0292	4	1.598936834	Up
P28715;P28715-2	2;1	DNA repair protein complementing XP-G cells	ERCC5	1.9	133.11	1186	4.9559	3.026	2	1.597411988	Up
P21397;B4DF46	4;3	Amine oxidase [flavin-containing] A	MAOA	8	59.681	527	4.5016	3.0017	2	1.585779796	Up
Q4U2R6	3	39S ribosomal protein L51, mitochondrial	MRPL51	22.7	15.095	128	5.4537	2.9988	5	1.584385307	Up
Q9HB90;Q9NQL2;Q9NQL2-	4;3;2	Ras-related GTP-binding protein C;Ras-related GTP-bindi	RRAGC;RRAGD	12	44.223	399	5.0586	2.9938	4	1.581977846	Up
C9JLE3;P51654;G3V1R0;H0Y	9;9;7;6;1	Glypican-3;Secreted glypican-3	GPC3	20.1	68.413	603	6.3239	2.991	21	1.58062791	Up
O00214-2;O00214;F6V2D4;	2;2;2;1;1;1;1;1;1	Galectin-8	LGALS8	6.4	40.397	359	5.032	2.9853	2	1.577875919	Up
Q96G23;Q5SZE1;H0YNU7;C	8;7;6;5;5;5;4;3;3	Ceramide synthase 2	CERS2;LASS2	24.5	44.876	380	6.0694	2.9731	6	1.571967989	Up
Q96Q06-2;Q96Q06	10;10	Perilipin-4	PLIN4	15.3	140.67	1423	5.2466	2.9725	8	1.57167681	Up
Q14157-5;Q14157;F8W726;	7;7;7;7;7;7;4;2;2;2;2;	Ubiquitin-associated protein 2-like	UBAP2L	9.8	116.64	1104	4.5264	2.9689	9	1.569928501	Up
P12270;Q5SWX9	119;5	Nucleoprotein TPR	TPR	49.8	267.29	2363	6.7207	2.9684	198	1.569685512	Up
Q01740;B7Z3P4;Q5QPT3	4;2;2	Dimethylaniline monooxygenase [N-oxide-forming] 1	FMO1	8.3	60.31	532	5.3454	2.9587	3	1.564963421	Up
O14976;E9PGR2;H0Y8H5;D(	3;3;1;1;1;1;1	Cyclin-G-associated kinase	GAK	4.2	143.19	1311	5.69	2.9535	3	1.562425612	Up
Q9UQ35;Q9UQ35-2;I3L2L6;	35;19;19;7;5;5;5;4;1	Serine/arginine repetitive matrix protein 2	SRRM2	18.2	299.61	2752	7.8856	2.9513	54	1.561350578	Up
Q14315;Q14315-2	29;29	Filamin-C	FLNC	12.1	291.02	2725	5.641	2.9424	10	1.556993385	Up
Q96GC5;B4DN34;F5H702;F!	6;6;6;2	39S ribosomal protein L48, mitochondrial	MRPL48	39.6	23.934	212	6.4891	2.9422	9	1.556895319	Up
Q9UBT7;Q9UBT7-2;Q9UBT	4;4;3;1;1	Alpha-catulin	CTNNAL1	8.3	81.895	734	5.0393	2.9421	5	1.556846284	Up
Q14249	4	Endonuclease G, mitochondrial	ENDOG	17.5	32.62	297	5.3584	2.9347	4	1.553213031	Up
P08648;H0YIV7	18;3	Integrin alpha-5;Integrin alpha-5 heavy chain;Integrin alp	ITGA5	23.7	114.54	1049	7.7857	2.9315	20	1.551639057	Up
D3DNT2;E7EUU4;E9PGM1;E	41;41;41;41;40;40;40	Eukaryotic translation initiation factor 4 gamma 1	EIF4G1	30.1	175.62	1600	8.5087	2.9298	44	1.550802184	Up
P16383;P16383-2	2;2	GC-rich sequence DNA-binding factor 2	GCFC2	3.5	89.384	781	4.1778	2.9297	2	1.550752941	Up
Q9UHQ9;H7C0R7	10;9	NADH-cytochrome b5 reductase 1	CYB5R1	35.1	34.094	305	7.7434	2.9274	10	1.549619889	Up
P42771-4;P42771;J3QRG6;K	3;3;3;2;2;2;1;1;1	Cyclin-dependent kinase inhibitor 2A, isoforms 1/2/3	CDKN2A	24.6	17.883	167	6.8085	2.9266	3	1.549225575	Up
P53701	9	Cytochrome c-type heme lyase	HCCS	39.9	30.601	268	7.4904	2.926	7	1.548929769	Up
Q01628;E9PS44;Q01629;P1	3;3;2;2;2;2	Interferon-induced transmembrane protein 3;Interferon	IFITM3;IFITM2;IFI	31.6	14.632	133	6.3715	2.9212	4	1.546561136	Up
O15162;C9J7K9;B4DTE8;C9.	2;2;2;1;1;1;1	Phospholipid scramblase 1	PLSCR1	7.2	35.049	318	5.2061	2.9189	2	1.545424786	Up
Q5HYI8;C9JXM3;F8WDC7;F	2;1;1;1;1;1	Rab-like protein 3	RABL3	9.3	26.422	236	4.6367	2.9069	4	1.539481442	Up
O75964;E9PN17	6;6	ATP synthase subunit g, mitochondrial	ATP5L	47.6	11.428	103	8.9566	2.8985	9	1.535306486	Up
Q13084;Q4TT38;A2IDC6;Q4	11;11;10;9;7	39S ribosomal protein L28, mitochondrial	MRPL28	59.4	30.156	256	6.5917	2.894	12	1.533064922	Up
A4D1E9;A4D1E9-2;C9JEQ8;	6;5;4;4;4;2;2;2;2	GTP-binding protein 10	GTPBP10	17.1	42.932	387	5.3538	2.8893	6	1.530720009	Up
P56385	5	ATP synthase subunit e, mitochondrial	ATP5I	62.3	7.9331	69	6.5701	2.8876	11	1.529870909	Up
Q9Y320;Q9Y320-2;G3V155;	9;8;4;4;4;4;4	Thioredoxin-related transmembrane protein 2	TMX2	41.2	34.037	296	7.8758	2.8872	11	1.529671048	Up
Q96DA6;C9JBV1;F2Z3A7;G!	7;5;4;3	Mitochondrial import inner membrane translocase subu	DNAJC19	63.8	12.498	116	5.1351	2.8818	7	1.526970214	Up
P24539;Q5QNZ2	16;14	ATP synthase subunit b, mitochondrial	ATP5F1	54.3	28.908	256	8.468	2.8792	25	1.525668007	Up
G3V4K0;O95406;G3V3B2;G	1;1;1;1	Protein cornichon homolog	CNIH	15.6	18.562	160	5.7281	2.8754	2	1.523762665	Up
Q9Y666;H0YB78;Q9Y666-2;(	15;6;3;1;1	Solute carrier family 12 member 7	SLC12A7	19.8	119.1	1083	7.5366	2.8727	15	1.522407338	Up
G3V180;G3V1D3;Q9NY33;C	2;2;2;2;1;1;1;1	Dipeptidyl peptidase 3	DPP3	3.6	84.375	757	5.2849	2.8686	2	1.520346812	Up
Q8TAT6-2;Q8TAT6;B4DG89	2;2;1;1;1	Nuclear protein localization protein 4 homolog	NPLOC4	5.7	69.46	617	4.4233	2.851	2	1.511468039	Up
O00192;E9PDC3;O00192-2;	2;2;2;2	Armadillo repeat protein deleted in velo-cardio-facial sy	ARVCF	3.1	104.64	962	4.3957	2.8492	2	1.510556895	Up
O75376;O75376-2;E7EVK1;I	4;3;2;2;2;2;2;1	Nuclear receptor corepressor 1	NCOR1	2.3	270.21	2440	5.4456	2.8463	3	1.50908773	Up
Q96A33;Q96A33-2;J3QR89	14;13;3	Coiled-coil domain-containing protein 47	CCDC47	31.9	55.873	483	7.9977	2.8456	16	1.50873288	Up
O75352;J3QRD5;J3QW43;B	4;4;4;4;4;4;4;3;3;3;2;	Mannose-P-dolichol utilization defect 1 protein	MPDU1	21.1	26.638	247	4.8277	2.8387	12	1.50523039	Up
O75911;O75911-2;Q5SUY4	10;4;4	Short-chain dehydrogenase/reductase 3	DHRS3	46.7	33.548	302	6.3482	2.8356	10	1.503654035	Up
P49768;P49768-2;E7ES96;P	4;4;4;3;2;2;2;2;2;1;1;	Presenilin-1;Presenilin-1 NTF subunit;Presenilin-1 CTF su	PSEN1;PSEN2	10.9	52.667	467	5.9066	2.8299	3	1.500751074	Up
Q86Y39;C9JT23;Q86Y39-2;K	5;3;3;3;3;3;3	NADH dehydrogenase [ubiquinone] 1 alpha subcomplex	NDUFA11	48.2	14.852	141	5.2313	2.8273	3	1.499424973	Up
Q9NRY2;Q9NRY2-2	3;1	SOSS complex subunit C	SSBIP1	42.3	11.425	104	6.4679	2.8251	3	1.498301935	Up
E9PDL9;B5M453;Q9Y6M7;E	2;2;2;2;2;2;2;2;2;2;2;	Sodium bicarbonate cotransporter 3	SLC4A7	1.7	140.76	1259	4.6863	2.8196	2	1.495490511	Up
Q8TCC3-2;Q8TCC3;Q8TCC3	3;3;2;2	39S ribosomal protein L30, mitochondrial	MRPL30	19.9	21.838	191	7.2078	2.8185	3	1.494927567	Up
Q8NBM4;Q8NBM4-2;Q8NE	5;4;4;4;1;1	Ubiquitin-associated domain-containing protein 2	UBAC2	17.4	38.963	344	5.2764	2.8093	4	1.490210695	Up
O43490;O43490-2;O43490-	8;8;8;8;8;8;8;1;1	Prominin-1	PROM1	14.6	97.201	865	5.6091	2.8084	6	1.489748433	Up
O95865;Q5SRR8;Q5SSV3;A	4;3;3;3;2	N(G),N(G)-dimethylarginine dimethylaminohydrolase 2	DDAH2	20.7	29.644	285	4.931	2.8064	4	1.488720653	Up
P07686;Q5URX0;H0Y9B6;H(	5;4;4;3	Beta-hexosaminidase subunit beta;Beta-hexosaminidase	HEXB	11.9	63.111	556	4.0304	2.7952	2	1.482951513	Up
Q10471;G3V1S6;B7Z8V8	7;7;5	Polypeptide N-acetylgalactosaminyltransferase 2;Polype	GALNT2	14.2	64.732	571	7.0949	2.7952	6	1.482951513	Up
Q96RT1-8;Q96RT1;Q96RT1-	4;4;4;4;4;4;4;4;4;3	Protein LAP2	ERBB2IP	4.8	159.02	1419	4.4713	2.7941	4	1.482383655	Up
Q9HD33;Q9HD33-2;Q9HD3	9;9;5	39S ribosomal protein L47, mitochondrial	MRPL47	35.6	29.45	250	6.8591	2.7891	12	1.479799661	Up
G8JLI3;Q5H9R7-5;Q5H9R7;(	4;4;4;4;4;4;4;4;3;2;	Serine/threonine-protein phosphatase 6 regulatory subu	PPP6R3	6.5	98.268	879	4.2076	2.7882	6	1.479334051	Up
Q4G0N4;Q4G0N4-2;B7Z8V	14;12;10;10;3;2	NAD kinase domain-containing protein 1	NADKD1	39.8	49.432	442	8.1145	2.7875	16	1.478971805	Up
E9PR17;P13987;E9PNW4;H	4;4;4;2	CD59 glycoprotein	CD59	24.6	14.529	130	8.8349	2.7852	9	1.477780929	Up

A6NH11	3	Glycolipid transfer protein domain-containing protein 2	GLTPD2	13.4	31.641	291	4.9635	2.7757	3	1.472851649	Up
Q9BQC6	1	Ribosomal protein 63, mitochondrial	MRP63	12.7	12.266	102	6.9336	2.7754	2	1.472695713	Up
Q9NPL8;G3XA94;C9JU35;C9	6;3;3;2;1;1;1;1	Translocase of inner mitochondrial membrane domain-co	TIMMDC1	29.8	32.177	285	4.275	2.7686	5	1.469156632	Up
Q969N2;Q969N2-5;G8JLF5;	6;6;4;4;4;3;3;2	GPI transamidase component PIG-T	PIGT	11.2	65.699	578	4.7302	2.7657	4	1.467644673	Up
Q14160-3;Q14160;Q14160-	24;24;21;7	Protein scribble homolog	SCRIB	21.8	177.69	1655	6.0822	2.7598	18	1.46456372	Up
Q14108;E7EM68	2;1	Lysosome membrane protein 2	SCARB2	6.7	54.29	478	4.9208	2.7567	2	1.462942273	Up
O15047;C9J2Z9	4;3	Histone-lysine N-methyltransferase SETD1A	SETD1A	3.2	186.03	1707	4.0587	2.7524	5	1.460690149	Up
Q92797;Q92797-2	24;14	Symplekin	SYMPK	25.6	141.15	1274	6.425	2.7521	20	1.460532893	Up
O00461;F8W785	6;6	Golgi integral membrane protein 4	GOLIM4	9.3	81.879	696	4.864	2.7501	4	1.459484079	Up
Q5BJD5;E9PJ42;Q5BJD5-2;E	6;4;4;4	Transmembrane protein 41B	TMEM41B	27.1	32.513	291	8.8994	2.7477	5	1.458224496	Up
P10909-2;P10909-5;P10909	5;5;5;5;5;5;3;3;1;1	Clusterin;Clusterin beta chain;Clusterin alpha chain;Clust	CLU	15.2	57.832	501	7.66	2.7279	9	1.447790759	Up
Q9BTT6;Q9BTT6-2;Q5T0G3	11;5;4	Leucine-rich repeat-containing protein 1	LRRC1	31.9	59.241	524	4.6228	2.7226	9	1.44498504	Up
Q9P032	6	NADH dehydrogenase [ubiquinone] 1 alpha subcomplex	NDUFAF4	37.7	20.266	175	5.3525	2.7222	7	1.444773066	Up
O75616;J3QRV9;J3QSB2;O7	5;4;4;4;2;1	GTPase Era, mitochondrial	ERAL1	15.6	48.349	437	6.9772	2.7205	6	1.443871828	Up
H0YK83;P67812;H0YK72;H0	5;5;5;5;3;3;3;2	Signal peptidase complex catalytic subunit SEC11A	SEC11A;SEC11L1	29.7	21.454	185	7.7629	2.7183	9	1.442704685	Up
Q13393;Q13393-2;Q13393-	5;5;5;3;2;1	Phospholipase D1	PLD1	6.4	124.18	1074	4.2841	2.709	4	1.437760394	Up
Q9H9B4;D6RFI0;D6RDG7;D	9;5;4;2	Sideroflexin-1	SFXN1	41.3	35.619	322	7.1298	2.7071	8	1.436748182	Up
Q16718;Q5H9R2;H7BYD0;C	6;6;6;3;3	NADH dehydrogenase [ubiquinone] 1 alpha subcomplex	NDUFA5;DKFZp78	52.6	13.459	116	6.7307	2.7066	5	1.436481692	Up
P55196-5;J3KN01;A8MQ02	42;42;42;42;42;42;39	Afadin	MLLT4	32	207.79	1834	7.0139	2.7006	38	1.433279971	Up
Q99624	1	Sodium-coupled neutral amino acid transporter 3	SLC38A3	3.2	55.772	504	3.963	2.6894	4	1.427284346	Up
Q96A26;F8W7Q4;E9PH05	7;7;5	Protein FAM162A	FAM162A	37.7	17.342	154	6.432	2.6864	13	1.425674135	Up
Q8NHG7	2	Small VCP/p97-interacting protein	SVIP	32.5	8.4426	77	7.3751	2.682	2	1.423309237	Up
E2QRJ6;Q9H1A6;P52435;Q	3;3;3;3;2;2;2;1;1;1;1;	DNA-directed RNA polymerase II subunit RPB11-a;DNA-d	POLR2J3;POLR2J1	21.5	17.189	158	4.3458	2.68	2	1.422233001	Up
Q6PD62;H0YCE8	14;4	RNA polymerase-associated protein CTR9 homolog	CTR9	15.3	133.5	1173	5.5908	2.6799	13	1.422179168	Up
Q96KR1;H0Y8W1	3;1	Zinc finger RNA-binding protein	ZFR	3.4	117.01	1074	4.3937	2.6782	2	1.421263701	Up
P55285;P55285-2;D6RF86	5;4;4	Cadherin-6	CDH6	6.5	88.308	790	5.9685	2.6771	4	1.42067103	Up
J3KQ83;P18859;A8MUH2	4;4;2	ATP synthase-coupling factor 6, mitochondrial	ATP5J	41.4	13.388	116	6.648	2.677	3	1.420617139	Up
Q96JM3	3	Chromosome alignment-maintaining phosphoprotein 1	CHAMP1	3.6	89.098	812	3.5081	2.6769	3	1.420563246	Up
P36542;P36542-2;B4DL14	9;8;8	ATP synthase subunit gamma, mitochondrial;ATP synthas	ATP5C1	39.3	32.996	298	8.0851	2.6766	15	1.420401554	Up
E7ERS3;Q86VM9;Q86VM9-	4;4;4;1;1	Zinc finger CCCH domain-containing protein 18	ZC3H18	4.5	108.94	977	7.7055	2.6639	3	1.413539926	Up
Q9UHA4;J3KMX4	4;4	Ragulator complex protein LAMTOR3	LAMTOR3	33.9	13.623	124	8.0538	2.6633	4	1.413214946	Up
P49790;F6QR24	23;22	Nuclear pore complex protein Nup153	NUP153	23.3	153.94	1475	6.2092	2.6611	21	1.412022726	Up
P51809;P51809-2;P51809-3	5;4;4;1	Vesicle-associated membrane protein 7	VAMP7	25	24.935	220	5.0222	2.6547	3	1.408548835	
Q8WVK2;B8ZZ98	3;1	U4/U6.U5 small nuclear ribonucleoprotein 27 kDa protein	SNRNP27	17.4	18.86	155	4.749	2.6542	4	1.408277085	
P35610;A8K3P4;B4DU95;B1	5;4;4;3;1	Sterol O-acyltransferase 1	SOAT1	13.8	64.734	550	3.752	2.6536	5	1.407950917	
Q9NYK5-2;Q9NYK5;C9JG87	9;9;8	39S ribosomal protein L39, mitochondrial	MRPL39	32	40.457	353	5.9137	2.6508	7	1.406427825	
F5H2M7;Q9H4A3-6;Q9H4A	14;14;14;14;14;14;11	Serine/threonine-protein kinase WNK1	WNK1	5.3	304.64	2880	5.6717	2.6473	9	1.404521695	
Q96S52;Q96S52-2;F5H019	6;6;4	GPI transamidase component PIG-S	PIGS	18.4	61.655	555	4.7035	2.6452	8	1.403376807	
P11233;H7C3P7;C9JPE8	6;4;2	Ras-related protein Ral-A	RALA	29.6	23.567	206	6.6648	2.642	4	1.401630467	
P42224;J3KPM9;P42224-2;E	3;3;3;1;1;1	Signal transducer and activator of transcription 1-alpha/b	STAT1	4.8	87.334	750	6.5206	2.6419	3	1.401575859	
Q8TBQ9	2	Protein kish-A	TMEM167A	25	8.0598	72	4.4484	2.6346	6	1.39758394	
Q9Y2R0;K7EPV0	7;6	Coiled-coil domain-containing protein 56	CCDC56	47.2	11.731	106	7.3842	2.6325	7	1.396433531	
O60437;K7EKI8;K7EQ71;RE	49;49;30;1;1;1;1;1;1	Periplakin	PPL	32.7	204.74	1756	6.6082	2.624	35	1.39176772	
P29966	7	Myristoylated alanine-rich C-kinase substrate	MARCKS	44.6	31.554	332	5.4332	2.6221	11	1.390722707	
Q16795;H3BRM9;F5H0J3	8;4;2	NADH dehydrogenase [ubiquinone] 1 alpha subcomplex	NDUFA9	25.7	42.509	377	6.1946	2.6155	7	1.38708677	
Q9Y6C9;E9PIE4;E9PNT8;F5	11;8;7;3	Mitochondrial carrier homolog 2	MTCH2	49.8	33.331	303	6.6785	2.6133	8	1.385872752	
REV__Q9C000;REV__Q9C0	2;2;2;2;2;2			1	165.86	1473	7.1036	2.6118	2	1.385044426	
O15121	2	Sphingolipid delta(4)-desaturase DES1	DEGS1	9.6	37.866	323	7.4374	2.6107	3	1.384436685	
Q9Y3B7;Q9Y3B7-2;A6NLT0;	11;9;7;3;2	39S ribosomal protein L11, mitochondrial	MRPL11	56.2	20.683	192	7.8664	2.6107	13	1.384436685	
B8ZZS0;Q9NYM9;H7BXT7;E	3;3;3;2;1	BET1-like protein	BET1L	18.4	17.107	152	4.6797	2.6106	2	1.384381423	
P50148;B1AM21	9;3	Guanine nucleotide-binding protein G(q) subunit alpha	GNAQ	32.9	42.142	359	5.2819	2.6076	4	1.38272258	
P16615;P16615-3;P16615-2	38;37;37;37;35;32;13	Sarcoplasmic/endoplasmic reticulum calcium ATPase 2	ATP2A2	40.6	114.76	1042	8.4281	2.6063	47	1.382003156	
P48047;H7C0C1;H7C068;H7	12;5;3;3	ATP synthase subunit O, mitochondrial	ATP5O	67.1	23.277	213	8.1621	2.6052	13	1.381394132	
J3KQW8;A8MYQ9;Q9BZG1;	11;11;11;10;10;10;9;8	Ras-related protein Rab-34	RAB34	40.8	35.184	316	4.7274	2.5974	13	1.377068206	
O14828;O14828-2	6;4	Secretory carrier-associated membrane protein 3	SCAMP3	30.8	38.287	347	6.6755	2.5947	6	1.375567743	
P08574	9	Cytochrome c1, heme protein, mitochondrial	CYC1	35.7	35.422	325	5.8822	2.5894	8	1.372617844	
Q13330;E7ESY4;Q13330-3;H	5;5;5;5;2;2;2;1;1;1	Metastasis-associated protein MTA1	MTA1	7.7	80.785	715	4.439	2.5876	3	1.371614618	
Q7Z3C6;Q7Z3C6-2;H7C1G6	4;4;4;2	Autophagy-related protein 9A	ATG9A	5.1	94.446	839	4.6258	2.5831	3	1.369103496	
P05067;P05067-9;P05067-8	7;7;7;7;7;7;6;6;5;5;5;5	Amyloid beta A4 protein;N-APP;Soluble APP-alpha;Solub	APP	10.9	86.942	770	6.7001	2.5817	6	1.368321366	
Q9BR76;E7EW44;F5H390;F5	9;6;6;4;1;1	Coronin-1B	CORO1B	18.2	54.234	489	6.9912	2.5799	6	1.367315146	
P78537;P78537-2;G8JLQ3;F	3;3;3;1;1;1;1	Biogenesis of lysosome-related organelles complex 1 sub	BLOC1S1	20.9	17.262	153	5.2551	2.5757	3	1.364964568	
Q9HC07;B4DHW1;D6RD79;	3;2;1;1	Transmembrane protein 165	TMEM165	17.3	34.905	324	6.2858	2.5728	2	1.363339312	
H7C2I1;Q99873;G5E9B6;Q9	19;19;19;19;19;19;12	Protein arginine N-methyltransferase 1	PRMT1	55.8	42.461	371	6.7787	2.5692	30	1.361319202	
P61009	3	Signal peptidase complex subunit 3	SPCS3	16.7	20.313	180	7.4916	2.5586	3	1.355354621	
Q9NVI7;Q9NVI7-2;H0Y2W	8;8;7;7;4;4;4;3;2;1	ATPase family AAA domain-containing protein 3A;ATPase	ATAD3A;ATAD3B;	12.5	71.368	634	4.2483	2.5522	6	1.351741389	
Q8IXM3	7	39S ribosomal protein L41, mitochondrial	MRPL41	65	15.383	137	6.9184	2.552	11	1.351628329	
P63218	5	Guanine nucleotide-binding protein G(I)/G(S)/G(O) subu	GNG5	79.4	7.3184	68	4.298	2.5492	9	1.350044566	
Q9UHB6-4;Q9UHB6;Q9UHE	7;7;4;4;4;4;4;1;1	LIM domain and actin-binding protein 1	LIMA1	12.8	85.223	760	5.2794	2.5462	2	1.348345745	
P35658-5;P35658-3;P35658	14;14;14;14;14;4;2;2	Nuclear pore complex protein Nup214	NUP214	9	215.4	2093	4.983	2.5396	10	1.344601283	
Q9Y394;H0YJ66;Q9Y394-2;H	18;17;16;9;8;1	Dehydrogenase/reductase SDR family member 7	DHRS7	67.3	38.298	339	6.5846	2.539	27	1.344260395	
Q15125;C9JJ78;C9J719	2;1;1	3-beta-hydroxysteroid-Delta(8),Delta(7)-isomerase	EBP	12.2	26.352	230	8.9906	2.5363	3	1.342725401	
O43395;E7EVD1;B4DSY9	5;4;2	U4/U6 small nuclear ribonucleoprotein Prp3	PRPF3	7.2	77.528	683	5.383	2.5332	5	1.340960985	
Q9NX40;D6RBN5;G8JLN7;C	9;7;7;7;7;7;7;5;5;5;5	OCIA domain-containing protein 1	OCIAD1	38	27.626	245	7.0325	2.5323	8	1.34044833	
Q9P003;A6NLH6	1;1	Protein cornichon homolog 4	CNIH4	14.4	16.093	139	8.2901	2.5315	2	1.339992484	
P62312	7	U6 snRNA-associated Sm-like protein LSm6	LSM6	88.8	9.1275	80	4.5695	2.5314	12	1.339935493	
Q9HCM4;Q9HCM4-3;Q9HC	2;2;2;2;1	Band 4.1-like protein 5;Band 4.1-like protein 4B	EPB41L5;EPB41L4	2.5	81.855	733	3.9922	2.53	3	1.339137385	
P61081	2	NEDD8-conjugating enzyme Ubc12	UBE2M	13.1	20.9	183	4.2756	2.5279	2	1.337939394	
Q9H9P8;C9JVN9;Q9H9P8-2	3;3;3;1;1	L-2-hydroxyglutarate dehydrogenase, mitochondrial	L2HGDH	7.6	50.315	463	4.8725	2.5273	5	1.337596928	
O14656	5	Torsin-1A	TOR1A	17.2	37.808	332	5.8107	2.523	3	1.335140206	
O75976;F5GZH6;J3QQJ4;J3	42;34;7;1	Carboxypeptidase D	CPD	43	152.93	1380	8.2598	2.5226	60	1.334911461	
O95292;O95292-2;E5RK64	10;3;3	Vesicle-associated membrane protein-associated protein	VAPB	39.9	27.228	243	5.8426	2.5211	20	1.334053344	
P78357;K7EMM9	15;7	Contactin-associated protein 1	CNTNAP1	12.9	156.26	1384	4.9946	2.5185	8	1.332564731	
Q9NQC3-2;Q9NQC3;Q9NQ	10;9;9;9;6;6;5;5;5	Reticulon-4	RTN4	47.5	40.317	373	3.9679	2.5096	34	1.327457434	
Q9NZE8;D3YTC1;Q9NZE8-2	2;1;1	39S ribosomal protein L35, mitochondrial	MRPL35	9.6	21.514	188	5.6361	2.5089	2	1.327054969	
Q9NQ50;F8WBK5	4;4	39S ribosomal protein L40, mitochondrial	MRPL40	24.8	24.49	206	5.7074	2.5084	5	1.326767425	
Q969E2;Q969E2-2;Q969E2-	2;2;1;1;1	Secretory carrier-associated membrane protein 4	SCAMP4	10	25.728	229	6.5189	2.5061	2	1.325443983	
P60953;E7ETU3;P60953-1;C	10;8;8;7;1;1;1;1;1;1	Cell division control protein 42 homolog	CDC42	62.8	21.258	191	6.7588	2.5034	10	1.323888827	
Q99470;K7ELI9	4;1	Stromal cell-derived factor 2	SDF2	36	23.026	211	6.4558	2.5026	7	1.323427718	
Q9NX20;E9PI14	8;4	39S ribosomal protein L16, mitochondrial	MRPL16	37.1	28.449	251	6.1243	2.5003	9	1.322101208	
REV__H3BQK9;REV__H3BP	3;3;3;3			0.4	861.07	7592	7.9177	2.4985	2	1.321062218	
J3KN66;Q5JTV8;H0Y4R4;E9	15;14;8;7;6;5;1	Torsin-1A-interacting protein 1	TOR1AIP1	31.1	67.821	599	4.8732	2.4978	15	1.320657964	
O95071;J3KMW7;E7EMW7;	36;36;35;6;3;3;2	E3 ubiquitin-protein ligase UBR5	UBR5	17.5	309.35	2799	5.4521	2.492	23	1.317304068	
Q16740	7	Putative ATP-dependent Clp protease proteolytic subuni	CLPP	34.3	30.18	277	4.2198	2.4914	4	1.316956668	
P27105;B4E2V5;P27105-2	5;5;1	Erythrocyte band 7 integral membrane protein	STOM	24	31.73	288	3.9062	2.4911	3	1.316782937	
E7EQD5;Q15833;Q15833-2	4;4;4	Syntaxin-binding protein 2	STXBP2	9.9	67.694	604	3.7497	2.491	4	1.316725022	
B5ME97;Q9P0V9-2;E7EX04	6;6;6;6;6;5;4;4;1;1;1;	Septin-10	41527	16.9	62.944	544	5.9956	2.489	4	1.31556623	
P20339;B4DJA5	9;8	Ras-related protein Rab-5A	RAB5A	60.9	23.658	215	5.7874	2.484	14	1.312665174	
P15907;H7C472;B2R513;C9	6;3;1;1;1;1	Beta-galactoside alpha-2,6-sialyltransferase 1	ST6GAL1	18.5	46.604	406	6.2966	2.4831	6	1.312142363	
P00846	1	ATP synthase subunit a	MT-ATP6	4.4	24.817	226	8.046	2.4779	3	1.309117966	
Q9UJS0-2;Q9UJS0;F5GX33;	12;12;10;3;3	Calcium-binding mitochondrial carrier protein Aralar2	SLC25A13	22.9	74.303	676	4.0122	2.4772	6	1.308710351	
Q9NYV4;Q9NYV4-2;Q9NYV	5;5;4;4	Cyclin-dependent kinase 12	CDK12	4.1	164.15	1490	5.7179	2.4742	2	1.306962124	
P78345;Q5VUC3;Q5VUC9	1;1;1	Ribonuclease P protein subunit p38	RPP38;RP11-455B	3.9	31.834	283	8.3246	2.4735	2	1.306553899	
Q8NFF5;Q8NFF5-2;Q8NFF5	5;5;5;3;2;1;1	FAD synthase;Molybdenum cofactor biosynthesis protein	FLAD1	10.6	65.265	587	3.3372	2.4706	5	1.304861451	
P83111;P83111-2;H0YNN5	12;7;2	Serine beta-lactamase-like protein LACTB, mitochondrial	LACTB	28.9	60.693	547	7.2316	2.4641	14	1.301060806	
Q14697-2;E9PKU7;E9PNH1	54;44;7;5;2;1		GANAB	70.3	109.44	966	8.2179	2.4627	145	1.300240893	
P98160;Q5SZJ2;Q5SZJ1	53;4;1	Basement membrane-specific heparan sulfate proteogly	HSPG2	15.9	468.83	4391	5.3224	2.4623	38	1.300006547	
P43304;P43304-2;F8W6E4;I	24;23;19;10	Glycerol-3-phosphate dehydrogenase, mitochondrial	GPD2	45.7	80.852	727	6.1934	2.4619	33	1.299772162	
Q969V3;Q969V3-2;K7EMW	8;8;7;4;3;2;1	Nicalin	NCLN	18.7	62.974	563	5.4216	2.4588	7	1.29795439	
P09110;C9JDE9;H7C131;G5	14;12;11;9;6;4;3;2;2	3-ketoacyl-CoA thiolase, peroxisomal	ACAA1	45.5	44.292	424	6.6141	2.4579	21	1.297426221	
B7ZKM8;Q95487;O95487-2	5;5;5	Protein transport protein Sec24B	SEC24B	4.7	140.42	1298	4.5577	2.4489	6	1.292133863	
J3KPM8;K7EN75;P14854;K7	6;6;6;1	Cytochrome c oxidase subunit 6B1	COX6B1	56.3	12.035	103	8.1663	2.4489	7	1.292133863	
O75531	4	Barrier-to-autointegration factor	BANF1	56.2	10.058	89	4.2212	2.444	5	1.289244285	
P37198	4	Nuclear pore glycoprotein p62	NUP62	13.4	53.254	522	4.9654	2.4439	3	1.289185254	
Q5BJF2;J3KTD1;Q86XC5;J3	4;3;2;1	Transmembrane protein 97	TMEM97	39.2	20.848	176	3.8521	2.4428	6	1.28853575	

B5MCA4;P16422;C9JKY3	12;12;3	Epithelial cell adhesion molecule	EPCAM	40.4	37.893	342	5.1566	2.441	29	1.287472295
P12236;I7HJJ0	13;7	ADP/ATP translocase 3	SLC25A6	38.9	32.866	298	5.0805	2.4391	6	1.286348908
O43491;E9PHY5;E9PPD9;E9	37;34;32;30;29;27;27	Band 4.1-like protein 2	EPB41L2;DKFZp78	48	112.59	1005	5.8958	2.4318	47	1.282024581
Q9BTV4	6	Transmembrane protein 43	TMEM43	20.2	44.875	400	5.2684	2.4298	7	1.280837569
Q8IX12;Q8IX12-2;F5H2E6;F	25;25;18;15;11;5;1;1	Cell division cycle and apoptosis regulator protein 1	CCAR1	26.5	132.82	1150	6.9756	2.4294	19	1.280600049
B0YIW6;P48444;E9PEU4;E9	12;12;7;3	Coatomer subunit delta	ARCN1	21.7	61.626	552	5.5975	2.4294	11	1.280600049
Q04656-3;Q04656-2;Q0465	3;3;3;3;1	Copper-transporting ATPase 1	ATP7A	1.9	172.08	1581	4.1333	2.4282	2	1.279887255
H0UI06;D6RGV5;D6RIE3;P1	2;2;2;2;1	Cytochrome c oxidase subunit 7A2, mitochondrial	COX7A2	20	12.844	115	4.0925	2.4273	3	1.279352428
O14497;O14497-3;H0Y488;(	6;6;5;4;3	AT-rich interactive domain-containing protein 1A	ARID1A	3.3	242.04	2285	6.5521	2.4201	4	1.275066662
O15258;Q5T091;Q5T092;Q5	6;5;5;3	Protein RER1	RER1	33.7	22.958	196	5.1851	2.4176	6	1.273575566
P57088;D6RAA6;H0Y8N0;D	12;8;3;1;1	Transmembrane protein 33	TMEM33	44.5	27.978	247	7.7029	2.4159	20	1.272560739
Q9H9J2	12	39S ribosomal protein L44, mitochondrial	MRPL44	39.8	37.535	332	5.7154	2.4142	14	1.271545198
Q8N3X1-2;Q8N3X1	2;2	Formin-binding protein 4	FNBP4	2.1	110.45	1019	4.9384	2.4135	2	1.271126827
Q4G0J3;D6RFF0;D6RAF3;D(	3;3;1;1;1	La-related protein 7	LARP7	6.4	66.898	582	4.6548	2.4071	2	1.267296078
Q9NYU1;E7EMU6;A6NP03;(	14;1;1;1	UDP-glucose:glycoprotein glucosyltransferase 2	UGGT2	10.3	174.73	1516	3.8416	2.4009	7	1.263575315
O60244	2	Mediator of RNA polymerase II transcription subunit 14	MED14	2.3	160.6	1454	3.9889	2.3973	2	1.26141046
Q9HC36;I3L0T6;I3L443	4;3;1	RNA methyltransferase-like protein 1	RNMTL1	14.5	47.019	420	3.5744	2.3959	3	1.260567694
Q5RI15-2;Q5RI15	4;4	Cytochrome c oxidase protein 20 homolog	COX20	38.5	14.669	130	7.6332	2.3897	4	1.256829515
P21796;C9JI87	6;4	Voltage-dependent anion-selective channel protein 1	VDAC1	21.9	30.772	283	6.5844	2.3859	6	1.254533577
Q9Y6N5;H3BNX3;H3BMS6;I	19;11;7;5;4	Sulfide:quinone oxidoreductase, mitochondrial	SQRDL	46.9	49.96	450	6.823	2.3858	15	1.254473108
F6XEV2;Q9BPX6-3;H9KVB8	3;3;3;3;3;3;2	Calcium uptake protein 1, mitochondrial	MICU1	7.1	54.55	478	3.9893	2.3839	3	1.253323719
Q8ND76;Q8ND76-2;Q8ND7	3;3;3;3;2;2;2;2;1;1	Cyclin-Y;Cyclin-Y-like protein 1	CCNY;CCNYL1	14.1	39.336	341	3.2509	2.3832	2	1.252900029
F5H2A9;P49748;P49748-2;(	38;38;37;14;9;7;6;5;4	Very long-chain specific acyl-CoA dehydrogenase, mitocl	ACADVL	66.4	72.926	678	7.4519	2.3817	62	1.251991702
P56199	23	Integrin alpha-1	ITGA1	23.2	130.85	1179	7.4215	2.3788	28	1.250233981
Q9BYD1;E5RJI7;E5RFI2;H0Y	11;8;3;3	39S ribosomal protein L13, mitochondrial	MRPL13	64.6	20.692	178	5.7801	2.3761	14	1.248595554
Q9NVJ2;B4DI85	11;5	ADP-ribosylation factor-like protein 8B	ARL8B	70.4	21.539	186	5.7236	2.3739	14	1.247259163
P49755;G3V2K7	10;4	Transmembrane emp24 domain-containing protein 10	TMED10	44.3	24.976	219	6.4388	2.3737	18	1.247137612
Q6UW63	16	KDEL motif-containing protein 1	KDELC1	39.8	58.042	502	5.2122	2.373	14	1.246712101
Q9NTI5;Q9NTI5-2;Q9NTI5-	8;8;3;1;1	Sister chromatid cohesion protein PDS5 homolog B	PDS5B	8.1	164.67	1447	3.9512	2.3719	3	1.246043187
Q9BX93	3	Group XIIB secretory phospholipase A2-like protein	PLA2G12B	25.1	21.659	195	4.8716	2.3703	2	1.245069667
P50454;E9PPV6;E9PR70;E9	19;14;13;10;10;10;8;6	Serpin H1	SERPINH1	55	46.44	418	7.3511	2.3703	39	1.245069667
O60784-2;O60784;O60784-	6;6;5;5;5;3;2;1;1;1;1;	Target of Myb protein 1	TOM1	20.1	53.874	493	4.8392	2.3695	4	1.244582661
J3KNA0;Q15070;Q15070-2;	4;4;4;3;2;2	Mitochondrial inner membrane protein OXA1L	OXA1L	7.7	55.269	495	2.8584	2.3687	3	1.24409549
Q9BXB4;Q9BXB5;Q9BXB5-2	2;1;1;1	Oxysterol-binding protein-related protein 11;Oxysterol-I	OSBPL11;OSBPL1(	3.1	83.642	747	4.6625	2.3682	2	1.243790925
P63272;J3QSB9;D3DTZ5	2;2;2	Transcription elongation factor SPT4	SUPT4H1	19.7	13.193	117	4.2029	2.3661	3	1.242511049
Q00325-2;Q00325;F8VVM2	14;13;11;4;3;3	Phosphate carrier protein, mitochondrial	SLC25A3	54	39.958	361	6.0251	2.3645	21	1.241535142
Q5HYK3;B4DP72;F8VVX6;C	3;2;2;1;1;1	2-methoxy-6-polyprenyl-1,4-benzoquinol methylase, mi	COQ5	11.9	37.14	327	6.3551	2.3601	3	1.238847989
Q53FV1;F8VXD5;B9A069;F8	4;3;2;2	ORM1-like protein 2	ORMDL2;ORMDL1	35.3	17.363	153	6.8352	2.3595	4	1.238481171
P43121;P43121-2	5;4	Cell surface glycoprotein MUC18	MCAM	9.9	71.607	646	4.8612	2.3556	2	1.236094579
Q8WVM7;Q6P275;Q68DW7	11;11;8;7;1	Cohesin subunit SA-1	STAG1;DKFZp781I	9.9	144.43	1258	3.6582	2.3555	3	1.236033332
O00267;O00267-2	15;15	Transcription elongation factor SPT5	SUPT5H	20.9	121	1087	5.3857	2.3542	16	1.235236889
Q96RQ3;E9PHF7;E9PG35;G	11;11;11;7;7	Methylcrotonoyl-CoA carboxylase subunit alpha, mitoch	MCCC1	21.7	80.472	725	7.045	2.3465	12	1.23051046
J3KQJ1;Q8NBJ7;C9J660;H7(	10;10;9;9;9;8;8;8;8;5;	Sulfatase-modifying factor 2	SUMF2	35.3	35.922	320	6.8767	2.3453	9	1.229772477
P27449;H3BR50	1;1	V-type proton ATPase 16 kDa proteolipid subunit	ATP6V0C	11.6	15.736	155	4.1644	2.3444	2	1.229218742
O15439;O15439-2;O15439-	3;3;3;3	Multidrug resistance-associated protein 4	ABCC4	2.2	149.52	1325	3.845	2.3388	3	1.225768496
Q9Y625;O75487;B4E2C0	8;2;2	Glypican-6;Secreted glypican-6	GPC6	19.1	62.735	555	6.1321	2.3388	11	1.225768496
Q9Y2W1	20	Thyroid hormone receptor-associated protein 3	THRAP3	23.9	108.66	955	6.6149	2.3352	33	1.223546116
Q9UPN9;Q9UPN9-2;H0Y61	6;6;5;2	E3 ubiquitin-protein ligase TRIM33	TRIM33	6	122.53	1127	4.7232	2.3345	7	1.223113589
E7ETU7;H0Y9G6;P09001;E9	12;12;12;7;6;1	39S ribosomal protein L3, mitochondrial	MRPL3	36	41.628	375	5.4362	2.3342	8	1.22292818
Q9BS26	14	Endoplasmic reticulum resident protein 44	ERP44	49.3	46.971	406	6.4058	2.3341	16	1.222866372
Q8TCT9;Q8TCT9-5;Q8TCT9-	6;6;5;5	Minor histocompatibility antigen H13	HM13	27.1	41.488	377	4.6612	2.3341	12	1.222866372
Q96ST3;H3BP90;H3BNZ3	22;1;1	Paired amphipathic helix protein Sin3a	SIN3A	21.4	145.17	1273	5.4048	2.3325	18	1.221877081
Q9ULT8;H0YJD4;G3V4V5;H(	5;2;2;1	E3 ubiquitin-protein ligase HECTD1	HECTD1	2.1	289.38	2610	4.6799	2.3275	3	1.218781168
Q96D05-2;Q96D05;Q5VVH9	3;3;2	Uncharacterized protein C10orf35	C10orf35	23.9	17.512	163	7.0917	2.3247	3	1.21704455
P62487;E9PKH3;H0YEE4;E9	4;1;1;1	DNA-directed RNA polymerase II subunit RPB7	POLR2G	31.4	19.294	172	3.8109	2.3242	4	1.21673422
P50281;F8W1B7	6;3	Matrix metalloproteinase-14	MMP14	10.5	65.893	582	5.6951	2.3237	6	1.216423822
P60468	3	Protein transport protein Sec61 subunit beta	SEC61B	37.5	9.9743	96	7.4363	2.3221	3	1.215430102
Q96A35	6	39S ribosomal protein L24, mitochondrial	MRPL24	32.9	24.915	216	6.5812	2.3209	8	1.214684363
O94776;Q68DB1	11;8	Metastasis-associated protein MTA2	MTA2;DKFZp686F	18.6	75.022	668	5.1789	2.3196	7	1.213876043
O95832	5	Claudin-1	CLDN1	34.6	22.744	211	6.2533	2.316	7	1.211635253
Q13277;Q13277-3;F8W9Y0;	5;5;5;5;3	Syntaxin-3	STX3	16.6	33.155	289	6.4833	2.3117	3	1.208954185
Q8NEN9	18	PDZ domain-containing protein 8	PDZD8	18.2	128.56	1154	5.6238	2.3104	14	1.208142647
Q9BWF3;E9PB51;Q9BQ04;(	14;13;10;9;9;9;9;9;8;8	RNA-binding protein 4;RNA-binding protein 4B	RBM4;RBM4B	50	40.313	364	6.4409	2.307	17	1.206018004
Q9UBL3;Q9UBL3-3;F5H8F7;	4;4;4;3;2;2	Set1/Ash2 histone methyltransferase complex subunit A	ASH2L	10.4	68.722	628	4.3363	2.3054	4	1.205017088
P01019	9	Angiotensinogen;Angiotensin-1;Angiotensin-2;Angioten	AGT	32.4	53.154	485	6.1098	2.3045	10	1.204453768
O15020;O15020-2	23;23	Spectrin beta chain, brain 2	SPTBN2	11.6	271.32	2390	4.8408	2.3041	6	1.204203332
P25705;A8K092;K7ENP3;K7	29;28;26;24;13;11;11	ATP synthase subunit alpha, mitochondrial;ATP synthase	ATP5A1	57	59.75	553	7.064	2.3037	45	1.203952854
Q6P1J9	6	Parafibromin	CDC73	14.9	60.576	531	3.1771	2.3027	8	1.203326466
Q9NVS2-2;Q5QPA5;Q9NVS	7;7;7;4	28S ribosomal protein S18a, mitochondrial	MRPS18A	25.9	30.539	270	5.9964	2.3021	7	1.202950503
P51114;B4DXZ6;P51114-2;F	8;8;8;7;7;7;2;1;1;1;1;	Fragile X mental retardation syndrome-related protein 1	FXR1	12.6	69.72	621	5.0141	2.3003	11	1.201822027
P30049	4	ATP synthase subunit delta, mitochondrial	ATP5D	37.5	17.49	168	7.5187	2.2981	3	1.200441577
P02649;H0Y7L5;E7ERP7;E9F	19;11;10;10	Apolipoprotein E	APOE	65.3	36.154	317	6.5709	2.2971	33	1.199813663
O14735;B3KY94;H3BTV1;H3	4;3;3;2	CDP-diacylglycerol--inositol 3-phosphatidyltransferase	CDIPT	23.9	23.539	213	4.9803	2.296	5	1.199122642
Q13523;F5H2U2;H0YDJ3;H(	17;16;11;7	Serine/threonine-protein kinase PRP4 homolog	PRPF4B	17.8	116.99	1007	5.7888	2.2958	31	1.198996966
Q8NFQ8	11	Torsin-1A-interacting protein 2	TOR1AIP2	32.1	51.263	470	4.2183	2.2922	9	1.196732928
P01009;P01009-2;P01009-3	9;8;5;2;2;2;1	Alpha-1-antitrypsin;Short peptide from AAT	SERPINA1	25.8	46.736	418	5.8639	2.2922	10	1.196732928
Q15427;Q5SZ64	4;2	Splicing factor 3B subunit 4	SF3B4	18.4	44.385	424	5.1938	2.2895	7	1.195032565
J3KNL3;Q9BWS9-2;Q9BWS	12;12;12;10;6;4;4;4;4	Chitinase domain-containing protein 1	CHID1	37.8	48.215	423	5.876	2.2886	12	1.194465332
Q07666;Q07666-2;Q07666-	5;5;4	KH domain-containing, RNA-binding, signal transduction	KHDRBS1	15.1	48.227	443	4.9913	2.2874	10	1.193708673
P24390	5	ER lumen protein retaining receptor 1	KDELR1	34	24.542	212	5.2649	2.2863	6	1.193014721
P49792;E9PGT0;F8W705;Q9	67;45;6;6;2;2;2;2;1;1;	E3 SUMO-protein ligase RanBP2;Putative peptidyl-prolyl	RANBP2	26.8	358.2	3224	4.9997	2.2842	69	1.191688976
P37802;Q9UI15;C9J5W6	9;1;1	Transgelin-2	TAGLN2	67.8	22.391	199	4.0153	2.2831	9	1.190994051
Q9Y5U9	3	Immediate early response 3-interacting protein 1	IER3IP1	64.6	8.9687	82	4.4819	2.2798	8	1.188907267
Q86WB0;Q86WB0-2;C9J0I9	3;3;3;3	Nuclear-interacting partner of ALK	ZC3HC1	8.8	55.261	502	4.6888	2.2762	4	1.186627327
I3L080;Q7Z4V5;K7EQZ6;Q7	8;8;7;7;5;3;2;2;1;1	Hepatoma-derived growth factor-related protein 2	HDGFRP2	15.7	74.815	676	4.8272	2.2747	8	1.185676287
O43615	8	Mitochondrial import inner membrane translocase subur	TIMM44	21.2	51.355	452	3.3923	2.2736	7	1.18497846
Q9UNS1;Q9UNS1-2;E7EWS	5;5;2	Protein timeless homolog	TIMELESS	3.7	138.66	1208	3.5004	2.2712	3	1.183454754
Q9UEU0;Q9UEU0-2;H0YJJ5;	4;2;1;1;1;1	Vesicle transport through interaction with t-SNAREs hom	VTI1B	22.4	26.688	232	3.9773	2.2698	3	1.182565182
Q96A57-2;Q96A57;Q5JWB9	2;2;2	Transmembrane protein 230	TMEM230;C20orf3	13.7	19.91	183	4.1838	2.2654	2	1.179765808
Q96ND0;K7EK00;K7ERQ2;K	3;3;1;1	Protein FAM210A	FAM210A	17.3	30.776	272	5.3717	2.2652	3	1.179638435
Q9NZ01;B3KSQ1;Q9NZ01-2	9;8;2	Trans-2,3-enoyl-CoA reductase	TECR	24.7	36.034	308	5.5868	2.2631	10	1.178300335
Q9NVI1;Q9NVI1-1;Q9NVI1	7;7;7;7;6;2;1;1;1;1	Fanconi anemia group I protein	FANCI	6.6	149.32	1328	3.573	2.2599	7	1.176258935
J9JID7;Q03252	33;33	Lamin-B2	LMNB2	54.8	69.948	620	6.1211	2.2579	42	1.174981592
Q8N5M9	4	Protein jagunal homolog 1	JAGN1	20.2	21.125	183	6.1421	2.2505	5	1.170245565
Q96DV4;B3KN96	6;2	39S ribosomal protein L38, mitochondrial	MRPL38	16.6	44.596	380	4.3819	2.2471	7	1.168064328
Q9UQB3;Q9UQB3-2;E7EPC	5;5;5;5;5;5;1;1	Catenin delta-2	CTNND2	5.1	132.65	1225	3.4968	2.2449	2	1.166651181
Q01082;Q01082-2;F8W6C1;	162;150;43;7;7;7;1	Spectrin beta chain, brain 1	SPTBN1	75	274.61	2364	5.8055	2.2424	340	1.165043649
Q15363;F5GX39;E7EQ72	7;6;4	Transmembrane emp24 domain-containing protein 2	TMED2	42.8	22.761	201	5.9573	2.2389	14	1.162790092
P01031;CON__Q1A7A4	18;3	Complement C5;Complement C5 beta chain;Complemen	C5	14.3	188.3	1676	5.0457	2.2365	16	1.161242758
O95716	8	Ras-related protein Rab-3D	RAB3D	41.6	24.267	219	5.3329	2.2361	11	1.160984708
J3KN00;B4DF76;Q9P0J0;E7	6;6;6;4;4;4	NADH dehydrogenase [ubiquinone] 1 alpha subcomplex	NDUFA13	25.1	25.821	227	4.3138	2.2339	4	1.159564605
O60664;O60664-3;K7EQF4;	7;7;7;5;4;3;3	Perilipin-3	PLIN3	23.7	47.074	434	4.9856	2.2327	4	1.158789414
P06576;F8VPV9;H0YH81;F8	27;25;21;15;9;7;6	ATP synthase subunit beta, mitochondrial;ATP synthase s	ATP5B	72.8	56.559	529	7.0304	2.2325	67	1.158660175
Q9Y3A6;B1AKT4;B1AKT3	7;3;3	Transmembrane emp24 domain-containing protein 5	TMED5	32.3	26.005	229	6.1083	2.2286	9	1.156137698
Q13045;J3QLG3;E7EPM0;Q	6;6;5;5;2;1;1;1;1	Protein flightless-1 homolog	FLII	5.3	144.75	1269	4.8519	2.2283	4	1.155943478
Q13740;B4DTU0;Q13740-2;	23;23;23;23;11;8	CD166 antigen	ALCAM	49.9	65.102	583	6.9175	2.2282	30	1.155878733
O00629;H7C4F6	13;4	Importin subunit alpha-4	KPNA4	44.7	57.886	521	4.5676	2.2251	14	1.153870175
Q9UPU5;B7WPF4	22;22	Ubiquitin carboxyl-terminal hydrolase 24;Ubiquitin carbo	USP24	10.9	294.36	2620	4.5968	2.2236	13	1.152897287
P02671;P02671-2	3;3	Fibrinogen alpha chain;Fibrinopeptide A;Fibrinogen alph	FGA	4.5	94.972	866	4.5432	2.223	2	1.152507948
O96005;F5H8J3;B4DDS3;K7	9;9;8;2;2;1	Cleft lip and palate transmembrane protein 1	CLPTM1	15.2	76.096	669	6.3256	2.2228	9	1.152378145
P04843;B7Z4L4;F8WF32	31;23;5	Dolichyl-diphosphooligosaccharide--protein glycosyltran	RPN1	63.3	68.569	607	6.967	2.2219	56	1.151793887
Q9UH62	4	Armadillo repeat-containing X-linked protein 3	ARMCX3	13.2	42.5	379	4.6092	2.2207	4	1.151014509
Q9BUJ2;Q9BUJ2-2;B7Z4B8;	22;22;22;22;18;2	Heterogeneous nuclear ribonucleoprotein U-like protein	HNRNPUL1	33.1	95.737	856	5.8993	2.2203	23	1.150754622

B4DUT8;Q99439;B4DDF4;Q	2;2;2;1;1;1;1;1	Calponin-2	CNN2	7.6	35.943	330	3.7473	2.2196	2	1.150299708
P84095	10	Rho-related GTP-binding protein RhoG	RHOG	58.1	21.308	191	5.0197	2.2163	9	1.148153179
O14974;O14974-2;O14974-	3;3;3;3;3;3;1;1;1;1	Protein phosphatase 1 regulatory subunit 12A	PPP1R12A	4	115.28	1030	3.4672	2.2138	3	1.146524891
G3V1L9;Q07157;G5E9E7;Q(	25;25;24;24;4;2;2	Tight junction protein ZO-1	TJP1	17.4	197.46	1768	5.065	2.2108	14	1.144568518
P26885;F5H0N4	10;5	Peptidyl-prolyl cis-trans isomerase FKBP2;Peptidyl-proly	FKBP2	61.3	15.649	142	4.0082	2.2106	19	1.144437998
O14949	4	Cytochrome b-c1 complex subunit 8	UQCRQ	36.6	9.9062	82	5.2323	2.2095	4	1.143719931
P83876;K7ES07;O14835;K7I	2;1;1;1;1;1;1	Thioredoxin-like protein 4A	TXNL4A	16.2	16.786	142	4.1853	2.2091	2	1.143458727
P55957-2;P55957;P55957-4	2;2;2	BH3-interacting domain death agonist;BH3-interacting do	BID	10.8	26.836	241	3.1314	2.2084	4	1.143001506
Q9UBF2	5	Coatomer subunit gamma-2	COPG2	9.4	97.621	871	3.4831	2.1996	2	1.137241192
Q5VY93;Q92974;Q92974-2;	4;4;4;4	Rho guanine nucleotide exchange factor 2	ARHGEF2	6.3	111.49	987	4.9004	2.1989	2	1.136781996
Q14562;F5H658	5;4	ATP-dependent RNA helicase DHX8	DHX8	4.9	139.31	1220	4.5381	2.1988	4	1.136716384
P10644;K7ER48;K7EPB2;K7I	2;2;2;2	cAMP-dependent protein kinase type I-alpha regulatory	PRKAR1A	7.6	42.981	381	5.0738	2.1985	2	1.136519533
Q6IAA8;F5H3Y3;F5GX19;F5	6;5;5;4;4	Ragulator complex protein LAMTOR1	LAMTOR1	55.3	17.745	161	7.186	2.1918	8	1.13211616
Q5JWF2;Q5JWF2-2;A6NI00	15;15;15;15;15;15;5;4	Guanine nucleotide-binding protein G(s) subunit alpha is	GNAS	17.6	111.02	1037	5.9622	2.1909	22	1.131523636
E9PF82;Q13557-11;Q13557	3;3;3;3;3;3;3;3;3;3;	Calcium/calmodulin-dependent protein kinase type II su	CAMK2D;CAMK2E	5.1	60.004	533	3.98	2.1872	3	1.129085148
Q9NRW7;F5GYC3;F5H8K1;(	7;5;4;4	Vacuolar protein sorting-associated protein 45	VPS45	13.7	65.076	570	3.9438	2.1869	5	1.128887252
Q9UG56;Q9UG56-2;H0Y7P7	5;5;4;3;3	Phosphatidylserine decarboxylase proenzyme;Phosphat	PISD	11.7	46.671	409	3.9248	2.1857	6	1.128095396
Q05519;Q05519-2;Q5T760;(	3;3;3;1;1;1	Serine/arginine-rich splicing factor 11	SRSF11;SFRS11	8.1	53.542	484	5.6353	2.1807	4	1.124791311
Q92600;B7Z1E5;B5MDQ4;H	6;5;5;2;1	Cell differentiation protein RCD1 homolog	RQCD1	22.7	33.631	299	2.5186	2.178	4	1.123003954
P05455;E9PFH8;E7ERC4;E9I	15;15;10;6;5	Lupus La protein	SSB	40	46.836	408	4.8898	2.1762	20	1.121811151
Q9UPT8	6	Zinc finger CCCH domain-containing protein 4	ZC3H4	6.3	140.26	1303	5.1039	2.1757	4	1.121479642
Q86YH6;B4DKU5;Q86YH6-2	5;5;3	Decaprenyl-diphosphate synthase subunit 2	PDSS2	21.6	44.128	399	2.8127	2.1755	3	1.121347017
P00734;E9PIT3;C9JV37	3;3;1	Prothrombin;Activation peptide fragment 1;Activation pe	F2	7.4	70.036	622	5.7472	2.1751	2	1.12108173
Q96HE7;G3V3E6;G3V5B3;G	24;8;6;4;3;3;3	ERO1-like protein alpha	ERO1L	63.9	54.392	468	6.1276	2.1678	29	1.116231661
P51571;A6NLM8;H7C1C6	5;3;3	Translocon-associated protein subunit delta	SSR4	36.4	18.998	173	5.9533	2.1676	8	1.116098552
Q9Y6E0;Q9Y6E0-2;B4DR80;	3;3;3;3;3;2;2;2;1;1;	Serine/threonine-protein kinase 24;Serine/threonine-pr	STK24;MST4	7.2	49.307	443	2.6443	2.1623	4	1.112566698
F8WCY5;Q14257;A8MXP8;I	7;7;5;3	Reticulocalbin-2	RCN2	32.5	39.139	335	5.394	2.1622	10	1.112499976
O75880;J3QL56	9;8	Protein SCO1 homolog, mitochondrial	SCO1	34.6	33.814	301	4.7787	2.1615	8	1.112032836
P08590;P05976;P05976-2	2;2;2	Myosin light chain 3;Myosin light chain 1/3, skeletal musc	MYL3;MYL1	8.2	21.932	195	3.8394	2.1614	3	1.11196609
Q6JUT2;Q9Y3B3;Q8NBU8;G	7;7;7;1	Transmembrane emp24 domain-containing protein 7	TIRAP3;TMED7;TM	21.3	46.172	404	6.1138	2.1577	10	1.109494291
P04844;P04844-2;Q5JYR7;C	21;21;8;6;4;4;4	Dolichyl-diphosphooligosaccharide--protein glycosyltran	RPN2	53.6	69.283	631	5.9085	2.1564	35	1.108624815
P63096;B4E2V1;C9JPP4;P0	15;14;8;5;2	Guanine nucleotide-binding protein G(i) subunit alpha-1	GNAI1	52	40.361	354	5.4394	2.1548	23	1.10755397
P42345;B1AKP8	21;8	Serine/threonine-protein kinase mTOR	MTOR	12	288.89	2549	4.3007	2.1526	22	1.10608026
Q9BXW7;Q9BXW7-2;A8MY	5;5;2	Cat eye syndrome critical region protein 5	CECR5	17.5	46.321	423	4.3673	2.1521	4	1.105745116
O14910;H0YIA8;H0YI92;J3K	5;5;4;3	Protein lin-7 homolog A	LIN7A	24.9	25.996	233	4.9698	2.1519	4	1.105611037
Q08J23;Q08J23-2;G3V1R4;I	8;8;5;2	tRNA (cytosine(34)-C(5))-methyltransferase	NSUN2	12.3	86.47	767	5.421	2.1513	7	1.105208723
Q2TB90;Q2TB90-2;Q2TB90-	16;13;12	Putative hexokinase HKDC1	HKDC1	18	102.54	917	4.4218	2.1502	8	1.104470858
Q7Z5L9;Q7Z5L9-2;Q9H1B7	8;8;1	Interferon regulatory factor 2-binding protein 2	IRF2BP2	22.7	61.024	587	3.7964	2.1492	8	1.103799743
Q14061;C9J8T6;H7C4E5	4;3;3	Cytochrome c oxidase copper chaperone	COX17	63.5	6.9151	63	5.6323	2.1472	5	1.102456577
P14927;B7Z2R2;E5RJU0;E5F	3;2;2;1	Cytochrome b-c1 complex subunit 7	UQCRB	30.6	13.53	111	5.2116	2.1458	3	1.101515615
Q9NSE4;F6SBX2	34;34	Isoleucine--tRNA ligase, mitochondrial	IARS2	44.2	113.79	1012	5.904	2.1424	23	1.099227866
P62306;F8W0W6	10;6	Small nuclear ribonucleoprotein F	SNRPF	98.8	9.7251	86	3.8595	2.1416	22	1.098689044
Q13232;H3BPR2	5;4	Nucleoside diphosphate kinase 3	NME3	33.1	19.015	169	4.623	2.1415	4	1.098621677
J3KNL6;O15027-5;O15027;(	7;6;6;6;6;6;1	Protein transport protein Sec16A	SEC16A	4.2	251.89	2357	3.7398	2.141	2	1.098284796
Q07065	16	Cytoskeleton-associated protein 4	CKAP4	34.6	66.022	602	4.3711	2.139	8	1.096936483
P82663;B4DFJ5;E7EPW2;B4	12;8;8;6	28S ribosomal protein S25, mitochondrial	MRPS25	67.1	20.116	173	4.5517	2.138	15	1.096261853
Q9BUB7;Q9BUB7-2;D4PHA	3;2;1;1	Transmembrane protein 70, mitochondrial	TMEM70	11.5	28.969	260	6.0853	2.1337	3	1.093357346
P17252;P05771-2;P05771;J3	2;1;1;1	Protein kinase C alpha type;Protein kinase C beta type	PRKCA;PRKCB	3.9	76.749	672	4.3257	2.1292	2	1.090311471
G3V325;P56134-2;C9JJT5;C	4;3;3;3;2;2;1		PTCD1;ATP5J2	6.3	84.109	749	6.1704	2.129	7	1.09017595
P51858;Q5SZ07;Q5SZ08;A8	7;6;6;5	Hepatoma-derived growth factor	HDGF	37.5	26.788	240	4.8326	2.1275	8	1.089159132
O15228;B4DNM9;Q5TBH8;(	14;13;12;6	Dihydroxyacetone phosphate acyltransferase	GNPAT	30.1	77.187	680	4.3268	2.1273	15	1.089023502
Q969H8	6	UPF0556 protein C19orf10	C19orf10	37	18.795	173	5.4783	2.1249	8	1.087394948
Q9P035;H3BS72;H3BPZ1;B4	9;8;7;5;3;2	3-hydroxyacyl-CoA dehydratase 3	PTPLAD1	30.4	43.159	362	5.4876	2.1238	19	1.086647913
Q9C0J8;Q9C0J8-3;C9J8B4	2;1;1	pre-mRNA 3 end processing protein WDR33	WDR33	1.5	145.89	1336	3.9773	2.1218	2	1.085288675
Q13438;Q13438-4;G3XA88;	5;5;4;4;4;4;4;4;4;3	Protein OS-9	OS9	10.8	75.561	667	4.4143	2.1175	4	1.08236197
Q9BXW9;Q9BXW9-2;Q9BX\	7;7;3;2;1	Fanconi anemia group D2 protein	FANCD2	5	166.46	1471	3.8829	2.1114	6	1.07819992
Q9BW27;J3KT10;B4DMQ3;J	3;3;3;3;3;2;2;2;1;1;1;	Nuclear pore complex protein Nup85	NUP85	7	75.019	656	3.3177	2.1104	3	1.07751647
Q9P0L0;Q9P0L0-2;J3QKM9	8;7;1	Vesicle-associated membrane protein-associated protei	VAPA	36.1	27.893	249	4.9438	2.109	9	1.076559095
Q9BRJ6;C9JQV0;H7C2R9;H	4;4;3;3	Uncharacterized protein C7orf50	C7orf50	35.6	22.083	194	3.775	2.1086	4	1.076285443
REV__J3KNF5;REV__O1507	2;2;1			0.6	290.54	2481	3.9825	2.1063	2	1.074710934
Q8IZL8-2;C9JFV4;I3L3A8;Q	3;3;3;3;3;2;2;1;1;1;1	Proline-, glutamic acid- and leucine-rich protein 1	PELP1	2.4	135.49	1275	3.7134	2.1061	2	1.074573939
Q7Z7F7-2;Q7Z7F7	4;4	39S ribosomal protein L55, mitochondrial	MRPL55	29.3	18.902	164	6.0838	2.1055	6	1.074162876
Q8NET6	4	Carbohydrate sulfotransferase 13	CHST13	10.9	38.919	341	3.037	2.1047	4	1.073614609
Q4ZIN3;Q4ZIN3-2	3;3	Membralin	C19orf6	7.3	67.888	620	3.0002	2.1045	3	1.07347751
P04004	3	Vitronectin;Vitronectin V65 subunit;Vitronectin V10 subu	VTN	7.3	54.305	478	4.1799	2.103	2	1.07244885
Q68E01;Q68E01-2;Q68E01-	9;9;6;3	Integrator complex subunit 3	INTS3	11.6	118.07	1043	4.8399	2.0955	3	1.067294521
Q9Y3E0;F5H6U7;G3V1U5;F	3;3;2;1;1	Vesicle transport protein GOT1B	GOLT1B	17.4	15.425	138	3.392	2.0938	7	1.066123642
O76031;H0YM48	12;3	ATP-dependent Clp protease ATP-binding subunit clpX-li	CLPX	22.9	69.223	633	5.1246	2.0928	10	1.065434446
Q9NTX5;Q9NTX5-2;Q9NTX	10;10;10;8;6;4;4;4;4;	Ethylmalonyl-CoA decarboxylase	ECHDC1	44.3	33.698	307	4.2184	2.0926	6	1.065296567
E9PCT1;A9Z1X7;Q8IYB3;Q8	7;7;7;7;2	Serine/arginine repetitive matrix protein 1	SRRM1	11.4	103.79	916	5.252	2.0926	12	1.065296567
O75533;H7C341;B4DGZ4;E	62;9;7;7;1	Splicing factor 3B subunit 1	SF3B1	64	145.83	1304	6.966	2.0925	116	1.065227623
Q9Y5U8;H7BXW7	3;2	Brain protein 44-like protein	BRP44L	50.5	12.347	109	6.544	2.0895	3	1.063157759
Q9Y3L5;F6U784	6;4	Ras-related protein Rap-2c	RAP2C;RAP2A	44.3	20.745	183	4.0889	2.0877	6	1.061914413
O00217;E9PPW7;E9PKH6;F	5;5;5;5;5;2	NADH dehydrogenase [ubiquinone] iron-sulfur protein 8	NDUFS8	23.8	23.705	210	4.3845	2.0869	4	1.061361471
Q92643;A6NEM5;B4E2M3;E	2;2;2;2	GPI-anchor transamidase	PIGK	5.6	45.251	395	4.5873	2.0869	2	1.061361471
O43505	2	N-acetyllactosaminide beta-1,3-N-acetylglucosaminyltra	B3GNT1	10.6	47.119	415	4.0453	2.0862	2	1.060877473
Q9C0C2;Q9C0C2-2;E9PKK0	3;1;1	182 kDa tankyrase-1-binding protein	TNKS1BP1	2.3	181.79	1729	3.5862	2.0849	2	1.059978188
Q9BYD6;H0Y8N7;H0YAB5	8;5;3	39S ribosomal protein L1, mitochondrial	MRPL1	33.2	36.908	325	5.979	2.0825	6	1.058316496
C9IZQ1;E9PAL7;P43307;C9J	5;5;5;5;5;4;4;1;1	Translocon-associated protein subunit alpha	SSR1	20.8	33.887	298	5.7691	2.0792	5	1.056028539
P50402;Q5HY57;F8WEQ1	2;2;1	Emerin	EMD	11.4	28.994	254	3.7138	2.0791	2	1.05595915
K7ELL7;P14314;P14314-2;K	22;22;22;10;3;3;1	Glucosidase 2 subunit beta	PRKCSH	37.9	60.192	535	7.0796	2.078	46	1.055195654
O60828;O60828-2;O60828-	2;2;2;2;1;1;1;1;1;1;	Polyglutamine-binding protein 1	PQBP1	6.8	30.472	265	4.4986	2.0779	2	1.055126225
Q0VGL1;C9JXA7	2;1	UPF0539 protein C7orf59	C7orf59	28.3	10.741	99	6.7272	2.0698	3	1.04949137
J3QLS3;Q9Y2R9;J3QKW2;J3	12;12;9;9;8	28S ribosomal protein S7, mitochondrial	MRPS7	46.9	31.704	271	4.5631	2.0655	16	1.04649106
P39656;E7EWT1	15;13	Dolichyl-diphosphooligosaccharide--protein glycosyltran	DDOST	50.4	50.8	456	5.9335	2.0652	26	1.046281503
Q13243;Q13243-3;B4DUA4;	6;5;3;3;2;2	Serine/arginine-rich splicing factor 5	SRSF5	25	31.263	272	5.7057	2.0648	5	1.046002047
O00541;O00541-2;B5MCF9;	5;5;5;5;2	Pescadillo homolog	PES1	8	68.002	588	2.8409	2.0626	5	1.044464067
Q12872-2;Q12872;F5H5X1	2;2;2	Splicing factor, suppressor of white-apricot homolog	SFSWAP	2.1	110.01	1003	4.1311	2.0618	2	1.043904394
O95373;E9PLB2	26;3	Importin-7	IPO7	39.4	119.52	1038	5.6278	2.0595	29	1.042294126
Q8N2K0-2;Q8N2K0;Q8N2K	3;3;3;2;1;1;1	Monoacylglycerol lipase ABHD12	ABHD12	8.7	45.558	404	3.7645	2.0589	4	1.041873761
O00161;O00161-2;H3BPJ0;I	9;9;7;7;7;7;3;3;3;3;3;	Synaptosomal-associated protein 23;Synaptosomal-asso	SNAP23	50.7	23.354	211	5.1441	2.0588	8	1.041803688
P42858;H0YA07	43;2	Huntingtin	HTT	18	347.6	3142	4.4298	2.056	37	1.039840265
Q13795;Q13795-2;Q9BQK5	4;4;1	ADP-ribosylation factor-related protein 1	ARFRP1	25.9	22.614	201	3.2905	2.0549	4	1.039068188
E9PFA8;H0Y5K5;H7BXI5;Q9	2;2;2;2;2;2;1;1;1	Endoplasmic reticulum-Golgi intermediate compartment	ERGIC3	4.8	44.828	398	5.6503	2.0516	2	1.036749476
O15400;O15400-2	8;8	Syntaxin-7	STX7	44.8	29.815	261	6.0339	2.0513	8	1.0365385
Q9NP92	6	28S ribosomal protein S30, mitochondrial	MRPS30	18	50.364	439	5.8798	2.0507	6	1.036116453
Q14376;Q5QPP4;Q5QPP3;(	5;5;5;4;4;1	UDP-glucose 4-epimerase	GALE	15.2	38.281	348	2.848	2.0475	4	1.033863452
P52701;B4DF41;P52701-2;F	19;17;17;16;2;2;2;2;1	DNA mismatch repair protein Msh6	MSH6	16.6	152.78	1360	5.3954	2.047	13	1.033511102
P02765;C9JV77	4;2	Alpha-2-HS-glycoprotein;Alpha-2-HS-glycoprotein chain	AHSG	8.2	39.324	367	5.0461	2.0458	2	1.032665112
P35611-3;P35611;E7ENY0;A	13;13;13;13;13;13;12	Alpha-adducin	ADD1	27.1	84.302	768	4.9482	2.0438	9	1.031254025
Q13637;P57729;H0YEA4	14;2;1	Ras-related protein Rab-32	RAB32	69.8	24.997	225	4.8663	2.0419	25	1.029912213
Q9BQA9;E1B6X3;J3KSJ5;J3(	5;5;4;4;3;3;3;3;2;1;1;	Uncharacterized protein C17orf62	C17orf62	29.9	20.774	187	6.5251	2.0404	3	1.028852006
Q13813-3	187			75	282.28	2452	4.8234	2.04	2	1.028569152
Q8NC51;Q8NC51-3;Q8NC5	9;9;8;8	Plasminogen activator inhibitor 1 RNA-binding protein	SERBP1	28.2	44.965	408	3.7971	2.0398	10	1.028427705
Q4G148;Q4G148-2	4;4	Glucoside xylosyltransferase 1	GXYLT1	10.5	50.566	440	4.2108	2.0393	3	1.028074025
Q8N766;Q8N766-2;Q8N76	5;5;4;4;3;1	Uncharacterized protein KIAA0090	KIAA0090	6.2	111.76	993	2.8706	2.0374	3	1.02672925
Q9H2W6	9	39S ribosomal protein L46, mitochondrial	MRPL46	44.4	31.705	279	5.1097	2.0361	14	1.025808419
Q9BZE1;A6NHR2;H0Y4J2	15;5;3	39S ribosomal protein L37, mitochondrial	MRPL37	45.9	48.117	423	4.5009	2.036	12	1.025737561
Q13310-3;Q13310;Q13310-	14;14;14;14;11;5;4;4;	Polyadenylate-binding protein 4	PABPC4	26.2	72.39	660	5.184	2.0347	6	1.024816096
O95479	20	GDH/6PGL endoplasmic bifunctional protein;Glucose 1-d	H6PD	30.6	88.892	791	5.7694	2.0328	19	1.023468281
P49406;H7C2J0	10;3	39S ribosomal protein L19, mitochondrial	MRPL19	38.4	33.535	292	5.5771	2.0327	9	1.023397308
Q7Z4H8;Q7Z4H8-2;Q7Z4H8	7;5;5;2	KDEL motif-containing protein 2	KDELC2	17	58.572	507	3.9944	2.0315	6	1.022545365
Q9Y4A5;F2Z2U4;Q9Y4A5-2;	19;19;19;19	Transformation/transcription domain-associated protein	TRRAP	5.5	437.6	3859	3.3164	2.031	15	1.02219024
Q92922;F8WE13	14;4	SWI/SNF complex subunit SMARCC1	SMARCC1	16.6	122.87	1105	4.9558	2.029	7	1.020768865
Q8N490-2	3			30.3	15.396	142	6.4763	2.0222	3	1.01592569
Q9UIG0;Q9UIG0-2	2;2	Tyrosine-protein kinase BAZ1B	BAZ1B	1.5	170.9	1483	3.3049	2.0212	2	1.015212085
O00483	6	NADH dehydrogenase [ubiquinone] 1 alpha subcomplex	NDUFA4	77.8	9.3697	81	4.3554	2.0206	10	1.014783753
O75306;J3KPM7;B7Z9L2	8;8;5	NADH dehydrogenase [ubiquinone] iron-sulfur protein 2	NDUFS2	18.4	52.545	463	5.0715	2.0174	8	1.012497163
P18754-2;P18754;C9JW69;(	5;5;5;4;4;4;4;4	Regulator of chromosome condensation	RCC1	12.8	48.145	452	5.4853	2.013	3	1.009347172
Q8N5M1;E9PMH4;C9J2Q2;I	9;3;3;1	ATP synthase mitochondrial F1 complex assembly factor	ATPAF2	43.6	32.772	289	3.1566	2.0121	3	1.008702008
Q13283;F5H4D6;E5RIZ6;E5F	14;7;6;5;5;5;5;5;2	Ras GTPase-activating protein-binding protein 1	G3BP1	38.4	52.164	466	5.5804	2.0098	18	1.007051943
F8VXC8;Q8TAQ2;Q8TAQ2-:	16;16;16;16;4	SWI/SNF complex subunit SMARCC2	SMARCC2	15.2	136.18	1245	4.7792	2.0087	12	1.006262113
E9PFU1;Q8WUM4;C9IZF9;F	8;8;2;1	Programmed cell death 6-interacting protein	PDCD6IP	11	96.771	873	3.2268	2.0052	3	1.003746139
Q9H936;E9PJH7;Q9H1K4;E	4;3;2;2;1;1;1;1;1;1;	Mitochondrial glutamate carrier 1;Mitochondrial glutama	SLC25A22;SLC25A	17.3	34.47	323	3.8968	2.0017	4	1.00122577
Q5JRA6;Q5JRA6-2;G8JLC6;I	33;33;32;20;10;9	Melanoma inhibitory activity protein 3	MIA3	21.8	213.7	1907	4.4657	1.9998	36	0.999855723
Q14847-2;Q14847;C9J9W2;	6;6;5;4;2;1;1	LIM and SH3 domain protein 1	LASP1	19.2	36.014	323	2.657	1.9995	3	0.999639281
P33993;P33993-3;P33993-2	29;22;12;6	DNA replication licensing factor MCM7	MCM7	53.3	81.307	719	5.9983	1.9992	35	0.999422807
Q7L5D6;Q7L5D6-2;C9JHN8;	5;4;2;2;1	Golgi to ER traffic protein 4 homolog	GET4	20.8	36.504	327	3.9616	1.9953	8	0.996605677
Q9BUR5;Q9BUR5-2;C9J574;	3;3;3;3;2	Apolipoprotein O	APOO	21.2	22.284	198	5.2548	1.9922	3	0.994362489

O43819;C9JBU1	6;3	Protein SCO2 homolog, mitochondrial	SCO2	35	29.81	266	5.6477	1.9905	8	0.993130871
Q9UBE0;B3KNJ4;G3XAK6;F	8;4;4;4;4	SUMO-activating enzyme subunit 1	SAE1	26.9	38.449	346	3.5488	1.9896	7	0.992478413
Q01081;Q01081-2;Q8WU68	10;9;5;5;5;4;4;2;2;2;1	Splicing factor U2AF 35 kDa subunit;Splicing factor U2AF 2	U2AF1;U2AF1L4	45	27.872	240	5.5915	1.9863	12	0.990083536
Q7L014;D6RJA6;H0Y9U3	29;12;2	Probable ATP-dependent RNA helicase DDX46	DDX46	30.7	117.36	1031	5.4889	1.9821	39	0.987029751
O60841	11	Eukaryotic translation initiation factor 5B	EIF5B	13.2	138.83	1220	4.1547	1.982	11	0.986956963
P38646;D6RJI2;H0YBG6;H0Y	32;5;4;4;3	Stress-70 protein, mitochondrial	HSPA9	51.7	73.68	679	6.5967	1.98	61	0.98550043
P07919	4	Cytochrome b-c1 complex subunit 6, mitochondrial	UQCRH	38.5	10.739	91	6.4704	1.9783	3	0.984261221
Q8TD57;Q8TD57-3	3;1	Dynein heavy chain 3, axonemal	DNAH3	0.6	470.77	4116	5.1533	1.9772	2	0.983458812
Q9BWH2;H7C0K8	7;2	FUN14 domain-containing protein 2	FUNDC2	36	20.675	189	3.4325	1.9758	5	0.982436918
G3XAN8;Q9Y5J9;E9PIR3	4;4;1	Mitochondrial import inner membrane translocase subui	TIMM8B	42.9	11.155	98	5.3745	1.9704	7	0.978488533
Q9BTU6;E9PAM4	2;2	Phosphatidylinositol 4-kinase type 2-alpha	PI4K2A	3.5	54.022	479	3.2436	1.9686	2	0.97717
O75197	2	Low-density lipoprotein receptor-related protein 5	LRP5	1.7	179.14	1615	3.0824	1.9671	2	0.976070301
P30084	14	Enoyl-CoA hydratase, mitochondrial	ECHS1	52.8	31.387	290	5.0853	1.9668	22	0.97585026
Q15542;Q15542-2	2;2	Transcription initiation factor TFIID subunit 5	TAF5	3.4	86.829	800	2.8955	1.9658	2	0.97511655
Q9H0P0;Q9H0P0-1;Q9H0P(	2;2;2;2;1;1	Cytosolic 5-nucleotidase 3	NT5C3	7.1	37.948	336	3.4522	1.9657	2	0.975043158
P11171;P11171-2;P11171-5	7;7;7;6;6;6;5;5	Protein 4.1	EPB41	9	97.016	864	4.3906	1.9648	6	0.974382466
P45877	3	Peptidyl-prolyl cis-trans isomerase C	PPIC	14.6	22.763	212	4.9862	1.9629	3	0.972986677
Q8WXH0-2;Q8WXH0;G3V5	20;20;17;7;3;3;3;2;2;1	Nesprin-2	SYNE2	3	798.85	6907	4.2299	1.9619	9	0.972251508
P05141	16	ADP/ATP translocase 2	SLC25A5	52.7	32.852	298	4.9565	1.9605	16	0.971221642
P78310;B7WPI3;P78310-2;[	7;6;6;6;4;3;2	Coxsackievirus and adenovirus receptor	CXADR	25.2	40.029	365	4.4813	1.9601	7	0.970927259
Q13185;B8ZZ43;C9JMM0	8;2;2	Chromobox protein homolog 3	CBX3	52.5	20.811	183	4.7336	1.959	18	0.970117398
O94905;E5RHW4;O94905-3	10;10;5;5;3	Erlin-2	ERLIN2	43.4	37.839	339	4.2867	1.9578	11	0.969233393
Q9Y376	3	Calcium-binding protein 39	CAB39	9.1	39.869	341	2.9785	1.9509	2	0.96413983
Q7L0Y3;C9JVB6	15;11	Mitochondrial ribonuclease P protein 1	RG9MTD1	45.2	47.346	403	4.8979	1.9485	13	0.962363932
P30459;P10314;A2BDY9;F8'	14;13;13;10;4	HLA class I histocompatibility antigen, A-74 alpha chain;H	HLA-A	47.1	40.89	365	4.9533	1.9484	2	0.962289888
P61006;B4DEK7	11;8	Ras-related protein Rab-8A	RAB8A	47.3	23.668	207	4.0462	1.9477	18	0.961771479
P10301	7	Ras-related protein R-Ras	RRAS	46.8	23.48	218	5.3564	1.9477	5	0.961771479
Q29RF7;Q29RF7-3;H0Y9L9;	16;7;2;1;1	Sister chromatid cohesion protein PDS5 homolog A	PDS5A	17.1	150.83	1337	4.584	1.9446	15	0.959473426
Q14444;Q14444-2;G3V153;	11;11;9;7;5	Caprin-1	CAPRIN1	17.5	78.365	709	6.6225	1.9439	14	0.958954004
Q9Y2X3;H7BZ72	5;3	Nucleolar protein 58	NOP58	16.1	59.578	529	3.5631	1.9425	6	0.957914599
Q16540;A8MYK1;A8MVT4;/	7;6;6;6;5	39S ribosomal protein L23, mitochondrial	MRPL23	40.5	17.781	153	6.5144	1.9407	7	0.956577119
Q15691;Q9UPY8;Q9UPY8-2	6;1;1;1	Microtubule-associated protein RP/EB family member 1	MAPRE1	26.9	29.999	268	4.2646	1.9389	7	0.955238397
Q6IAN0;J3KRS1;J3QKT1;J3(	8;5;2;2;1	Dehydrogenase/reductase SDR family member 7B	DHRS7B	33.2	35.119	325	4.0459	1.9369	9	0.953749471
Q04917;A2IDB2;A2IDB1;F8'	13;8;1;1	14-3-3 protein eta	YWHAH	53.3	28.218	246	4.901	1.9367	13	0.953600494
P10155;Q5LJ99;Q5LJA0;Q5I	5;5;5;5;3;2	60 kDa SS-A/Ro ribonucleoprotein	TROVE2	10	60.67	538	3.8641	1.9361	5	0.95315347
A6NCI0;Q9Y6G3;J3KPP0	2;2;2	39S ribosomal protein L42, mitochondrial	MRPL42	21.8	16.602	142	3.7339	1.9314	6	0.949646983
Q8WXA9-2;E5RFV3	2;1		SREK1	3.8	71.649	624	3.0266	1.9306	3	0.949049284
F8VTL3;P35580-3;F8W6L6;I	98;98;98;98;98;8;2;1	Myosin-10	MYH10	56	232.53	2007	5.6724	1.9284	139	0.947404335
Q9UKV3-5;Q9UKV3;E7EQT4	22;21;21;9;8;8;3	Apoptotic chromatin condensation inducer in the nucleu	ACIN1	20.5	150.56	1328	4.6131	1.9267	12	0.946131952
Q9UN86;Q9UN86-2;D6RB1	8;8;5;5;4;3;3;3;3;3;2;1	Ras GTPase-activating protein-binding protein 2	G3BP2	19.7	54.12	482	5.0934	1.9255	5	0.945233123
Q13263;Q13263-2	29;28	Transcription intermediary factor 1-beta	TRIM28	54.1	88.549	835	6.9816	1.9243	63	0.944333734
Q9P015;E5RHF4;E5RIZ4	11;5;5	39S ribosomal protein L15, mitochondrial	MRPL15	41.2	33.419	296	5.0011	1.9239	13	0.944033813
Q8N442	2	Translation factor GUF1, mitochondrial	GUF1	4.8	74.327	669	2.7793	1.9234	2	0.943658824
Q15291;Q15291-2	1;1	Retinoblastoma-binding protein 5	RBBP5	1.7	59.152	538	3.4228	1.9215	2	0.942232976
O60216;Q9H4I0;Q9H4I0-2;I	3;1;1;1;1;1;1;1;1	Double-strand-break repair protein rad21 homolog	RAD21	5.4	71.689	631	4.3689	1.9212	3	0.942007714
O75844	10	CAAX prenyl protease 1 homolog	ZMPSTE24	26.3	54.812	475	4.9583	1.9208	9	0.941707309
Q9HBR0;Q9HBR0-3	2;1	Putative sodium-coupled neutral amino acid transporter	SLC38A10	2.6	119.76	1119	3.7651	1.9205	2	0.941481964
P09669	5	Cytochrome c oxidase subunit 6C	COX6C	57.3	8.7813	75	5.6151	1.9203	3	0.941331714
E9PDR0;P05166;E7EX59;C9	21;21;20;20;20;20;19	Propionyl-CoA carboxylase beta chain, mitochondrial	PCCB	57.6	59.403	550	5.4185	1.9189	19	0.94027953
Q86TS9;G3XCN9;G5E9P5	2;2;2	39S ribosomal protein L52, mitochondrial	MRPL52	26.8	13.664	123	4.3988	1.9183	2	0.939828359
Q8NBN7;G8JLA1;Q8NBN7-	10;7;5;4;1;1;1	Retinol dehydrogenase 13	RDH13	39.3	35.932	331	4.8659	1.9138	11	0.93644007
Q9BSH5	11	Haloacid dehalogenase-like hydrolase domain-containin	HDHD3	62.5	28	251	5.6654	1.9114	8	0.934629724
Q8TEM1;Q8TEM1-2	30;15	Nuclear pore membrane glycoprotein 210	NUP210	22	205.11	1887	5.826	1.9106	28	0.93402577
B7Z596;H7BYY1;F5H7S3;H0	13;13;13;11;11;10;10;8;8;8;4;4;2		TPM1	35.3	31.753	275	5.6651	1.9075	6	0.931683057
B3KTC7;P49959;F8W7U8;P4	3;3;3;3;2;1;1	Double-strand break repair protein MRE11A	MRE11A	5.2	81.069	711	3.8845	1.9044	3	0.929336534
Q5T160;H0Y450	4;1	Probable arginine--tRNA ligase, mitochondrial	RARS2	7.1	65.505	578	2.6739	1.8985	4	0.924859999
Q9NZU0	3	Leucine-rich repeat transmembrane protein FLRT3	FLRT3	5.4	73.003	649	3.7377	1.8969	3	0.923643625
O95721;C9JAF7	5;3	Synaptosomal-associated protein 29;Synaptosomal-asso	SNAP29	22.1	28.97	258	4.9345	1.8967	5	0.923491507
Q8IZ81;D6RBS5;D6RHX2	12;10;6	ELMO domain-containing protein 2	ELMOD2	46.4	34.96	293	5.187	1.8931	12	0.920750621
Q9BRJ2;J3KSS2;J3QSH3;J3K	7;1;1;1	39S ribosomal protein L45, mitochondrial	MRPL45	23.9	35.351	306	5.331	1.8897	6	0.918157217
Q9Y679;Q9Y679-2;Q9Y679-	5;5;5	Ancient ubiquitous protein 1	AUP1	12.6	53.028	476	4.1716	1.8871	5	0.916170875
P49736;H0Y8E6;F8WDM3;C	32;31;1;1;1	DNA replication licensing factor MCM2	MCM2	41.7	101.89	904	5.8888	1.8837	30	0.913569218
Q8N684-3;Q8N684;Q8N684	2;2;2;2;1;1;1;1;1;1;1	Cleavage and polyadenylation specificity factor subunit 7	CPSF7	7	56.374	514	2.8612	1.8812	2	0.911653237
E9PAU2;Q8IY67-2;Q8IY67;K	4;4;2;1	Ribonucleoprotein PTB-binding 1	RAVER1	8.7	79.578	756	4.9073	1.8806	5	0.911193023
P27797;K7EJB9;K7EL50	19;13;5	Calreticulin	CALR	63.5	48.141	417	5.0909	1.8788	30	0.909811499
Q12769;G3V198;E9PR16;Q1	21;19;13;3;3;2;1	Nuclear pore complex protein Nup160	NUP160	18.7	162.12	1436	5.2062	1.8784	19	0.909504314
P26368;P26368-2;K7ENG2;I	12;12;10;1;1	Splicing factor U2AF 65 kDa subunit	U2AF2	37.3	53.5	475	4.8832	1.8774	20	0.908736064
P08236;P08236-2;E9PCV0;F	8;8;7;2;2;1	Beta-glucuronidase	GUSB	13.7	74.731	651	5.773	1.8751	7	0.906967537
Q96EL3;B9A051	6;1	39S ribosomal protein L53, mitochondrial	MRPL53	72.3	12.107	112	6.0302	1.8748	8	0.9067367
P04179;B3KUK2;P04179-2;F	13;13;11;10;10;10;7;7	Superoxide dismutase [Mn], mitochondrial;Superoxide d	SOD2	71.6	24.722	222	5.5836	1.8746	15	0.906582788
Q6UW78	2	UPF0723 protein C11orf83	C11orf83	32.3	10.081	93	5.2326	1.8734	3	0.90565897
Q8N5N7;B7Z358	8;5	39S ribosomal protein L50, mitochondrial	MRPL50	66.5	18.325	158	5.9565	1.8733	13	0.905581959
Q14165;F5H1S8;F5GX14;H0	5;4;2;2	Malectin	MLEC	22.6	32.233	292	2.8417	1.873	5	0.9053509
P46977;E9PNQ1;E9PI32;E9I	8;5;3;3;3	Dolichyl-diphosphooligosaccharide--protein glycosyltran	STT3A	14.3	80.529	705	5.2684	1.8709	11	0.903732448
Q96PU8;Q96PU8-3;Q96PU8	7;7;7;7;7;7;5;5;3;3;2;1	Protein quaking	QKI	26.1	37.67	341	4.6049	1.8685	5	0.901880564
Q15637-5;Q15637;Q15637-	9;9;9;9;9;9;8;5;2;2;1	Splicing factor 1	SF1	17.4	80.62	764	5.9939	1.868	13	0.901494455
Q15746;Q15746-6;Q15746-	2;2;2;2;2;2;1;1	Myosin light chain kinase, smooth muscle;Myosin light cl	MYLK	0.8	210.71	1914	2.9339	1.8669	2	0.900644652
E9PHG5;Q6UW02;C9J8K7;F	6;6;4;4	Cytochrome P450 20A1	CYP20A1	14.9	53.448	470	3.7875	1.8668	4	0.900567372
Q01844-5;Q01844;Q01844-	7;7;7;7;7;6;6;5;1;1	RNA-binding protein EWS	EWSR1	15.4	68.965	661	5.8021	1.8649	9	0.899098272
O00159;O00159-3;F5H6E2;(	18;18;18;18;5;5;5;5;4	Unconventional myosin-Ic	MYO1C	21.8	121.68	1063	3.0424	1.8649	13	0.899098272
Q8TAE8	8	Growth arrest and DNA damage-inducible proteins-inter	GADD45GIP1	52.3	25.384	222	5.3553	1.8647	7	0.898943543
Q06136;B4DMX0;K7EQS7;K	6;4;4;3	3-ketodihydrosphingosine reductase	KDSR	21.1	36.187	332	4.6142	1.8629	6	0.897550233
Q7Z2W9;F5H7V8;C9JPR2;H	6;4;4;3	39S ribosomal protein L21, mitochondrial	MRPL21	35.1	22.814	205	5.2434	1.8628	7	0.897472787
O75489;E9PS48;B4DFM8;G	8;2;2;1;1	NADH dehydrogenase [ubiquinone] iron-sulfur protein 3	NDUFS3	41.7	30.241	264	5.0263	1.8624	14	0.897162963
P82094-2;P82094;F8WF45	5;5;3	TATA element modulatory factor	TMF1	4.6	123.15	1096	3.0199	1.8601	5	0.895380183
Q9NRP0;D6RH22	4;3	Oligosaccharyltransferase complex subunit OSTC	OSTC	24.8	16.829	149	4.0931	1.8594	5	0.894837161
D3DQV9;P78344;H0Y3P2;P	5;5;5;5;3;1;1;1;1	Eukaryotic translation initiation factor 4 gamma 2	EIF4G2	5.2	102.33	907	2.5747	1.8592	2	0.894681974
P78527;P78527-2;E7EUY0;F	241;237;236;7;4	DNA-dependent protein kinase catalytic subunit	PRKDC	63.7	469.08	4128	4.4747	1.8579	369	0.893672852
Q99436;Q5TBG5;B4E0P1	10;4;3	Proteasome subunit beta type-7	PSMB7	49.1	29.965	277	4.0564	1.8579	15	0.893672852
P10620;G5EA53;F5H6X2;F5	7;4;4;4;3;2;2	Microsomal glutathione S-transferase 1	MGST1	76.1	17.598	155	5.5007	1.8554	19	0.891730247
Q9HCK8;Q9HCK8-2;Q9P2D	5;4;3;3;3;1;1;1	Chromodomain-helicase-DNA-binding protein 8;Chromo	CHD8;CHD7;CHD6	1.6	290.52	2581	5.9182	1.8548	5	0.891263632
Q709C8;Q709C8-3;Q709C8-	15;15;15;15	Vacuolar protein sorting-associated protein 13C	VPS13C	4.9	422.39	3753	3.5664	1.8544	8	0.890952471
Q9UK45	3	U6 snRNA-associated Sm-like protein LSm7	LSM7	32	11.602	103	4.5254	1.8541	5	0.890719057
Q13405;H0YDP7;E9PNF1;E9	7;6;2;1	39S ribosomal protein L49, mitochondrial	MRPL49	41	19.198	166	5.5359	1.8532	8	0.890018587
Q7Z6Z7;Q7Z6Z7-3;Q7Z6Z7-	96;96;96;73;41;23;4;2	E3 ubiquitin-protein ligase HUWE1	HUWE1	28.7	481.89	4374	4.2545	1.8523	90	0.889317778
Q9UBV8	3	Peflin	PEF1	11.6	30.381	284	3.2705	1.8522	3	0.889239889
Q86Z14	8	Beta-klotho	KLB	9.6	119.81	1044	4.0285	1.8503	9	0.887759202
Q16181;E7EPK1;E7ES33;Q1	17;17;17;17;16;16;15	Septin-7	41524	47.6	50.679	437	5.2825	1.8492	19	0.886901268
P01024;O95568	102;1	Complement C3;Complement C3 beta chain;Complemen	C3	75.1	187.15	1663	5.3594	1.8483	193	0.886198941
C9J7E5;Q9Y5L0-1;Q9Y5L0;C	10;10;10;10;8;8	Transportin-3	TNPO3	16.6	108.05	957	3.5732	1.8461	10	0.884480703
Q9UK58;C9JPL0;Q9UK58-4;	1;1;1;1	Cyclin-L1	CCNL1	2.9	59.633	526	2.4563	1.8435	2	0.882447416
P35222;P35222-2;E7EMJ5;E	35;5;4;2;2	Catenin beta-1	CTNNB1	63	85.496	781	6.4333	1.8429	81	0.881977789

Q9NZ08-2;Q9NZ08;H0Y9X5	19;19;3	Endoplasmic reticulum aminopeptidase 1	ERAP1	24.2	107.84	948	5.1339	1.8427	16	0.881821213
Q8NI60;Q8NI60-3;Q5T7A4;	3;3;3;3;2;1;1	Chaperone activity of bc1 complex-like, mitochondrial	ADCK3;CABC1	7.7	71.949	647	2.5646	1.8427	2	0.881821213
B4DR61;P61619;P61619-3;(	7;7;4;2;2;2;2;2;2;2;	Protein transport protein Sec61 subunit alpha isoform 1	SEC61A1	28	52.949	482	4.2596	1.8405	10	0.88009775
Q9Y305-4;Q9Y305;Q9Y305-	15;15;15;14;11;7	Acyl-coenzyme A thioesterase 9, mitochondrial	ACOT9	36.2	50.85	448	4.0165	1.8386	11	0.878607646
J3KPD3;Q9Y580;F5GY08;F5(	2;2;1;1;1;1;1	RNA-binding protein 7	RBM7	9.7	30.59	267	3.4011	1.8378	2	0.877979773
F5GYL8;O60684;F5H4G7;Q5	5;5;5;4;3;1	Importin subunit alpha;Importin subunit alpha-7;Importi	KPNA6;KPNA5	14.4	60.593	541	2.5786	1.837	2	0.877351626
Q14669-3;Q14669-2;Q1466	6;6;6;6;2	Probable E3 ubiquitin-protein ligase TRIP12	TRIP12	3.9	225.52	2040	3.2076	1.8358	6	0.876408894
P27338;B7Z242;B7Z5H3;H0\	16;16;14;1	Amine oxidase [flavin-containing] B	MAOB	36.3	58.762	520	5.7774	1.8358	17	0.876408894
Q9UHE5	3	Probable N-acetyltransferase 8	NAT8	15.9	25.619	227	5.4388	1.8355	3	0.876173114
O15120;O15120-2	4;3	1-acyl-sn-glycerol-3-phosphate acyltransferase beta	AGPAT2	17.6	30.914	278	3.4491	1.835	3	0.875780063
Q96Q11;Q96Q11-2;F8W8C:	4;4;1;1;1	CCA tRNA nucleotidyltransferase 1, mitochondrial	TRNT1	9.2	50.127	434	3.0684	1.8338	4	0.874836303
Q7Z406-6;Q7Z406-2;Q7Z40	76;75;75;74;74;63;55	Myosin-14	MYH14	46.6	228.66	2003	5.0615	1.8338	66	0.874836303
J3KPP4;O95232;B4DJ96;D6I	6;6;4;4;2;2;1;1;1;1	Luc7-like protein 3	LUC7L3	16.8	58.22	489	3.5229	1.8329	8	0.874128077
P08709;P08709-2;F5H8B0	2;2;2	Coagulation factor VII;Factor VII light chain;Factor VII hea	F7	5.8	51.593	466	3.2518	1.8326	2	0.873891924
O00429-6;J3KPI2;O00429;C	3;3;3;3;3;3;3;3;2;2;2;	Dynamin-1-like protein	DNM1L	4.5	83.398	749	3.0883	1.8297	3	0.871607122
P26572	2	Alpha-1,3-mannosyl-glycoprotein 2-beta-N-acetylglucos	MGAT1	3.6	50.878	445	2.3522	1.8297	2	0.871607122
G5E948;O95819-3;O95819-!	12;12;12;12;12;12;12	Mitogen-activated protein kinase kinase kinase kinase 4;	MAP4K4;TNIK;MI	11	151.08	1320	3.9055	1.8287	13	0.870818419
Q9H0E2;E7EN89;B3KR28;F2	3;2;2;2;2;2;2;1	Toll-interacting protein	TOLLIP	17.5	30.281	274	5.4045	1.8244	3	0.867422075
Q9NUL7	3	Probable ATP-dependent RNA helicase DDX28	DDX28	6.3	59.58	540	2.6938	1.8226	2	0.865997973
O14787-2;O14787	9;8	Transportin-2	TNPO2	10.8	100.41	887	3.3143	1.8194	3	0.863462758
Q9UBS4;H7C2Y5	12;5	DnaJ homolog subfamily B member 11	DNAJB11	33.8	40.513	358	5.6084	1.8194	16	0.863462758
P52948;P52948-5;P52948-2	6;6;5;5;5;4;2;1	Nuclear pore complex protein Nup98-Nup96;Nuclear por	NUP98	4.1	197.58	1817	3.747	1.8176	3	0.86203474
Q969Z0;Q969Z0-2;C9IZN7;(	17;10;9;8;6	Protein TBRG4	TBRG4	44.5	70.737	631	5.0753	1.8175	19	0.861955364
Q9P0M9;H7C5U8;D6RAN8;	5;5;4;2;2	39S ribosomal protein L27, mitochondrial	MRPL27	35.8	16.073	148	6.0505	1.8158	5	0.860605307
E7EQ34;O14653-2;O14653;(	6;6;6;5;5;4;4;4;1	Golgi SNAP receptor complex member 2	GOSR2	36.6	26.864	232	4.8808	1.8154	12	0.860287463
Q53EP0;Q53EP0-2	4;2	Fibronectin type III domain-containing protein 3B	FNDC3B	4	132.89	1204	3.1969	1.8146	4	0.859651564
H9KV44;Q14156;Q14156-2;	3;3;3;2	Protein EFR3 homolog A	EFR3A	4.9	92.94	821	2.7509	1.8136	3	0.858856296
O75439;G3V0E4;E7ERZ4;F8	15;14;11;6;3;3	Mitochondrial-processing peptidase subunit beta	PMPCB	42.9	54.366	489	4.6672	1.8123	14	0.857821792
Q9BYD3;K7ES61;Q9BYD3-2,	10;10;7;6;4;4;2	39S ribosomal protein L4, mitochondrial	MRPL4	53.1	34.919	311	4.6768	1.8106	16	0.85646786
E9PRY8;P29692-2;P29692;E	12;12;12;11;9;7;7;7;7	Elongation factor 1-delta	EEF1D	23.8	76.569	697	5.5575	1.8105	14	0.856388177
B4DLN1;P52815;Q9UBX3-2,	12;7;5;5;5;1	39S ribosomal protein L12, mitochondrial	SLC25A10;MRPL1:	33.9	48.099	442	5.2801	1.8095	13	0.855591108
Q99570;D6RJ98;D6RBB7;D(	4;1;1;1	Phosphoinositide 3-kinase regulatory subunit 4	PIK3R4	3.1	153.1	1358	3.228	1.8086	2	0.854873369
P63261;I3L3I4;I3L4N8;I3L1L	29;24;16;15;15;12;11	Actin, cytoplasmic 2;Actin, cytoplasmic 2, N-terminally pr	ACTG1	91.5	41.792	375	4.9195	1.8075	214	0.853995647
Q8IZV5;E5RK48	5;3	Retinol dehydrogenase 10	RDH10	18.5	38.087	341	4.459	1.8062	6	0.852957651
P09874	24	Poly [ADP-ribose] polymerase 1	PARP1	31.1	113.08	1014	4.6602	1.8059	21	0.852718007
O15460-2;O15460;E7EPI9;E	17;16;3;3;2;2;2;2;1;1	Prolyl 4-hydroxylase subunit alpha-2	P4HA2	42.6	60.632	533	4.7606	1.8056	15	0.852478324
Q99567;J3KMX1;I3L2W3	3;2;1	Nuclear pore complex protein Nup88	NUP88	8.5	83.541	741	3.6866	1.8047	3	0.851759034
Q8N2U0;K7ERE1;K7ENI6	2;2;2	UPF0451 protein C17orf61	C17orf61	31.9	11.741	113	5.1742	1.804	2	0.851199339
P57105	6	Synaptojanin-2-binding protein	SYNJ2BP	57.9	15.928	145	5.8245	1.8037	5	0.850959403
O75367;O75367-3;O75367-	10;10;10;5;4;1	Core histone macro-H2A.1;Histone H2A	H2AFY	38.7	39.617	372	4.8354	1.8031	7	0.850479411
Q9BYD2;Q5SZR1	10;8	39S ribosomal protein L9, mitochondrial	MRPL9	40.4	30.243	267	5.5164	1.8019	9	0.849518948
Q9UBM7;E9PM00;E9PMT8;	7;4;4;3;2;2;2;2	7-dehydrocholesterol reductase	DHCR7	17.1	54.489	475	4.8662	1.8002	6	0.848157197
P09234;Q5TAL2	4;1	U1 small nuclear ribonucleoprotein C	SNRPC	30.2	17.394	159	4.5678	1.8002	8	0.848157197
P62873;F6UT28;B1AKQ8;F6	14;9;8;7;6;2;2;2;2;2	Guanine nucleotide-binding protein G(I)/G(S)/G(T) subui	GNB1	57.4	37.377	340	4.9842	1.8001	22	0.848077054
P51398;P51398-3;P51398-2	18;18;18	28S ribosomal protein S29, mitochondrial	DAP3	56.5	45.566	398	4.7352	1.7996	21	0.847676272
Q99417	4	C-Myc-binding protein	MYCBP	56.3	11.967	103	3.645	1.799	6	0.847195187
Q8NBL1;H7C5H0;B4DVA9;F	3;1;1;1	Protein O-glucosyltransferase 1	POGLUT1	9.4	46.189	392	2.8805	1.7969	2	0.845510123
P61803;F5GXX5;F5H895	5;5;2	Dolichyl-diphosphooligosaccharide--protein glycosyltran	DAD1	54	12.497	113	5.9102	1.7965	6	0.845188935
Q9Y241-2;C9JNU6;Q9Y241;	3;3;3;3;1	HIG1 domain family member 1A	HIGD1A	45.8	11.77	107	4.7555	1.7957	5	0.844546345
Q9BV40;B8ZZT4;C9JXZ5	6;4;3	Vesicle-associated membrane protein 8	VAMP8	53	11.438	100	5.2576	1.7945	9	0.843581923
P10606	2	Cytochrome c oxidase subunit 5B, mitochondrial	COX5B	9.3	13.696	129	3.1864	1.7919	2	0.841490128
Q9UPY3;Q9UPY3-2;B3KRG4	5;5;2	Endoribonuclease Dicer	DICER1	3.6	218.68	1922	2.9472	1.7915	4	0.841168044
Q9H2M9;Q9H2M9-2;H7C4Y	19;3;1;1	Rab3 GTPase-activating protein non-catalytic subunit	RAB3GAP2	16.4	155.98	1393	4.7355	1.7904	15	0.840281941
Q9HCN8	8	Stromal cell-derived factor 2-like protein 1	SDF2L1	63.3	23.598	221	5.48	1.7903	11	0.84020136
Q92544;F2Z2L1;F8WCN2	6;1;1	Transmembrane 9 superfamily member 4	TM9SF4	11.8	74.518	642	4.5985	1.7852	9	0.836085712
Q04837;E7EUY5;C9K0U8	9;7;5	Single-stranded DNA-binding protein, mitochondrial	SSBP1	58.8	17.259	148	4.8504	1.7848	21	0.835762419
P33991;J3KPV4;E5RG31;E7I	38;38;13;8;7;4;4;1	DNA replication licensing factor MCM4	MCM4	56.8	96.557	863	5.7963	1.7847	41	0.835681584
P84243;K7EK07	7;7	Histone H3.3	H3F3A	48.5	15.328	136	5.635	1.7836	3	0.834792105
Q14566	29	DNA replication licensing factor MCM6	MCM6	40	92.888	821	6.4793	1.7829	28	0.834225787
P68032;P62736;P63267;P68	16;15;15;14;13;13;8;8	Actin, alpha cardiac muscle 1;Actin, aortic smooth muscle	ACTC1;ACTA2;ACT	37.9	42.019	377	5.9715	1.78	9	0.831877241
P15144;H0YMC1;H0YLZ8	26;1;1	Aminopeptidase N	ANPEP	29.4	109.54	967	4.4023	1.78	21	0.831877241
Q15019-2;Q15019;B5MCX3	15;15;14;9;7;7;7;6;6;5	Septin-2	41519	51.8	45.46	396	5.1512	1.7796	21	0.831553004
Q9BVK6	10	Transmembrane emp24 domain-containing protein 9	TMED9	41.3	27.277	235	5.1831	1.7789	17	0.830985412
Q9Y2S7	15	Polymerase delta-interacting protein 2	POLDIP2	53	42.033	368	4.8271	1.7761	8	0.828712812
P14923	33	Junction plakoglobin	JUP	61.7	81.744	745	5.4499	1.7752	36	0.827981573
P60604;A6NMQ7	3;3	Ubiquitin-conjugating enzyme E2 G2	UBE2G2	24.8	18.566	165	4.5926	1.7732	3	0.826355268
Q9Y5J7;G3V502;G3V2F3;G3	6;4;3;1	Mitochondrial import inner membrane translocase subui	TIMM9	77.5	10.378	89	4.2092	1.7718	5	0.825215762
Q13501;Q13501-2;E7EMC7;	3;3;2;2;1;1	Sequestosome-1	SQSTM1	10.9	47.687	440	4.5182	1.7717	2	0.825134335
P54727;Q5W0S5;G5E9P0;H	7;4;3;2;2;1;1;1;1	UV excision repair protein RAD23 homolog B	RAD23B	17.8	43.171	409	3.5947	1.7712	5	0.824727127
Q9NYF8;Q9NYF8-2;E9PK91	7;7;7;7;7;7;7;7;6;2	Bcl-2-associated transcription factor 1	BCLAF1	9.8	106.12	920	4.9958	1.7709	18	0.824482748
Q9HCC0;Q9HCC0-2;D6RD67	21;21;10;8;8	Methylcrotonoyl-CoA carboxylase beta chain, mitochond	MCCC2	49.6	61.332	563	4.9924	1.7702	25	0.823912367
J3KQY1;E7ESL0;Q9NWU5;C	8;7;7;5;5	39S ribosomal protein L22, mitochondrial	MRPL22	37.1	26.475	232	4.6458	1.7692	7	0.823097147
Q92621;F8WCW4;F2Z2D0;E	41;6;6;5	Nuclear pore complex protein Nup205	NUP205	29.3	227.92	2012	3.9408	1.7687	42	0.822689364
Q8N0U8;E7ETM5	3;1	Vitamin K epoxide reductase complex subunit 1-like prot	VKORC1L1	17	19.835	176	3.2907	1.7664	7	0.820812077
Q92665	5	28S ribosomal protein S31, mitochondrial	MRPS31	15.9	45.318	395	4.325	1.7662	5	0.820648719
P82932	9	28S ribosomal protein S6, mitochondrial	MRPS6	67.2	14.226	125	4.9096	1.7638	12	0.818686981
O60716-3;O60716-5;O6071	42;42;42;42;42;42;41	Catenin delta-1	CTNND1	54.5	104.98	939	5.7171	1.7636	79	0.818523382
Q15005;E9PI68;E9PL01;E9P	10;9;9;5;3	Signal peptidase complex subunit 2	SPCS2	53.1	25.003	226	6.0614	1.7627	8	0.817786958
P54710;P54710-2	2;1	Sodium/potassium-transporting ATPase subunit gamma	FXYD2	39.4	7.2832	66	3.7505	1.7622	2	0.817377671
P02787;J3KN47;H7C5E8;C9.	44;36;7;7;5;4;4;1;1	Serotransferrin	TF	64.5	77.063	698	6.7484	1.7587	98	0.814509408
B4DRT2;Q92552;G5EA06;D(	16;16;16;14;7;3	28S ribosomal protein S27, mitochondrial	MRPS27	44.6	49.149	428	4.3064	1.7532	23	0.809990584
Q14197;J3KS15;J3QRF8	6;6;1	Peptidyl-tRNA hydrolase ICT1, mitochondrial	ICT1	32.5	23.63	206	5.6514	1.7528	5	0.809661389
Q6PI48;Q9H9M6	12;2	Aspartate--tRNA ligase, mitochondrial	DARS2	19.7	73.562	645	3.9151	1.7521	12	0.809085118
P48960;P48960-3;P48960-2	2;2;2	CD97 antigen;CD97 antigen subunit alpha;CD97 antigen s	CD97	3.1	91.868	835	4.0187	1.7509	2	0.808096689
P51159;P51159-2;H3BN55;I	7;6;6;5;5;5;3;2;1	Ras-related protein Rab-27A	RAB27A	33.9	24.868	221	5.4137	1.7494	7	0.806860199
E7EUE1;P28288;F5GYC1;P2:	3;3;3;3	ATP-binding cassette sub-family D member 3	ABCD3	6	78.401	683	3.1801	1.7486	3	0.806200304
Q96GW9	3	Methionine--tRNA ligase, mitochondrial	MARS2	6.2	66.59	593	3.4601	1.7475	4	0.805292456
K7EKS3;O00411;K7EMH3;K	22;22;6;1	DNA-directed RNA polymerase, mitochondrial	POLRMT	24	140.21	1245	4.9786	1.7466	23	0.804549245
Q9NP81;Q9NP81-2;E7EX87	17;16;13;9	Serine--tRNA ligase, mitochondrial	SARS2;FBXO17	44.4	58.282	518	4.9044	1.746	14	0.804053559
P00450;E9PFZ2;H7C5R1;D6	13;13;8;2;1;1	Ceruloplasmin	CP	18.8	122.2	1065	4.9273	1.7417	13	0.800496148
Q9NPH3;Q9NPH3-5;Q9NPI	16;9;8;7;5;4;3;2	Interleukin-1 receptor accessory protein	IL1RAP	28.9	65.418	570	4.5875	1.7407	15	0.799667584
O00743-3;O00743;O00743-	7;7;7;5;1	Serine/threonine-protein phosphatase 6 catalytic subuni	PPP6C	24.3	38.946	342	3.8972	1.7402	6	0.799253124
P05556;P05556-3;P05556-4	20;18;18;18;18;6;5;5;	Integrin beta-1	ITGB1	31.1	88.414	798	5.212	1.7397	39	0.798838544
P55795	9	Heterogeneous nuclear ribonucleoprotein H2	HNRNPH2	27.8	49.263	449	4.4082	1.7395	7	0.798672679
E9PLN8;Q9BQB6;Q9BQB6-2	3;3;3;3;3;3	Vitamin K epoxide reductase complex subunit 1	VKORC1	25.3	17.945	166	4.5454	1.7383	4	0.797677087
Q9BSJ8-2;Q9BSJ8;F8VZB1	39;39;5	Extended synaptotagmin-1	ESYT1	48.5	124	1114	5.5664	1.735	77	0.794935663
Q08945;E9PMD4;E9PPZ7	15;6;5	FACT complex subunit SSRP1	SSRP1	28.3	81.074	709	5.5294	1.7349	13	0.794852508
P83916;J3KS05;B5MD17;K7	8;7;6;5;4	Chromobox protein homolog 1	CBX1	60	21.418	185	4.3634	1.7342	7	0.79427029
Q8N983-4;B1AL06;C9J5Q3;	9;8;8;8;8;8;8;8;5	39S ribosomal protein L43, mitochondrial	MRPL43	65.4	17.853	159	5.879	1.7332	9	0.793438142
Q15366-3;Q15366-6;Q1536	11;11;10;10;10;10;9;9	Poly(rC)-binding protein 2	PCBP2	45	38.221	362	4.8708	1.7299	11	0.790688643
Q9BU61;Q9BU61-2	4;3	NADH dehydrogenase [ubiquinone] 1 alpha subcomplex	NDUFAF3	21.7	20.35	184	4.3998	1.7291	2	0.790021308
Q92890-1;Q92890;A6NJ11;,	4;4;4;3;3;2;2	Ubiquitin fusion degradation protein 1 homolog	UFD1L	13.1	38.725	343	4.5525	1.7288	3	0.789770977
Q6S8J3;P0CG38;Q9BYX7;P(	10;7;6;5	POTE ankyrin domain family member E;POTE ankyrin dor	POTEE;POTEI;POT	10.3	121.36	1075	5.5971	1.7284	3	0.789437136
F5H3I5;Q96JB5;Q96JB5-2;J:	11;11;7;6;6;5;5;3;3;3;	CDK5 regulatory subunit-associated protein 3	CDK5RAP3	20.9	59.588	531	3.0746	1.7279	9	0.789019726
P33992;B1AHB1;B1AHB2;F8	30;28;9;6;6	DNA replication licensing factor MCM5	MCM5	48.9	82.285	734	5.6434	1.7278	34	0.788936229
Q16630-2;Q16630;F8WJN3,	6;6;6;6;1	Cleavage and polyadenylation specificity factor subunit 6	CPSF6	15.5	63.47	588	4.712	1.7271	11	0.788351618
Q92598;Q92598-2;B4DYH1;	31;31;30;29;23;3	Heat shock protein 105 kDa	HSPH1	46.4	96.864	858	5.0908	1.7237	24	0.785508704
Q9NRX2;E9PKV2	9;8	39S ribosomal protein L17, mitochondrial	MRPL17	45.1	20.05	175	5.5262	1.7192	10	0.781737388
P29992;K7EL62;O95837;G3\	13;8;5;3;1;1;1;1;1;1	Guanine nucleotide-binding protein subunit alpha-11	GNA11	44	42.123	359	4.1053	1.7175	13	0.780310099
Q15526;Q15526-2	2;2	Surfeit locus protein 1	SURF1	7	33.331	300	4.2658	1.7174	2	0.780226097
Q15428;K7EKV8;K7EMT0;K	9;4;4;4	Splicing factor 3A subunit 2	SF3A2	24.6	49.255	464	3.9938	1.7144	10	0.777703755
A6NEM2;P51610;P51610-2;	7;7;7;3;2;1	Host cell factor 1;HCF N-terminal chain 1;HCF N-terminal	HCFC1	4	213.47	2080	4.5175	1.7141	9	0.777451278
P25205;J3KQ69;Q7Z6P5	40;37;6	DNA replication licensing factor MCM3	MCM3	55.8	90.98	808	5.5679	1.7108	42	0.774671112
Q5T1Z8;Q5T1Z4;E9PCJ0;Q1	3;3;3;3;3;3;3;3;3;2;2	Pumilio homolog 1;Pumilio homolog 2	PUM1;PUM2	3.1	130.11	1224	2.8093	1.7106	2	0.774502445
P13861;Q9BUB1;H7C1L0;C9	10;10;3;3;3;2;1	cAMP-dependent protein kinase type II-alpha regulatory	PRKAR2A	35.9	45.518	404	3.9941	1.7104	8	0.774333758
Q9Y2Q5;Q9Y2Q5-2;Q9Y2Q5	6;5;3	Ragulator complex protein LAMTOR2	LAMTOR2	60	13.507	125	4.3806	1.7088	8	0.772983552
Q14997;Q14997-2;F8WB44,	25;14;12;12;10	Proteasome activator complex subunit 4	PSME4	18.1	211.33	1843	4.0935	1.7083	18	0.772561353
Q9H0N5;H0YA52	4;4	Pterin-4-alpha-carbinolamine dehydratase 2	PCBD2	31.5	14.365	130	4.1429	1.7076	4	0.771970068
Q9BRR6;Q9BRR6-2;E7EQY8	8;8;5;5;3;3;2;2;2;1;1	ADP-dependent glucokinase	ADPGK	20.3	54.088	497	3.6879	1.7072	5	0.771632081
Q9NP77;Q9NP77-2	3;1	RNA polymerase II subunit A C-terminal domain phospha	SSU72	18	22.574	194	2.7934	1.706	3	0.770617647
Q9UHX1;Q9UHX1-2;Q9UHX	17;17;17;17;17;17;10	Poly(U)-binding-splicing factor PUF60	PUF60	43.6	59.875	559	4.9279	1.706	12	0.770617647
Q9HD20;Q9HD20-2	5;4	Probable cation-transporting ATPase 13A1	ATP13A1	5.9	132.95	1204	2.639	1.706	3	0.770617647
Q96JJ7;B4DIE3;Q96JJ7-2	2;1;1	Protein disulfide-isomerase TMX3	TMX3	4.6	51.871	454	2.8471	1.7056	2	0.770279343
O14979;O14979-2;O14979-	11;11;11	Heterogeneous nuclear ribonucleoprotein D-like	HNRPDL	19	46.437	420	3.8158	1.7051	16	0.769856352
Q9NYL9;H0YNJ8;H0YKU1;H	6;4;4;1	Tropomodulin-3	TMOD3	25.9	39.594	352	4.1034	1.7038	4	0.768755995
P36404;G3V184	9;6	ADP-ribosylation factor-like protein 2	ARL2	58.7	20.878	184	4.0653	1.7003	7	0.765789317
O14964;O14964-2;I3L1P5;I3	6;6;4;1;1	Hepatocyte growth factor-regulated tyrosine kinase subs	HGS	8.6	86.191	777	3.2235	1.7001	4	0.765619608
B7Z5Z2;P62070;P62070-3;E	7;7;6;6;5;2;1;1	Ras-related protein R-Ras2	RRAS2	39	24.195	210	5.3687	1.6991	10	0.764770764
P21964;P21964-2;E7EMS6;E	17;17;12;11;6;6	Catechol O-methyltransferase	COMT	73.4	30.037	271	4.4272	1.6983	31	0.764091329
Q14126;J3KSI6	17;3	Desmoglein-2	DSG2	21.7	122.29	1118	4.9443	1.6982	17	0.764006378

Q16401;B4DZM8;F2Z3J2;Q4	9;9;4;1	26S proteasome non-ATPase regulatory subunit 5	PSMD5		19.8	56.195	504	3.9229	1.698	7	0.763836459
P62995;P62995-3;H7BXF3;H	8;8;5;4	Transformer-2 protein homolog beta	TRA2B		35.4	33.665	288	4.4679	1.6962	14	0.762306289
O60613;A8MZD0;O60613-2	5;2;2	15 kDa selenoprotein		41532	48.8	17.808	162	4.0443	1.6957	7	0.761880954
Q9UIQ6;Q9UIQ6-2;Q9UIQ6	7;7;7	Leucyl-cystinyl aminopeptidase;Leucyl-cystinyl aminope	LNPEP		9.2	117.35	1025	3.9718	1.6957	4	0.761880954
Q13505;Q13505-3;H0Y6Y5;(	6;6;5;3	Metaxin-1	MTX1		16.7	51.476	466	4.5355	1.6939	5	0.760348707
P20700;E9PBF6	33;28	Lamin-B1	LMNB1		56.1	66.408	586	4.7138	1.6931	50	0.759667186
P43686;P43686-2	16;15	26S protease regulatory subunit 6B	PSMC4		47.1	47.366	418	4.383	1.6918	15	0.758559027
Q7Z7K0;H7C3D8;C9JES7	5;4;2	COX assembly mitochondrial protein homolog	CMC1		52.8	12.49	106	3.7117	1.6893	6	0.756425557
O14617-5;O14617;O14617-	7;7;7;5;5;5;5;1	AP-3 complex subunit delta-1	AP3D1		6.5	136.65	1215	3.9461	1.6887	6	0.755913054
F5GZX4;Q9H9Y6;Q9H9Y6-2;	3;3;3;3;3;1	DNA-directed RNA polymerase;DNA-directed RNA polym	POLR1B		3.2	132.24	1173	2.4198	1.6882	2	0.755485829
P20290;P20290-2;D6RDG3;	2;2;2;2	Transcription factor BTF3	BTF3		19.9	22.168	206	3.8269	1.6881	2	0.755400369
P61289-2;P61289;K7ESG5;E	9;9;7;7;6;4;4;4;2	Proteasome activator complex subunit 3	PSME3		38.2	30.886	267	4.4186	1.6862	11	0.753775664
Q9BZJ0;Q5JY65;Q9BZJ0-3;C	3;3;3;3	Crooked neck-like protein 1	CRNKL1		3.3	100.45	848	4.0967	1.6857	3	0.753347806
P98179	5	Putative RNA-binding protein 3	RBM3		47.8	17.17	157	4.8308	1.6854	9	0.75309103
Q9BV79;Q9BV79-2	2;2	Trans-2-enoyl-CoA reductase, mitochondrial	MECR		5.1	40.461	373	2.7031	1.6836	2	0.751549415
Q8TC12;Q8TC12-2;B4DDW(	11;10;7;7;6;5;5;4	Retinol dehydrogenase 11	RDH11		45	35.386	318	4.9382	1.6819	12	0.75009193
H3BLZ8;Q92841;Q92841-3;(	28;28;26;26	Probable ATP-dependent RNA helicase DDX17	DDX17		45.1	80.439	731	5.6681	1.6804	43	0.748804691
Q9UBT2;B3KWB9;K7EPL2;K	7;5;3;3;1	SUMO-activating enzyme subunit 2	UBA2		14.1	71.223	640	4.5079	1.6776	7	0.746398767
Q9BZQ6;B7ZLZ2;H0Y498	8;8;3	ER degradation-enhancing alpha-mannosidase-like 3	EDEM3		10.2	104.66	932	3.8965	1.6772	6	0.746054735
Q14435;C9J2C3;E7EUL0;Q1	4;2;2;1	Polypeptide N-acetylgalactosaminyltransferase 3	GALNT3		7.7	72.609	633	3.6266	1.6765	3	0.745452483
Q8WUK0;Q8WUK0-2;E9PQ	5;5;3;3	Protein-tyrosine phosphatase mitochondrial 1	PTPMT1		30.3	22.843	201	4.0835	1.6763	6	0.745280365
Q08170;F6T1J1	6;4	Serine/arginine-rich splicing factor 4	SRSF4		12.3	56.678	494	4.9095	1.6762	3	0.745194298
O75935;O75935-3;O75935-	2;2;1;1;1	Dynactin subunit 3	DCTN3		8.6	21.119	186	3.2688	1.6755	2	0.744591687
O43447;C9JQD4;A6NNE7;H	7;7;4;4;3	Peptidyl-prolyl cis-trans isomerase H;Peptidyl-prolyl cis-	PPIH		44.6	19.208	177	3.7593	1.6748	8	0.743988823
Q9Y333	5	U6 snRNA-associated Sm-like protein LSm2	LSM2		68.4	10.834	95	4.696	1.674	9	0.743299528
Q00765;E2QRG8;H0Y8J8;B7	8;3;1;1;1;1	Receptor expression-enhancing protein 5	REEP5		31.7	21.493	189	3.7241	1.6737	10	0.743040957
Q9BUL8;C9J5C3;C9J363;C9J	4;3;3;2;2;2;2;1;1	Programmed cell death protein 10	PDCD10		19.3	24.701	212	4.828	1.6703	2	0.740107246
Q9NY12;Q9NY12-2	2;2	H/ACA ribonucleoprotein complex subunit 1	GAR1		11.5	22.348	217	3.3618	1.6696	2	0.739502506
O43172;O43172-2	13;13	U4/U6 small nuclear ribonucleoprotein Prp4	PRPF4		28.9	58.449	522	4.1046	1.6687	11	0.738724609
P50750-2;P50750	8;8	Cyclin-dependent kinase 9	CDK9		17.4	53.365	489	3.0239	1.6656	8	0.736041974
Q86V81	11	THO complex subunit 4	ALYREF		50.6	26.888	257	3.5766	1.663	27	0.733788169
P20020;P20020-4;P20020-3	31;31;31;31;28;28;23	Plasma membrane calcium-transporting ATPase 1	ATP2B1		28.5	138.75	1258	5.1216	1.6583	44	0.729705026
Q6P1L8	6	39S ribosomal protein L14, mitochondrial	MRPL14		42.8	15.947	145	4.8304	1.6583	9	0.729705026
B0V043;P26640;H0Y4A6;E7	56;56;53;26;18;12;8;7	Valine--tRNA ligase	VARS		56.7	140.46	1264	5.3582	1.658	71	0.729444007
Q9Y676;Q5STN0;B4DFG6	8;5;4	28S ribosomal protein S18b, mitochondrial	MRPS18B		46.5	29.395	258	4.4544	1.6575	7	0.72900887
P30876;C9J2Y9;C9J4M6	15;15;15	DNA-directed RNA polymerase II subunit RPB2;DNA-dire	POLR2B		14.2	133.9	1174	3.8516	1.6572	18	0.728747725
H0YG20;Q9UKM7;H0YGV7;	4;4;3;3;1;1	Endoplasmic reticulum mannosyl-oligosaccharide 1,2-alp	MAN1B1		8.2	79.898	705	3.0155	1.6556	4	0.727354154
Q9Y4Z0	7	U6 snRNA-associated Sm-like protein LSm4	LSM4		51.1	15.35	139	4.7621	1.6543	9	0.726220885
Q9H082;Q14088	3;1	Ras-related protein Rab-33B	RAB33B		14.4	25.717	229	2.897	1.652	2	0.724213687
Q96CS3	6	FAS-associated factor 2	FAF2		18	52.623	445	5.1324	1.6505	5	0.722903139
P12235;Q9H0C2	10;3	ADP/ATP translocase 1	SLC25A4		31.5	33.064	298	3.8176	1.6501	2	0.722553458
P12814;P12814-3;P12814-2	62;61;59;58;51;20;18	Alpha-actinin-1	ACTN1		78.7	103.06	892	5.7902	1.6494	69	0.721941313
P60510;H3BTA2;H3BV22;I3	6;4;4;2	Serine/threonine-protein phosphatase 4 catalytic subuni	PPP4C		23.5	35.08	307	4.0131	1.6491	4	0.721678885
P62805	15	Histone H4	HIST1H4A		61.2	11.367	103	3.4421	1.6476	61	0.720366031
P30533;D6REW6	17;1	Alpha-2-macroglobulin receptor-associated protein	LRPAP1		42.9	41.465	357	5.011	1.6472	23	0.720015735
Q9BRA2;I3L3M7;I3L0K2;I3L	4;3;3;2	Thioredoxin domain-containing protein 17	TXNDC17		38.2	13.941	123	3.3056	1.6438	4	0.717034778
P30042;H7C2G3;H7C1F6;P3	3;3;2;2;2;2;2	ES1 protein homolog, mitochondrial	C21orf33		16.8	28.17	268	3.2618	1.6425	2	0.715893371
Q02818;H7BZI1;C9JKZ2;C9J	5;3;2;1;1	Nucleobindin-1	NUCB1		13	53.879	461	2.3007	1.6414	2	0.714926858
Q99541;E9PG83;Q5SYF5;Q5	16;10;4;3;3	Perilipin-2	PLIN2		52.4	48.075	437	4.7532	1.6411	16	0.714663152
P49590;B4DDY8;E9PG66;C9	3;3;3;2;2;2;1;1	Probable histidine--tRNA ligase, mitochondrial	HARS2;HARSL		6.5	56.888	506	2.7586	1.6401	2	0.713783781
P00491;G3V5M2;G3V308;G	7;4;2;2;1	Purine nucleoside phosphorylase	PNP		31.1	32.118	289	3.9562	1.6383	6	0.712199563
B7WP27;Q9HCG8;B7WP74	3;3;3	Pre-mRNA-splicing factor CWC22 homolog	CWC22		4.8	105.52	908	2.8712	1.6375	2	0.711494907
P35637;P35637-2;H3BPE7	9;9;8	RNA-binding protein FUS	FUS		21.9	53.425	526	4.4081	1.6368	24	0.71087805
P08621;P08621-2;P08621-3	16;15;10;6	U1 small nuclear ribonucleoprotein 70 kDa	SNRNP70		34.8	51.556	437	5.0315	1.6357	18	0.709908171
P24928;Q6NX41	41;13	DNA-directed RNA polymerase II subunit RPB1	POLR2A		26.3	217.17	1970	3.8712	1.6356	40	0.709819968
Q13242;H0YIB4	12;8	Serine/arginine-rich splicing factor 9	SRSF9		53.4	25.542	221	3.8827	1.6335	22	0.707966455
P61978-2;P61978;Q5T6W1	23;23;13	Heterogeneous nuclear ribonucleoprotein K	HNRNPK		64	51.028	464	4.6439	1.6316	68	0.706287412
Q9UKX7;Q9UKX7-2;B4E2D3	9;9;5;2	Nuclear pore complex protein Nup50	NUP50		32.5	50.144	468	4.2206	1.6314	9	0.706110557
Q7Z7H8-2;Q7Z7H8	4;4	39S ribosomal protein L10, mitochondrial	MRPL10		33.9	30.307	271	5.3956	1.6287	6	0.70372089
P50336;H0YFP3;B4DQQ7;F5	5;2;1;1;1	Protoporphyrinogen oxidase	PPOX		16.8	50.765	477	3.0871	1.6274	5	0.702568896
P0DI81-3;G1K394;P0DI81;P	2;2;2;2;1	Trafficking protein particle complex subunit 2;Trafficking	TRAPPC2;TRAPPC		14.9	20.374	174	3.1099	1.6255	2	0.700883556
Q6P1A2;F5H0M4;F5GYT3;B	2;1;1;1;1;1	Lysophospholipid acyltransferase 5	LPCAT3		8	56.034	487	4.4754	1.6243	2	0.699818116
P82650;G5E9V5;G5E9W7;H	19;19;19;9;9;9;8;1	28S ribosomal protein S22, mitochondrial	MRPS22		56.7	41.28	360	4.2815	1.6241	26	0.699640466
P31930	5	Cytochrome b-c1 complex subunit 1, mitochondrial	UQCRC1		12.5	52.645	480	3.0515	1.6234	5	0.699018519
P82675;P82675-2	14;6	28S ribosomal protein S5, mitochondrial	MRPS5		35.6	48.006	430	4.1731	1.621	11	0.696884091
Q92791;K7ERA3	2;2	Synaptonemal complex protein SC65	LEPREL4		5.3	50.381	437	2.913	1.6209	2	0.696795088
Q14203;A8MY36;E7EX90;E9	22;22;22;22;22;20;20	Dynactin subunit 1	DCTN1;DKFZp686		23.5	141.69	1278	3.6735	1.6202	15	0.696171913
Q7KZ85;Q7KZ85-3;Q7KZ85-	58;17;17	Transcription elongation factor SPT6	SUPT6H		41.1	199.07	1726	4.4449	1.62	57	0.695993813
Q9UEY8;Q9UEY8-2	3;3	Gamma-adducin	ADD3		5.7	79.154	706	3.6197	1.6199	3	0.695904755
P07954;P07954-2	14;14	Fumarate hydratase, mitochondrial	FH		42.2	54.636	510	4.7062	1.6199	12	0.695904755
Q86TM6;Q86TM6-3;E9PK19	3;3;3;3;2;2;2	E3 ubiquitin-protein ligase synoviolin	SYVN1		4.5	67.684	617	2.2841	1.6198	4	0.695815692
O75882;O75882-2;O75882-	14;12;11	Attractin	ATRN		12.1	158.54	1429	3.7202	1.6192	12	0.695281195
O43402;C9JB21	3;2	Neighbor of COX4	COX4NB		13.8	23.773	210	2.3903	1.6162	2	0.692605738
O75251;F5GXJ1;B3KRI2;F5H	4;3;3;3	NADH dehydrogenase [ubiquinone] iron-sulfur protein 7	NDUFS7		27.7	23.563	213	4.974	1.6153	4	0.691802133
Q9P2R3-4;Q9P2R3-2;Q9P2I	6;6;6;5;1;1	Ankyrin repeat and FYVE domain-containing protein 1	ANKFY1		6.8	132.82	1211	3.2178	1.6136	4	0.690282989
P11047;Q5JTC4;Q9Y6N6	39;1;1	Laminin subunit gamma-1	LAMC1		30.9	177.6	1609	4.4333	1.613	37	0.689746438
P60059	2	Protein transport protein Sec61 subunit gamma	SEC61G		36.8	7.7412	68	3.7711	1.6123	2	0.689120211
Q5T4U5;P11310-2;P11310;E	6;6;6;6;4;2;1	Medium-chain specific acyl-CoA dehydrogenase, mitocho	ACADM		20.3	50.27	454	3.2531	1.6103	4	0.687329488
Q96EY8;J3KN55;F5H4Z7;F5	3;2;1;1	Cob(I)yrinic acid a,c-diamide adenosyltransferase, mitoc	MMAB		11.6	27.388	250	4.5456	1.6076	3	0.684908483
Q9NUI1;Q4VXZ8;Q9NUI1-3	11;11;8;4;3;3;3	Peroxisomal 2,4-dienoyl-CoA reductase	DECR2		46.6	30.777	292	4.3949	1.6069	10	0.684280151
Q9Y2Q9;H0YAT2;H7C5V3;E	7;6;5;5;4;3;2;1	28S ribosomal protein S28, mitochondrial	MRPS28		43.9	20.843	187	4.6293	1.6069	10	0.684280151
Q96KP4;Q96KP4-2;J3QKT2;	2;2;1;1;1;1;1;1;1;1;1;	Cytosolic non-specific dipeptidase	CNDP2		4	52.878	475	2.5474	1.6039	2	0.681584195
B7Z888;Q9UPN6	4;4	Protein SCAF8	SCAF8		3.4	147.34	1337	3.5721	1.6033	4	0.681044399
Q14240-2;Q14240;E7EQG2;	10;10;10;6;4;2;2;2;2	Eukaryotic initiation factor 4A-II	EIF4A2		28.2	46.489	408	4.5293	1.6018	2	0.679694025
Q5JTZ9	9	Alanine--tRNA ligase, mitochondrial	AARS2		12.1	107.34	985	3.9216	1.601	8	0.678973308
P21266	8	Glutathione S-transferase Mu 3	GSTM3		40	26.559	225	2.8416	1.5997	7	0.677801374
P63000;P63000-2;P60763;P	11;10;7;5;5;5;4;4;4	Ras-related C3 botulinum toxin substrate 1;Ras-related C	RAC1;RAC3		68.8	21.45	192	4.4508	1.5997	11	0.677801374
Q8N3U4-2;Q8N3U4;F8WAK	18;18;18;7;2;1;1;1;1;	Cohesin subunit SA-2	STAG2		17.8	145.75	1268	4.6882	1.5996	16	0.677711186
I3L0E3;Q9Y2R5;E9PE17	4;4;4	28S ribosomal protein S17, mitochondrial	MRPS17		29.8	25.72	225	4.262	1.5994	6	0.677530793
A0FGR8-2;H7BXI1;A0FGR8-	9;9;8;8;5;1;1	Extended synaptotagmin-2	ESYT2		14.3	98.9	893	3.4629	1.5993	9	0.677440588
Q9BXP5;Q9BXP5-3;Q9BXP5	32;32;32;32;31;22;4;2	Serrate RNA effector molecule homolog	SRRT		38	100.67	876	4.9051	1.5947	26	0.673285045
Q9H0U6	5	39S ribosomal protein L18, mitochondrial	MRPL18		33.9	20.576	180	4.6757	1.5947	6	0.673285045
H3BP13;Q9UL33;Q9UL33-2;	2;2;2;2;1;1	Trafficking protein particle complex subunit 2-like protei	TRAPPC2L		9.5	27.302	241	2.5057	1.5945	2	0.673104098
O15260;Q5T8U7;Q5T8U5;O	6;6;6;6;4	Surfeit locus protein 4	SURF4		27.5	30.394	269	3.6629	1.5907	35	0.669661774
Q9P013	4	Protein CWC15 homolog	CWC15		18.3	26.624	229	4.3048	1.5903	3	0.669298946
O43809;H3BND3;H3BV41	13;9;5	Cleavage and polyadenylation specificity factor subunit 5	NUDT21		67.4	26.227	227	3.3738	1.5899	16	0.668936027
P35573;P35573-3;P35573-2	11;10;10	Glycogen debranching enzyme;4-alpha-glucanotransfera	AGL		8.6	174.76	1532	2.9596	1.5895	10	0.668573016
E9PGN7;B4E1F0;P05155;H9	1;1;1;1;1	Plasma protease C1 inhibitor	SERPING1		2.4	59.491	543	5.839	1.5892	2	0.668300699
Q15149;Q15149-2;Q15149-	205;205;205;205;205	Plectin	PLEC		50.8	531.78	4684	3.6567	1.5874	205	0.666665711
Q9NZL4;K7EN20	2;2	Hsp70-binding protein 1	HSPBP1		5.8	39.474	362	2.3327	1.5857	2	0.665119852
P17980;E9PM69;E9PKD5;E9	18;17;12;12;10;7;5;3;	26S protease regulatory subunit 6A	PSMC3		54.7	49.203	439	3.9433	1.5839	20	0.663481253
Q08AM6;H3BQD9;B3KSM8;	5;3;2;2	Protein VAC14 homolog	VAC14		8.1	87.972	782	2.6065	1.5835	4	0.663116867
Q8WWY3;E7ESA8;E7EVX8;(	9;9;8;8;7;6;6;6	U4/U6 small nuclear ribonucleoprotein Prp31	PRPF31		23.6	55.455	499	4.5313	1.5835	9	0.663116867
Q5SWX8;Q5SWX8-4;Q5SW	5;5;5;3	Protein odr-4 homolog	ODR4		15.9	51.103	454	3.4643	1.5816	5	0.661384776
Q96HS1;Q96HS1-2;F5GXG4	3;3;1	Serine/threonine-protein phosphatase PGAM5, mitocho	PGAM5		10	32.004	289	3.7186	1.5815	2	0.661293556
P26641;B4DTG2;E7EMT2	24;23;12	Elongation factor 1-gamma	EEF1G		66.1	50.118	437	4.5676	1.5806	28	0.660472313
P45973;G3V1X9;F8VNY3	10;10;3	Chromobox protein homolog 5	CBX5		53.4	22.225	191	3.5254	1.5803	20	0.660198462
Q9Y4Y9;B8ZZF8	1;1	U6 snRNA-associated Sm-like protein LSm5	LSM5		20.9	9.9374	91	4.7129	1.5801	2	0.660015865
P48509;E9PMR4;E9PL82;E9	2;2;2;1	CD151 antigen	CD151		7.5	28.295	253	5.4181	1.5801	3	0.660015865
Q7Z5G4;J3KQ24;J3KN76;Q2	4;4;4;1	Golgin subfamily A member 7	GOLGA7		30.7	15.824	137	4.8702	1.5797	5	0.659650603
Q9H0U3;Q6P577	5;3	Magnesium transporter protein 1	MAGT1		15.2	38.036	335	2.994	1.5785	5	0.658554261
Q9P2W9;D6RF48;H0YAL4;D	2;2;1;1;1	Syntaxin-18	STX18		6.3	38.673	335	1.9509	1.5774	2	0.657548548
P19387;H3BRR2	4;1	DNA-directed RNA polymerase II subunit RPB3	POLR2C		21.1	31.441	275	3.3945	1.5764	4	0.656633655
P52758;H0YB34;H0YBX3;E5	6;5;4;4	Ribonuclease UK114	HRSP12		73	14.494	137	3.4617	1.5761	7	0.656359073
O00203;E5RJ68;B7ZKS0;Q1	5;5;1;1;1;1	AP-3 complex subunit beta-1	AP3B1		4.8	121.32	1094	2.4799	1.5759	3	0.65617599
P57740;B4DZ67;Q6PJE1;H0	11;11;8;1	Nuclear pore complex protein Nup107	NUP107		12.2	106.37	925	3.6995	1.5744	6	0.654802126
P17612;P17612-2;K7ERP6;C	3;3;2;2	cAMP-dependent protein kinase catalytic subunit alpha	PRKACA;KIN27		8.8	40.589	351	4.0623	1.5722	3	0.652784755
P36776;Q8N8K8;K7EKE6;K7	17;17;17;17;15;6;4;2;	Lon protease homolog, mitochondrial;Lon protease hom	LONP1		21.8	106.49	959	4.0791	1.5722	13	0.652784755
P62316;J3KPM5;K7ERG4;K7	11;8;6;3	Small nuclear ribonucleoprotein Sm D2	SNRPD2		67.8	13.527	118	3.9184	1.5698	30	0.650580765
Q5T653;C9IY40	5;3	39S ribosomal protein L2, mitochondrial	MRPL2		27.2	33.3	305	4.231	1.5693	6	0.650121176
O95249;E9PCW1;G5E9T8;K7	7;7;6;6;5;5;2	Golgi SNAP receptor complex member 1	GOSR1		31.6	28.612	250	3.6079	1.5676	8	0.648557478
Q02790;F5H1U3;H0YFG2	8;1;1	Peptidyl-prolyl cis-trans isomerase FKBP4;Peptidyl-proly	FKBP4		22.9	51.804	459	3.8875	1.567	8	0.64800518
Q8WWX9	1	Selenoprotein M	SELM		9.7	16.249	145	3.7602	1.5651	2	0.646254839
F5H7K4;J3KN69;Q6PIU2-2;(	5;5;5;5;3;3;1	Neutral cholesterol ester hydrolase 1	NCEH1		13.6	49.881	448	4.1285	1.5642	4	0.645424989
Q15154;E7ETA6;Q15154-2;I	2;2;2;2;1;1;1	Pericentriolar material 1 protein	PCM1		1	228.53	2024	4.616	1.5632	2	0.644502372
Q92506	8	Estradiol 17-beta-dehydrogenase 8	HSD17B8		39.8	26.973	261	3.9407	1.563	9	0.644317778
P07339;H7C469;C9JH19;F8V	14;10;8;6;6;4	Cathepsin D;Cathepsin D light chain;Cathepsin D heavy cl	CTSD		38.1	44.552	412	3.2722	1.5628	25	0.644133161
F8W8Q7;Q13439;Q13439-4	15;15;15;15;15;4;1	Golgin subfamily A member 4	GOLGA4		7.3	262.33	2243	3.1215	1.5624	13	0.643763854
Q16629;C9JAB2;G5E9M3;Q	8;8;8;8;8;2	Serine/arginine-rich splicing factor 7	SRSF7		29.4	27.366	238	4.4001	1.562	10	0.643394453
C9JME2;Q9Y4F1-2;Q9Y4F1;	21;21;21;3;2;1	FERM, RhoGEF and pleckstrin domain-containing protein	FARP1		24.8	122.09	1076	3.8804	1.5618	17	0.643209718
B7ZKL3;Q9H6S3;Q9H6S3-2	2;2;1	Epidermal growth factor receptor kinase substrate 8-like	EPS8L2		3.6	82.322	731	3.1175	1.5613	2	0.642747774
P08582;P08582-2	5;1	Melanotransferrin	MFI2		7.5	80.214	738	4.4754	1.56	4	0.641546029
Q9UFN0;Q9BS92;F2Z3L7	7;2;2	Protein NipSnap homolog 3A	NIPSNAP3A		34.8	28.466	247	3.7959	1.5595	9	0.641083553
O60231;B0V0F8;Q5JP46;Q5	3;2;2;2;1	Putative pre-mRNA-splicing factor ATP-dependent RNA I	DHX16		3.1	119.26	1041	2.5013	1.5594	2	0.64099104
P84103;B4E241	10;10	Serine/arginine-rich splicing factor 3	SRSF3		46.3	19.329	164	4.0002	1.5584	24	0.640065583
Q13247;Q13247-3;Q13247-	9;9;4	Serine/arginine-rich splicing factor 6	SRSF6		24.4	39.586	344	4.9937	1.5564	18	0.638212885
Q9BU23;Q9BU23-2;Q9BU23	2;2;2;1	Lipase maturation factor 2	LMF2		3.4	79.697	707	2.5783	1.5564	2	0.638212885
B7Z4Q3;Q96HJ9	5;2		LUC7L2		12.2	54.223	458	2.6394	1.5558	4	0.637656612
Q15056;Q15056-2	5;5	Eukaryotic translation initiation factor 4H	EIF4H		28.2	27.385	248	2.9397	1.5548	4	0.636729013

P14625;Q58FF3;H0YIV0;F8\	65;10;8;4;2	Endoplasmin	HSP90B1	66.7	92.468	803	4.3006	1.5541	215	0.636079338
Q07021;I3L3B0;I3L3Q7	10;8;8	Complement component 1 Q subcomponent-binding pro	C1QBP	62.4	31.362	282	4.9618	1.5541	38	0.636079338
O75179;O75179-2;E7EUV3;	4;4;4;4;4;3;2;2;2;2;2;	Ankyrin repeat domain-containing protein 17;Ankyrin re	ANKRD17;ANKHD	1.7	274.25	2603	2.5148	1.5468	2	0.62928667
Q8WVC0;Q8WVC0-2	4;4	RNA polymerase-associated protein LEO1	LEO1	7.1	75.403	666	2.8738	1.5468	3	0.62928667
O00487;C9JW37	10;2	26S proteasome non-ATPase regulatory subunit 14	PSMD14	55.5	34.577	310	4.0161	1.5463	12	0.628820246
P51665;B4DXI8;H3BTM8;H3	14;11;8;8;2	26S proteasome non-ATPase regulatory subunit 7	PSMD7	60.2	37.025	324	3.8396	1.5461	30	0.628633634
P61981	15	14-3-3 protein gamma;14-3-3 protein gamma, N-terminal	YWHAG	66.4	28.302	247	3.4846	1.5455	20	0.628073654
Q9Y490;Q5TCU6	109;102	Talin-1	TLN1	59.9	269.76	2541	3.7671	1.5452	133	0.627793583
O75165;H0Y8Q2	11;2	DnaJ homolog subfamily C member 13	DNAJC13	5.8	254.41	2243	2.4396	1.545	6	0.627606838
P61586;E9PN11;P08134;Q5	11;7;7;7;7;7;6;6;6;3;3	Transforming protein RhoA;Rho-related GTP-binding pro	RHOA;RHOC	71.5	21.768	193	3.8474	1.5432	21	0.625925048
Q92900;Q92900-2	19;19	Regulator of nonsense transcripts 1	UPF1	20.5	124.34	1129	3.5906	1.5419	20	0.624709202
Q9H2U2-2;Q9H2U2;E2QRM	11;11;10;10;9;7;7;5;4	Inorganic pyrophosphatase 2, mitochondrial	PPA2	41.8	39.638	349	3.7202	1.5418	8	0.624615633
P08183;B5AK60;P21439;P2	4;4;1;1;1;1	Multidrug resistance protein 1	ABCB1	5	141.48	1280	2.7425	1.5417	2	0.624522058
P41235-4;P41235;P41235-2	3;3;3;3;3;3;3;1;1	Hepatocyte nuclear factor 4-alpha	HNF4A	8.1	56.138	504	3.1267	1.54	3	0.622930351
O14773;O14773-2	7;7	Tripeptidyl-peptidase 1	TPP1	18.5	61.247	563	4.8087	1.5393	9	0.622274431
Q9BYN8	11	28S ribosomal protein S26, mitochondrial	MRPS26	42.4	24.211	205	3.747	1.5381	15	0.621149304
J3KQB0;Q9NXG2;H3BNW0	4;4;2	THUMP domain-containing protein 1	THUMPD1	12.8	48.548	439	3.1935	1.5374	2	0.620492574
Q8IZA0;E7EN73;Q8IZA0-2;(	6;6;6;4;4;2;1	Dyslexia-associated protein KIAA0319-like protein	KIAA0319L	5.2	115.66	1049	3.4222	1.5357	4	0.618896412
F8W9N1;Q9UHK6;D6RB81;(	3;3;3;2;2	Alpha-methylacyl-CoA racemase	AMACR	9.1	43.86	394	3.0808	1.5346	2	0.61786266
Q13492;Q13492-5;Q13492-	7;7;7;7;7;3;3;2;2;2;2;	Phosphatidylinositol-binding clathrin assembly protein	PICALM	15.2	70.754	652	4.0061	1.5341	6	0.617392528
Q1KMD3;H3BQZ7	27;25	Heterogeneous nuclear ribonucleoprotein U-like protein	HNRNPUL2;hCG_2	41.6	85.104	747	5.1189	1.5341	24	0.617392528
O95168;O95168-2;C9JXQ9;	3;3;1;1	NADH dehydrogenase [ubiquinone] 1 beta subcomplex s	NDUFB4	31.8	15.208	129	4.7064	1.5335	2	0.616828167
Q92945	33	Far upstream element-binding protein 2	KHSRP	52.5	73.114	711	5.5384	1.5316	50	0.615039565
P62820;E7END7;B7Z8M7;P(	11;9;9;7;7;6;1;1	Ras-related protein Rab-1A	RAB1A	68.3	22.677	205	3.8738	1.531	30	0.614474283
Q86VP6;Q86VP6-2;H0YH27	32;29;7;7;2;2;2;1	Cullin-associated NEDD8-dissociated protein 1	CAND1	34	136.37	1230	3.7173	1.5305	42	0.614003045
Q7L2E3-2;Q7L2E3;H7BXY3;(	17;17;17;17	Putative ATP-dependent RNA helicase DHX30	DHX30	18.7	136.11	1222	3.3739	1.5291	19	0.612682759
Q15286;F5H157;Q15286-2;I	9;8;4;1	Ras-related protein Rab-35	RAB35	50.7	23.025	201	4.2944	1.5284	8	0.612022163
P02786;G3V0E5;F5H6B1;H7	33;30;20;5;3	Transferrin receptor protein 1;Transferrin receptor prote	TFRC	52.9	84.87	760	5.6065	1.5283	53	0.611927767
Q14571;B7ZMI3;Q14571-2	29;2;1	Inositol 1,4,5-trisphosphate receptor type 2	ITPR2	12.1	308.06	2701	3.206	1.5277	22	0.611361264
Q13200;E7EW34;E9PCS3;H7	37;32;31;9;7;4;2	26S proteasome non-ATPase regulatory subunit 2	PSMD2	56.5	100.2	908	4.3582	1.5274	53	0.611077929
P62633;P62633-2;P62633-3	3;3;3;3;2;2;2	Cellular nucleic acid-binding protein	CNBP	19.8	19.463	177	4.8106	1.5273	2	0.610983471
D3DVS1;B7Z3K1;Q5BJH2	2;2;1	Transmembrane protein 128	TMEM128	16.4	18.822	165	3.0176	1.5266	2	0.610322096
Q96I24;Q96I24-2	8;4	Far upstream element-binding protein 3	FUBP3	16.8	61.64	572	3.5519	1.5256	6	0.609376749
P34896;P34896-2;B4DPM9;	6;6;6;4;4	Serine hydroxymethyltransferase, cytosolic;Serine hydro	SHMT1	14.5	53.082	483	2.1013	1.5256	3	0.609376749
P58107	37	Epiplakin	EPPK1	36.4	555.61	5090	3.3366	1.5236	28	0.607484193
Q9UHN6;Q9UHN6-2;F5H6B	42;41;15;11;6;4	Transmembrane protein 2	TMEM2	37.2	154.37	1383	4.6462	1.5231	43	0.607010666
P61964	3	WD repeat-containing protein 5	WDR5	12.3	36.588	334	2.7234	1.5226	2	0.606536983
P26358-2;F5GX68;P26358;F	19;19;19;16;6;2;1;1	Cytosine-specific methyltransferase;DNA (cytosine-5)-m	DNMT1	14.8	184.82	1632	3.8553	1.521	13	0.605020153
P23368;P23368-2;Q16798;E	8;4;1;1;1;1	NAD-dependent malic enzyme, mitochondrial	ME2	17.8	65.443	584	3.1205	1.5196	6	0.603691617
P49643	2	DNA primase large subunit	PRIM2	4.3	58.805	509	1.8364	1.5184	2	0.602551897
P48556;K7EJR3;K7EJC1;K7E	12;11;7;4;3	26S proteasome non-ATPase regulatory subunit 8	PSMD8	43.1	39.611	350	4.0725	1.5184	15	0.602551897
E7ENU4;P55265-4;P55265;F	14;14;14;14;13;13;13	Double-stranded RNA-specific adenosine deaminase	ADAR	13.9	140.83	1269	3.072	1.5182	9	0.602361857
P50213;H0YL72;P50213-2;B	8;8;5;5;5;4;3;3;2;1;1;	Isocitrate dehydrogenase [NAD] subunit alpha, mitochor	IDH3A	27.3	39.591	366	3.0059	1.5172	3	0.601411277
P61020;B4DKD7;H0YHA1;F8	8;6;4;4	Ras-related protein Rab-5B	RAB5B	47.9	23.707	215	4.4728	1.5166	4	0.600840628
P30740;B4DNT0	3;3	Leukocyte elastase inhibitor	SERPINB1	8.2	42.741	379	3.2886	1.5143	3	0.598651048
Q15006;H0YAS9	4;2	Tetratricopeptide repeat protein 35	TTC35	14.1	34.833	297	3.4353	1.514	3	0.598365205
O00754;G5E928	4;4	Lysosomal alpha-mannosidase;Lysosomal alpha-mannos	MAN2B1	5	113.74	1011	4.3086	1.5139	4	0.598269912
P05997	4	Collagen alpha-2(V) chain	COL5A2	4.7	144.91	1499	2.7886	1.5135	4	0.597888675
P50851;F5H1X8;P50851-2;E	19;19;19;18;11;2	Lipopolysaccharide-responsive and beige-like anchor pro	LRBA	7.3	319.1	2863	2.8904	1.513	12	0.597411988
H0YMD1;J3KMZ9;P01130;H	2;2;2;2;2;2;2;2;1	Low-density lipoprotein receptor	LDLR	3.1	104.75	948	2.6038	1.511	2	0.595503661
P82933;H7BZC4	16;1	28S ribosomal protein S9, mitochondrial	MRPS9	55.6	45.834	396	3.9298	1.5109	17	0.595408178
P21127;J3QR44;J3QKR5;P2	14;14;14;14;14;14;14	Cyclin-dependent kinase 11B;Cyclin-dependent kinase 1	CDK11B;CDK11A;(	19.5	92.706	795	4.0263	1.51	15	0.59454855
O00422;H7BZW6	5;4	Histone deacetylase complex subunit SAP18	SAP18	34	17.561	153	2.474	1.5085	4	0.593114696
Q5JVF3-4;Q5JVF3;Q5JVF3-	5;5;5;4	PCI domain-containing protein 2	PCID2	13.7	52.099	453	2.0747	1.5072	6	0.59187087
Q96ST2;Q96ST2-3;Q96ST2-	5;5;5	Protein IWS1 homolog	IWS1	7.1	91.954	819	2.5279	1.507	3	0.591679417
Q8N7V9;P54619;B4DDT7;F	5;5;5;5;4;3;2;2;1;1;1;	5-AMP-activated protein kinase subunit gamma-1	PRKAG1	22.1	38.532	340	2.513	1.5069	6	0.591583681
Q8N138;Q8N138-4;J3QRM	4;4;3;3	ORM1-like protein 3	ORMDL3	34	17.494	153	3.4854	1.5057	6	0.590434352
P42695;G3V1A9;E9PKK4;E9	5;4;3;2;1	Condensin-2 complex subunit D3	NCAPD3	4.3	168.89	1498	2.653	1.5042	5	0.588996402
Q96RP9;C9IZ01;Q96RP9-2;I	6;6;5;5;2	Elongation factor G, mitochondrial	GFM1	10.5	83.471	751	3.1801	1.5037	5	0.588516767
Q96PK6;B8ZZ74;Q96PK6-2;	5;3;3;3;3;3;3	RNA-binding protein 14	RBM14;RBM14-RE	10.6	69.491	669	4.3668	1.5029	2	0.587749018
Q5TGA3;Q9UNP9;Q9UNP9-	3;3;3;3;3	Peptidyl-prolyl cis-trans isomerase E;Peptidyl-prolyl cis-t	PPIE	10.8	34.991	314	2.342	1.5023	3	0.587172939
O43242;B4DT72;H0YGV8	25;18;5	26S proteasome non-ATPase regulatory subunit 3	PSMD3	49.1	60.977	534	4.0129	1.5015	26	0.586404475
Q9BUQ8;H0YI52;F8VVA2	20;3;1	Probable ATP-dependent RNA helicase DDX23	DDX23	27.1	95.581	820	4.6669	1.5007	18	0.585635601
Q99519;E9PIF4	2;2	Sialidase-1	NEU1	4.6	45.467	415	2.492	1.5005	3	0.585443319
Q9UKS6;E9PIY1;E9PNM9;E	3;3;2;2	Protein kinase C and casein kinase substrate in neurons p	PACSIN3	7.3	48.486	424	3.6335	1.4988	3	0.583807883
Q8NE71;Q8NE71-2;H0YGW	13;12;10;5;5;5;3;1	ATP-binding cassette sub-family F member 1	ABCF1	18.2	95.925	845	4.4025	1.4977	11	0.582748671
Q9UBV2;Q9UBV2-2;G3V3B	15;4;3	Protein sel-1 homolog 1	SEL1L	30.7	88.754	794	4.4913	1.4963	13	0.581399457
Q9UNM6;Q9UNM6-2;J3KN	22;21;19;13;11;3;1	26S proteasome non-ATPase regulatory subunit 13	PSMD13	73.4	42.945	376	4.1891	1.496	25	0.581110175
Q99459	11	Cell division cycle 5-like protein	CDC5L	18.5	92.25	802	4.2873	1.4954	6	0.580531438
Q8N1S5;Q8N1S5-2;J3QS49;	2;2;2;2;2;1;1;1;1	Zinc transporter ZIP11	SLC39A11	10.2	35.396	342	3.7456	1.4928	2	0.578020891
Q8N2G8;E9PDB5;K7ESN3;C	6;6;5;5;1;1;1	GH3 domain-containing protein	GHDC	11.9	57.522	530	3.0817	1.4927	6	0.577924245
Q9UMX5	6	Neudesin	NENF	42.4	18.856	172	3.1798	1.4924	4	0.577634265
Q93074-2;Q93074;Q93074-	4;4;4;4;1	Mediator of RNA polymerase II transcription subunit 12	MED12	2	243.39	2180	2.3364	1.4921	3	0.577344228
O43157;O43157-2;O43157-	5;5;3;2	Plexin-B1	PLXNB1	3	232.3	2135	3.2922	1.4913	4	0.576570509
B3KQV7;Q99808	4;4	Equilibrative nucleoside transporter 1	SLC29A1	8.4	58.962	535	2.863	1.4877	3	0.573083631
P02545;P02545-3;Q6UYC3;I	39;38;37;31;31	Prelamin-A/C;Lamin-A/C	LMNA	59.6	74.139	664	3.8578	1.4835	68	0.569004927
J3QRS3;O14950;P19105;P2	7;7;7;5;4;3	Myosin regulatory light chain 12B;Myosin regulatory light	MYL12B;MYL12A;I	49.2	20.457	177	4.1376	1.4834	12	0.568907674
P51149;C9J8S3;C9J592;C9J	18;14;12;12;9;9;6	Ras-related protein Rab-7a	RAB7A	77.3	23.489	207	4.3044	1.4827	35	0.568226722
Q92973;Q92973-2;Q92973-	22;22;19;5;5;4;4;1;1	Transportin-1	TNPO1	35.9	102.35	898	3.6853	1.482	21	0.567545448
P51572-2;P51572;C9J0M4;(	14;14;9;9;9;8;4	B-cell receptor-associated protein 31	BCAP31	35.5	34.752	313	3.2645	1.482	27	0.567545448
P49721	12	Proteasome subunit beta type-2	PSMB2	82.6	22.836	201	3.0054	1.4813	28	0.566863852
Q9H5Q4	10	Dimethyladenosine transferase 2, mitochondrial	TFB2M	25.8	45.348	396	3.2896	1.4803	8	0.565889584
Q9NVP1;H7C452	6;2	ATP-dependent RNA helicase DDX18	DDX18	10.3	75.406	670	2.0545	1.4785	5	0.564134243
Q9Y399;Q5T8A0	12;10	28S ribosomal protein S2, mitochondrial	MRPS2	39.9	33.249	296	3.9681	1.4755	18	0.561203921
P31942;P31942-2;P31942-3	9;9;8;7;6;4;4	Heterogeneous nuclear ribonucleoprotein H3	HNRNPH3	42.8	36.926	346	4.0941	1.4753	13	0.561008354
O60925;E5RGS4;D6RGG5	4;4;1	Prefoldin subunit 1	PFDN1	32	14.21	122	2.4138	1.4736	3	0.559344967
Q5JWU6;O43399-5;O43399	7;7;7;6;6;6;4	Tumor protein D54	TPD52L2	46.7	24.853	229	3.1461	1.4728	5	0.558561532
C9JJ19;P82930;H3BP88	11;11;6	28S ribosomal protein S34, mitochondrial	MRPS34	55.6	26.332	225	4.3356	1.4715	11	0.557287542
P26038	12	Moesin	MSN	17.5	67.819	577	2.9639	1.4712	2	0.556993385
J9JIE6;Q9UM00;Q9UM00-2	4;4;4;3;2;1	Transmembrane and coiled-coil domain-containing prote	TMCO1	19.2	27.079	239	3.5734	1.471	5	0.556797247
Q7L8L6	4	FAST kinase domain-containing protein 5	FASTKD5	7.3	86.573	764	2.1023	1.47	5	0.555816155
O14802	5	DNA-directed RNA polymerase III subunit RPC1	POLR3A	3.7	155.64	1390	2.5339	1.4699	2	0.555718009
I3L416;I3L3T0;I3L0X9;I3L1U	3;3;3;3;3	Mitochondrial import inner membrane translocase subur	PAM16	4.7	114.16	1048	3.8855	1.4691	3	0.554932602
O94826	18	Mitochondrial import receptor subunit TOM70	TOMM70A	35.9	67.454	608	4.2614	1.4691	31	0.554932602
O75694;O75694-2;E9PF10;I	30;29;28;1	Nuclear pore complex protein Nup155	NUP155	28.6	155.2	1391	4.3583	1.468	24	0.553851968
Q99729-2;D6RBZ0;Q99729-	9;9;9;9;9;8;8	Heterogeneous nuclear ribonucleoprotein A/B	HNRNPAB	27.1	35.967	332	2.9475	1.4669	20	0.552770524

O94874;O94874-2;O94874-	16;13;13	E3 UFM1-protein ligase 1	UFL1		27.2	89.594	794	4.096	1.4669	17	0.552770524
Q5SRE5;Q5SRE5-2;H7C4K7;	7;7;1;1	Nucleoporin NUP188 homolog	NUP188		4.3	196.04	1749	3.0143	1.465	6	0.550900665
A7MAP1;A7MAP0;Q9ULV4	12;12;12;10;6;3;3;3;3	Coronin-1C	CORO1C		28.8	58.947	527	4.2132	1.4641	9	0.550014095
Q9HAV7	9	GrpE protein homolog 1, mitochondrial	GRPEL1		47.5	24.279	217	3.1907	1.4607	7	0.546659906
P62318;B4DJP7;H3BT13	5;5;3	Small nuclear ribonucleoprotein Sm D3	SNRPD3		53.2	13.916	126	3.2382	1.4603	18	0.546264783
O75475;O75475-2;O75475-	4;2;2	PC4 and SFRS1-interacting protein	PSIP1		8.3	60.103	530	2.2707	1.4598	5	0.545770726
Q969X5;Q969X5-2;F2Z3A2;	7;7;3;3;3;3;3	Endoplasmic reticulum-Golgi intermediate compartment	ERGIC1		24.5	32.592	290	2.8999	1.4589	5	0.544880997
P20338;P61018-2;P61018	4;1;1	Ras-related protein Rab-4A	RAB4A		22.1	23.871	213	3.3239	1.4576	3	0.543594864
Q13435;E9PPJ0;H0YCG1;E9	33;30;13;13;12;8;4;2	Splicing factor 3B subunit 2	SF3B2		39.9	100.23	895	4.2912	1.4545	43	0.540523296
G3V1K3;Q15165;Q15165-1;	10;10;9;9;9;6	Serum paraoxonase/arylesterase 2	PON2		56.3	41.527	375	3.6708	1.4544	28	0.540424105
Q9H307;Q9H307-2;G3V579	10;8;1;1	Pinin	PNN		15.9	81.613	717	3.9009	1.4538	9	0.539828811
O14672;B4DU28	4;1	Disintegrin and metalloproteinase domain-containing pr	ADAM10		7	84.141	748	3.6729	1.4514	4	0.537445175
H3BU86;G8JLE0;O14662;O1	7;7;7;7;7;7;7;6;6;6;6;	Syntaxin-16	STX16-NPEPL1;ST		30.1	43.229	382	3.6953	1.4495	7	0.535555333
O43278;O43278-2;F5H877;I	4;4;4;4;4;3;3;1;1	Kunitz-type protease inhibitor 1	SPINT1		8.7	58.397	529	2.603	1.4472	4	0.533264313
Q8N4T8;H0Y962;Q8N4T8-2	7;5;5;1;1;1	Carbonyl reductase family member 4	CBR4		37.1	25.301	237	3.746	1.4459	9	0.531967777
Q13162;H7C3T4;A6NJJ0;A6	19;11;8;7	Peroxiredoxin-4	PRDX4		74.9	30.54	271	3.996	1.4456	34	0.531668411
O14657;H0Y7C8	3;2	Torsin-1B	TOR1B		12.8	37.978	336	3.001	1.4436	3	0.529671048
Q7Z3B4;B4DT35;E7EUM5;Q	5;5;5;3	Nucleoporin p54	NUP54		12.2	55.435	507	3.1951	1.4427	4	0.528771332
P19388	7	DNA-directed RNA polymerases I, II, and III subunit RPAE	POLR2E		45.7	24.551	210	3.3456	1.4426	7	0.528671329
G8JLA2;P60660-2;B7Z6Z4;G	9;9;8;8;8;8;8;7;6;6;5;	Myosin light polypeptide 6	MYL6		73	17.089	152	4.0299	1.4423	19	0.528371278
O14936;H9KV37;O14936-2;	10;10;10;10;10;10;10	Peripheral plasma membrane protein CASK	CASK		16.4	105.12	926	3.4655	1.4412	5	0.527270557
Q15008;C9IZE4;Q6UV22;E9	25;24;20;20;10;8;6	26S proteasome non-ATPase regulatory subunit 6	PSMD6		65.3	45.531	389	3.7886	1.44	32	0.526068812
Q587I9	4	Vesicle transport protein SFT2C	SFT2D3		27	21.789	215	3.5616	1.4391	4	0.525166845
P82914	9	28S ribosomal protein S15, mitochondrial	MRPS15		28.8	29.842	257	3.893	1.4384	8	0.524464926
P27708;F8VPD4;H7C2E4;H7	38;38;5;5;2	CAD protein;Glutamine-dependent carbamoyl-phosphat	CAD		21.3	242.98	2225	3.3031	1.4383	41	0.524364624
Q9NRF8	5	CTP synthase 2	CTPS2		11.4	65.677	586	2.3122	1.4373	2	0.523361219
P07099;B1AQP9;B1AQP8	24;6;4	Epoxide hydrolase 1	EPHX1		66.4	52.948	455	4.4607	1.4341	26	0.520145627
O94925;O94925-3;H7BZD1;	10;10;4;2;1;1;1;1;1	Glutaminase kidney isoform, mitochondrial	GLS		22.6	73.46	669	3.2554	1.4338	8	0.519843797
O43795;E9PDF6;O43795-2;	10;10;10;5;4;1	Unconventional myosin-Ib	MYO1B		12	131.98	1136	3.0811	1.431	5	0.517023672
Q9Y3D9;J3QLR8	9;8	28S ribosomal protein S23, mitochondrial	MRPS23		52.1	21.77	190	4.6043	1.4308	15	0.516822023
A4D212;Q8N201;H7C582	11;11;1	Integrator complex subunit 1	DKFZP586J0619;IN		5.9	265.75	2394	3.0804	1.4298	8	0.515813357
P62879;E7EP32;C9JXA5;C9J	12;9;9;9;8;5;4;2;2	Guanine nucleotide-binding protein G(I)/G(S)/G(T) subu	GNB2		47.9	37.331	340	3.7014	1.4287	8	0.51470301
O95602;B9ZVN9	24;23	DNA-directed RNA polymerase I subunit RPA1;DNA-direc	POLR1A		18.3	194.81	1720	3.5358	1.4282	18	0.514198023
Q9Y3D5;D6RCM2;H0YAG5	4;3;3	28S ribosomal protein S18c, mitochondrial	MRPS18C		31.7	15.85	142	2.9928	1.4275	3	0.513490746
O43427;O43427-2;E9PSD3;	2;2;1;1;1	Acidic fibroblast growth factor intracellular-binding prot	FIBP		5.2	41.878	364	2.7233	1.4272	2	0.51318752
J3KQ18;P30046;B5MC82;A6	6;6;5;5;5;1	D-dopachrome decarboxylase;D-dopachrome decarboxy	DDT;DDTL		44.7	14.192	132	4.5872	1.4239	6	0.50984783
Q92542;Q92542-2;E7ENA9;	7;7;6;5;4;3;3;2;1;1	Nicastrin	NCSTN		13.5	78.41	709	3.3238	1.4234	11	0.509341141
P06213;P06213-2;P08069;C	20;20;4;4;1	Insulin receptor;Insulin receptor subunit alpha;Insulin re	INSR		16.8	156.33	1382	3.2963	1.4205	25	0.506398832
Q15907	12	Ras-related protein Rab-11B	RAB11B		57.8	24.488	218	4.2024	1.4202	17	0.506094112
D6RER5;Q9NVA2;D6RGI3;C	8;8;8;7;6;3;3;3;3;2;	Septin-11		41528	23.8	49.808	432	4.2299	1.4184	10	0.504264441
P62333;H0YJC0;H0YJS8;H0Y	14;6;4;2;1;1	26S protease regulatory subunit 10B	PSMC6		44.2	44.172	389	3.7018	1.4182	19	0.504061001
B7Z2A4;P18433;P18433-3;F	4;4;4;4;4;4	Receptor-type tyrosine-protein phosphatase alpha	PTPRA		6.4	91.92	813	2.9024	1.4181	4	0.503959271
P62195;A8K3Z3;J3QQM1;J3	17;16;11;10;9;9;6;6;5	26S protease regulatory subunit 8	PSMC5		58.1	45.626	406	4.0953	1.4178	19	0.503654035
J3KN10;P42356;P42356-2;A	11;11;6;4;4;4;2;2	Phosphatidylinositol 4-kinase alpha	PI4KA		7.4	236.83	2102	2.7962	1.4163	10	0.502126889
E9PF49;Q9Y6M9;E7EWZ0;E	2;2;2;2	NADH dehydrogenase [ubiquinone] 1 beta subcomplex s	NDUFB9		10.4	26.585	221	2.7161	1.4155	2	0.501311749
Q8NBS9;Q86UY0;B2RDM2	17;17;17	Thioredoxin domain-containing protein 5	TXNDC5		47	47.628	432	4.5812	1.414	19	0.49978212
Q9Y5B9;G3V2X0;G3V401;G	24;1;1;1	FACT complex subunit SPT16	SUPT16H		27.8	119.91	1047	3.3884	1.4135	16	0.499271883
Q96AB3;Q96AB3-2;K7EKW	6;5;5;4;3	Isochorismatase domain-containing protein 2, mitochon	ISOC2		68.3	22.337	205	3.8669	1.4129	6	0.49865936
P07237;H7BZ94;F5H8J2;I3L	41;36;35;35;20;19;17	Protein disulfide-isomerase	P4HB		71.7	57.116	508	4.1251	1.4128	98	0.498557248
Q13257;Q8IZX3	6;2	Mitotic spindle assembly checkpoint protein MAD2A	MAD2L1		38.5	23.51	205	3.2512	1.4113	6	0.497024694
Q14498;Q14498-2;Q14498-	12;12;12;11;10;9;3;2;	RNA-binding protein 39	RBM39		28.1	59.379	530	3.6864	1.4113	12	0.497024694
P49458;P49458-2;E9PE20	6;4;4	Signal recognition particle 9 kDa protein	SRP9		55.8	10.112	86	3.9557	1.4112	5	0.496922466
P62310	4	U6 snRNA-associated Sm-like protein LSm3	LSM3		73.5	11.845	102	4.4361	1.4096	4	0.495285829
P49750-4;P49750;F8VU51;F	9;8;8;7;7;4	YLP motif-containing protein 1	YLPM1		4.6	241.64	2146	2.1428	1.4092	8	0.49487638
P30101;G5EA52	43;42	Protein disulfide-isomerase A3	PDIA3		74.9	56.782	505	3.8313	1.409	121	0.494671612
P18084;H7C4W1;H7C5U2	14;5;4	Integrin beta-5	ITGB5		24.3	88.053	799	3.6305	1.409	14	0.494671612
P62308;A8MWD9;F5H013;C	5;4;4;4;2;2	Small nuclear ribonucleoprotein G;Small nuclear ribonuc	SNRPG		76.3	8.496	76	3.9136	1.4088	8	0.494466814
O95777;C9JNV3;F2Z2Y6;C9	6;5;5;4	N-alpha-acetyltransferase 38, NatC auxiliary subunit	NAA38		89.6	10.403	96	4.4032	1.4077	8	0.493339909
O43148-2;O43148;K7EP06;I	5;5;5;3;1	mRNA cap guanine-N7 methyltransferase	RNMT		11.5	57.724	504	3.1388	1.407	4	0.492622329
P51946;D6RG18;D6RHI7	4;4;4	Cyclin-H	CCNH		16.7	37.643	323	3.2222	1.4064	3	0.492006976
P80303;P80303-2;E9PKG6;J	5;5;5;4;3;3;3;3;1;1	Nucleobindin-2	NUCB2		15.7	50.195	420	3.3887	1.4059	3	0.491493981
Q8WXF1;Q8WXF1-2	14;14	Paraspeckle component 1	PSPC1		31.4	58.743	523	3.9244	1.4053	14	0.490878146
Q9BSE5	14	Agmatinase, mitochondrial	AGMAT		43.2	37.66	352	3.5563	1.4051	14	0.49067281
Q562R1	9	Beta-actin-like protein 2	ACTBL2		24.2	42.003	376	4.4708	1.405	4	0.49057013
Q96EP5;Q96EP5-2;K7EQ02;	7;7;7;3	DAZ-associated protein 1	DAZAP1		25.3	43.383	407	4.4513	1.4027	7	0.488206488
O95983;O95983-2;K7EIE8	4;4;4	Methyl-CpG-binding domain protein 3	MBD3		12.4	32.844	291	3.4243	1.4014	4	0.486868801
O15091;O15091-2;O15091-	3;3;3;1;1	Mitochondrial ribonuclease P protein 3	KIAA0391		5.8	67.315	583	2.6855	1.4012	3	0.486662893
O75340;Q2YDC2;H0Y9N1;H	4;4;2;2;1;1	Programmed cell death protein 6	PDCD6		31.9	21.868	191	3.7988	1.3997	5	0.485117645
Q9Y4L1;E9PL22;E9PJ21;B7Z	52;48;39;31;14;13;8;8	Hypoxia up-regulated protein 1	HYOU1		58.1	111.33	999	4.9877	1.3985	87	0.483880254
P61088;F8VV71;F8VSD4;Q5	7;5;5;4;4;3	Ubiquitin-conjugating enzyme E2 N;Putative ubiquitin-co	UBE2N;UBE2NL		53.3	17.138	152	2.6294	1.3977	8	0.483054736
P61224;B4DQI8;A6NIZ1;F5I	12;12;10;10;10;9;9;9;	Ras-related protein Rap-1b;Ras-related protein Rap-1b-li	RAP1B		62	20.825	184	3.0681	1.3975	23	0.482848283
G5E9Q2;E9PGZ0;O75534;O	4;4;4;4;4	Cold shock domain-containing protein E1	CSDE1		4.1	93.741	844	2.5836	1.3965	3	0.481815574
O00571;O00571-2;O15523;I	22;22;16;16;9;4;3;3;1	ATP-dependent RNA helicase DDX3X;ATP-dependent RN	DDX3X;DDX3Y		40	73.243	662	3.6814	1.396	24	0.481298942
Q9BWJ5	5	Splicing factor 3B subunit 5	SF3B5		83.7	10.135	86	2.9512	1.3957	11	0.480988873
O95159;E9PNY1;E9PQ47;E9	7;5;5;4;3;2;2;1	Zinc finger protein-like 1	ZFPL1		29.7	34.114	310	3.8595	1.3951	4	0.480368537
Q9Y6E2;E7ETZ4;B5MCH7;B5	4;4;3;3;2;1;1;1;1	Basic leucine zipper and W2 domain-containing protein 2	BZW2		9.3	48.162	419	3.1295	1.3941	2	0.479334051
E7EP74;Q14789;E7EU81;H0	55;55;24;15;1	Golgin subfamily B member 1	GOLGB1		19.9	377.13	3269	3.1156	1.3936	30	0.478816529
Q96DI7;B4DQJ1;Q9NSS8	11;10;3	U5 small nuclear ribonucleoprotein 40 kDa protein	SNRNP40		43.1	39.31	357	3.1926	1.3934	10	0.478609468
Q9NQ94;Q9NQ94-4;F8W9F	4;4;4;4;4;4;3;3;1;1;1;	APOBEC1 complementation factor	A1CF		9.1	65.202	594	3.5582	1.392	2	0.477159211
O43707;D6PXK4;F5GXS2;H7	69;48;37;22;11;7	Alpha-actinin-4	ACTN4		80.6	104.85	911	4.9758	1.3916	171	0.476744584
Q86Y82;B1AJQ6	3;3	Syntaxin-12	STX12		15.2	31.642	276	3.6064	1.388	3	0.473007568
Q9Y291;C9JBY7	4;3	28S ribosomal protein S33, mitochondrial	MRPS33		31.1	12.629	106	2.4627	1.3864	4	0.47134356
P09661;H0YKK0;H0YMA0;H	12;7;6;5	U2 small nuclear ribonucleoprotein A	SNRPA1		60	28.415	255	3.4157	1.3856	24	0.470510835
Q8IXI1	1	Mitochondrial Rho GTPase 2	RHOT2		1.6	68.117	618	2.1206	1.3853	2	0.47019844
Q96E29;Q96E29-2;G3V130;	2;2;2;1;1	mTERF domain-containing protein 1, mitochondrial	MTERFD1		6	47.971	417	1.9752	1.3844	2	0.469260847
O00232;O00232-2	19;17	26S proteasome non-ATPase regulatory subunit 12	PSMD12		46.3	52.904	456	3.918	1.3839	23	0.468739698
Q5HY81;P11441	4;4	Ubiquitin-like protein 4A	UBL4A		23.3	20.569	180	2.8908	1.3837	4	0.468531186
Q9NPJ8-3;Q9NPJ8-2;Q9NP	2;2;2;2;1	NTF2-related export protein 2;NTF2-related export prote	NXT2;NXT1		7.6	22.728	197	3.5969	1.3825	2	0.46727948
J3KTL2;Q07955;Q07955-2;C	15;15;14;14;6;5;5	Serine/arginine-rich splicing factor 1	SRSF1		46.6	28.329	253	3.9114	1.3792	19	0.46383168
Q9NPA8;G3V117;E5RHX8;E	5;5;2;1;1;1;1	Enhancer of yellow 2 transcription factor homolog	ENY2		41.6	11.528	101	3.7013	1.3787	3	0.463308566
Q13217	16	DnaJ homolog subfamily C member 3	DNAJC3		45.4	57.579	504	3.8517	1.3783	15	0.462889938
O00231;O00231-2;J3QRY4;J	18;17;6;2	26S proteasome non-ATPase regulatory subunit 11	PSMD11		48.6	47.463	422	4.0523	1.3783	19	0.462889938
Q6IN85;Q6IN85-2;G3V5Z3;	8;8;7;7;5;5;3;1;1;1	Serine/threonine-protein phosphatase 4 regulatory subu	SMEK1		11.2	95.367	833	3.2458	1.3781	7	0.462680579
O96011;O96011-2;H7C3V6	4;3;1	Peroxisomal membrane protein 11B	PEX11B		14.7	28.431	259	2.5549	1.3774	2	0.461947582
O00148;Q8N5M0;B1Q2N1;	17;11;9;8;7;4	ATP-dependent RNA helicase DDX39A	DDX39A;DDX39		48.7	49.129	427	4.164	1.377	4	0.461528559
Q9HBD4;P51532;P51532-4;	21;21;21;21;21;21;11	Transcription activator BRG1;Probable global transcriptio	SMARCA4;SMARC		15.7	188.15	1679	3.48	1.3768	14	0.461319002
Q53GS9;B9A018;B8ZZD1;G5	10;10;9;9;7;3;3;1	U4/U6.U5 tri-snRNP-associated protein 2	USP39		25.8	65.38	565	3.6812	1.3761	10	0.460585313
O95295	4	SNARE-associated protein Snapin	SNAPIN		41.9	14.874	136	2.9602	1.3749	3	0.459326692
O00410-3;O00410;H0Y8C6;	37;37;34;32;31;8;7;7;	Importin-5	IPO5		47	125.54	1115	4.3114	1.3746	33	0.459011864
C9JWM7;F8WCF6;P59998;I	11;11;11;8;6;6;6;3;2	Actin-related protein 2/3 complex subunit 4	ARPC4;ARPC4-TTL		64.7	21.588	187	2.9594	1.3742	16	0.458591988
Q9UKN8	2	General transcription factor 3C polypeptide 4	GTF3C4		3.4	91.981	822	2.3187	1.3724	3	0.456701031
Q9Y3D3;B4E032;A6ND22	7;4;4	28S ribosomal protein S16, mitochondrial	MRPS16		69.3	15.345	137	4.1884	1.3714	9	0.455649427
Q9BT73	3	Proteasome assembly chaperone 3	PSMG3		30.3	13.104	122	2.26	1.3711	3	0.455333797
P28838;P28838-2;H0Y9Q1;I	13;13;5;1	Cytosol aminopeptidase	LAP3		32.8	56.166	519	2.8059	1.3707	2	0.454912848
P13521;C9JQI2;C9JDT0	2;1;1	Secretogranin-2;Secretoneurin	SCG2		2.9	70.94	617	3.4903	1.3707	2	0.454912848
Q92905;E5RHH5;E5RHF2;E5	7;3;1;1;1;1	COP9 signalosome complex subunit 5	COPS5		20.1	37.578	334	2.8014	1.3698	5	0.453965265
P46940;H0YLE8;Q86VI3;F22	64;41;5;5;3	Ras GTPase-activating-like protein IQGAP1	IQGAP1		47.7	189.25	1657	4.3716	1.3695	60	0.453649266
H3BVG0;Q8N1F7;Q8N1F7-2	8;8;5;3;3;3;2;2;2;2;1;	Nuclear pore complex protein Nup93	NUP93		14.4	99.554	880	3.8735	1.3671	8	0.451118776
Q12874;E7EUT8	20;14	Splicing factor 3A subunit 3	SF3A3		49.5	58.848	501	3.479	1.3628	20	0.446573853
O43290;E9PQI8	13;3	U4/U6.U5 tri-snRNP-associated protein 1	SART1		22.2	90.254	800	4.3926	1.3628	10	0.446573853
P52292;J3KS65;J3QLL0;J3Q	15;4;4;1	Importin subunit alpha-2	KPNA2		46.9	57.861	529	3.3408	1.3626	15	0.446362112
P00966;Q5T6L6;Q5T6L5;Q5	6;4;3;2	Argininosuccinate synthase	ASS1		18.4	46.53	412	3.3907	1.3612	6	0.444879056
P31948;G3XAD8;F5H0T1;F5	23;22;20;10;8;5	Stress-induced-phosphoprotein 1	STIP1		42.5	62.639	543	3.5395	1.3597	29	0.443288375
P61201-2;P61201;B4DIH5;H	5;5;5;1	COP9 signalosome complex subunit 2	COPS2		15.1	52.404	450	2.176	1.3594	5	0.442970028
Q96KR6	3	Protein FAM210B	FAM210B		20.8	20.424	192	3.0172	1.3585	3	0.442014566
Q9HBH5;C9J2C7	8;1	Retinol dehydrogenase 14	RDH14		33.9	36.864	336	3.0458	1.3571	6	0.440527032
O94973-2;O94973;O94973-	27;27;21;18;6;3;3;3;2	AP-2 complex subunit alpha-2	AP2A2		41.5	104.09	940	3.5877	1.3553	19	0.438612232
B4DKT0;Q9H8Y8;Q9H8Y8-2	5;5;3	Golgi reassembly-stacking protein 2	GORASP2		17	48.674	464	3.5869	1.3538	6	0.437014622
P30153;F5H3X9;E9PH38;P3	16;15;14;3;3;3;3;3;3;	Serine/threonine-protein phosphatase 2A 65 kDa regulat	PPP2R1A		39.6	65.308	589	2.9961	1.3529	17	0.436055206
Q9GZT3;J3KMY7;G3V2S9;G	11;11;8;8;8;7;3;3;1	SRA stem-loop-interacting RNA-binding protein, mitoch	SLIRP		78	12.349	109	3.6876	1.3513	15	0.434348001
P62191;B4DR63;G3V4X1	13;10;1	26S protease regulatory subunit 4	PSMC1		38.2	49.184	440	3.9974	1.3507	16	0.433707277
Q9H7D7;Q9H7D7-2;H0Y9R3	3;3;1;1	WD repeat-containing protein 26	WDR26		7	72.123	661	3.6405	1.3504	2	0.433386809
P78330;C9JBI3	7;7	Phosphoserine phosphatase	PSPH		37.8	25.007	225	3.0195	1.3487	6	0.431569476
Q15036;B4DQM7;B4DDM3;	2;2;1;1;1;1	Sorting nexin-17	SNX17		3.8	52.901	470	2.8621	1.3474	2	0.430178204
Q5VT66-2;Q5VT66;H7BYZ9;	6;6;5;5;2	MOSC domain-containing protein 1, mitochondrial		41334	22.3	39.453	354	3.1331	1.3472	5	0.429964044
H7BZJ3	10	Thioredoxin	PDIA3		77.2	13.519	123	4.0392	1.3466	6	0.42932137
Q15758;E9PC01;B4DWS4	7;3;3	Neutral amino acid transporter B(0)	SLC1A5		16.3	56.598	541	4.3227	1.3462	11	0.428892762
P48735;B4DFL2;B4DSZ6;H0	24;23;17;11	Isocitrate dehydrogenase [NADP], mitochondrial;Isocitra	IDH2		50.2	50.909	452	4.0193	1.3461	44	0.42878559
O14981	3	TATA-binding protein-associated factor 172	BTAF1		2.4	206.89	1849	2.1297	1.3458	3	0.428464026
Q9Y606;Q9Y606-2;G8JLB3;F	4;4;4;4;2;1;1;1;1	tRNA pseudouridine synthase A, mitochondrial;Pseudou	PUS1		9.8	47.47	427	3.0892	1.3457	3	0.428356822
P31431;B4E1S6;P31431-2	2;2;1	Syndecan-4;Syndecan	SDC4		10.6	21.641	198	2.6701	1.3449	2	0.427498905
H7C1N3;O15155;Q68DU7;C	2;2;1;1;1	BET1 homolog	BET1;DKFZp781C0		21.8	14.872	133	2.6063	1.3449	4	0.427498905

Q16186	3	Proteasomal ubiquitin receptor ADRM1	ADRM1	9.1	42.153	407	4.0531	1.3439	3	0.426425791
Q6NZY4;Q6NZY4-2;F5GXQ(	4;3;2;2;2;1	Zinc finger CCHC domain-containing protein 8	ZCCHC8	8.8	78.576	707	3.1804	1.343	4	0.425459305
Q96AJ9;Q96AJ9-1	3;3	Vesicle transport through interaction with t-SNAREs hom	VTI1A	23	25.217	217	2.4317	1.3403	2	0.422555956
G5EA30;Q92879-4;Q92879;	3;3;3;3;3;3;1;1;1;1;1;	CUGBP Elav-like family member 1	CELF1	6.4	55.142	514	3.2792	1.34	3	0.422233001
Q9BW92;Q9H9V2;Q5T5E9;(	17;12;9;3;3	Threonine--tRNA ligase, mitochondrial	TARS2	29.1	81.035	718	3.8966	1.3369	17	0.418891556
P08754;P09471;A8MTJ3;P1	12;3;2;2;2;1	Guanine nucleotide-binding protein G(k) subunit alpha	GNAI3	42.1	40.532	354	3.6976	1.3353	6	0.417163907
P62304;A6NHK2	5;3	Small nuclear ribonucleoprotein E	SNRPE	69.6	10.803	92	3.876	1.3352	16	0.41705586
J3KNX9;Q92614;Q92614-4;(	13;13;13;13;13;11;2	Unconventional myosin-XVIIIa	MYO18A	8.8	233.14	2054	3.5731	1.3332	5	0.414893223
P11387;Q969P6;Q969P6-2;I	11;1;1;1	DNA topoisomerase 1	TOP1	15	90.725	765	3.7826	1.3323	7	0.413918977
P41250;F8WCK4;H7C443	23;1;1	Glycine--tRNA ligase	GARS	37.8	83.165	739	3.7169	1.3291	18	0.410449655
B4E040;Q6ZS74;P11234;C9J	7;7;7;5;4;1;1	Ras-related protein Ral-B	RALB	28.1	25.966	228	3.3625	1.3288	10	0.410123978
E9PAV3;F8VU71;Q13765;H(	4;4;4;4;4;4;3;3;1;1	Nascent polypeptide-associated complex subunit alpha	NACA	2.6	205.42	2078	3.4346	1.3269	6	0.408059648
Q9Y6A4;H3BPA3	5;1	UPF0468 protein C16orf80	C16orf80	21.2	22.774	193	2.0304	1.3254	7	0.406427825
Q99460;Q99460-2;H7C378;(	36;36;10;8;7;1	26S proteasome non-ATPase regulatory subunit 1	PSMD1	52.3	105.84	953	4.0011	1.3249	34	0.405883473
P49720;J3KRR2;J3KSM3	10;7;7	Proteasome subunit beta type-3	PSMB3	55.6	22.949	205	3.3568	1.3234	20	0.404249185
B4DVB8;Q15717;B1AM49;E	14;14;1;1;1;1;1;1;1;1;	ELAV-like protein 1	ELAVL1	50.7	38.996	353	3.9031	1.3221	22	0.402831302
G8JLB6;P31943;E9PCY7;D6F	15;15;14;9;9;8;8;7;7;7	Heterogeneous nuclear ribonucleoprotein H;Heterogene	HNRNPH1	47.7	51.229	472	2.9945	1.3219	58	0.402613043
Q12959-2;Q12959-7;Q1295	12;12;12;12;12;12;12	Disks large homolog 1	DLG1	18.4	103.32	926	3.5265	1.3206	8	0.401193551
Q8WXC9;E7ESZ7;O95299;C	3;3;3;2;2	NADH dehydrogenase [ubiquinone] 1 alpha subcomplex	NDUFA10	11.2	48.562	429	3.7586	1.3197	3	0.400210007
P52294;C9JYI4;C9J4U1;F2Z3	6;3;1;1;1;1	Importin subunit alpha-1	KPNA1	16.5	60.221	538	2.7164	1.3191	7	0.399553938
P28074;E9PAV2;P28074-2;I	14;10;6;6	Proteasome subunit beta type-5	PSMB5	57.8	28.48	263	3.8714	1.3186	26	0.399006986
P68400;E7EU96;P68400-2	15;15;10	Casein kinase II subunit alpha	CSNK2A1	43.7	45.143	391	3.0889	1.3177	20	0.39802195
P34897;P34897-3;P34897-2	22;22;21;15;13;11;9;8	Serine hydroxymethyltransferase, mitochondrial;Serine	SHMT2	59.5	55.992	504	3.8323	1.3176	24	0.39791246
Q9NQG5;A2A2M0;E9PIQ9;(	11;4;4;2;2	Regulation of nuclear pre-mRNA domain-containing prot	RPRD1B	37.4	36.899	326	3.3089	1.3175	12	0.397802962
Q9NUP9;G3V1D4	6;6	Protein lin-7 homolog C	LIN7C	33.5	21.834	197	4.0016	1.3157	7	0.39583057
O00165-2;O00165;O00165-	4;4;4;3;2;2;2;1	HCLS1-associated protein X-1	HAX1	13.6	32.418	287	2.7605	1.3155	4	0.395611249
Q9NRP2;H3BPP2;H3BTB6;H	3;2;1;1;1;1	COX assembly mitochondrial protein 2 homolog	CMC2	26.6	9.4598	79	3.5225	1.3153	3	0.395391894
P62314;J3QLI9;J3QLR7	5;3;1	Small nuclear ribonucleoprotein Sm D1	SNRPD1	54.6	13.281	119	4.1649	1.3149	23	0.394953085
O43852-3;O43852;O43852-	16;16;12;12;11;9;7;6	Calumenin	CALU	55.1	38.05	323	3.0652	1.3136	12	0.393526032
Q13619;Q13619-2;A6NE76	11;10;2	Cullin-4A	CUL4A	16.9	87.679	759	3.1537	1.3129	10	0.392757034
Q86WA6;Q86WA6-2;B4DM	6;6;2;2;1	Valacyclovir hydrolase	BPHL	33.7	32.542	291	2.7802	1.3118	2	0.39154778
Q15166;F8WD41;C9JZ99	2;1;1	Serum paraoxonase/lactonase 3	PON3	6.2	39.607	354	2.659	1.3112	2	0.39088776
Q9Y6D9;B3KR41;C9JP81;C9	13;13;4;4;4;2;2;1;1	Mitotic spindle assembly checkpoint protein MAD1	MAD1L1	22.8	83.066	718	3.8538	1.3098	13	0.389346536
P38435;E9PEE1;F8WDU9;F8	10;8;3;1;1	Vitamin K-dependent gamma-carboxylase	GGCX	15.3	87.56	758	4.0784	1.3091	11	0.388575307
Q49B96;F8WEL9	4;2	Cytochrome c oxidase assembly protein COX19	COX19	46.7	10.394	90	2.8496	1.309	4	0.388465097
Q8IWJ2;B8ZZW2;B3KR21;B	15;7;3;2;1;1	GRIP and coiled-coil domain-containing protein 2	GCC2	9.3	195.91	1684	2.7726	1.3089	8	0.38835488
P04792;F8WE04;C9J3N8	11;6;2	Heat shock protein beta-1	HSPB1	51.2	22.782	205	3.3423	1.3086	18	0.388024176
Q5TFE4;Q5QPD0;H0YDA5	5;2;1	5-nucleotidase domain-containing protein 1	NT5DC1	14.9	51.844	455	2.3136	1.3083	5	0.387693396
P08579	11	U2 small nuclear ribonucleoprotein B	SNRPB2	50.2	25.486	225	3.4874	1.3078	15	0.387141928
Q9NP73;D6RE84;Q9NP73-2	4;4;4	UDP-N-acetylglucosamine transferase subunit ALG13 hor	ALG13	4	126.05	1137	3.4467	1.3069	4	0.386148755
G3V1Z5;P46379-3;P46379;E	20;20;20;20;20;18;18	Large proline-rich protein BAG6	BAG6;BAT3	25.5	122.35	1162	4.1421	1.3069	18	0.386148755
P12830;Q9UII8;H3BNC6;H3	13;12;7;5;3;2;2;1	Cadherin-1;E-Cad/CTF1;E-Cad/CTF2;E-Cad/CTF3	CDH1	18.5	97.455	882	4.5661	1.3051	22	0.384160354
B4DNJ6;Q9Y3F4;B0AZV0;H(	6;6;3;2	Serine-threonine kinase receptor-associated protein	STRAP	21.5	39.777	363	2.8147	1.305	6	0.384049807
I1E4Y6;Q6Y7W6-3;Q6Y7W6	4;4;4;4;3;2;2;1;1;1;1;	PERQ amino acid-rich with GYF domain-containing protei	GIGYF2	4.2	152.53	1321	3.1635	1.304	3	0.38294387
P13995;B9A062;B8ZZU9;Q7	5;3;2;2;1	Bifunctional methylenetetrahydrofolate dehydrogenase	MTHFD2	31.4	37.895	350	3.2699	1.3039	5	0.382833229
Q08211;Q08211-2	64;11	ATP-dependent RNA helicase A	DHX9	62.7	140.96	1270	4.4604	1.3017	131	0.380396992
Q9UI26-2;Q9UI26;F8WDV0	2;2;2;1;1	Importin-11	IPO11	4.2	117.01	1015	1.7816	1.3016	2	0.380286156
Q9GZS3;H0YN81;H0YMF9;H	8;8;7;6;3;2;1	WD repeat-containing protein 61	WDR61	38.7	33.58	305	3.218	1.3013	8	0.379953597
P55072;C9IZA5;C9JUP7;E7E	45;8;7;1	Transitional endoplasmic reticulum ATPase	VCP	71.6	89.321	806	4.638	1.3009	58	0.379510067
P35998;B7Z5E2;C9JLS9	23;14;8	26S protease regulatory subunit 7	PSMC2	58	48.633	433	3.6981	1.3006	22	0.379177329
Q03001;F8W9J4;E7ERU0;E9	18;18;18;17;17;16;16	Dystonin	DST	2.6	860.65	7570	2.6266	1.2988	11	0.37717929
P45974;P45974-2;Q92995;C	11;11;1;1;1;1	Ubiquitin carboxyl-terminal hydrolase 5	USP5	18.1	95.785	858	3.1692	1.2985	11	0.376846014
P25788;P25788-2;G3V4X5;H	11;11;6;2;2;2	Proteasome subunit alpha type-3	PSMA3	45.5	28.433	255	3.512	1.298	21	0.376290383
Q13418;B7Z418;B7Z1I0	2;2;2	Integrin-linked protein kinase	ILK	7.5	51.419	452	1.9026	1.2956	2	0.373620373
Q8WVY7	3	Ubiquitin-like domain-containing CTD phosphatase 1	UBLCP1	11.9	36.804	318	2.0399	1.2956	3	0.373620373
P62979;P0CG48;F5H041;F5I	10;6;6;6;6;6;6;6;6;6	Ubiquitin-40S ribosomal protein S27a;Ubiquitin;40S ribos	RPS27A;UBC;UBB;	58.3	17.965	156	4.2621	1.2953	51	0.373286274
Q92887;O15440;Q86UX3	4;1;1	Canalicular multispecific organic anion transporter 1	ABCC2	2.8	174.21	1545	2.33	1.2947	2	0.372617844
Q6DN03;Q6DRA6	3;3	Putative histone H2B type 2-C;Putative histone H2B type	HIST2H2BC;HIST2I	13	21.472	193	3.1246	1.2939	2	0.371726122
J3KQ48;Q9Y3E5	6;6	Peptidyl-tRNA hydrolase 2, mitochondrial	PTRH2	51.7	19.325	180	3.3529	1.2927	8	0.370387504
Q9UHD8-5;Q9UHD8;Q9UHI	17;16;16;16;13;10;10	Septin-9	41526	38.5	64.681	579	3.6053	1.2926	31	0.370275897
P23470;P23470-2	9;9	Receptor-type tyrosine-protein phosphatase gamma	PTPRG	7.5	162	1445	3.3192	1.2923	9	0.369941022
Q13363;Q13363-2;D6RAX2;	8;8;6;5;5;5;4;2;1;1;1;	C-terminal-binding protein 1	CTBP1	26.8	47.535	440	3.6275	1.2918	7	0.369382725
B1AHD1;P55769	10;10	NHP2-like protein 1	NHP2L1	66.7	14.627	132	3.0187	1.2914	12	0.368935932
F5H619;Q86XA9;Q86XA9-2	3;3;3;2;2;2;1	HEAT repeat-containing protein 5A	HEATR5A	2	222.7	2046	2.0698	1.2907	2	0.368153711
P09525;Q6P452;B4DDF9	15;14;11	Annexin A4;Annexin	ANXA4	60.2	35.882	319	3.4796	1.2897	14	0.367035516
Q15020;B7ZKM0;F8VV04;Q	30;29;21;8;5;4;4;4;1	Squamous cell carcinoma antigen recognized by T-cells 3	SART3	36.7	109.93	963	3.0468	1.2883	26	0.365468586
Q92504;F5H0B5;E7ESG6	2;2;2	Zinc transporter SLC39A7	SLC39A7	8.5	50.117	469	2.5675	1.2873	3	0.364348307
P11388-4;P11388-3;P11388	15;15;15;15;1	DNA topoisomerase 2-alpha	TOP2A	10.3	182.68	1612	3.0701	1.2844	8	0.36109457
Q12907;D6RBV2;D6RDX1;D	11;10;9;8;3	Vesicular integral-membrane protein VIP36	LMAN2	43	40.228	356	3.7712	1.2839	10	0.360532839
Q9H490;E7EVL4;Q9H490-2	2;2;2	Phosphatidylinositol glycan anchor biosynthesis class U p	PIGU	6.4	50.051	435	2.9609	1.283	2	0.35952117
P0C0S5;Q71UI9;C9J0D1;Q7	5;5;4;4;4;3;2;1	Histone H2A.Z;Histone H2A.V;Histone H2A	H2AFZ;H2AFV	53.9	13.553	128	3.2492	1.2826	13	0.359071312
O14966;O14966-2;C9JE77;E	4;2;1;1	Ras-related protein Rab-7L1	RAB7L1	20.7	23.155	203	2.5143	1.2818	3	0.358171175
Q8WX92	6	Negative elongation factor B	COBRA1	13.3	65.697	580	3.0447	1.2817	6	0.358058618
P18827;H7C1K4;E9PHH3	3;1;1	Syndecan-1	SDC1	11.9	32.461	310	4.0951	1.2815	3	0.357833479
P50502;Q8IZP2;H7C3I1;Q8I	5;4;4;3;3;1	Hsc70-interacting protein;Putative protein FAM10A4;Put	ST13;ST13P4;ST13	16.5	41.331	369	3.3647	1.2803	5	0.356481902
P48426;B4DGX2;H7BXS3;P7	3;3;2;1;1	Phosphatidylinositol 5-phosphate 4-kinase type-2 alpha	PIP4K2A	8.1	46.224	406	2.433	1.28	2	0.35614381
K7ELJ7;P04632;K7EM73;K7I	4;4;4;3;2;1;1;1;1	Calpain small subunit 1	CAPNS1	20.9	29.44	278	2.7353	1.2796	5	0.355692898
P62258;P62258-2;K7EM20;I	17;14;7;6;6;5;1	14-3-3 protein epsilon	YWHAE	59.6	29.174	255	3.1828	1.2792	23	0.355241844
P25786-2;P25786;F5GX11;E	17;17;15;9;1	Proteasome subunit alpha type-1;Proteasome subunit al	PSMA1	66.9	30.239	269	3.5569	1.2791	17	0.355129058
Q9BZZ5;G3V1C3;Q9BZZ5-2;	11;11;11;10;9;8;7;4;4	Apoptosis inhibitor 5	API5	29.6	59.004	524	3.4534	1.2787	10	0.354677829
A8MW61;O43660;O43660-2	4;4;4;2;2;1	Pleiotropic regulator 1	PLRG1	9.3	57.181	514	3.5374	1.2769	3	0.352645545
Q9H3U1;Q9H3U1-2	3;3	Protein unc-45 homolog A	UNC45A	3.8	103.08	944	2.1577	1.2738	3	0.349138777
Q96HV5;F8WDE2;H7BXL1	2;1;1	Transmembrane protein 41A	TMEM41A	10.6	29.665	264	3.8121	1.2733	2	0.34857237
Q7L523;Q5VZM2;Q5VZM2-	3;2;2;1	Ras-related GTP-binding protein A;Ras-related GTP-bindi	RRAGA;RRAGB	11.8	36.566	313	3.3098	1.2716	3	0.346644922
Q96F25	2	UDP-N-acetylglucosamine transferase subunit ALG14 hor	ALG14	9.3	24.15	216	3.4605	1.2716	2	0.346644922
O15269;O15269-2	14;4	Serine palmitoyltransferase 1	SPTLC1	36.6	52.743	473	3.2574	1.2716	14	0.346644922

Q9H223;Q9NZN3;Q9H4M9;	5;1;1;1;1;1	EH domain-containing protein 4	EHD4	9.6	61.174	541	2.9683	1.2696	3	0.344374033
Q9NYY8;Q9NYY8-2	3;3	FAST kinase domain-containing protein 2	FASTKD2	6.3	81.462	710	2.7394	1.2686	3	0.343237247
P13804;P13804-2;H0YLU7;	16;15;14;12;12;11;10	Electron transfer flavoprotein subunit alpha, mitochondr	ETFA	68.2	35.079	333	3.4344	1.2682	16	0.342782282
P52298;P52298-2;E9PAR5;	5;4;4;4;4;1	Nuclear cap-binding protein subunit 2	NCBP2	31.4	18.001	156	3.0872	1.2671	3	0.341530387
P18564;E9PEE8;C9JA97	1;1;1	Integrin beta-6;Integrin beta	ITGB6	1.1	85.935	788	5.0247	1.2665	2	0.340847077
P34932	36	Heat shock 70 kDa protein 4	HSPA4	51.5	94.33	840	3.9977	1.2662	36	0.340505301
O95297;O95297-2;O95297-	2;2;2;1	Myelin protein zero-like protein 1	MPZL1	7.8	29.082	269	2.8203	1.2659	2	0.340163443
Q13427;E9PG73;Q13427-2;	2;2;2;2;2;2	Peptidyl-prolyl cis-trans isomerase G;Peptidyl-prolyl cis-	PPIG	3.4	88.616	754	2.7136	1.2654	3	0.339593501
Q8IXH7;Q8IXH7-4	3;3	Negative elongation factor C/D	TH1L	6.9	66.246	590	2.0319	1.265	3	0.339137385
O15254;O15254-2;D6RJ89	19;16;2	Peroxisomal acyl-coenzyme A oxidase 3	ACOX3	39.6	77.628	700	2.9703	1.265	22	0.339137385
H3BQK9;H3BPE1;F8W8Q1;	41;41;40;40;40;38;38	Microtubule-actin cross-linking factor 1, isoforms 1/2/3/	MACF1	6.6	861.07	7592	2.7468	1.2638	28	0.337768171
Q8WWC4;H7C0V0	7;4	Uncharacterized protein C2orf47, mitochondrial	C2orf47	26.5	32.544	291	3.1564	1.2637	7	0.337654011
P78556;P78556-2	3;3	C-C motif chemokine 20;CCL20(1-67);CCL20(1-64);CCL20(	CCL20	33.3	10.762	96	2.2848	1.2604	6	0.333881659
Q14318-2;J3KQ73;Q14318;	5;4;4;3	Peptidyl-prolyl cis-trans isomerase FKBP8	FKBP8	13.8	44.648	413	3.385	1.2592	5	0.332507446
Q15075	64	Early endosome antigen 1	EEA1	48.3	162.46	1411	4.107	1.2584	63	0.331590576
Q8N4Q1-2;Q8N4Q1	4;4	Mitochondrial intermembrane space import and assemb	CHCHD4	31.6	17.385	155	3.0997	1.2572	4	0.330214177
P35659;B4DFG0;P35659-2;	8;7;7;5;4;3;2	Protein DEK	DEK	23.7	42.674	375	2.9086	1.2572	10	0.330214177
Q7L576;Q7L576-2;Q7L576-	22;15;12;9;9;9;9;9;8;	Cytoplasmic FMR1-interacting protein 1	CYFIP1	21.1	145.18	1253	2.6576	1.255	23	0.327687364
Q15287;H3BTC0;H3BMM9;	2;2;2;2;2;2;2;1;1	RNA-binding protein with serine-rich domain 1	RNPS1	8.9	34.208	305	2.5173	1.2545	2	0.327112471
O00151	12	PDZ and LIM domain protein 1	PDLIM1	51.1	36.071	329	2.5629	1.254	7	0.326537348
Q13148-2;Q13148;G3V162;	7;7;6;6;4;4;4;3;1;1	TAR DNA-binding protein 43	TARDBP	26.4	44.991	416	3.8414	1.254	11	0.326537348
Q6NUM9;G5E9N3;Q6NUM	10;9;6;6;5;4	All-trans-retinol 13,14-reductase	RETSAT	20.7	66.819	610	3.2696	1.252	7	0.324234562
P48729-2;P48729;Q71TU5;	2;2;2;2;2;1;1	Casein kinase I isoform alpha;Casein kinase I isoform alp	CSNK1A1;CSNK1A	5.2	41.937	365	2.391	1.2515	2	0.323658291
P13797;B4DGB4;F8W8D8;B	4;4;4;4;2;1	Plastin-3	PLS3	6.7	70.81	630	2.7225	1.251	4	0.32308179
Q7L1Q6-3;Q7L1Q6-4;Q7L1	3;3;3;2;2;1;1;1;1;1	Basic leucine zipper and W2 domain-containing protein 1	BZW1	11.3	51.281	451	1.7537	1.2491	2	0.32088898
F8VPJ2;Q9NQT8;E7ERX9;H	8;8;6;3;3;3;3;3;3;1	Kinesin-like protein KIF13B	KIF13B	5.2	202.79	1826	2.7401	1.2491	6	0.32088898
Q99487;Q5SY00;Q5SY01	2;1;1	Platelet-activating factor acetylhydrolase 2, cytoplasmic	PAFAH2	6.9	44.035	392	2.2717	1.2478	2	0.319386715
O95477	3	ATP-binding cassette sub-family A member 1	ABCA1	1.4	254.3	2261	2.8633	1.2476	3	0.319155458
Q9Y673;Q9Y673-2;B4DR67	11;10;7	Dolichyl-phosphate beta-glucosyltransferase	ALG5	46	36.946	324	3.0172	1.2476	9	0.319155458
P78347;P78347-3;P78347-4	44;44;44;44;12;2	General transcription factor II-I	GTF2I	48.8	112.42	998	3.8037	1.2469	64	0.318345767
F5HFY4;Q99733;C9JZI7;C9J	7;7;6;6;5;4;4;4;4;3;	Nucleosome assembly protein 1-like 4	NAP1L4b;NAP1L4	21.5	44.078	386	3.3694	1.2467	7	0.318114344
J3KPX7;Q99623;F5GY37;B4	15;15;14;12;11;11;10	Prohibitin-2	PHB2	57.5	33.402	299	3.676	1.2458	17	0.317072478
Q15477;B4DM01;F8VNU1;	4;4;4;4;4;4;1;1	Helicase SKI2W	SKIV2L	4.1	137.75	1246	2.2921	1.2455	2	0.316725022
Q8IXB1;Q8IXB1-2;Q3B7W8	15;14;6;5;1	DnaJ homolog subfamily C member 10	DNAJC10	24.7	91.079	793	3.973	1.2445	13	0.31556623
P82673;P82673-2;H0YG82	5;3;2	28S ribosomal protein S35, mitochondrial	MRPS35	21.4	36.844	323	3.3348	1.2427	5	0.313478058
P55036;Q5VWC4;P55036-2	9;8;6;6;4;3;1	26S proteasome non-ATPase regulatory subunit 4	PSMD4	30.8	40.736	377	3.8073	1.2419	8	0.31254901
P24534;C9JZW3;F2Z2G2;F8	8;4;2;1	Elongation factor 1-beta	EEF1B2	54.7	24.763	225	4.1456	1.2416	9	0.312200463
Q8WW12;Q8WW12-3	1;1	PEST proteolytic signal-containing nuclear protein	PCNP	7.3	18.925	178	1.7401	1.2414	2	0.311968051
Q9BTL3	4	RNMT-activating mini protein	FAM103A1	37.3	14.381	118	2.1856	1.2412	2	0.311735602
P63151-2;Q66LE6;P63151;C	2;2;2;1;1;1;1;1;1;1;1	Serine/threonine-protein phosphatase 2A 55 kDa regulat	PPP2R2D;PPP2R2	5.5	52.999	457	1.7448	1.2394	2	0.309641873
Q9NVH6-8;Q9NVH6;Q9NV	3;3;3;2;2;2;2;2	Trimethyllysine dioxygenase, mitochondrial	TMLHE	7.2	50.832	432	1.7176	1.238	2	0.308011315
Q9BRJ7;K7EIN2;K7ENA3;Q	4;4;3;2;1;1	Protein syndesmos	NUDT16L1	23.7	23.338	211	2.2052	1.238	3	0.308011315
Q14677-3;Q14677;Q14677-	1;1;1	Clathrin interactor 1	CLINT1	1.6	70.294	643	1.8328	1.2373	2	0.307195344
Q92620;B4DVG8;J3KRT1;H	7;3;2;2;2;1	Pre-mRNA-splicing factor ATP-dependent RNA helicase	DHX38	7.5	140.5	1227	2.481	1.2367	9	0.306495572
O00186	8	Syntaxin-binding protein 3	STXBP3	15.9	67.764	592	2.2981	1.2348	8	0.304277388
Q15031;E9PHM2;C9IZR7	9;9;1	Probable leucine--tRNA ligase, mitochondrial	LARS2	13.4	101.97	903	2.2758	1.2335	6	0.302757716
Q92947;Q92947-2;B4DK85;	2;2;2;1	Glutaryl-CoA dehydrogenase, mitochondrial	GCDH	7.8	48.127	438	1.9131	1.2306	2	0.299361898
Q96HY6;Q96HY6-2	5;4	DDRGK domain-containing protein 1	DDRGK1	30.9	35.61	314	3.3136	1.2304	5	0.299127408
O14818;O14818-2;H0Y586;	13;8;7;6;5;5;5;2	Proteasome subunit alpha type-7	PSMA7	63.7	27.887	248	3.3965	1.2294	25	0.29795439
Q13616	7	Cullin-1	CUL1	9.9	89.677	776	3.1532	1.2256	7	0.293488202
Q96A72;A6NEC0;F5H6P7;F	11;7;7;7;3;1	Protein mago nashi homolog 2	MAGOHB	76.4	17.276	148	2.6069	1.2254	12	0.293252756
O76094;G5E9Z8;D6RDY6	7;5;4	Signal recognition particle 72 kDa protein	SRP72	14.2	74.605	671	2.6094	1.2249	10	0.292663973
P35232;C9JW96;E9PCW0;E	13;11;9;9;9;6;5	Prohibitin	PHB	72.4	29.804	272	3.4422	1.2234	17	0.290896181
Q5LJA9;Q5LJA5;Q9Y5K5;Q	3;3;3;3;3;3;2;2;2;2;2;	Ubiquitin carboxyl-terminal hydrolase isozyme L5	UCHL5	8.2	41.694	368	2.6196	1.2233	3	0.290778251
REV__Q9BZ29-4;REV__Q9	2;2;2;2;1;1;1;1			1.2	238.84	2093	1.887	1.2227	2	0.29007047
Q99943;Q9HBM8	4;4	1-acyl-sn-glycerol-3-phosphate acyltransferase alpha	AGPAT1	21.2	31.716	283	3.7644	1.2215	5	0.288653864
Q16706	8	Alpha-mannosidase 2	MAN2A1	8.5	131.14	1144	2.0312	1.2211	8	0.288181352
Q9HCS7;F5H315	4;4	Pre-mRNA-splicing factor SYF1	XAB2	4.2	100.01	855	3.4077	1.2211	3	0.288181352
Q96P70	6	Importin-9	IPO9	11.5	115.96	1041	3.4658	1.2205	5	0.287472295
O43617;A6NDN0;A6NKE1	2;2;2	Trafficking protein particle complex subunit 3	TRAPPC3	8.9	20.274	180	2.657	1.2189	2	0.28557977
Q3ZCQ8-2;Q3ZCQ8;Q330K	8;8;5	Mitochondrial import inner membrane translocase subu	TIMM50	22.1	50.464	456	3.551	1.2182	8	0.28475101
Q6NXG1;Q6NXG1-3;Q6NX	2;2;2;2;2;1;1	Epithelial splicing regulatory protein 1	ESRP1	4.3	75.585	681	2.3512	1.2172	2	0.283566239
P11279;B4DWL3	5;4	Lysosome-associated membrane glycoprotein 1	LAMP1	12.2	44.882	417	4.2794	1.2164	7	0.282617721
Q9UNS2;Q9UNS2-2;H7C3P	4;3;3;3;2;2;1;1;1	COP9 signalosome complex subunit 3	COPS3	13	47.873	423	2.3785	1.2162	4	0.282380495
P11142;E9PKE3;P11142-2;E	34;33;25;24;19;18;14	Heat shock cognate 71 kDa protein	HSPA8	55.1	70.897	646	4.0045	1.216	96	0.282143229
Q99598;Q5VVQ1	6;3	Translin-associated protein X	TSNAX	29.7	33.112	290	2.8146	1.2146	3	0.280481274
Q9H9Q2;J3KQ41;Q9H9Q2-	3;2;2;2;2;2;1;1;1;1;1;	COP9 signalosome complex subunit 7b	COPS7B	19.3	29.622	264	2.5167	1.2146	4	0.280481274
P05026;P05026-2;A6NGH2;	9;9;8;8	Sodium/potassium-transporting ATPase subunit beta-1	ATP1B1	39.6	35.061	303	3.5285	1.2128	9	0.278341659
A5YKK6;A5YKK6-2;A5YKK6-	27;27;23;20;13;9;6;1;	CCR4-NOT transcription complex subunit 1	CNOT1	14.4	266.94	2376	2.6195	1.2125	23	0.277984747
Q14696;B4DW84;H0YLI4	11;1;1	LDLR chaperone MESD	MESDC2	50.4	26.076	234	3.0593	1.2115	16	0.276794405
P55210-2;Q5SVL1	2;1		CASP7	6.3	28.03	253	3.8645	1.2112	3	0.27643711
P13473-3;P13473;P13473-2	3;3;3;3;3;1	Lysosome-associated membrane glycoprotein 2	LAMP2	7.1	45.17	411	2.319	1.2104	4	0.275483893
O75083;O75083-3;D6RD66	7;4;3	WD repeat-containing protein 1	WDR1	17.3	66.193	606	3.0977	1.2098	6	0.274768566
Q96SI9;Q96SI9-2	5;5	Spermatid perinuclear RNA-binding protein	STRBP	7.7	73.652	672	2.9079	1.2076	2	0.272142662
P52597	14	Heterogeneous nuclear ribonucleoprotein F;Heterogene	HNRNPF	48.4	45.671	415	3.6516	1.2074	24	0.271903706
Q96QC0;F5H5K4	4;3	Serine/threonine-protein phosphatase 1 regulatory sub	PPP1R10	6.2	99.057	940	1.7178	1.2074	5	0.271903706
O60306;H0YH15;H0YLN9	30;11;2	Intron-binding protein aquarius	AQR	26.7	171.29	1485	3.3507	1.2058	29	0.269990634
Q92805;H0YE54	3;1	Golgin subfamily A member 1	GOLGA1	5.7	88.183	767	1.7932	1.2043	2	0.268194823
Q12931;F5H897;I3L0K7;I3L	14;14;11;3;2;1	Heat shock protein 75 kDa, mitochondrial	TRAP1	27.1	80.109	704	2.8046	1.2041	11	0.267955212
Q15459;E9PAW1;H7C1L2;F	31;26;5;3;1	Splicing factor 3A subunit 1	SF3A1	45.3	88.885	793	4.0819	1.2041	36	0.267955212
Q16543;K7EL68;K7EQA9;K7	6;4;3;2;1	Hsp90 co-chaperone Cdc37	CDC37	20.9	44.468	378	2.3871	1.204	5	0.267835392
P62750;A8MUS3;K7ERT8;K	11;9;9;9;9;6	60S ribosomal protein L23a	RPL23A	51.3	17.695	156	2.4222	1.2036	15	0.267356012
J3KPA3;Q96SU4-6;B1AKJ6;	2;2;2;2;2;2;2;2;1;1;1;	Oxysterol-binding protein;Oxysterol-binding protein-rel	OSBPL9	3.6	85.182	754	1.8219	1.2033	2	0.266996372
P23284	15	Peptidyl-prolyl cis-trans isomerase B	PPIB	56	23.742	216	3.589	1.2022	39	0.265676925
O00425;O00425-2;F8WD15	18;8;3	Insulin-like growth factor 2 mRNA-binding protein 3	IGF2BP3	37.3	63.704	579	3.5231	1.2022	25	0.265676925
P09429;Q5T7C6;Q5T7C4;B2	11;10;10;6;6;2	High mobility group protein B1;Putative high mobility gr	HMGB1;HMGB1P	51.2	24.893	215	2.9836	1.2012	24	0.26447638
P14324;E9PCI9	5;5	Farnesyl pyrophosphate synthase	FDPS	12.4	48.275	419	3.2142	1.1988	3	0.261590989
P09651;F8W6I7;P09651-2;F	22;22;22;22;22;22;21	Heterogeneous nuclear ribonucleoprotein A1;Heterogen	HNRNPA1;HNRNF	48.4	38.746	372	3.1484	1.1977	83	0.260266587
J3KQL0;P31327;P31327-2;E	22;22;15;1;1	Carbamoyl-phosphate synthase [ammonia], mitochondri	CPS1	19.5	165.65	1506	2.2754	1.1975	3	0.260025656
Q9H6E4	3	Coiled-coil domain-containing protein 134	CCDC134	21.8	26.56	229	2.5955	1.1962	2	0.258458623
E7EWR4;P33240;P33240-2;	6;6;6;5	Cleavage stimulation factor subunit 2	CSTF2	13.9	62.942	597	1.9912	1.1961	5	0.258338011
P62877	3	E3 ubiquitin-protein ligase RBX1	RBX1	25	12.274	108	2.8317	1.1949	2	0.256889886
E9PFW3;Q96CW1;Q96CW1	9;9;9;6;4;4;3;3;1	AP-2 complex subunit mu	AP2M1	22.6	52.303	460	2.839	1.1948	8	0.256769143
Q6KC79;Q6KC79-2	2;2	Nipped-B-like protein	NIPBL	1	316.05	2804	1.8497	1.1946	2	0.256527627
Q2TAY7;B4E3L0	5;2	WD40 repeat-containing protein SMU1	SMU1	9	57.543	513	3.0631	1.1929	5	0.254473108
P25787;C9JCK5;Q6MZI6;H3	12;6;2;2;1	Proteasome subunit alpha type-2	PSMA2	53.4	25.898	234	3.3929	1.191	20	0.252173413
P11717	89	Cation-independent mannose-6-phosphate receptor	IGF2R	44.4	274.37	2491	2.6781	1.19	92	0.250961574
Q5T2W1;Q5T2W1-2;A8MU	5;5;3;1	Na(+)/H(+) exchange regulatory cofactor NHE-RF3;Putati	PDZK1;PDZK1P1	11.9	57.129	519	2.4876	1.1898	5	0.250719083
G3V5Z7;P60900;B4DXJ9;G3	11;11;9;9;7;6;4;2;1;1	Proteasome subunit alpha type;Proteasome subunit alp	PSMA6	44	28.147	252	3.7085	1.1897	22	0.250597823
J3KN36;Q5JPE7;Q15155;P6	37;37;37;37;37;33;33	Nodal modulator 2;Nodal modulator 1;Nodal modulator	NOMO2;NOMO1;	42.6	139.38	1267	3.4512	1.1888	42	0.249506021
P49257	10	Protein ERGIC-53	LMAN1	33.1	57.548	510	3.4536	1.1883	11	0.248899107
Q8WUM0;F5H5C2;Q5T8N1	5;5;4	Nuclear pore complex protein Nup133	NUP133	6.4	128.98	1156	2.479	1.1877	7	0.248170473
Q9Y2S0;Q9Y2S0-2	4;1	DNA-directed RNA polymerases I and III subunit RPAC2	POLR1D	42.9	15.237	133	2.5691	1.1873	3	0.247684513
P23527;P33778;Q16778;P0	7;7;7;6;6;4	Histone H2B type 1-O;Histone H2B type 1-B;Histone H2B	HIST1H2BO;HIST1	54.8	13.906	126	3.3781	1.1871	4	0.247441471
Q9NWU2	2	Protein C20orf11	C20orf11	14.9	26.748	228	2.6916	1.1869	3	0.247198389
B7ZLA8;Q9C0E8;Q9C0E8-2;	4;4;4;4;4	Protein lunapark	KIAA1715;LNP	9.4	50.848	459	2.5697	1.1867	4	0.246955265
Q6P2Q9;I3L0J9;I3L3Z8;I3L1	116;43;12;2	Pre-mRNA-processing-splicing factor 8	PRPF8	60.4	273.6	2335	3.1393	1.1863	250	0.246468895
Q99497;K7ELW0;K7EN27	15;14;11	Protein DJ-1	PARK7	78.8	19.891	189	2.4024	1.1862	16	0.246347277
P17858;P17858-2;J3KNX3;C	22;21;2;2;2;2;1;1;1	6-phosphofructokinase, liver type	PFKL	41.3	85.018	780	3.6436	1.1857	22	0.245739032
P28072;I3L3X7	6;2	Proteasome subunit beta type-6	PSMB6	37.7	25.357	239	3.8568	1.1849	5	0.244765308
Q6DKK2;J3QT19;K7EKH7;K	5;3;1;1	Tetratricopeptide repeat protein 19, mitochondrial	TTC19	12.6	42.456	380	3.2433	1.1845	3	0.244278199
P12004	9	Proliferating cell nuclear antigen	PCNA	34.5	28.768	261	3.1581	1.1834	10	0.2429378
Q96AQ6;Q96AQ6-2;F5H2F	6;6;6;6;4;1	Pre-B-cell leukemia transcription factor-interacting prot	PBXIP1	10.7	80.642	731	2.6969	1.1833	3	0.242815884
P36954;K7EKS1;K7EK49	4;4;1	DNA-directed RNA polymerase II subunit RPB9	POLR2I	45.6	14.523	125	3.7225	1.1831	3	0.242572021
Q96ER3;E9PRZ1;J3KND1;E9	3;2;2;1;1	Protein SAAL1	SAAL1	11.4	53.557	474	1.9639	1.1822	2	0.241474126
P61106	15	Ras-related protein Rab-14	RAB14	82.8	23.897	215	3.5146	1.1819	19	0.241107975
Q9UL25	12	Ras-related protein Rab-21	RAB21	59.6	24.347	225	3.3805	1.1814	24	0.240497517
O43684;O43684-2;J3QT28;	12;12;12;8;6	Mitotic checkpoint protein BUB3	BUB3	49.1	37.154	328	3.4221	1.1798	15	0.238542314
Q9H0T7;H7C1P7;C9J0T6;Q	6;2;2;2;1;1	Ras-related protein Rab-17	RAB17	39.6	23.491	212	2.1876	1.1786	5	0.237074171
Q9H845;H0Y8Z9;D6RCD8	13;9;1	Acyl-CoA dehydrogenase family member 9, mitochondri	ACAD9	30.8	68.76	621	3.1738	1.1774	13	0.235604533
Q14103;Q14103-3;H0Y8G5;	15;15;14;13;13;13;13	Heterogeneous nuclear ribonucleoprotein D0	HNRNPD	37.5	38.434	355	3.3415	1.1766	36	0.234623941
P53007;B4DP62	8;5	Tricarboxylate transport protein, mitochondrial	SLC25A1	35.4	34.012	311	3.0305	1.1744	11	0.231923873
Q7L2J0;Q7L2J0-2	2;2	7SK snRNA methylphosphate capping enzyme	MEPCE	2.8	74.354	689	3.111	1.1738	2	0.231186613
O95166;H6UMI1;Q9H0R8-2	2;2;1;1;1;1	Gamma-aminobutyric acid receptor-associated protein;	GABARAP;GABAR	23.9	13.918	117	2.6568	1.1735	3	0.230817842
O60506;O60506-2;O60506-	18;18;18;18;12;7	Heterogeneous nuclear ribonucleoprotein Q	SYNCRIP	33.7	69.602	623	3.1771	1.172	25	0.22897257
Q9Y5Q9;H7BZV8;Q9Y5Q9-2	4;2;1;1;1	General transcription factor 3C polypeptide 3	GTF3C3	5	101.27	886	1.7159	1.1716	4	0.228480098
P30085;Q5T0D2;E9PGI8	5;4;3	UMP-CMP kinase	CMPK1	31.1	22.222	196	1.5057	1.1701	5	0.226631832
O00264;B7Z1L3	13;9	Membrane-associated progesterone receptor componen	PGRMC1	62.1	21.671	195	3.2921	1.1698	13	0.226261894

P22626;P22626-2	25;24	Heterogeneous nuclear ribonucleoproteins A2/B1	HNRNPA2B1	69.4	37.429	353	3.1405	1.1697	74	0.226138561
Q8N0X4;Q8N0X4-2;B4DU6	3;3;3;2;2;1	Citrate lyase subunit beta-like protein, mitochondrial	CLYBL	12.4	37.359	340	2.7513	1.1682	3	0.22428729
B4DIY7;C9J2U4;Q96BW9;B4	3;3;2;1;1	Mitochondrial translocator assembly and maintenance p	TAMM41	8.2	51.066	452	2.2746	1.167	2	0.222804561
C9J2K4;Q16864	2;2	V-type proton ATPase subunit F	ATP6V1F	21.1	16.401	147	2.6023	1.1661	2	0.221691514
P21333;P21333-2;Q5HY54;I	130;130;129;45;28;16	Filamin-A	FLNA	66.5	280.74	2647	2.8488	1.1654	205	0.220825216
Q7Z4G4;Q7Z4G4-2;F2Z2Q4	3;3;2;2;2;2;1	tRNA (guanine(10)-N2)-methyltransferase homolog	TRMT11	5.6	53.42	463	3.8939	1.1651	2	0.220453786
O43681;K7ERW9	5;2	ATPase ASNA1	ASNA1	18.7	38.792	348	2.939	1.1644	4	0.219586745
P18031;B4DSN5	9;7	Tyrosine-protein phosphatase non-receptor type 1	PTPN1	27.8	49.966	435	2.4512	1.1638	8	0.218843151
P46926;D6R9P4;E7EVU7;D6	4;3;3;3;3;2;2;1;1;1;1	Glucosamine-6-phosphate isomerase 1	GNPDA1	23.5	32.668	289	2.6949	1.1634	3	0.218347209
Q6YHU6;C9JJB1;Q6YHU6-2;	2;2;2;2;1;1;1;1;1;1	Thyroid adenoma-associated protein	THADA;tmp_locu	1.5	219.6	1953	1.7603	1.1633	2	0.218223197
Q9Y4C2;Q9Y4C2-2	2;2	Protein FAM115A	FAM115A	2.7	102.12	921	1.8435	1.1621	2	0.21673422
P08240;E9PJS4	11;10	Signal recognition particle receptor subunit alpha	SRPR	21.6	69.81	638	2.3954	1.161	17	0.215367972
J3KR44;Q96FW1;F5GYJ8;F5	8;8;7;7;7;3;2	Ubiquitin thioesterase OTUB1	OTUB1	28.6	35.214	308	1.9423	1.1609	4	0.215243704
P28070	8	Proteasome subunit beta type-4	PSMB4	48.5	29.204	264	3.6567	1.1597	16	0.213751646
C9JSL2;Q5JRX3-2;Q5JRX3;E	14;14;14;12;2;2;1	Presequence protease, mitochondrial	PITRM1	18.8	117.51	1038	2.5038	1.1595	3	0.21350282
P49321-3;P49321;F5H3J2;P	17;17;15;8;7;6;6;6;4;4	Nuclear autoantigenic sperm protein	NASP	32	86.267	790	3.1125	1.1593	13	0.213253951
O15145;C9JZD1;F8VR50	8;3;2	Actin-related protein 2/3 complex subunit 3	ARPC3	55.6	20.546	178	2.9175	1.1578	8	0.211386062
P28066;B4E2V4	10;7	Proteasome subunit alpha type-5	PSMA5	52.3	26.411	241	3.4187	1.1576	22	0.211136827
Q00059;H7BYN3;A8MRB2	9;7;7	Transcription factor A, mitochondrial	TFAM	35	29.096	246	2.7165	1.1566	6	0.209890007
P18858;B4DTU4;F5GZ28	1;1;1	DNA ligase 1;DNA ligase	LIG1	1.5	101.73	919	1.6214	1.1552	2	0.208142647
P46939;Q5T097;H0Y337;Q5	132;26;7;5;2;2;2;1;1;1	Utrophin	UTRN	48.5	394.46	3433	2.7829	1.1543	173	0.207018226
P10319;P18465;H0Y5E5;H0	7;6;4;4;4	HLA class I histocompatibility antigen, B-58 alpha chain;H	HLA-B	24.3	40.337	362	2.1308	1.1542	2	0.206893236
Q70CQ2;Q70CQ2-2;Q70CQ	34;34;32;12;4	Ubiquitin carboxyl-terminal hydrolase 34	USP34	12.7	404.23	3546	2.2598	1.1532	28	0.205642742
P01111	7	GTPase NRas	NRAS	52.4	21.229	189	2.5797	1.1519	7	0.204015477
Q71DI3;Q16695;K7ES00;B4	7;6;5;5;5;4;3	Histone H3.2;Histone H3.1t;Histone H3;Histone H3.3C	HIST2H3A;HIST3H	48.5	15.388	136	2.8328	1.1508	23	0.202637126
Q93008;Q93008-1;O00507;	45;45;24;23;22	Probable ubiquitin carboxyl-terminal hydrolase FAF-X;Pr	USP9X;USP9Y	20.7	292.28	2570	2.5364	1.1498	37	0.201382936
Q12846;A8MXY0;C9JFM5;H	8;6;4;2	Syntaxin-4	STX4	36.4	34.18	297	2.7867	1.1468	9	0.19761381
Q5SRQ6;P67870;Q5SRQ3	11;11;9	Casein kinase II subunit beta	CSNK2B	68.8	26.925	234	2.7109	1.1466	15	0.197362184
Q01518;Q01518-2;Q5T0R1;	18;17;7;7;7;7;7;7;7;6;	Adenylyl cyclase-associated protein 1	CAP1	39.8	51.901	475	2.8433	1.1463	18	0.196984664
P13010;C9JZ81;H7C0H9	40;7;2	X-ray repair cross-complementing protein 5	XRCC5	63.4	82.704	732	4.0037	1.1456	70	0.196103398
P42126;H3BP91;P42126-2;(	11;11;10;10;9	Enoyl-CoA delta isomerase 1, mitochondrial	ECI1;DCI	53	32.816	302	2.8196	1.1447	14	0.19496955
P05783;F8VZY9;CON__H-IN	29;27;3;2;2;2;2	Keratin, type I cytoskeletal 18	KRT18	68.1	48.057	430	3.5986	1.1441	85	0.194213156
Q13190;Q13190-2;B4DKR0;	4;4;4;3;3;3;1	Syntaxin-5	STX5	19.7	39.672	355	2.8758	1.1436	2	0.193582525
P20618	12	Proteasome subunit beta type-1	PSMB1	69.7	26.489	241	3.3879	1.1426	25	0.192320435
E7ES68;Q9BQ52;G5E9D5;Q	3;2;2;2;1;1;1;1	Zinc phosphodiesterase ELAC protein 2	ELAC2	7.5	70.106	626	3.0558	1.1423	2	0.191941593
Q9BPX3;H0Y9Z8	7;1	Condensin complex subunit 3	NCAPG	8.9	114.33	1015	2.7209	1.1422	4	0.19181529
P82912;P82912-2;H0YL99;P	6;6;3;2	28S ribosomal protein S11, mitochondrial	MRPS11	37.1	20.616	194	3.0465	1.142	6	0.191562651
Q9Y2A7-2;Q9Y2A7	8;8	Nck-associated protein 1	NCKAP1	9.3	129.52	1134	2.2617	1.1404	3	0.189539944
O14579;O14579-2;A6NE29	10;9;7	Coatomer subunit epsilon	COPE	50.6	34.482	308	2.226	1.1403	11	0.189413431
Q92979	6	Ribosomal RNA small subunit methyltransferase NEP1	EMG1	27.5	26.72	244	2.4735	1.1393	5	0.188147687
Q8N6M3	1	Fat storage-inducing transmembrane protein 2	FITM2	6.5	29.855	262	1.3175	1.1391	2	0.187894405
J3KPV7;P25325;B1AH49	9;9;8	3-mercaptopyruvate sulfurtransferase;Sulfurtransferase	MPST	39.4	35.249	317	2.8118	1.1388	7	0.187514398
E7EX29;P63104;B0AZS6;E7	17;17;12;10;9;9;9;6;6	14-3-3 protein zeta/delta	YWHAZ	63.4	28.036	246	3.2536	1.1388	30	0.187514398
Q8NE01;Q8NE01-3;Q8NE01	4;4;4	Metal transporter CNNM3	CNNM3	8.2	76.118	707	2.1023	1.1377	4	0.186120184
P19022;A8MWK3;P55283;G	12;12;2;2;2;2	Cadherin-2	CDH2	18	99.808	906	3.4129	1.1367	11	0.184851546
Q12981;Q12981-1;Q12981-	3;2;1	Vesicle transport protein SEC20	BNIP1	12.3	26.132	228	2.6145	1.1359	2	0.183835831
O60264;P28370;P28370-2;F	10;5;5;5	SWI/SNF-related matrix-associated actin-dependent reg	SMARCA5;SMARC	9.3	121.9	1052	2.0152	1.1359	7	0.183835831
O75600-2;O75600	2;2	2-amino-3-ketobutyrate coenzyme A ligase, mitochondri	GCAT	7.9	47.974	445	2.3723	1.1358	2	0.183708817
P35250;P35250-2;H7C5P4;H	9;8;3;2;2;2;2;2;1	Replication factor C subunit 2	RFC2	33.1	39.157	354	2.5342	1.1341	8	0.181547856
P42704;B8ZZ38;C9JCA9;H7	100;30;24;4	Leucine-rich PPR motif-containing protein, mitochondria	LRPPRC	77.1	157.9	1394	4.0004	1.1338	162	0.181166174
P54578;B7Z4N8;J3QRZ5;A6	3;3;3;3;1;1	Ubiquitin carboxyl-terminal hydrolase 14;Ubiquitin carbo	USP14	9.7	56.068	494	1.8941	1.1328	3	0.179893171
P09012	12	U1 small nuclear ribonucleoprotein A	SNRPA	47.5	31.279	282	3.0571	1.1327	26	0.179765808
P43155;P43155-2;P43155-3	6;6;5;2;1	Carnitine O-acetyltransferase	CRAT	12.5	70.857	626	2.3907	1.1308	6	0.177343788
P53004;C9J1E1	2;1	Biliverdin reductase A	BLVRA	8.8	33.428	296	1.57	1.1303	2	0.176705738
P49914;H3BQ75	4;4	5-formyltetrahydrofolate cyclo-ligase	MTHFS;ST20-MTH	23.6	23.255	203	1.9576	1.1293	2	0.175428791
Q8N1G2;Q5T7F5;H0YCQ1;H	13;4;3;1	Cap-specific mRNA (nucleoside-2-O-)-methyltransferase	FTSJD2	20.6	95.32	835	2.1215	1.1292	12	0.175301034
P25789;H0YL69;H0YMZ1;H	13;13;13;11;11;10;9;9	Proteasome subunit alpha type-4;Proteasome subunit al	PSMA4	71.6	29.483	261	3.3008	1.1282	16	0.174022842
Q6FI13;Q16777	7;7	Histone H2A type 2-A;Histone H2A type 2-C	HIST2H2AA3;HIST	58.5	14.095	130	3.3327	1.128	24	0.173767068
Q9Y3B4	5	Pre-mRNA branch site protein p14	SF3B14	48	14.585	125	2.6632	1.1271	16	0.172615522
Q9Y5K6	6	CD2-associated protein	CD2AP	9.5	71.45	639	1.7161	1.127	4	0.172487516
P62136;P62136-2;A6NNR3;	16;15;12;11;3;3	Serine/threonine-protein phosphatase PP1-alpha catalyt	PPP1CA	58.8	37.512	330	3.3354	1.1269	20	0.172359498
Q9NPE3;H0YM60	3;1	H/ACA ribonucleoprotein complex subunit 3	NOP10	57.8	7.7059	64	3.58	1.1264	4	0.171719239
P22087	8	rRNA 2-O-methyltransferase fibrillarin	FBL	39.9	33.784	321	2.917	1.1253	6	0.170309669
Q9H3P7	6	Golgi resident protein GCP60	ACBD3	15.2	60.593	528	1.9834	1.1246	6	0.169411952
P18206;P18206-2;Q5JQ13;E	9;9;6;2;1	Vinculin	VCL	9.9	123.8	1134	1.8023	1.1242	2	0.16889872
P33908;Q6P052	13;9	Mannosyl-oligosaccharide 1,2-alpha-mannosidase IA	MAN1A1	31.5	72.968	653	2.7668	1.124	19	0.168642036
Q8NF37	7	Lysophosphatidylcholine acyltransferase 1	LPCAT1	14.2	59.151	534	2.443	1.1236	7	0.16812853
P82664	6	28S ribosomal protein S10, mitochondrial	MRPS10	49.3	22.999	201	2.8432	1.1236	4	0.16812853
Q5XKP0;K7EKR0;K7EIR2	2;1;1	Protein QIL1	QIL1	35.6	13.087	118	1.6293	1.1227	3	0.166972472
O95831;O95831-3;O95831-	30;29;15;15;14;14;13	Apoptosis-inducing factor 1, mitochondrial	AIFM1	58.1	66.9	613	3.2375	1.1222	53	0.166329818
H0Y7A7;E7ETZ0;P62158;E7	11;11;11;10;8;7;5;3;3	Calmodulin	CALM2;CALM1	64.7	20.762	187	2.5694	1.1217	18	0.165686877
Q4G176;F5H2G6;F5H5A1;F	3;2;2;2;2;1;1;1;1	Acyl-CoA synthetase family member 3, mitochondrial	ACSF3	4.7	64.129	576	1.8986	1.119	3	0.162210036
Q9Y5S9;Q9Y5S9-2	6;5	RNA-binding protein 8A	RBM8A	46.6	19.889	174	2.7649	1.1168	7	0.159370847
Q9NQZ5;C9JTD3	4;2	StAR-related lipid transfer protein 7, mitochondrial	STARD7	12.4	43.113	370	2.9323	1.1165	4	0.158983251
O14561;H3BNK3;I3L505	4;3;2	Acyl carrier protein, mitochondrial;Acyl carrier protein	NDUFAB1	21.2	17.417	156	1.8273	1.1156	7	0.157819839
Q99747;B4DFC9;J3QKW4;J	2;1;1;1	Gamma-soluble NSF attachment protein	NAPG	6.7	34.746	312	2.3542	1.1153	2	0.157431827
P62330	4	ADP-ribosylation factor 6	ARF6	36	20.082	175	2.3308	1.1152	4	0.157302466
P36969;P36969-2;K7ERP4;K	6;6;6;5;5;5;4	Phospholipid hydroperoxide glutathione peroxidase, mi	GPX4	38.6	22.192	197	2.873	1.115	5	0.15704371
D6W592;Q8WVV9;Q8WVV	3;3;3;3;3;1;1;1;1;1	Heterogeneous nuclear ribonucleoprotein L-like	HNRPLL	6.5	60.099	542	1.9287	1.1117	2	0.152767519
Q9BZK7;O60907;O60907-2;	8;4;4;4;4;1;1;1;1	F-box-like/WD repeat-containing protein TBL1XR1;F-box	TBL1XR1;TBL1X;TE	22.8	55.594	514	2.256	1.11	8	0.150559677
Q9NUQ7;D6RGX2;D6RA67;	3;2;2;2;1;1;1	Ufm1-specific protease 2	UFSP2	9.6	53.261	469	2.2988	1.1099	2	0.150429698
Q9NYU2;Q9NYU2-2;H7BZG	87;87;6;1;1	UDP-glucose:glycoprotein glucosyltransferase 1	UGGT1	72.1	177.19	1555	3.799	1.1086	148	0.148738913
A3KMH1;A3KMH1-3;E2QR	31;31;30;18;4	Uncharacterized protein KIAA0564	KIAA0564	20	214.82	1905	2.94	1.1086	28	0.148738913
Q13510-2;Q13510;E7EMM4	6;6;6;2	Acid ceramidase;Acid ceramidase subunit alpha;Acid cer	ASAH1	17.8	46.503	411	2.4852	1.1074	4	0.147176427
Q8IZ83;Q8IZ83-3;F5H4B6;F	3;3;2;2;1	Aldehyde dehydrogenase family 16 member A1	ALDH16A1	4.9	85.126	802	2.082	1.107	4	0.146655222
P09601;B1AHA8	5;4	Heme oxygenase 1	HMOX1	25.7	32.818	288	2.9037	1.107	4	0.146655222
Q9H0S4;G5E955	2;2	Probable ATP-dependent RNA helicase DDX47	DDX47	6.2	50.646	455	1.7777	1.106	2	0.145351386
P52907	13	F-actin-capping protein subunit alpha-1	CAPZA1	64	32.922	286	3.0589	1.106	16	0.145351386
P26196	12	Probable ATP-dependent RNA helicase DDX6	DDX6	38.1	54.416	483	2.6102	1.1059	10	0.145220937
Q86YS6;A6NDJ8;E9PBQ0;Q	8;8;2;1;1;1;1;1;1	Ras-related protein Rab-43;Putative Rab-43-like protein	RAB43	38.2	23.339	212	2.8872	1.1058	8	0.145090477
Q96I59;E9PRK2;G3V178;H0	5;3;2;2	Probable asparagine--tRNA ligase, mitochondrial	NARS2	15.9	54.089	477	1.6462	1.1048	4	0.143785225
A2A274;Q99798	23;23	Aconitate hydratase, mitochondrial	ACO2	39.9	87.819	805	2.5366	1.1039	10	0.142609487
Q12789;Q12789-3;H3BU37;	11;11;3;2	General transcription factor 3C polypeptide 1	GTF3C1	6.3	238.87	2109	2.0902	1.1037	13	0.142348082
P55157;E9PBP6;D6REL9;Q6	48;47;11;8	Microsomal triglyceride transfer protein large subunit	MTTP	60.6	99.35	894	3.7968	1.1029	72	0.141301988
Q9Y639;F5GZD0;Q9Y639-5;	4;4;4;4;4;4;4;4;1;1;1	Neuroplastin	NPTN;DKFZp566H	12.3	44.387	398	3.3309	1.1025	4	0.140778656
P30038;D2D4A3;P30038-2;(	10;9;9;4	Delta-1-pyrroline-5-carboxylate dehydrogenase, mitoch	ALDH4A1	22.9	61.719	563	2.7078	1.1022	10	0.140386032
Q9H3N1;G3V448	10;2	Thioredoxin-related transmembrane protein 1	TMX1	27.9	31.791	280	3.2392	1.1003	10	0.137896932
E9PK25;P23528;E9PP50;G3	14;14;13;13;10;7;7	Cofilin-1	CFL1	58.3	22.728	204	2.6023	1.0998	29	0.137241192
Q9Y287	1	Integral membrane protein 2B;BRI2, membrane form;BRI	ITM2B	3.8	30.338	266	1.1942	1.0978	2	0.134615244
Q8WYA6;B4DE16;Q8WYA6-	6;5;5;5	Beta-catenin-like protein 1	CTNNBL1	10.5	65.173	563	2.4009	1.0952	5	0.131194352
P09382;F8WEI7	8;2	Galectin-1	LGALS1	71.9	14.716	135	2.2347	1.0916	9	0.1264443
O75494;O75494-2;O75494-	6;6;6;6;6;6;6;3	Serine/arginine-rich splicing factor 10;Serine/arginine-ri	SRSF10;FUSIP1;SR	23.7	31.3	262	3.2289	1.0904	10	0.124857467
F8W1H5;P51148;K7ERQ8;K	10;10;6;6;5;5;4;3;2;1;	Ras-related protein Rab-5C	RAB5C	62.2	27.035	249	2.9974	1.0902	32	0.124592825
Q6GMV3	2	Putative peptidyl-tRNA hydrolase PTRHD1	PTRHD1	16.4	15.805	140	2.3799	1.0895	2	0.123666196
Q10713;Q5SXN0;B4DKL3;Q	12;5;4;2	Mitochondrial-processing peptidase subunit alpha	PMPCA	29.1	58.252	525	3.0907	1.0894	7	0.123533772
Q5JTJ3-2;Q5JTJ3;Q5JTJ3-3	5;5;5	Uncharacterized protein C1orf31	C1orf31	27.6	18.094	156	2.9986	1.089	5	0.123003954
P02753;Q5VY30	6;6	Retinol-binding protein 4;Plasma retinol-binding protein	RBP4	34.8	23.01	201	2.2443	1.0867	4	0.119953718
P06493;E5RIU6;P06493-2	3;3;1	Cyclin-dependent kinase 1	CDK1	12.5	34.095	297	1.7129	1.0867	2	0.119953718
O15143;F8WCG3;C9J6C8;C	9;7;6;6;6;6;6;5;5;4;1;	Actin-related protein 2/3 complex subunit 1B	ARPC1B	35.2	40.949	372	3.3297	1.0854	10	0.118226814
P84090;G3V279	7;5	Enhancer of rudimentary homolog	ERH	53.8	12.259	104	2.6144	1.0851	15	0.117828004
Q9H488;B4DUV4;Q9H488-2	11;6;4	GDP-fucose protein O-fucosyltransferase 1	POFUT1	40.7	43.955	388	2.6654	1.0837	10	0.115965432
P07384;E9PRM1;E9PQB3;E	7;1;1;1;1;1;1;1;1;1;1	Calpain-1 catalytic subunit	CAPN1	9.8	81.889	714	2.2863	1.0821	6	0.113833829
Q9UBB4;Q9UBB4-2	3;3	Ataxin-10	ATXN10	7.2	53.488	475	1.4766	1.0818	4	0.113433803
Q14683;G8JLG1;H0Y7K8	42;21;10	Structural maintenance of chromosomes protein 1A	SMC1A	40.5	143.23	1233	3.1369	1.0815	42	0.113033665
O75909-4;O75909;G3V5E1;	4;4;3;3;3;3;2	Cyclin-K	CCNK	9	66.136	600	2.9036	1.0809	5	0.112233058
P14543;P14543-2;Q14112;(	3;3;1;1;1	Nidogen-1	NID1	3.4	136.38	1247	2.3733	1.079	3	0.109694865
Q9Y6N7-2;Q9Y6N7;Q9Y6N	11;11;11;11;11;11;3;2	Roundabout homolog 1	ROBO1	9.9	181.33	1655	2.4375	1.0787	15	0.109293689
Q13617-2;Q13617;Q5T2B5;	3;3;3;3;1	Cullin-2	CUL2	5	89.491	764	2.424	1.0771	4	0.107152199
P43034;I3L2U8;I3L3N5;B4D	3;3;1;1;1	Platelet-activating factor acetylhydrolase IB subunit alph	PAFAH1B1	9.3	46.637	410	1.9988	1.0768	3	0.106750315
Q6P9B9	2	Integrator complex subunit 5	INTS5	2.6	107.99	1019	1.785	1.0767	2	0.106616329
P06730-2;D6RBW1;P06730-	3;3;3;3;2;1;1;1	Eukaryotic translation initiation factor 4E	EIF4E	10.1	28.777	248	3.1571	1.0756	3	0.10514166
Q3KQZ2;O43760;K7ENG9;K	4;4;3;3;2;1	Synaptogyrin-2	SYNGR2	13.8	30.375	275	3.523	1.0721	3	0.100439479
Q96EX1;E5RH51	3;2	UPF0767 protein C1orf212	C1orf212	31.5	10.799	92	1.7545	1.0711	4	0.099093179
O95573;H7BYZ7;H7C260;F5	33;7;6;5;4;3	Long-chain-fatty-acid--CoA ligase 3	ACSL3	57.2	80.419	720	3.4117	1.0694	63	0.096801582
Q9C0B1;B4DHN4;Q9C0B1-2	3;1;1;1;1	Alpha-ketoglutarate-dependent dioxygenase FTO	FTO	7.7	58.281	505	1.3783	1.067	3	0.093560176
P37108;H0YLA2	6;5	Signal recognition particle 14 kDa protein	SRP14	40.4	14.57	136	3.1186	1.0667	12	0.093154488
Q8WUD1;E9PE37	13;5	Ras-related protein Rab-2B	RAB2B	71.3	24.214	216	1.9347	1.0664	5	0.092748686
Q07954;Q6PJ72;H0YJI8;Q9	62;3;3;1	Prolow-density lipoprotein receptor-related protein 1;L	LRP1	17.4	504.6	4544	2.3887	1.0663	68	0.092613393
Q99536;B4DPX4;B0AZP7;K	16;13;10;7;6;3;3;2;1	Synaptic vesicle membrane protein VAT-1 homolog	VAT1	62.6	41.92	393	3.2929	1.0656	22	0.091665988

J3KPL4;O94822;H7BYG8;O9	4;4;2;1	E3 ubiquitin-protein ligase listerin	LTN1	2.8	205.17	1812	1.7319	1.0652	3	0.091124334
P11021	39	78 kDa glucose-regulated protein	HSPA5	57.3	72.332	654	3.2575	1.065	151	0.09085343
P22059	3	Oxysterol-binding protein 1	OSBP	4.2	89.42	807	2.3753	1.065	2	0.09085343
Q9NTJ3;E9PD53;Q9NTJ3-2;	26;26;24;11;6;5;5;2;2	Structural maintenance of chromosomes protein 4;Struct	SMC4	23	147.18	1288	2.8325	1.0643	22	0.089904868
P31040;E9PBJ5;D6RFM5;H0	12;11;11;1	Succinate dehydrogenase [ubiquinone] flavoprotein sub	SDHA	24.8	72.691	664	2.2803	1.0637	4	0.089091319
Q15631;E9PGT1;H7C1D4;B7	13;11;9;7	Translin	TSN	71.1	26.183	228	3.0706	1.0618	11	0.086512047
H0Y886;O43674;E7EWP0;O	3;3;3;3	NADH dehydrogenase [ubiquinone] 1 beta subcomplex s	NDUFB5	11.2	23.434	206	2.5962	1.0618	4	0.086512047
O14975;O14975-2;G3V1R7	4;4;2	Very long-chain acyl-CoA synthetase	SLC27A2	8.2	70.311	620	2.0164	1.0616	5	0.086240276
Q12797;F5H667;E5RG56;Q1	20;19;2;2;2;2;2;2;1;1;	Aspartyl/asparaginyl beta-hydroxylase	ASPH	33.5	85.862	758	2.8943	1.0607	15	0.085016673
Q9H3Z4;Q9H3Z4-2	4;4	DnaJ homolog subfamily C member 5	DNAJC5	37.4	22.149	198	1.8051	1.0604	2	0.084608575
Q9HD34;F5H189;C9JRX8;H7	4;2;2;2;2	LYR motif-containing protein 4	LYRM4	37.4	10.758	91	2.095	1.0598	2	0.083792032
P33527;P33527-9;P33527-3	20;19;19;19;18;18;17	Multidrug resistance-associated protein 1	ABCC1	17.4	171.59	1531	2.9639	1.0589	15	0.082566351
P63208;E5RJR5;P63208-2;F	9;8;6;6;6;4	S-phase kinase-associated protein 1	SKP1	69.3	18.658	163	1.7646	1.0581	9	0.081475982
P50453;P50452;O75830;P5(	3;2;1;1;1;1;1	Serpin B9;Serpin B8	SERPINB9;SERPIN	8.5	42.403	376	1.5604	1.0571	2	0.08011186
P35249;C9JZI1;C9J8M3;C9J	7;6;4;2;2;2	Replication factor C subunit 4	RFC4	19.6	39.681	363	2.7476	1.0571	5	0.08011186
O75643;C9JAQ9;O75643-2;	110;26;26;25	U5 small nuclear ribonucleoprotein 200 kDa helicase	SNRNP200	65.4	244.5	2136	3.0361	1.057	230	0.079975377
Q15021;F5GZJ1;E7EN77;F5(	19;17;12;2;1;1	Condensin complex subunit 1	NCAPD2	17.7	157.18	1401	2.5001	1.0562	16	0.078883047
Q96N67;Q96N67-6;Q96N67	4;4;4;4;4;4;3;1	Dedicator of cytokinesis protein 7	DOCK7	2.6	242.56	2140	1.8914	1.0557	4	0.07819992
P01116-2;P01116;G3V5T7;(	6;5;1;1	GTPase KRas;GTPase KRas, N-terminally processed	KRAS	44.7	21.424	188	3.4733	1.0553	2	0.077653186
Q8NHH9;B7Z7X8;Q8NHH9-	8;8;7;7;6;5;4;4;2	Atlastin-2	ATL2	17.8	66.228	583	2.083	1.055	11	0.077242999
Q9UQE7	42	Structural maintenance of chromosomes protein 3	SMC3	41.8	141.54	1217	3.0091	1.054	36	0.075874867
P07900-2;P07900;Q86U12;(	44;44;30;11;8;7	Heat shock protein HSP 90-alpha	HSP90AA1	46.6	98.16	854	3.4872	1.0529	51	0.074368422
Q9P287-2;Q9P287;Q9P287-	2;2;2;2;2	BRCA2 and CDKN1A-interacting protein	BCCIP	7.1	36.214	322	2.2912	1.0519	2	0.07299756
P52789;E9PB90;P52790	26;24;1	Hexokinase-2	HK2	36.8	102.38	917	2.8481	1.0489	16	0.068877141
Q6UXN9;C9JBU3	8;1	WD repeat-containing protein 82	WDR82	31	35.079	313	2.5875	1.0488	11	0.068739591
P02647;F8W696	7;5	Apolipoprotein A-I;Truncated apolipoprotein A-I	APOA1	28.8	30.777	267	2.3395	1.0477	6	0.067225673
Q96NT0;B8ZZ99;F8WCZ3;B	2;2;1;1	Coiled-coil domain-containing protein 115	CCDC115	15	19.76	180	2.5735	1.0468	2	0.065985829
Q9H0D6;B4DZC3;Q9H0D6-2	23;23;22	5-3 exoribonuclease 2	XRN2	29.5	108.58	950	2.4756	1.0466	14	0.065710164
P43897-2;P43897;F8W6R3;(	6;6;5;4;3;3;3;3;2;1	Elongation factor Ts, mitochondrial;Elongation factor Ts	TSFM	28.9	37.656	346	2.4019	1.0464	6	0.065434446
Q8NCW5;Q8NCW5-2;Q5T3	3;3;1;1	NAD(P)H-hydrate epimerase	APOA1BP	17	31.674	288	1.843	1.045	2	0.063502942
Q14139-2;Q14139;B7Z7P0	11;11;5	Ubiquitin conjugation factor E4 A	UBE4A	15.5	123.52	1073	2.9875	1.0447	6	0.063088712
P46060;F8W7I9;H0Y4Q3;B0	16;11;5;4;4;2	Ran GTPase-activating protein 1	RANGAP1	32.2	63.541	587	2.5393	1.0447	15	0.063088712
Q9Y5M8;H7C4H2;C9J5Z8	16;11;3	Signal recognition particle receptor subunit beta	SRPRB	63.8	29.702	271	2.8202	1.0446	35	0.062950609
H0Y368;Q5QPK2;O60762;E(	14;14;14;11;8	Dolichol-phosphate mannosyltransferase	DPM1	54.9	33.218	295	2.7143	1.0444	19	0.062674363
G3XAI2;P07942;E7EPA6	15;15;4	Laminin subunit beta-1	LAMB1	10.5	200.48	1810	3.0041	1.0436	12	0.061568849
P82921	3	28S ribosomal protein S21, mitochondrial	MRPS21	39.1	10.741	87	3.1332	1.0432	6	0.061015775
B7Z5W1;Q9Y624;B7Z941	13;13;9	Junctional adhesion molecule A	F11R	50.2	32.858	303	2.7239	1.0431	19	0.060877473
Q9NX24;D6RCB9;D6RC52;J	3;2;2;1;1	H/ACA ribonucleoprotein complex subunit 2	NHP2	25.5	17.201	153	3.4006	1.0429	3	0.06060083
Q86Y79;C9J7Z1	2;1	Probable peptidyl-tRNA hydrolase	PTRH1	8.9	22.936	214	1.9761	1.0426	2	0.060185765
P52434;C9JLU1;C9JCU7;C9J	5;5;4;3;3;1	DNA-directed RNA polymerases I, II, and III subunit RPAE	POLR2H	40.7	17.143	150	3.1394	1.0424	5	0.059908989
Q96HR9;A8MWX0;A8MXN	9;5;5	Receptor expression-enhancing protein 6	REEP6	44.6	20.733	184	2.1443	1.0423	15	0.059770581
P61604;B8ZZL8;B8ZZ54	18;13;7	10 kDa heat shock protein, mitochondrial	HSPE1	89.2	10.932	102	1.6669	1.041	45	0.057970069
O15511;O15511-2;B1ALC0	5;4;4	Actin-related protein 2/3 complex subunit 5	ARPC5	39.1	16.32	151	2.8306	1.0404	5	0.057138304
Q8NEW0;H0Y362	3;2	Zinc transporter 7	SLC30A7	10.9	41.625	376	2.1791	1.0396	3	0.056028539
A6NHR9;A6NHR9-2;J3KTL8	2;2;2;1	Structural maintenance of chromosomes flexible hinge d	SMCHD1	1	226.37	2005	1.9645	1.0392	2	0.055473336
P62937;C9J5S7;F8WE65;Q5	12;8;8;7;2;1;1	Peptidyl-prolyl cis-trans isomerase A;Peptidyl-prolyl cis-	PPIA	67.3	18.012	165	3.0656	1.0387	25	0.054779032
O14744;O14744-2;G3V5W5	14;13;12;11;9;8;8;7;7	Protein arginine N-methyltransferase 5;Protein arginine	PRMT5	22.9	72.683	637	2.0924	1.0384	6	0.054362288
Q5VZU9;P29144	8;8	Tripeptidyl-peptidase 2	TPP2	8.9	139.76	1262	1.763	1.0384	3	0.054362288
P55268	12	Laminin subunit beta-2	LAMB2	9	195.98	1798	2.1885	1.0379	9	0.053667449
Q69YL0	4	Uncharacterized protein DKFZp762I1415		51.5	10.89	99	2.8074	1.0363	4	0.051441711
P53634;H0YCY8	7;3	Dipeptidyl peptidase 1;Dipeptidyl peptidase 1 exclusion	CTSC	23.5	51.853	463	2.4644	1.0353	6	0.05004888
P30419;P30419-2;B7Z8J4;K	6;4;3;2;1;1;1;1;1;1	Glycylpeptide N-tetradecanoyltransferase 1	NMT1	16.7	56.806	496	1.9247	1.0351	5	0.049770152
H7BYY3;Q5JWT2;Q9ULK4;C	12;12;12;12;12;12;9;7	Mediator of RNA polymerase II transcription subunit 23	MED23;CRSP3	11.8	157.1	1374	2.4151	1.0342	12	0.04851521
P16144;P16144-3;P16144-2	9;9;9;9;2;1;1	Integrin beta-4	ITGB4	6.9	202.16	1822	2.5929	1.0329	6	0.046700587
Q99584	4	Protein S100-A13	S100A13	40.8	11.471	98	1.9292	1.032	4	0.045442971
Q9H553;Q9H553-2	10;7	Alpha-1,3/1,6-mannosyltransferase ALG2	ALG2	33.2	47.091	416	2.4097	1.0307	11	0.043624477
O75937	7	DnaJ homolog subfamily C member 8	DNAJC8	30	29.841	253	3.0275	1.0307	6	0.043624477
G3V5T9;P24941;P24941-2;E	6;6;6;6;2;2;1;1;1;1;1;	Cyclin-dependent kinase 2	CDK2	23.1	39.178	346	1.8534	1.0304	5	0.043204499
Q6UX53	5	Methyltransferase-like protein 7B	METTL7B	21.7	27.775	244	1.9345	1.0301	5	0.042784398
P23921;E9PD78;E9PL69;F5H	2;2;2;1;1;1	Ribonucleoside-diphosphate reductase large subunit;Rib	RRM1	2.9	90.069	792	2.3398	1.0299	2	0.042504263
Q9H1A4;H0Y564	4;4	Anaphase-promoting complex subunit 1	ANAPC1	2.6	216.5	1944	2.2443	1.0292	3	0.041523362
P24752;H0YEL7;G3XAB4;E9	21;8;7;6;1	Acetyl-CoA acetyltransferase, mitochondrial	ACAT1	67.4	45.199	427	3.0743	1.0288	35	0.040962548
O60832;O60832-2;C9IYT0;H	6;5;3;2;2;1;1	H/ACA ribonucleoprotein complex subunit 4	DKC1	13.8	57.673	514	2.7531	1.0251	4	0.035764654
P67809;H0Y449;C9J5V9;P16	8;7;4;3;3;3;3	Nuclease-sensitive element-binding protein 1	YBX1	45.4	35.924	324	2.6981	1.0243	10	0.034638318
P06756;E7EWZ6;P06756-2;I	32;32;31;1	Integrin alpha-V;Integrin alpha-V heavy chain;Integrin al	ITGAV	37.1	116.04	1048	3.0425	1.0234	39	0.033370138
E9PEN8;Q9UIA9;E7ESC6;H0	17;17;16;5;1;1	Exportin-7	XPO7	18.4	124.84	1096	2.1638	1.0233	11	0.033229161
P49419;P49419-2;E7EPT3;F	12;12;10;10;5;5;4;2;2	Alpha-aminoadipic semialdehyde dehydrogenase	ALDH7A1	29.5	58.486	539	1.9898	1.0226	2	0.032241931
P40937;P40937-2;F8W9B4;	9;9;8;3;2;2;1	Replication factor C subunit 5	RFC5	31.2	38.496	340	2.667	1.0223	9	0.031818626
P04040	35	Catalase	CAT	71.3	59.755	527	3.0621	1.0209	99	0.029841557
Q5QNW6-2;P58876;P62807	7;7;7;7;7;7;7;7;6;6;6	Histone H2B type 1-D;Histone H2B type 1-C/E/F/G/I;Histo	HIST1H2BD;HIST1	51.5	14.841	134	3.1991	1.0208	30	0.029700234
P67775;P62714;P67775-2;E	11;10;9;6;5;5;4;4;2	Serine/threonine-protein phosphatase 2A catalytic subu	PPP2CA;PPP2CB	52.1	35.594	309	2.7692	1.0193	11	0.027578727
P12956;B1AHC9;B1AHC8;B	30;29;26;25;24	X-ray repair cross-complementing protein 6	XRCC6	51.7	69.842	609	2.9582	1.0172	60	0.024603367
B4DGP8;P27824;B4E2T8;H0	32;32;24;13;11;11;10	Calnexin	CANX	50.9	71.502	627	3.559	1.0171	43	0.02446153
O75400;O75400-2;O75400-	12;12;11;4;2;1	Pre-mRNA-processing factor 40 homolog A	PRPF40A	14.3	108.8	957	2.7467	1.0166	16	0.023752136
O15144;G5E9J0;C9JTV5;H7(	17;6;6;4;2	Actin-related protein 2/3 complex subunit 2	ARPC2	68	34.333	300	2.9644	1.0152	26	0.021763974
P30040;F8VY02;F8W1G0;C9	18;10;4;1	Endoplasmic reticulum resident protein 29	ERP29	62.8	28.993	261	3.1833	1.015	38	0.021479727
Q96QK1;I3L4P4;I3L4S0	13;1;1	Vacuolar protein sorting-associated protein 35	VPS35	22.5	91.706	796	2.2667	1.0123	14	0.017636903
P53597;H7C233	8;1	Succinyl-CoA ligase [ADP/GDP-forming] subunit alpha, m	SUCLG1	29.5	36.249	346	2.6533	1.0116	2	0.016638942
Q15836;K7N7D2;F8WCA0;F	7;3;3;3;3;3;3;1;1;1;1;	Vesicle-associated membrane protein 3	VAMP3	53	11.309	100	2.9956	1.011	12	0.015782997
Q13151	13	Heterogeneous nuclear ribonucleoprotein A0	HNRNPA0	52.5	30.84	305	2.85	1.0103	18	0.014783753
P07307;I3L1H2;I3L1N6	7;2;1	Asialoglycoprotein receptor 2	ASGR2	38.3	35.092	311	3.0358	1.0096	10	0.013783815
P62491;H3BSC1;B4DT13;H3	11;10;10;10;10;5;1;1	Ras-related protein Rab-11A	RAB11A	58.3	24.393	216	3.1875	1.0091	3	0.01306915
P61163;B4DXP9;F5H3I4	4;3;3	Alpha-centractin	ACTR1A	18.9	42.613	376	2.5222	1.0087	5	0.012497163
B1AK88;P47756-2;B1AK87;	19;19;18;15;13;12;5	F-actin-capping protein subunit beta	CAPZB	63.5	33.781	301	2.8499	1.0085	29	0.012211084
P30048;E9PH29	13;13	Thioredoxin-dependent peroxide reductase, mitochondı	PRDX3	65.6	27.692	256	2.6547	1.0083	24	0.011924948
Q9NUJ1;B7Z6A8;C9IZX5	11;8;4	Abhydrolase domain-containing protein 10, mitochondri	ABHD10	44.8	33.932	306	2.1187	1.0077	2	0.011066201
O00469-2;O00469;E7ETU9;I	28;28;27;16;9;1	Procollagen-lysine,2-oxoglutarate 5-dioxygenase 2	PLOD2	44.2	87.097	758	3.2618	1.007	29	0.010063683
P80365	2	Corticosteroid 11-beta-dehydrogenase isozyme 2	HSD11B2	8.4	44.126	405	2.6945	1.0069	2	0.00992041
Q7L5N1;E7EM64;H7C3T0	5;5;1	COP9 signalosome complex subunit 6	COPS6	19	36.163	327	2.7086	1.0044	3	0.006333934
Q92747;E9PF58;B4DLQ7;F8	5;4;3;1	Actin-related protein 2/3 complex subunit 1A	ARPC1A	18.9	41.569	370	2.2554	1.0024	3	0.00345832
O43143	28	Putative pre-mRNA-splicing factor ATP-dependent RNA I	DHX15	47.5	90.932	795	3.3596	1.0024	40	0.00345832
Q15392;B7Z817;F5H0H1;H7	20;19;12;8	Delta(24)-sterol reductase	DHCR24	51.4	60.101	516	2.7924	1.0014	37	0.002018361
Q8WU76;Q8WU76-2	2;2	Sec1 family domain-containing protein 2	SCFD2	3.1	75.126	684	1.3737	1.0006	2	0.000865357
P48449;E9PEI9;G5E9Q9;C9J	19;19;17;5;1	Lanosterol synthase	LSS	30.2	83.308	732	3.0593	0.9999	18	-0.000144277
P50613;D6RAD4;D6R9G1;D	2;2;1;1	Cyclin-dependent kinase 7	CDK7	10.1	39.038	346	2.5204	0.9988	2	-0.001732274
P19338;H7BY16;C9JWL1;C9	29;18;3;3;3;3	Nucleolin	NCL	33.1	76.613	710	3.9006	0.99812	76	-0.002714819
Q01105-2;Q01105;Q5VXV2	8;7;7;7;7	Protein SET	SET	38.6	32.103	277	2.9566	0.9979	9	-0.003032845
F5H2S7;Q13561-2;Q13561-	3;3;3;3;3;2;2;2;2;1;1;	Dynactin subunit 2	DCTN2	11.8	44.809	406	2.4519	0.99737	3	-0.003799286
P23378	38	Glycine dehydrogenase [decarboxylating], mitochondria	GLDC	47.3	112.73	1020	3.3456	0.99721	24	-0.004030745
O75821;K7EL20;K7ER90;K7I	6;4;4;4;3	Eukaryotic translation initiation factor 3 subunit G	EIF3G	19.7	35.611	320	2.3207	0.99635	5	-0.005275471
Q86Y56;Q86Y56-2;H0Y650;I	2;1;1;1;1	HEAT repeat-containing protein 2	HEATR2	2.7	93.52	855	1.7035	0.99538	2	-0.006680695
Q86U42;Q86U42-2;Q92843	5;5;4;3;3;2	Polyadenylate-binding protein 2	PABPN1	18	32.749	306	2.8105	0.99524	7	-0.006883624
P49589-3;P49589;B4DKY1;A	4;4;4;4;4	Cysteine--tRNA ligase, cytoplasmic	CARS	4.6	94.637	831	2.5759	0.99475	4	-0.007594101
Q14344;F5H1G8	8;8	Guanine nucleotide-binding protein subunit alpha-13	GNA13	23.6	44.049	377	2.6916	0.99161	6	-0.012155274
Q9Y697;Q9Y697-2;F5GYK5;I	8;8;6;6;5;2;1;1;1;1;1;	Cysteine desulfurase, mitochondrial	NFS1	20.6	50.195	457	2.1113	0.99049	4	-0.013785685
Q92575;C9JLR4;Q6PJ80;F8V	5;1;1;1	UBX domain-containing protein 4	UBXN4	15.6	56.777	508	2.6708	0.98912	9	-0.015782536
Q9Y2Z4;H0YHS6	9;8	Tyrosine--tRNA ligase, mitochondrial	YARS2	24.9	53.198	477	2.3079	0.98878	10	-0.016278533
Q8NE62;C9J7D8;C9JYW4	5;1;1	Choline dehydrogenase, mitochondrial	CHDH	9.9	65.358	594	2.672	0.98855	2	-0.016614157
E9PB02;P78346;Q5VU11;Q5	5;5;4;2	Ribonuclease P protein subunit p30	RPP30;RP11-320F	21.7	35.564	322	1.911	0.98694	3	-0.018965715
Q15274;H3BP73	8;1	Nicotinate-nucleotide pyrophosphorylase [carboxylating	QPRT	40.7	30.845	297	2.2805	0.98673	7	-0.019272722
Q8IWX8;J3QK89	15;14	Calcium homeostasis endoplasmic reticulum protein	CHERP	22.6	103.7	916	2.5251	0.98647	11	-0.019652918
P30044;P30044-2;A6NG06;	12;12;11;6	Peroxiredoxin-5, mitochondrial	PRDX5	56.5	22.086	214	2.9996	0.98536	17	-0.021277187
H9KV28;O60610;E9PEZ3;E7	8;8;8;8;8;8;8;8;8;6	Protein diaphanous homolog 1	DIAPH1	8.2	141.57	1273	2.143	0.98473	5	-0.022199884
E9PF41;P61160;F5H6T1	13;13;8	Actin-related protein 2	ACTR2	48.4	45.376	399	2.847	0.9834	22	-0.02414974
P53396;P53396-2;B4E3P0;K	32;32;24;4;2	ATP-citrate synthase	ACLY	36	120.84	1101	2.6511	0.98018	25	-0.028881385
Q9UBC2-2;Q9UBC2;A2RRF3	5;5;5;3	Epidermal growth factor receptor substrate 15-like 1	EPS15L1	5.9	99.605	910	1.9997	0.97955	3	-0.02980896
O75396	12	Vesicle-trafficking protein SEC22b	SEC22B	51.6	24.593	215	3.1663	0.9794	33	-0.030029899
O14737;B4DE64;K7EL31;K7I	6;4;4;4;3;2;2	Programmed cell death protein 5	PDCD5	47.2	14.285	125	1.6866	0.97857	7	-0.03125304
P98175;P98175-2;P98175-3	2;2;2;2	RNA-binding protein 10	RBM10	2.8	103.53	930	2.8108	0.97784	2	-0.032329673
O14618;J3KNF4;E9PP76	3;3;3	Copper chaperone for superoxide dismutase;Superoxide	CCS	10.6	29.04	274	2.1895	0.97659	2	-0.03417509
Q96T76-8;Q96T76;Q96T76-	13;13;12;11;8;8;4;3;2	MMS19 nucleotide excision repair protein homolog	MMS19	15.2	115.7	1051	1.9092	0.97607	9	-0.034943479
P53999;D6R970;D6RC37	6;1;1	Activated RNA polymerase II transcriptional coactivator p	SUB1	44.9	14.395	127	2.8503	0.97559	13	-0.035653125
E9PHH6;Q86XX4-2;Q86XX4	33;33;32;22;12;12;4;1	Extracellular matrix protein FRAS1	FRAS1	10.6	443.63	4012	2.0805	0.9746	31	-0.037117872
P35244;B5MC59	6;5	Replication protein A 14 kDa subunit	RPA3	80.2	13.569	121	2.2666	0.9738	7	-0.038302594
F8VVA7;P61923;F8VWL5;F	6;6;5;4;4;4;4;4;3;3;	Coatomer subunit zeta-1	COPZ1	37.4	22.34	198	2.3162	0.97362	9	-0.038569291
P61086;C9JGP1;B4DIZ2;P61	3;3;3;2;2;1	Ubiquitin-conjugating enzyme E2 K	UBE2K	16	22.406	200	2.5252	0.9728	3	-0.039784866
Q9P2I0	3	Cleavage and polyadenylation specificity factor subunit 2	CPSF2	5.2	88.486	782	2.0602	0.96957	3	-0.044583035
O15173	10	Membrane-associated progesterone receptor componen	PGRMC2	39.5	23.818	223	3.0557	0.96848	12	-0.046205839
Q15029;Q15029-2;K7EJ81;K	41;41;40;13;6;1	116 kDa U5 small nuclear ribonucleoprotein component	EFTUD2	57.4	109.43	972	3.2084	0.96811	47	-0.046757114

O43493;J3KQ45;F8WBK2;O	7;7;7;7;7;7;6;4;4	Trans-Golgi network integral membrane protein 2	TGOLN2	20.6	51.112	480	2.1681	0.968	5	-0.046921047
Q09161;F2Z2T1	17;1	Nuclear cap-binding protein subunit 1	NCBP1	26.7	91.838	790	2.6769	0.96727	17	-0.048009441
Q09028;Q09028-3;Q09028-	13;13;12;11;6;5;5;4;4	Histone-binding protein RBBP4	RBBP4	50.8	47.655	425	2.5109	0.9655	15	-0.050651836
Q6UB35;H0Y327;Q6UB35-2	10;3;3;3;2;2;2;2;2;1;1	Monofunctional C1-tetrahydrofolate synthase, mitochon	MTHFD1L	13.4	105.79	978	1.4326	0.96517	2	-0.051145021
P08758;D6RBL5;E7ENQ5;D6	13;11;10;8;6;1	Annexin A5;Annexin	ANXA5	41.6	35.936	320	2.4631	0.96488	15	-0.051578566
Q9Y3C6	3	Peptidyl-prolyl cis-trans isomerase-like 1	PPIL1	21.1	18.237	166	1.9524	0.96372	3	-0.053314049
Q14232;J3KMX9;B4DGX0;F	3;3;3;3;2	Translation initiation factor eIF-2B subunit alpha	EIF2B1	9.5	33.712	305	1.816	0.96344	3	-0.053733272
A7YIJ8;P35241;F5H1A7;E9P	17;17;12;5;4;3;3;3	Radixin	RDX	30.5	71.048	604	2.446	0.96248	11	-0.055171533
O75190;O75190-3;O75190-	2;2;2;2;1;1;1;1;1;1;	DnaJ homolog subfamily B member 6;DnaJ homolog subf	DNAJB6;DNAJB2;	7.1	36.087	326	1.7591	0.96163	2	-0.05644619
Q86UL3	3	Glycerol-3-phosphate acyltransferase 4	AGPAT6	8.3	52.07	456	1.4393	0.96085	2	-0.057616868
Q9P086;I3L2Q0;I3L3E8	3;2;2	Mediator of RNA polymerase II transcription subunit 11	MED11	41.9	13.129	117	2.8871	0.96074	3	-0.05778204
Q9UMS4;F5GY56;F5H2I0;H(	12;7;2;1;1	Pre-mRNA-processing factor 19	PRPF19	38.9	55.18	504	2.7414	0.95978	14	-0.059224345
J3KPW7;Q92769;B3KRS5;H	9;9;9;4;4;4;4;4;4;4;	Histone deacetylase 2;Histone deacetylase	HDAC2	18	65.537	582	2.3118	0.95868	5	-0.06087876
Q53H82	10	Beta-lactamase-like protein 2	LACTB2	42.4	32.805	288	2.427	0.9586	7	-0.060999155
P42285;F5H7E2;H0YAC4;D6	32;29;3;1;1	Superkiller viralicidic activity 2-like 2	SKIV2L2	39.7	117.8	1042	2.4175	0.95844	29	-0.061239975
O00299	12	Chloride intracellular channel protein 1	CLIC1	67.6	26.922	241	2.8431	0.95788	14	-0.062083164
Q02127;I3NI32;I3L449;J3QF	9;8;3;3;1	Dihydroorotate dehydrogenase (quinone), mitochondria	DHODH	26.3	42.867	395	2.5403	0.95715	6	-0.06318306
O75718;C9JP16	7;7	Cartilage-associated protein	CRTAP	18.5	46.561	401	2.3874	0.95282	6	-0.069724399
Q9Y265;Q9Y265-2;E7ETR0;I	9;7;6;3;2;1	RuvB-like 1	RUVBL1	28.3	50.227	456	2.7911	0.95242	9	-0.070330179
Q9UHV9	3	Prefoldin subunit 2	PFDN2	24	16.648	154	1.9954	0.95212	3	-0.07078468
Q8WVM0	9	Dimethyladenosine transferase 1, mitochondrial	TFB1M	35.8	39.542	346	2.3407	0.95194	6	-0.07105745
O00567;Q5JXT2;H0YDU4;H(	12;6;6;6	Nucleolar protein 56	NOP56;NOL5A	28.8	66.049	594	2.3651	0.95038	12	-0.073423619
O15160;O15160-2;E7EQB9;	3;3;2;2;1	DNA-directed RNA polymerases I and III subunit RPAC1	POLR1C	8.7	39.249	346	2.1034	0.94972	3	-0.074425859
P61026	15	Ras-related protein Rab-10	RAB10	65.5	22.541	200	2.0063	0.94926	22	-0.075124803
P26599-3;P26599-2;P26599	17;17;17;8;7;6;5;5	Polypyrimidine tract-binding protein 1	PTBP1	51.2	59.632	557	2.8033	0.94858	35	-0.076158644
P46109	6	Crk-like protein	CRKL	26.4	33.777	303	1.7688	0.94727	4	-0.0781524
P31949	4	Protein S100-A11	S100A11	41	11.74	105	2.2185	0.94668	4	-0.079051251
Q15185;B4DP11;B4DP21;B4	5;5;4;3;2	Prostaglandin E synthase 3	PTGES3	32.5	18.697	160	2.0693	0.94623	7	-0.079737193
Q63HN8-4;Q63HN8;H3BLU	51;51;35;14;6;4	E3 ubiquitin-protein ligase RNF213	RNF213	12.4	596.48	5256	1.9911	0.94422	45	-0.082805053
P35914;B1AK13;P35914-2;I	8;8;3;1	Hydroxymethylglutaryl-CoA lyase, mitochondrial	HMGCL	29.8	34.36	325	2.673	0.94389	6	-0.083309356
E7EP72;Q9UFG5;Q9UFG5-2	3;3;2;2;2;1;1;1	UPF0449 protein C19orf25	C19orf25	23.4	17.055	158	1.4387	0.94297	2	-0.084716222
P47755;F8W9N7;B4DG50;C	12;9;7;7	F-actin-capping protein subunit alpha-2	CAPZA2	62.2	32.949	286	2.758	0.9426	9	-0.085282414
Q01650;Q92536;Q9UM01;C	5;1;1;1;1	Large neutral amino acids transporter small subunit 1	SLC7A5	10.8	55.01	507	2.3453	0.94211	6	-0.086032577
Q7RTV0	7	PHD finger-like domain-containing protein 5A	PHF5A	61.8	12.405	110	2.9726	0.94202	7	-0.086170405
Q15233;F5GYZ3;H7C367;C9	22;21;14;12;9;3	Non-POU domain-containing octamer-binding protein	NONO	50.1	54.231	471	2.8915	0.94198	29	-0.086231666
Q69YN4;Q69YN4-3;Q69YN4	12;12;12;8;8	Protein virilizer homolog	KIAA1429	8.9	202.02	1812	1.7839	0.94163	10	-0.08676781
O95347;O95347-2;Q5T821	18;18;1	Structural maintenance of chromosomes protein 2	SMC2	19.2	135.65	1197	2.6853	0.9404	17	-0.088653556
Q9Y3D6	8	Mitochondrial fission 1 protein	FIS1	48	16.937	152	1.6612	0.9402	8	-0.088960414
P55209;H0YIV4;F5H4R6;F8\	5;5;5;5;5;5;5;5;5;5;	Nucleosome assembly protein 1-like 1	NAP1L1	12.8	45.374	391	2.5824	0.93976	4	-0.089635733
P04899;P04899-4;B3KTZ0;P	14;13;13;13;13;13;12	Guanine nucleotide-binding protein G(i) subunit alpha-2	GNAI2	48.5	40.45	355	2.6556	0.93948	6	-0.090065646
Q9P258	8	Protein RCC2	RCC2	21.1	56.084	522	2.5399	0.93794	7	-0.092432458
K7ELG9;Q3MHD2-2;Q3MHD	5;5;5	Protein LSM12 homolog	LSM12	33.5	24.955	230	2.4061	0.93756	4	-0.093017075
P54920	13	Alpha-soluble NSF attachment protein	NAPA	62	33.232	295	2.6362	0.93734	15	-0.093355645
Q00341;E7EM71;H0Y394;H7	50;46;36;17;12;10;10	Vigilin	HDLBP	48.5	141.45	1268	2.8523	0.93654	52	-0.094587481
P61158;B4DXW1;F5H3P5;Q	19;17;16;3;3;2;1;1;1;	Actin-related protein 3	ACTR3	65.6	47.371	418	2.7744	0.93507	22	-0.096853725
O95782;O95782-2	20;20	AP-2 complex subunit alpha-1	AP2A1	24.9	107.54	977	2.5848	0.93486	6	-0.097177765
P05981;B7Z1L4;A6NIE9;H3E	6;6;1;1;1;1;1;1;1;1;1	Serine protease hepsin;Serine protease hepsin non-cata	HPN	14.9	45.011	417	1.8809	0.93325	6	-0.099664491
Q9UI30;J3KNG5;F5GX77;F5	5;4;4;3	tRNA methyltransferase 112 homolog	TRMT112	53.6	14.199	125	2.8398	0.93216	6	-0.101350488
P23246;P23246-2;H0Y9K7;I	24;21;6;1	Splicing factor, proline- and glutamine-rich	SFPQ	40.6	76.149	707	3.4455	0.93188	34	-0.101783907
P40938;C9JU95	4;4	Replication factor C subunit 3	RFC3	14.6	40.556	356	1.9441	0.93152	4	-0.10234135
O43264;A1A528;F5H3C1	4;4;1	Centromere/kinetochore protein zw10 homolog	ZW10	6.8	88.828	779	1.5437	0.93012	3	-0.104511236
O15371;B4DVY1;B0QYA5;B	9;9;4;3;2;2;2;1	Eukaryotic translation initiation factor 3 subunit D	EIF3D	21	63.972	548	2.1603	0.9297	11	-0.105162839
P15151;P15151-2;P15151-3	5;5;5;4;2	Poliovirus receptor	PVR	13.2	45.302	417	2.6544	0.92876	5	-0.106622256
P60842;J3QLC4;J3KT12;J3Q	12;10;10;9;8;8;8;7;7;(	Eukaryotic initiation factor 4A-I	EIF4A1	34.7	46.153	406	2.4776	0.92717	10	-0.109094208
P31483;F8W8I6;P31483-2;C	7;7;6;6;6;5;4;4;2;1;1;	Nucleolysin TIA-1 isoform p40	TIA1	21	42.963	386	2.2688	0.92625	6	-0.110526457
Q12905	19	Interleukin enhancer-binding factor 2	ILF2	67.7	43.062	390	2.5058	0.92607	41	-0.110806847
Q9UHG3;B7Z9P8;B7Z3Y2;F8	18;17;17;9;8;6;5	Prenylcysteine oxidase 1	PCYOX1	47.7	56.639	505	2.7395	0.92565	23	-0.1114613
Q96C19;H0Y4Y4;Q9BUP0;E	4;4;2;2;1;1	EF-hand domain-containing protein D2;EF-hand domain-	EFHD2;EFHD1	15.4	26.697	240	2.112	0.92367	2	-0.114550584
Q8TEX9-2;Q8TEX9;H0YN14	5;5;3;3;3;1;1	Importin-4	IPO4	5.4	118.9	1083	1.4248	0.92209	4	-0.117020524
O75691	6	Small subunit processome component 20 homolog	UTP20	2.7	318.38	2785	1.5473	0.92148	5	-0.117975241
P28331-2;P28331;E7ENF3;P	2;2;2;2;2;2	NADH-ubiquinone oxidoreductase 75 kDa subunit, mitoc	NDUFS1	4	80.996	741	1.4367	0.92023	2	-0.119933605
Q8WUY1;H0YAR9	13;8	UPF0670 protein THEM6	THEM6	53.8	23.865	208	2.3319	0.92018	12	-0.120011995
Q5TDF0;Q9BSD7	4;4	Cancer-related nucleoside-triphosphatase	C1orf57;NTPCR	24.6	25.072	228	2.4247	0.9184	4	-0.122805453
Q96DB5;B4DZW6;E5RH53;E	11;10;9;8;8;5;5;4;4;4;	Regulator of microtubule dynamics protein 1	FAM82B	35.7	35.808	314	2.2833	0.91834	14	-0.122899709
Q96AY3;F8WA23;H0Y827;C	13;11;7;6;4;4;3;1;1	Peptidyl-prolyl cis-trans isomerase FKBP10	FKBP10	25.9	64.244	582	2.6292	0.91809	9	-0.123292507
P51991	21	Heterogeneous nuclear ribonucleoprotein A3	HNRNPA3	43.7	39.594	378	2.2227	0.91787	57	-0.123638259
Q9NWM8;H7C1Z9;F8WBZ0	6;4;2	Peptidyl-prolyl cis-trans isomerase FKBP14;Peptidyl-prol	FKBP14	37.9	24.171	211	2.099	0.9174	3	-0.124377187
Q9H583;Q5T3Q7	10;10	HEAT repeat-containing protein 1	HEATR1	6.2	242.37	2144	1.5272	0.91626	7	-0.126171056
P00387-3;P00387;P00387-2	22;22;22;15;2	NADH-cytochrome b5 reductase 3;NADH-cytochrome b5	CYB5R3	58.7	38.226	334	2.5113	0.91585	35	-0.126816765
O75323;O75323-2;H7C333;(	8;7;4;4;3;1	Protein NipSnap homolog 2	GBAS	42	33.742	286	2.5186	0.91525	9	-0.127762226
O15235	2	28S ribosomal protein S12, mitochondrial	MRPS12	15.2	15.173	138	1.5714	0.91289	2	-0.131487064
Q8NFH4;F8VTY2	3;1	Nucleoporin Nup37	NUP37	12.9	36.707	326	2.0958	0.9123	2	-0.132419778
Q13618;Q13618-2;Q13618-	2;2;2	Cullin-3	CUL3	2.9	88.929	768	1.6832	0.91149	2	-0.133701266
O94906;O94906-2	25;24	Pre-mRNA-processing factor 6	PRPF6	31.1	106.92	941	2.9037	0.90947	20	-0.136902045
P49915;F8W720	18;17	GMP synthase [glutamine-hydrolyzing]	GMPS	37.4	76.715	693	2.4321	0.9085	14	-0.13844158
Q00839;Q00839-2;Q5RI18	36;33;11	Heterogeneous nuclear ribonucleoprotein U	HNRNPU	46.2	90.583	825	3.071	0.90681	89	-0.141127794
P37235;E9PC71;P84074;H7I	5;4;2;1;1	Hippocalcin-like protein 1	HPCAL1	29	22.313	193	1.9085	0.90614	5	-0.142194129
O76024	2	Wolframin	WFS1	2.1	100.29	890	1.6777	0.90572	2	-0.14286298
P53618;E9PP73;E9PKQ1;E9	34;20;5;5	Coatomer subunit beta	COPB1	47	107.14	953	2.747	0.90099	36	-0.150417001
Q9NU22;Q5T795;REV__P21	80;21;1;1;1;1;1;1	Midasin	MDN1	19.4	632.81	5596	2.0668	0.89946	72	-0.15286897
B5MDU6;Q9H6V9;Q9H6V9-	10;10;9;6;6;5;5;5;4;4;	UPF0554 protein C2orf43	C2orf43	29.5	42.668	370	1.9527	0.89905	8	-0.153526743
Q86XP3;Q86XP3-2;J3KTK9;	20;18;2;2;1	ATP-dependent RNA helicase DDX42	DDX42	29.4	102.97	938	2.6734	0.899	21	-0.153606979
Q2T9J0;Q2T9J0-2	4;4	Peroxisomal leader peptide-processing protease;Peroxis	TYSND1	10.4	59.308	566	1.6894	0.89869	3	-0.154104546
I3L4A1;Q96FZ7;I3L4G8;I3L3	4;4;3;2	Charged multivesicular body protein 6	CHMP6	18.8	25.824	224	2.4413	0.89842	4	-0.154538051
P36873-2;P36873;F8VYE8;F	14;14;14;13;13;3;2;2;	Serine/threonine-protein phosphatase PP1-gamma catal	PPP1CC	50.4	38.518	337	2.0242	0.89526	2	-0.159621366
P37837;F2Z393;E9PKI8;E9P	14;13;1;1	Transaldolase	TALDO1	38	37.54	337	2.4568	0.89483	11	-0.16031447
A0AV56;B7ZLP6;Q15424;F5	9;9;9;9;8;8;3;1	Scaffold attachment factor B1	SAFB	10.5	102.85	917	2.0836	0.89341	10	-0.162605692
Q99714;Q99714-2;Q5H928	18;16;12	3-hydroxyacyl-CoA dehydrogenase type-2	HSD17B10	89.3	26.923	261	2.8236	0.89307	47	-0.163154835
P60174;P60174-1;B7Z5D8	16;16;9	Triosephosphate isomerase	TPI1	69.2	30.791	286	2.4903	0.89306	42	-0.163170989
P82909;D6R941	6;2	28S ribosomal protein S36, mitochondrial	MRPS36	69.9	11.466	103	1.5852	0.89239	3	-0.164253748
P50416;P50416-2;H3BUV7;	28;26;3;3;3;2;1;1;1;1	Carnitine O-palmitoyltransferase 1, liver isoform	CPT1A	45	88.367	773	2.7688	0.8912	33	-0.166178862
Q3ZAQ7;Q3ZAQ7-2	3;2	Vacuolar ATPase assembly integral membrane protein V	VMA21	34.7	11.354	101	2.8277	0.8908	5	-0.166826537
B7WPD3;Q15370;B8ZZU8;I	5;5;4;3	Transcription elongation factor B polypeptide 2	TCEB2	33.5	17.911	161	1.3812	0.89077	6	-0.166875124
P26583;D6R9A6	6;5	High mobility group protein B2	HMGB2	31.6	24.033	209	1.7513	0.89056	9	-0.167215281
P15927-3;P15927-2;P15927	3;3;3;3	Replication protein A 32 kDa subunit	RPA2	13.1	38.809	358	2.1154	0.88914	3	-0.169517498
Q5T4S7-2;Q5T4S7;Q5T4S7-	60;60;60;60;32;9;8;2;	E3 ubiquitin-protein ligase UBR4	UBR4	14.6	575.95	5204	1.9226	0.88792	44	-0.171498397
Q9ULC3	4	Ras-related protein Rab-23	RAB23	22.8	26.659	237	1.9377	0.88654	4	-0.173742369
Q10567-3;Q10567;Q10567-	28;27;27;27;18;3	AP-1 complex subunit beta-1	AP1B1	35.9	103.56	939	2.7577	0.88405	22	-0.177800127
Q15181;Q5SQT6	6;3	Inorganic pyrophosphatase	PPA1	27	32.66	289	1.2815	0.88398	5	-0.177914366
Q9H3G5;C9JLV0;H7C0X5;H	6;3;3;2	Probable serine carboxypeptidase CPVL	CPVL	12.6	54.163	476	1.7703	0.8839	6	-0.178044935
Q15393;Q15393-3;Q15393-	44;20;10;4;3;2;1;1	Splicing factor 3B subunit 3	SF3B3	46.8	135.58	1217	3.0489	0.88374	61	-0.17830611
Q9Y230;B3KQ59	13;11	RuvB-like 2	RUVBL2	32	51.156	463	2.5828	0.88351	10	-0.178681631
Q9H4A6;D6REM1	5;1	Golgi phosphoprotein 3	GOLPH3	29.2	33.81	298	2.0256	0.88232	5	-0.180626107
Q96BP2;A6NJX6	2;1	Coiled-coil-helix-coiled-coil-helix domain-containing pro	CHCHD1	22.9	13.475	118	2.3164	0.88137	2	-0.182180304
P00505;E7ERW2	23;20	Aspartate aminotransferase, mitochondrial;Aspartate am	GOT2	56.7	47.517	430	2.4451	0.8812	26	-0.1824586
Q9H6R3;Q9H6R3-2;F8VZB4	8;5;1	Acyl-CoA synthetase short-chain family member 3, mitoc	ACSS3	17.1	74.777	686	1.621	0.87876	2	-0.186458893
Q9Y6D5;E5RHL7;E5RJB2	19;2;2	Brefeldin A-inhibited guanine nucleotide-exchange prot	ARFGEF2	14.5	202.04	1785	1.5215	0.87752	15	-0.188496088
P15924;P15924-2	101;77	Desmoplakin	DSP	42	331.77	2871	1.9506	0.87613	65	-0.190783142
Q86UP2;Q86UP2-2;Q86UP2	29;29;27;16;6;4;4;4;2	Kinectin	KTN1	25.3	156.27	1357	2.2743	0.87536	26	-0.192051634
P31946;P31946-2;Q4VY19;(	13;13;3;3	14-3-3 protein beta/alpha;14-3-3 protein beta/alpha, N-t	YWHAB	53.7	28.082	246	1.7615	0.87532	17	-0.19211756
Q9Y678;H0Y8X7;H0Y8G2	29;6;1	Coatomer subunit gamma-1	COPG1	48.1	97.717	874	2.5785	0.87529	30	-0.192167007
Q9HCE1;Q5JR04;Q9HCE1-2	10;10;7	Putative helicase MOV-10	MOV10	11.9	113.67	1003	2.0133	0.87515	6	-0.19239778
Q16881;B7Z904;Q16881-6;E	6;6;6;6;6;6;6;6;6;6;(	Thioredoxin reductase 1, cytoplasmic	TXNRD1	17.7	70.923	649	1.856	0.87488	3	-0.192842947
P27694	2	Replication protein A 70 kDa DNA-binding subunit	RPA1	4.7	68.137	616	2.1453	0.87471	2	-0.193123308
E7EWS7;P68036;B4DDG1	4;4;4	Ubiquitin-conjugating enzyme E2 L3	UBE2L3	25.9	24.003	212	1.8297	0.87347	2	-0.195169941
O75915;F8WF33;C9JQU6;F	2;1;1;1	PRA1 family protein 3	ARL6IP5	16	21.614	188	2.3288	0.87333	7	-0.195401195
Q14739;C9JXK0;H7C108	5;2;1	Lamin-B receptor	LBR	9.1	70.702	615	1.8534	0.87326	4	-0.195516836
Q16850;Q16850-2;H7C0D0;	9;6;2;1	Lanosterol 14-alpha demethylase	CYP51A1	26	56.805	503	1.8965	0.87253	11	-0.196723359
P08238;Q58FF7;H0Y6E4;H0	47;22;10;9;8;8;1	Heat shock protein HSP 90-beta	HSP90AB1	63.4	83.263	724	2.9704	0.87148	87	-0.198460539
P41091;Q2VIR3;F8W810;Q	12;11;8;8;5	Eukaryotic translation initiation factor 2 subunit 3;Putativ	EIF2S3;EIF2S3L	36.4	51.109	472	2.1365	0.87031	10	-0.200398722
B4DR87;Q02809;Q5JXB7;Q	27;27;8;6;5	Procollagen-lysine,2-oxoglutarate 5-dioxygenase 1	PLOD1	42.6	88.271	774	2.9144	0.87025	32	-0.200498186
P22033	12	Methylmalonyl-CoA mutase, mitochondrial	MUT	19.5	83.134	750	2.2873	0.86894	10	-0.202671532
K7ELC2;P62841;K7EM56;K7	5;5;4;4;4	40S ribosomal protein S15	RPS15	53.3	17.723	152	1.4441	0.86835	15	-0.203651438
P31689;B7Z5C0	8;4	DnaJ homolog subfamily A member 1	DNAJA1	32	44.868	397	2.2986	0.86789	6	-0.204415894
P01034	2	Cystatin-C	CST3	19.2	15.799	146	1.1108	0.86694	2	-0.205995945
P04080	2	Cystatin-B	CSTB	24.5	11.139	98	2.7128	0.86614	3	-0.207327859
P46459;I3L0N3;B4DFA2;K7I	28;28;26;6;6;5;1;1	Vesicle-fusing ATPase	NSF	42.5	82.593	744	2.9826	0.86546	31	-0.208460953
O14734;E9PJN0;H7C5A7;E9	4;4;3;2;2;2;1;1;1	Acyl-coenzyme A thioesterase 8	ACOT8	16.3	35.914	319	1.8851	0.86449	5	-0.210078819
Q99653;H0YNG9;F5GX29;H	11;8;7;7;5;1	Calcium-binding protein p22	CHP	67.7	22.456	195	2.2205	0.862	8	-0.214240226
E7EQF2;Q9UKG9;C9J3D7;Q	7;7;7;1	Peroxisomal carnitine O-octanoyltransferase	CROT	15.6	73.382	640	2.4696	0.86154	7	-0.215010315
Q9BWU0;H7C256	6;1	Kanadaptin	SLC4A1AP	10.2	88.813	796	2.0477	0.86062	4	-0.216551727
P27695;G3V3M6;G3V5Q1;C	10;8;7;6;5;5;4;4;2;1	DNA-(apurinic or apyrimidinic site) lyase;DNA-(apurinic c	APEX1	47.2	35.554	318	2.2565	0.85955	10	-0.218346531
Q14258	3	E3 ubiquitin/ISG15 ligase TRIM25	TRIM25	6.7	70.973	630	1.1647	0.85927	3	-0.218816568
Q13547;Q5TEE2;F5GXM1	11;7;6	Histone deacetylase 1	HDAC1	25.9	55.102	482	2.4447	0.85816	12	-0.220681438
Q92538	44	Golgi-specific brefeldin A-resistance guanine nucleotide	GBF1	31	206.44	1859	2.0397	0.85732	42	-0.222094295

REV__K7ENY1;REV__Q8WZ	3;3;3;3;3;3;3;3;3;3			0.1	3994.6	35991	1.7796	0.8572	2	-0.222296245
P14678-3;B3KVR1;P14678;F	10;10;10;10;10;10;6	Small nuclear ribonucleoprotein-associated protein;Sma	SNRPN;SNRPB	24.2	30.032	289	2.2821	0.85599	29	-0.224334152
O75356;G3V4I0;G3V450;RE	11;11;4;1;1;1;1;1;1;	Ectonucleoside triphosphate diphosphohydrolase 5	ENTPD5	30.4	47.517	428	2.6473	0.85539	9	-0.225345754
P42765;K7EME0;K7EJ68;K7I	21;16;3;2;1	3-ketoacyl-CoA thiolase, mitochondrial	ACAA2	73.3	41.924	397	2.504	0.85538	26	-0.22536262
Q9NTZ6;Q5JX62;A6PVI0;A6	10;1;1;1	RNA-binding protein 12	RBM12	12	97.394	932	1.8243	0.85458	5	-0.226712541
Q8IWB7;C9JJ54	9;4	WD repeat and FYVE domain-containing protein 1	WDFY1	28	46.323	410	2.3264	0.85255	8	-0.230143648
Q13838;F8VQ10;Q13838-2;	20;19;19;18;15;14;14	Spliceosome RNA helicase DDX39B	DDX39B;BAT1	65.2	48.991	428	2.4372	0.85068	31	-0.233311559
Q5VYK3;J3KN16;Q5VYK5	44;43;6	Proteasome-associated protein ECM29 homolog	ECM29	35.5	204.29	1845	2.0037	0.84987	34	-0.234685918
P22314;Q5JRR6;Q5JRS0;Q5	31;9;9;9;7;6;6	Ubiquitin-like modifier-activating enzyme 1	UBA1	41.3	117.85	1058	2.3096	0.84924	17	-0.23575577
P55789;H3BRD2;H3BQQ4;P	6;4;3;3;3	FAD-linked sulfhydryl oxidase ALR	GFER	45.4	23.449	205	2.0183	0.84804	6	-0.23779578
O15118;K7EQ23;B4DET3	3;2;2	Niemann-Pick C1 protein	NPC1	2.4	142.17	1278	2.2205	0.84727	2	-0.239106308
O60568;H7C2S8;H7C2V1;H7	26;11;9;3;2;1;1	Procollagen-lysine,2-oxoglutarate 5-dioxygenase 3	PLOD3	48.1	84.784	738	2.8122	0.84159	28	-0.248810533
P0C0S8;P20671;Q9BTM1;Q	7;7;7;7;7;6;4	Histone H2A type 1;Histone H2A type 1-D;Histone H2A.J;I	HIST1H2AG;HIST1	58.5	14.091	130	2.8957	0.84155	3	-0.248879104
Q15084-2;B5MCQ5;F8WA8	18;18;18;18;18	Protein disulfide-isomerase A6	PDIA6	47	53.9	492	2.526	0.84132	27	-0.249273454
P30566;E7ERF4;P30566-2;B	5;4;4;1	Adenylosuccinate lyase	ADSL	13	54.889	484	1.313	0.84126	3	-0.249376346
P60228;E5RGA2;H0YBR5;E5	14;10;7;5;5;2;1;1;1;1	Eukaryotic translation initiation factor 3 subunit E	EIF3E	34.4	52.22	445	2.0589	0.8403	15	-0.251023611
P19823;Q5T985;Q5T987;CC	13;13;6;4	Inter-alpha-trypsin inhibitor heavy chain H2	ITIH2	16.6	106.46	946	2.5441	0.83748	10	-0.255873357
Q04760;Q04760-2	7;7	Lactoylglutathione lyase	GLO1	47.3	20.777	184	2.252	0.83256	7	-0.264373848
O75298;O75298-2;K7EMR7;	3;3;3;3;2	Reticulon-2	RTN2	7	59.263	545	2.1755	0.83246	2	-0.264547143
Q99735;Q99735-2	2;1	Microsomal glutathione S-transferase 2	MGST2	17.7	16.62	147	2.3595	0.83198	3	-0.265379247
H3BRF5;Q5JPH6;H3BTB7	7;7;7	Probable glutamate--tRNA ligase, mitochondrial	EARS2	14.6	59.988	534	2.1666	0.83195	6	-0.26543127
P11940;P11940-2;E7EQV3;E	20;20;19;18;13;12;10	Polyadenylate-binding protein 1;Polyadenylate-binding	PABPC1;PABPC3	34.4	70.67	636	1.8553	0.82891	37	-0.270712627
P53621;P53621-2	61;60	Coatomer subunit alpha;Xenin;Proxenin	COPA	62.6	138.34	1224	2.6799	0.82816	68	-0.272018572
P50570;K7ESI9;P50570-2;E9	17;17;17;15;15;15;4;4	Dynamin-2	DNM2	21.5	98.063	870	1.9651	0.82681	14	-0.274372257
Q96DH6;B4DHE8;Q96DH6-2	5;5;5;4;3;3;2	RNA-binding protein Musashi homolog 2	MSI2	21.3	35.196	328	2.1707	0.82667	5	-0.274616563
Q9Y5L4;K7EIT2	8;6	Mitochondrial import inner membrane translocase subur	TIMM13	78.9	10.5	95	2.4047	0.82494	14	-0.277638903
Q14517;D6RCE4	22;1	Protocadherin Fat 1;Protocadherin Fat 1, nuclear form	FAT1	5.7	506.27	4588	1.8132	0.82477	16	-0.277936237
Q86X76-3;Q86X76;Q86X76-	2;2;2;2;1	Nitrilase homolog 1	NIT1	6.5	40.2	367	1.4877	0.82446	2	-0.278478594
O60488;O60488-2;D6RF95;I	33;33;11;8;7;6;6	Long-chain-fatty-acid--CoA ligase 4	ACSL4	58.9	79.187	711	2.6325	0.82423	49	-0.27888112
Q14554;Q9BV43;H7C4F9;C9	11;6;1;1	Protein disulfide-isomerase A5	PDIA5	23.5	59.594	519	2.1471	0.82386	10	-0.279528896
P63167;F8VRV5;F8VXL2;F8	7;3;3;1	Dynein light chain 1, cytoplasmic	DYNLL1	82	10.366	89	1.9956	0.82369	13	-0.279826621
P05198;G3V4T5;H0YJS4	8;7;7	Eukaryotic translation initiation factor 2 subunit 1	EIF2S1	29.2	36.112	315	2.2486	0.82367	5	-0.279861652
Q08379;Q08379-2;Q9NVV4	2;2;1	Golgin subfamily A member 2	GOLGA2	2.2	113.08	1002	1.5166	0.82294	3	-0.281140846
J3KNF8;O43169;H3BUX2;D6	6;5;4;3;2	Cytochrome b5 type B	CYB5B	73.3	16.694	150	1.7368	0.82134	7	-0.283948535
Q9NQT4	3	Exosome complex component RRP46	EXOSC5	19.6	25.249	235	2.0953	0.82126	2	-0.284089062
Q4KMQ2-2;Q4KMQ2;E9PB3	7;7;7;6	Anoctamin-6	ANO6	9.9	108.43	931	1.8291	0.81716	6	-0.291309509
E9PS42;P21291;E9PP21;E9F	2;1;1;1	Cysteine and glycine-rich protein 1	CSRP1	15.7	16.58	153	1.9482	0.81674	2	-0.292051209
O60783	4	28S ribosomal protein S14, mitochondrial	MRPS14	29.7	15.139	128	2.5283	0.81631	5	-0.292810964
P22307;C9JC79;A6NM69;B4	22;22;21;20;15;14;12	Non-specific lipid-transfer protein	SCP2	36.7	58.993	547	2.1491	0.81561	57	-0.294048631
P46777;Q5T7N0	11;3	60S ribosomal protein L5	RPL5	40.7	34.362	297	2.0753	0.81561	16	-0.294048631
Q9NR31;Q5SQT8;B4DQ19	6;4;3	GTP-binding protein SAR1a	SAR1A	36.9	22.367	198	2.003	0.81477	6	-0.295535234
Q9H0X4;Q9H0X4-2;B4DGG1	3;2;2;2;1;1;1;1;1	Protein ITFG3	ITFG3	6.2	59.659	552	2.0798	0.81299	3	-0.298690488
Q8NI27;H0Y815;F2Z2V2	20;9;2	THO complex subunit 2	THOC2	14.6	182.77	1593	2.0234	0.81084	18	-0.302510834
P53680;P53680-2	5;4	AP-2 complex subunit sigma	AP2S1	35.9	17.018	142	2.3542	0.81082	6	-0.30254642
Q92572;F5H459	3;1	AP-3 complex subunit sigma-1	AP3S1	18.7	21.732	193	1.9134	0.80851	3	-0.306662477
O95881	7	Thioredoxin domain-containing protein 12	TXNDC12	58.7	19.206	172	2.2717	0.80821	6	-0.307197893
O43592;F8WDU6	6;2	Exportin-T	XPOT	7.5	109.96	962	1.7575	0.8079	3	-0.307751364
Q13347;Q5TFK1	13;3	Eukaryotic translation initiation factor 3 subunit I	EIF3I	52	36.501	325	2.072	0.80679	12	-0.309734893
Q96SB4-3;H3BLV9;Q96SB4;	2;2;2;2;2;1;1;1;1;1;1;	SRSF protein kinase 1;SRSF protein kinase 2;SRSF protein	SRPK1;SRPK2;SRP	2.7	92.41	826	1.637	0.80669	2	-0.309913723
Q5RKV6	5	Exosome complex component MTR3	EXOSC6	21.7	28.235	272	1.6543	0.80668	3	-0.309931607
Q96EY7;Q96EY7-2;B4DF73;I	13;4;3;3;1	Pentatricopeptide repeat-containing protein 3, mitochor	PTCD3	24.2	78.549	689	2.0602	0.80659	16	-0.310092575
O95059	2	Ribonuclease P protein subunit p14	RPP14	29	13.693	124	1.8811	0.80597	3	-0.311201955
Q9BQ69	5	O-acetyl-ADP-ribose deacetylase MACROD1	MACROD1	22.5	35.505	325	2.2876	0.80472	5	-0.313441206
B5ME19;Q99613;H3BRV0;H	21;21;19;4;4;3	Eukaryotic translation initiation factor 3 subunit C	EIF3CL;EIF3C	23.3	105.47	914	2.2412	0.80466	17	-0.313548777
P52272;P52272-2	30;30	Heterogeneous nuclear ribonucleoprotein M	HNRNPM	47.3	77.515	730	2.6826	0.80223	44	-0.317912177
Q58FF8	11	Putative heat shock protein HSP 90-beta 2	HSP90AB2P	23.4	44.348	381	3.3207	0.80191	2	-0.318487766
P61019;P61019-2;E9PKL7;H	15;11;11;6;5;2;1	Ras-related protein Rab-2A	RAB2A	75	23.545	212	2.2403	0.80089	31	-0.320323989
J3KR54;P26440;B3KVI7;H7C	3;3;3;3;3;1;1	Isovaleryl-CoA dehydrogenase, mitochondrial	IVD	11.3	46.65	426	1.9886	0.80042	3	-0.321170879
P46781;C9JM19;B5MCT8;A8	14;11;11;3;3	40S ribosomal protein S9	RPS9	45.9	22.591	194	1.6067	0.80042	16	-0.321170879
P55786;E9PLK3;B7Z463;A6N	16;16;13;6;5;5;4;4;3;2	Puromycin-sensitive aminopeptidase	NPEPPS	21.9	103.28	919	2.0593	0.79928	7	-0.323227105
P09132	2	Signal recognition particle 19 kDa protein	SRP19	11.1	16.156	144	1.7554	0.79923	2	-0.323317358
E9PEB5;Q96AE4-2;Q96AE4;	21;21;21;19;7	Far upstream element-binding protein 1	FUBP1	31.9	68.903	655	2.5922	0.79886	18	-0.323985401
Q9C037;Q9C037-2;Q9C037-	1;1;1;1	Tripartite motif-containing protein 4	TRIM4	2.2	57.46	500	1.8005	0.79872	6	-0.324238256
P68431	7	Histone H3.1	HIST1H3A	48.5	15.404	136	2.1581	0.78943	8	-0.341116749
Q96BR5	7	Sel1 repeat-containing protein 1	SELRC1	31.2	25.709	231	1.9943	0.78868	9	-0.342488037
P62140;E7ETD8;C9JP48;C9J	15;10;7;7	Serine/threonine-protein phosphatase PP1-beta catalyti	PPP1CB	52.6	37.186	327	1.8905	0.78864	5	-0.342561209
O75436;F5H4L7;G3V1N8;A8	4;4;4;2	Vacuolar protein sorting-associated protein 26A	VPS26A	15.9	38.169	327	1.9205	0.78799	3	-0.343750774
Q9BXS5-2;Q9BXS5;E7ENJ6;	8;8;8;7;6;3;2;2;2;2;2;2	AP-1 complex subunit mu-1	AP1M1	20.5	49.84	435	1.9563	0.78791	6	-0.343897249
Q14839-2;Q14839;F5GWX5	74;74;74;17;14;14;14	Chromodomain-helicase-DNA-binding protein 4	CHD4	44.5	220.85	1940	1.694	0.78787	106	-0.343970493
Q8N8C4;Q9BSY4;F8WC14	5;5;2	Coiled-coil-helix-coiled-coil-helix domain-containing pro	CHCHD5	43.7	17.713	158	2.2285	0.78443	7	-0.350283383
B4DEG8;P26639;G3XAN9;E7	18;18;16;16;2;2;2;2;2	Threonine--tRNA ligase, cytoplasmic	TARS	27.1	86.86	756	2.1486	0.78403	17	-0.351019236
O15031;E2PU09;A6QRG9;A	53;15;9;4;2;2	Plexin-B2	PLXNB2	42.3	205.12	1838	2.4638	0.78398	67	-0.351111244
P61204;P84077;F5H423;F5H	8;8;7;5;4;1	ADP-ribosylation factor 3;ADP-ribosylation factor 1	ARF3;ARF1	54.7	20.601	181	2.1259	0.78377	4	-0.351497742
Q53GQ0;E9PI21;Q96EA9	19;6;4	Estradiol 17-beta-dehydrogenase 12	HSD17B12	73.1	34.324	312	2.2726	0.78369	49	-0.351645007
P20042	5	Eukaryotic translation initiation factor 2 subunit 2	EIF2S2	19.5	38.388	333	1.8671	0.77978	3	-0.358860942
Q9UIJ7;E7ET30;Q9UIJ7-2	15;13;12	GTP:AMP phosphotransferase, mitochondrial	AK3	67.8	25.565	227	1.9584	0.77973	16	-0.358953452
Q15365;F8VTZ0	12;1	Poly(rC)-binding protein 1	PCBP1	56.2	37.497	356	2.0877	0.77835	22	-0.361509058
Q9ULP9;Q9ULP9-2;H3BQ06	7;7;4;1	TBC1 domain family member 24	TBC1D24	16.6	62.919	559	1.7901	0.7773	7	-0.363456579
O75153;K7EIG1;I3L4B5;Q9Y	23;23;2;1;1;1	Protein KIAA0664	KIAA0664	22.6	146.67	1309	1.9283	0.77704	14	-0.363939228
P49411;H3BNU3	29;2	Elongation factor Tu, mitochondrial	TUFM	69.2	49.541	452	2.4847	0.77683	70	-0.364329178
P16949;P16949-2;A2A2D0	4;2;2	Stathmin	STMN1	24.2	17.302	149	1.5298	0.77645	3	-0.365035071
O75822;F5H425;B4DUI3;H0	4;3;3;1;1	Eukaryotic translation initiation factor 3 subunit J	EIF3J	11.6	29.062	258	1.8515	0.77524	2	-0.367285084
P31937;H7BZL2	11;5	3-hydroxyisobutyrate dehydrogenase, mitochondrial	HIBADH	48.2	35.329	336	1.8911	0.77522	4	-0.367322303
Q9UJZ1;B4E1K7;F2Z2I8	6;5;2	Stomatin-like protein 2	STOML2	27	38.534	356	2.0479	0.77284	5	-0.371758329
G5E9M5;Q12906;Q12906-6;	31;30;29;29;28;28;28	Interleukin enhancer-binding factor 3	ILF3	41.9	95.807	898	2.6783	0.77284	34	-0.371758329
Q9BQG0-2;Q9BQG0;I3L1L3;	16;16;14;2	Myb-binding protein 1A	MYBBP1A	15.5	149.37	1332	1.739	0.77192	14	-0.373476757
P42766;F2Z388;H0Y3A0	3;2;2	60S ribosomal protein L35	RPL35	26	14.551	123	1.8995	0.77173	5	-0.373831905
Q9Y295	6	Developmentally-regulated GTP-binding protein 1	DRG1	22.6	40.542	367	1.6958	0.77081	2	-0.375552807
E9PR30;P62861	3;3	40S ribosomal protein S30	FAU	12.2	10.905	98	1.5016	0.77006	4	-0.376957236

P68104;Q5VTE0;Q5JR01;A6	21;20;5;4	Elongation factor 1-alpha 1;Putative elongation factor 1-a	EEF1A1;EEF1A1P5	63.4	50.14	462	1.9367	0.76993	105	-0.377200809
Q92667;Q92667-2;I3NI36;I3	2;1;1;1	A-kinase anchor protein 1, mitochondrial	AKAP1	4.1	97.34	903	2.0592	0.76916	4	-0.378644357
P22695;H3BRG4;H3BSJ9;H3	7;6;5;2;2;2	Cytochrome b-c1 complex subunit 2, mitochondrial	UQCRC2	20.8	48.442	453	2.1059	0.7669	4	-0.382889625
Q92878-2;Q92878;Q92878-	35;35;32;17;14;5;3;3;	DNA repair protein RAD50	RAD50	28.9	154.59	1318	2.2628	0.76668	31	-0.383303549
P48728;E9PBG1;H0Y695;P4	3;3;3;3;1;1	Aminomethyltransferase, mitochondrial;Aminomethyltr	AMT	10.4	43.946	403	1.6444	0.7665	3	-0.383642303
P55884-2;P55884;C9JZG1;C	32;32;10;8	Eukaryotic translation initiation factor 3 subunit B	EIF3B	46.3	99.028	873	2.2595	0.76626	31	-0.384094098
Q6NVY1;Q6NVY1-2;B9A05	18;13;9;7;7;4;2;2	3-hydroxyisobutyryl-CoA hydrolase, mitochondrial	HIBCH	54.9	43.482	386	1.9447	0.76623	5	-0.384150583
B0QY89;Q9Y262;J3KN35;G8	18;18;17;17;11;8;8;5;	Eukaryotic translation initiation factor 3 subunit L	EIF3EIP;EIF3L	36.2	70.901	607	1.8146	0.76574	18	-0.385073473
Q9Y224;G3V4C6;H0YJB9;G3	10;6;5;1	UPF0568 protein C14orf166	C14orf166	50.8	28.068	244	1.5734	0.76471	18	-0.387015355
Q7L2H7;B4E2Q4;H0YCQ8;J3	12;9;8;7;3;3;1;1	Eukaryotic translation initiation factor 3 subunit M	EIF3M	47.9	42.502	374	1.8164	0.76455	14	-0.387317241
P10809;E7ESH4;E7EXB4;B7Z	47;14;13;4;3;2;2;1	60 kDa heat shock protein, mitochondrial	HSPD1	79.6	61.054	573	1.9142	0.76281	149	-0.390604338
P22413;E9PE72	15;4	Ectonucleotide pyrophosphatase/phosphodiesterase far	ENPP1	24.6	104.92	925	2.3253	0.76255	14	-0.391096157
P16435;H0Y4R2;E7EVY7;F5I	35;33;30;28;25;19;2;1	NADPH--cytochrome P450 reductase	POR	65.6	76.689	677	2.3158	0.76255	58	-0.391096157
Q9UKF6;G5E9W3;C9JZH6	6;5;1	Cleavage and polyadenylation specificity factor subunit 3	CPSF3	13.3	77.485	684	1.7675	0.7617	5	-0.392705199
Q96HQ2;Q96HQ2-2	4;4	CDKN2AIP N-terminal-like protein	CDKN2AIPNL	36.2	13.196	116	2.38	0.76149	4	-0.393103004
P35606;B4DZI8;D6R997;D6I	28;28;5;5;4;4;4;4;2	Coatomer subunit beta	COPB2	41.2	102.49	906	2.5336	0.75867	24	-0.398455604
Q9NP72;Q9NP72-2;H0Y6T8	9;8;5;5;4	Ras-related protein Rab-18	RAB18	54.4	22.977	206	2.3019	0.75734	10	-0.400986966
Q8TDN6	2	Ribosome biogenesis protein BRX1 homolog	BRIX1	5.1	41.401	353	1.5186	0.7573	2	-0.401063166
P30519;I3L430;I3L1F5;I3L15	12;11;8;8;5;5;5;4	Heme oxygenase 2	HMOX2	52.2	36.032	316	2.0664	0.75721	13	-0.401234631
Q5T440;G5EA38	9;6	Putative transferase CAF17, mitochondrial	IBA57	37.9	38.155	356	1.9077	0.75626	9	-0.403045781
Q92692;Q92692-2;K7EKE8	6;6;5	Poliovirus receptor-related protein 2	PVRL2	12.8	57.741	538	2.0009	0.75539	10	-0.40470641
Q13451;B7Z7Z8	5;5	Peptidyl-prolyl cis-trans isomerase FKBP5	FKBP5	10.3	51.212	457	1.911	0.75514	5	-0.405183956
I3L4Q0;C9JLW8;I3L2L5;I3L3	2;2;2;1;1	Protein FAM195B	FAM195B	24.6	12.95	118	1.5413	0.75412	2	-0.407133983
P12268;H0Y4R1;E7ETK5	10;10;4	Inosine-5-monophosphate dehydrogenase 2	IMPDH2	25.9	55.804	514	1.9021	0.75351	10	-0.408301437
P31947;P31947-2	5;5	14-3-3 protein sigma	SFN	17.7	27.774	248	2.4634	0.75088	2	-0.413345729
Q8WVM8;B7Z738;B7Z4U7;I	12;10;10;10;9;3;2;2;2	Sec1 family domain-containing protein 1	SCFD1	27.4	72.379	642	1.7121	0.74959	16	-0.415826388
J3KTA4;P17844;B4DLW8;J3	24;24;22;10;9;8;6;5;1	Probable ATP-dependent RNA helicase DDX5	DDX5	40.7	69.086	614	2.264	0.7489	18	-0.417155005
Q13868;A3KFL5;A3KFL4;A3	3;3;3;2;2;1	Exosome complex component RRP4	EXOSC2	12.3	32.789	293	1.7753	0.74485	2	-0.424978174
B1ANS9;B1ANS9-2;H0Y6L4;	2;1;1;1;1	WD repeat-containing protein 64	WDR64	1.3	123.63	1081	2.202	0.74424	2	-0.426160163
Q9NTJ5;B4DK71;E9PGZ4;F8	16;13;13;7;7;4;1;1	Phosphatidylinositide phosphatase SAC1	SACM1L	26.9	66.966	587	2.1021	0.7423	16	-0.429925726
Q9Y4B6;Q9Y4B6-2;H0Y4P3;	4;4;4;2	Protein VPRBP	VPRBP	3	169.01	1507	1.7415	0.74217	3	-0.430178409
Q99720;Q99720-3;Q99720-	3;2;2;1;1;1	Sigma non-opioid intracellular receptor 1	SIGMAR1	17.5	25.127	223	2.274	0.74208	4	-0.43035337
P63010-2;P63010;Q7Z451;K	27;27;26;23;6;5;3;2;1	AP-2 complex subunit beta	AP2B1	33.1	105.69	951	2.1996	0.74207	9	-0.430372811
Q13884;Q13884-2;Q13424;I	11;8;1;1;1;1	Beta-1-syntrophin	SNTB1	23.2	58.06	538	2.0717	0.74187	12	-0.430761693
Q5VZK9;Q5VZK9-2;Q5VZK9	5;5;3;2	Leucine-rich repeat-containing protein 16A	LRRC16A	4.9	151.56	1371	1.7925	0.74151	3	-0.431461946
E7EUC7;Q16851;Q16851-2;(	10;10;10;4;3;3;3;2;2;2	UTP--glucose-1-phosphate uridylyltransferase	UGP2	22.6	57.805	517	1.921	0.74079	6	-0.432863471
P40227;B4DPJ8;A6NCD2;J3	25;23;22;2	T-complex protein 1 subunit zeta	CCT6A	55.2	58.024	531	2.0169	0.74077	30	-0.432902422
P62273;P62273-2	4;3	40S ribosomal protein S29	RPS29	48.2	6.6767	56	1.2701	0.74001	7	-0.434383328
Q9HB71;Q5R370;Q9HB71-2	13;10;3;3	Calcyclin-binding protein	CACYBP	68.4	26.21	228	1.765	0.73901	9	-0.436334208
P38117-2;P38117	16;16	Electron transfer flavoprotein subunit beta	ETFB	43.9	37.434	346	1.9906	0.73841	25	-0.437506004
Q9Y3I0;E7EQS9	9;2	tRNA-splicing ligase RtcB homolog	C22orf28	24.4	55.21	505	1.8185	0.73776	7	-0.438776524
P23526;F5H737	15;14	Adenosylhomocysteinase	AHCY	40	47.716	432	2.1879	0.73747	11	-0.439343733
Q32P28-3;Q32P28;Q32P28-	8;8;3;2;2;2	Prolyl 3-hydroxylase 1	LEPRE1	10.7	90.615	804	2.0698	0.73743	6	-0.439421986
Q9BPX5	3	Actin-related protein 2/3 complex subunit 5-like protein	ARPC5L	21.6	16.941	153	2.483	0.73674	2	-0.440772522
P17066;P48741	9;6	Heat shock 70 kDa protein 6;Putative heat shock 70 kDa p	HSPA6;HSPA7	14.6	71.027	643	2.4083	0.73621	4	-0.441810749
Q9UIW2;F8VSZ4;Q9HCM2;I	10;10;3;3;3	Plexin-A1	PLXNA1	5.9	211.06	1896	1.6425	0.7352	7	-0.443791328
B3KS98;O15372;E5RJT0;E5F	8;8;4;4;3;3;1;1	Eukaryotic translation initiation factor 3 subunit H	EIF3S3;EIF3H	33.6	41.581	366	1.8972	0.73516	8	-0.443869823
Q14974;B7ZAV6;J3KTM9;F5	40;33;31;31;7;7;1;1	Importin subunit beta-1	KPNB1	62	97.169	876	2.5097	0.73457	68	-0.445028118
P05362;K7EKL8;E7ESS4	19;9;8	Intercellular adhesion molecule 1	ICAM1	34.2	57.825	532	2.6456	0.73101	29	-0.452036953
P48643;E9PCA1;B7ZAR1;E7	29;27;27;26;24;2;1	T-complex protein 1 subunit epsilon	CCT5	66.9	59.67	541	2.0199	0.73056	34	-0.45292533
Q13442	2	28 kDa heat- and acid-stable phosphoprotein	PDAP1	16	20.63	181	0.82506	0.72796	2	-0.458068916
B3KSH1;O00303;B4DEW9;H	6;6;4;3;1	Eukaryotic translation initiation factor 3 subunit F	EIF3F	21.8	39.146	372	2.2175	0.72775	5	-0.458485161
P22234;P22234-2;E9PBS1;D	20;19;19;15	Multifunctional protein ADE2;Phosphoribosylaminoimid	PAICS	55.1	47.079	425	2.0551	0.72759	23	-0.45880238
Q9NYJ1-2;Q9NYJ1	4;4	Coiled-coil-helix-coiled-coil-helix domain-containing pro	CHCHD8	31.2	11.308	96	1.5015	0.72751	4	-0.458961016
O75607	4	Nucleoplasmin-3	NPM3	35.4	19.343	178	2.3974	0.72731	5	-0.459357682
Q9NZI8;C9JT33;G8JLG2;Q1	22;16;2;2;2;1	Insulin-like growth factor 2 mRNA-binding protein 1	IGF2BP1	42.5	63.48	577	2.3	0.72506	34	-0.463827709
O75051;O75051-2	7;2	Plexin-A2	PLXNA2	3.8	211.1	1894	1.1432	0.72408	2	-0.465778993
P49368;B4DUR8;P49368-2;(	29;28;27;11;10;7;7;6;	T-complex protein 1 subunit gamma	CCT3	70.5	60.533	545	2.0137	0.72391	31	-0.466117749
P14866;P14866-2;H7C464	21;17;1	Heterogeneous nuclear ribonucleoprotein L	HNRNPL	59.6	64.132	589	2.3026	0.72386	31	-0.466217399
O15230;F2Z2S4;E2QRK0;Q5	30;3;2;2	Laminin subunit alpha-5	LAMA5	10.5	399.73	3695	1.4595	0.7237	24	-0.466536323
A6NMQ1;P09884	5;5	DNA polymerase;DNA polymerase alpha catalytic subuni	POLA1	4	166.46	1468	1.3027	0.72169	4	-0.47054883
P07108-5;P07108-4;P07108	5;5;5;5;5;1	Acyl-CoA-binding protein	DBI	36.5	16.495	148	1.1821	0.72168	7	-0.470568821
Q15024	2	Exosome complex component RRP42	EXOSC7	9.3	31.821	291	1.8776	0.7209	2	-0.472128946
Q96EL2	3	28S ribosomal protein S24, mitochondrial	MRPS24	17.4	19.015	167	2.1985	0.7206	4	-0.472729443
P51648-2;P51648;J3QRD1;I	19;19;16;8;4;4;4;4;3;3	Fatty aldehyde dehydrogenase	ALDH3A2	40.6	57.669	508	2.1175	0.72043	25	-0.473069836
P43246;E9PHA6;B4E2Z2;C9	20;19;19;3	DNA mismatch repair protein Msh2	MSH2	26.9	104.74	934	1.8625	0.71959	12	-0.474752957
Q9UBQ5;B7ZAM9;K7ERF1;I	7;7;5;5;5;4;2;2	Eukaryotic translation initiation factor 3 subunit K	EIF3K	44.5	25.059	218	2.014	0.71951	6	-0.474913357
Q8TCS8;H7BZ02;H7C3C5	8;5;2	Polyribonucleotide nucleotidyltransferase 1, mitochondi	PNPT1	14.4	85.95	783	1.8098	0.71947	6	-0.474993563
E9PCR7;Q02218;E9PDF2;Q(	28;28;27;27;27;23;12	2-oxoglutarate dehydrogenase, mitochondrial	OGDH	32.8	117.66	1038	2.0157	0.71811	16	-0.477723242
Q7Z3U7;Q7Z3U7-4;B9EGP5	9;9;9;9;9;9;5;3;1	Protein MON2 homolog	MON2	7	190.48	1718	1.6506	0.71587	8	-0.482230473
P42677;Q5T4L4;C9J1C5	5;4;1	40S ribosomal protein S27	RPS27	40.5	9.461	84	1.4908	0.71497	7	-0.484045387
Q86X55;Q86X55-1;Q86X55-	7;7;4;3;1;1;1	Histone-arginine methyltransferase CARM1	CARM1	13.7	65.853	608	1.7133	0.71397	5	-0.486064639
B7Z452;P33121;P33121-2;E	10;10;10;10;9;4;4;2	Long-chain-fatty-acid--CoA ligase 1	ACSL1	15.6	77.906	698	1.7122	0.71368	7	-0.486650752
P63172;Q5VTU3	4;3	Dynein light chain Tctex-type 1	DYNLT1	58.4	12.452	113	1.2457	0.71248	4	-0.489078578
Q8NBQ5;D6RCD0	14;13	Estradiol 17-beta-dehydrogenase 11	HSD17B11	52.7	32.935	300	2.1127	0.71153	20	-0.491003509
P13667;C9JMN9	49;2	Protein disulfide-isomerase A4	PDIA4	66.5	72.932	645	2.3096	0.71104	92	-0.491997373
P34741;E9PBI9;E7ESK6;E5R	3;3;3;2	Syndecan-2;Syndecan	SDC2	15.9	22.16	201	1.2422	0.71076	3	-0.492565603
P00167;P00167-2;F8WEU4;	6;6;5;1	Cytochrome b5	CYB5A	56	15.33	134	1.7093	0.70994	14	-0.494230993
P07814	73	Bifunctional glutamate/proline--tRNA ligase;Glutamate-	EPRS	60.9	170.59	1512	2.1223	0.70952	89	-0.495084743
B4DUC8;Q13126;Q13126-2;	4;4;3;3;3;2;2;2;2;1	S-methyl-5-thioadenosine phosphorylase	MTAP	23	33.186	300	1.8074	0.7092	3	-0.495735559
P62081;B5MCP9	12;8	40S ribosomal protein S7	RPS7	65.5	22.127	194	1.4882	0.7089	14	-0.496345965
Q9BT09	4	Protein canopy homolog 3	CNPY3	19.8	30.748	278	1.9192	0.70735	2	-0.499503851
Q00610;Q00610-2;J3KS13;K	102;102;29;11;7;7;5;1	Clathrin heavy chain 1	CLTC	72.6	191.61	1675	2.3134	0.70651	181	-0.501218114
Q9HBH1	4	Peptide deformylase, mitochondrial	PDF	16.5	27.013	243	1.5345	0.70482	3	-0.504673232
P17987;E7ERF2;E7EQR6;F5I	29;19;19;17;8;8;6;5;5	T-complex protein 1 subunit alpha	TCP1	70	60.343	556	2.1964	0.70481	37	-0.504693701
O15355	10	Protein phosphatase 1G	PPM1G	21.6	59.271	546	1.4767	0.70404	3	-0.506270697
P09327;B4DV78;Q96AC8;H	29;19;10;10;3;1;1	Villin-1	VIL1	48.9	92.694	827	2.4902	0.70369	27	-0.506988084
O60888-2;O60888;O60888-	3;3;3;2	Protein CutA	CUTA	27.8	20.925	198	2.255	0.70216	4	-0.510128282
P04626;P04626-4;B4DTR1;J	9;9;9;8;7;7;3;2;1;1;1;	Receptor tyrosine-protein kinase erbB-2	ERBB2	8.6	137.91	1255	1.6282	0.69978	3	-0.515026663
P53992;G5EA31;E7EP00;B4I	13;13;13;2	Protein transport protein Sec24C	SEC24C	14.7	118.32	1094	1.8217	0.69942	8	-0.515769044
Q07812-2;Q07812;Q07812-	9;9;9;8;7;6;4;4;1	Apoptosis regulator BAX	BAX	52.3	24.22	218	1.5316	0.69875	12	-0.517151717
P19784;H3BSA1;H3BV19;H3	9;6;4;3	Casein kinase II subunit alpha	CSNK2A2	34.6	41.213	350	1.8797	0.69811	9	-0.518473718
Q9BQA1;H0Y711;B4DP38	6;4;4	Methylosome protein 50	WDR77	24	36.724	342	1.6579	0.69799	5	-0.518721728
P78371;P78371-2;F5GWF6;	35;33;32;29	T-complex protein 1 subunit beta	CCT2	78.7	57.488	535	2.0305	0.69799	41	-0.518721728
P08195-4;J3KPF3;P08195;F	25;25;25;25;25;24;8;8	4F2 cell-surface antigen heavy chain	SLC3A2	41	71.122	661	2.5771	0.6959	30	-0.523048087
P35268;K7ERI7;K7EMH1;K7	6;5;4;4;4;4;3	60S ribosomal protein L22	RPL22	53.1	14.787	128	2.3445	0.69559	12	-0.523690903
F5H3A1;P05023;P05023-3;F	44;44;39;29;16;16;16	Sodium/potassium-transporting ATPase subunit alpha-1	ATP1A1	45.1	113	1023	2.4396	0.69433	80	-0.526306587
P62993;J3KT38;P62993-2;J3	10;7;6;5;1	Growth factor receptor-bound protein 2	GRB2	41	25.206	217	1.5942	0.69178	8	-0.53161479
P11216;P11217;P11217-2;E	8;4;4;3;1	Glycogen phosphorylase, brain form;Glycogen phosphor	PYGB;PYGM	11.6	96.695	843	1.4685	0.6911	7	-0.533033616
P84085;C9J1Z8	9;8	ADP-ribosylation factor 5	ARF5	60.6	20.529	180	1.7113	0.68931	5	-0.53677515
Q9GZZ1;E7EQ69;C9J5D1;Q9	3;3;3;3;2;2;1;1	N-alpha-acetyltransferase 50	NAA50	18.9	19.398	169	1.2587	0.68907	3	-0.537277547
P54886;P54886-2	30;30	Delta-1-pyrroline-5-carboxylate synthase;Glutamate 5-ki	ALDH18A1	48.4	87.301	795	2.2294	0.68779	35	-0.539959955
Q9C005;B4DIS3	3;2	Protein dpy-30 homolog	DPY30;LOC84661	45.5	11.25	99	1.9526	0.68632	4	-0.543046698
Q9Y5A9;Q9Y5A9-2;Q7Z739;	2;2;1;1;1	YTH domain family protein 2;YTH domain family protein 3	YTHDF2;YTHDF3;Y	3.8	62.333	579	1.303	0.68462	2	-0.546624656
P62913;P62913-2;Q5VVC8;	12;11;10;5	60S ribosomal protein L11	RPL11	55.6	20.252	178	2.2041	0.68406	10	-0.547805223
J3KN41;P23229;P23229-6;P	8;8;8;8;8;8;8;8;8;6;	Integrin alpha-6;Integrin alpha-6 heavy chain;Integrin alp	ITGA6	6.3	126.6	1130	1.7804	0.683	6	-0.550042516
O43752;B4DR17	2;1	Syntaxin-6	STX6	14.5	29.176	255	1.5327	0.68202	2	-0.552114048
O15270;H0YJV2	2;1	Serine palmitoyltransferase 2	SPTLC2	6.9	62.924	562	1.1439	0.67993	2	-0.556541869
Q9UJ83;B4DWI1;E9PEN4;B4	12;12;11;10;7;5;4;2;2	2-hydroxyacyl-CoA lyase 1	HACL1	28.9	63.728	578	1.6037	0.67923	13	-0.558027914
P51970;F5H7V7	6;5	NADH dehydrogenase [ubiquinone] 1 alpha subcomplex	NDUFA8	51.7	20.105	172	1.5313	0.67906	6	-0.558389042
Q9Y3B2;B1AMU7;B1AMU4;	3;3;2;2	Exosome complex component CSL4	EXOSC1;RP11-452	22.6	21.452	195	1.3257	0.67839	3	-0.559813191
Q02338;E9PCG9;C9JQ90;C9	2;1;1;1;1;1;1	D-beta-hydroxybutyrate dehydrogenase, mitochondrial	BDH1	6.1	38.157	343	1.1034	0.67826	2	-0.560089682
Q99627;Q99627-2;B8ZZP3;I	5;5;4;4;3	COP9 signalosome complex subunit 8	COPS8	44.5	23.225	209	2.1144	0.67731	4	-0.562111799
D6REX3;O94979-8;O94979;	28;28;28;28;28;28;28	Protein transport protein Sec31A	SEC31A	28.7	136.22	1251	2.0567	0.67701	31	-0.562750951
Q8NBX0	12	Saccharopine dehydrogenase-like oxidoreductase	SCCPDH	47.8	47.151	429	1.6823	0.67673	21	-0.563347749
P68402;P68402-4;J3KNE3;P	2;1;1;1;1	Platelet-activating factor acetylhydrolase IB subunit beta	PAFAH1B2	12.2	25.569	229	1.2042	0.67659	2	-0.563646241
P07306;P07306-2;J3QSZ2;K	14;12;9;7;6;4;2	Asialoglycoprotein receptor 1	ASGR1	53.6	33.186	291	1.9412	0.67642	22	-0.564008778
A6NCS6	7	Uncharacterized protein C2orf72	C2orf72	34.6	30.48	295	1.8997	0.67558	8	-0.565801476
Q15738;C9JDR0	14;10	Sterol-4-alpha-carboxylate 3-dehydrogenase, decarboxy	NSDHL	56.3	41.9	373	1.7704	0.67481	15	-0.567446742
O00233;J3KN29;O00233-2;I	2;2;2;2;2;2;2;1;1	26S proteasome non-ATPase regulatory subunit 9	PSMD9	9.4	24.682	223	1.4399	0.6746	2	-0.567895777
P10586;P10586-2;H0Y6Z7;H	26;26;22;16;13;10;4;3	Receptor-type tyrosine-protein phosphatase F	PTPRF	18.1	212.88	1907	2.1247	0.67389	21	-0.569414977
Q14204;H0YJ21	232;12	Cytoplasmic dynein 1 heavy chain 1	DYNC1H1	58.3	532.4	4646	1.595	0.67268	282	-0.57200773
P22102;B4DJ93;P22102-2;F	11;6;5;1;1;1;1;1;1	Trifunctional purine biosynthetic protein adenosine-3;Pl	GART	16.4	107.77	1010	1.9223	0.67256	6	-0.572265117
Q92616	99	Translational activator GCN1	GCN1L1	46.6	292.75	2671	1.6179	0.67189	126	-0.573703037
Q99832;Q99832-3;B8ZZC9;(	28;26;25;23;23;20;18	T-complex protein 1 subunit eta	CCT7	60.2	59.366	543	1.9134	0.67116	37	-0.575271359
Q15369;E5RHG8;E5RGD9;E5	5;5;4;3	Transcription elongation factor B polypeptide 1	TCEB1	57.1	12.473	112	1.5192	0.67041	9	-0.576884426
F8W930;Q9Y6M1;Q9Y6M1-	10;10;10;7;7;7;7	Insulin-like growth factor 2 mRNA-binding protein 2	IGF2BP2	21.3	66.785	605	1.6911	0.67033	8	-0.577056593
Q9BRX8;Q9BRX8-2	11;11	Redox-regulatory protein FAM213A	FAM213A	43.2	25.764	229	1.9393	0.669	15	-0.579921884
Q16762;B1AH48	5;1	Thiosulfate sulfurtransferase	TST	20.9	33.429	297	1.8795	0.66823	4	-0.581583341
Q9UNK0;K7EQB1;I3L305	4;3;1	Syntaxin-8	STX8	21.6	26.906	236	2.0415	0.66778	4	-0.58255521
P06748;P06748-2;P06748-3	14;14;13;3;2	Nucleophosmin	NPM1	59.5	32.575	294	1.2889	0.66719	74	-0.583830429

P23193;P23193-2;B7Z4S1;E	4;2;2;2;2;1;1;1	Transcription elongation factor A protein 1	TCEA1	14	33.969	301	1.8173	0.66704	4	-0.584154818
Q16836-2;J3KQ17;Q16836;I	12;12;12;11	Hydroxyacyl-coenzyme A dehydrogenase, mitochondrial	HADH	54.1	42.139	390	2.0486	0.66605	17	-0.586297611
A4D1W8;Q9UM22;C9JYS3;I	4;4;3;2;1	Mammalian ependymin-related protein 1	UCC1;EPDR1	11.9	38.15	344	1.3916	0.66428	4	-0.590136616
O15042;O15042-2;E7ET15;C	20;20;19;13;12;7;6;3;	U2 snRNP-associated SURP motif-containing protein	U2SURP	25.5	118.29	1029	1.364	0.6636	26	-0.591614209
P00439;J3KND8;F8W0A0;F8	4;3;1;1	Phenylalanine-4-hydroxylase	PAH	14.4	51.861	452	1.3944	0.66249	4	-0.594029417
Q05048	2	Cleavage stimulation factor subunit 1	CSTF1	9	48.357	431	1.8511	0.66226	2	-0.594530372
P13639	47	Elongation factor 2	EEF2	62	95.337	858	2.3928	0.65964	73	-0.60024921
Q14152;F5H335	62;57	Eukaryotic translation initiation factor 3 subunit A	EIF3A	46.3	166.57	1382	2.0686	0.65807	69	-0.603687041
Q9BRG1;K7EKV4;K7ENE3	2;2;1	Vacuolar protein-sorting-associated protein 25	VPS25	14.2	20.747	176	1.2361	0.65799	2	-0.603862437
P07737;K7EJ44;I3L3D5	13;9;5	Profilin-1	PFN1	91.4	15.054	140	1.8492	0.6577	39	-0.604498425
Q16698;B7Z6B8;E5RFV2;E5	12;12;7;6;5;3;1	2,4-dienoyl-CoA reductase, mitochondrial	DECR1	56.4	36.067	335	1.9971	0.65769	12	-0.60452036
A9R9N7;H3BM16;P01892;F	17;17;17;17;17;17;7;2	HLA class I histocompatibility antigen, A-2 alpha chain	HLA-A	51.5	41.449	371	1.7894	0.65613	24	-0.607946408
P09496;P09496-3;P09496-4	3;3;3;3;2;2;2	Clathrin light chain A	CLTA	15.3	27.076	248	1.8877	0.65448	3	-0.611578989
P30837;P47895;H0YKF9	14;3;1	Aldehyde dehydrogenase X, mitochondrial	ALDH1B1	35.8	57.206	517	1.7653	0.65409	8	-0.612438937
P23919;H7BZ20;H7C312;G5	13;7;7;4;2	Thymidylate kinase	DTYMK	63.7	23.819	212	1.5601	0.65343	12	-0.613895402
P17301;E7ESP4;E7EMF1;E9	30;26;20;13;3	Integrin alpha-2	ITGA2	31.6	129.29	1181	2.2359	0.64981	33	-0.621910149
P05091;E7EUE5;F8W0A9;F8	28;24;24;23;10	Aldehyde dehydrogenase, mitochondrial	ALDH2	70.8	56.381	517	1.7742	0.64945	19	-0.622709635
Q9BRQ8;Q9BRQ8-2	8;7	Apoptosis-inducing factor 2	AIFM2	22.3	40.526	373	1.6671	0.64907	7	-0.623554019
Q96AG4;I3L223	13;2	Leucine-rich repeat-containing protein 59	LRRC59	47.6	34.93	307	1.949	0.64869	15	-0.624398896
Q9H078;Q9H078-2;Q9H078	5;5;5;4;4;4;3;3;3;1;1	Caseinolytic peptidase B protein homolog	CLPB	11	78.728	707	1.6299	0.64517	5	-0.632248739
Q9P2J5;B4DER1;F5H698;B4	22;22;22;21	Leucine--tRNA ligase, cytoplasmic	LARS	24.6	134.46	1176	1.9657	0.64486	23	-0.632942111
Q9UHB9;G3V1U4;Q9UHB9-	9;9;8;6;2;2	Signal recognition particle 68 kDa protein	SRP68	20.6	70.729	627	0.72882	0.64304	3	-0.637019612
P62263;E5RH77;H0YB22	10;8;7	40S ribosomal protein S14	RPS14	68.2	16.273	151	1.2828	0.64206	15	-0.639219973
Q12996;E9PLP8;Q12996-2	9;2;1	Cleavage stimulation factor subunit 3	CSTF3	16	82.921	717	1.8748	0.64068	9	-0.64232414
P50990;G5E9B2;B4DEM7;B4	33;32;32;27;16;6	T-complex protein 1 subunit theta	CCT8	70.3	59.62	548	2.0226	0.64019	37	-0.643427953
P46783;F6U211;Q9NQ39	6;4;2	40S ribosomal protein S10	RPS10	38.2	18.898	165	1.846	0.63908	16	-0.645931556
P11172;E9PFD2;F8WDG4;F.	3;3;2;2;2;1	Uridine 5-monophosphate synthase;Orotate phosphorib	UMPS	6	52.221	480	1.3943	0.63484	2	-0.655535063
J3QRU1;P07947	6;6	Tyrosine-protein kinase Yes	YES1	13.9	61.386	548	1.584	0.63348	4	-0.658629023
Q5T5C7;P49591	5;5	Serine--tRNA ligase, cytoplasmic	SARS	11.2	61.312	536	1.2653	0.63291	5	-0.659927732
Q00535;Q00535-2	4;3	Cyclin-dependent kinase 5	CDK5	12.7	33.304	292	1.227	0.6319	3	-0.662231829
O95816;B4DXE2;A2A296	10;9;4	BAG family molecular chaperone regulator 2	BAG2	41.2	23.772	211	1.564	0.63183	8	-0.662391655
Q8IVM8;Q8IVM8-2	2;1	Solute carrier family 22 member 9	SLC22A9	4.2	62.169	553	1.3769	0.62981	2	-0.66701143
P58546;C9JL85	6;4	Myotrophin	MTPN	67.8	12.895	118	1.067	0.62966	5	-0.667355074
P56192;B3KVK7;A6NC17;H	25;17;16;7;5;5;3;3;3;	Methionine--tRNA ligase, cytoplasmic	MARS	43.2	101.11	900	1.9171	0.62942	25	-0.667905073
O75390;B4DJV2;H0YIC4;H0	20;20;7;6;6;6;5;5;5;4;	Citrate synthase, mitochondrial;Citrate synthase	CS	57.9	51.712	466	2.026	0.62773	23	-0.671783936
O95486;O95486-2	7;3	Protein transport protein Sec24A	SEC24A	6.9	119.75	1093	1.1228	0.62708	8	-0.673278588
P15880;H0YEN5;E9PQD7;E9	16;13;11;11;10;10;9;2	40S ribosomal protein S2	RPS2	54.6	31.324	293	1.6161	0.62656	24	-0.674475425
P50897;E9PMG2;P50897-2;	2;1;1;1;1;1	Palmitoyl-protein thioesterase 1	PPT1	9.2	34.193	306	1.1228	0.62377	2	-0.680913926
P08708;P0CW22;H0YK46;H	8;8;7;7;4;2	40S ribosomal protein S17;40S ribosomal protein S17-like	RPS17;RPS17L	59.3	15.55	135	1.518	0.62363	21	-0.681237764
I3L0C1;Q96Q15;J3KRA9;Q9	2;2;2;2;2;1;1;1	Serine/threonine-protein kinase SMG1;Putative unchara	SMG1	0.6	410.62	3662	1.2905	0.6232	2	-0.682232861
REV__Q9H0J4;REV__Q9H0J	1;1;1			0.4	180.82	1663	1.67	0.62264	2	-0.683529833
Q8N183;D6RA56;H0YA50	7;3;1	Mimitin, mitochondrial	NDUFAF2	55	19.856	169	1.5155	0.62134	6	-0.686545161
P07910-2;P07910;G3V4C1;E	15;14;14;14;14;13;13	Heterogeneous nuclear ribonucleoproteins C1/C2	HNRNPC	49.8	32.337	293	1.909	0.62109	42	-0.687125755
P78417;D3DRA3;Q5TA02;F5	7;6;6;5;4	Glutathione S-transferase omega-1	GSTO1	27.8	27.566	241	1.5781	0.62101	10	-0.687311595
P25685;B4DX52;Q9UDY4	6;5;1	DnaJ homolog subfamily B member 1	DNAJB1	21.5	38.044	340	1.5144	0.62044	7	-0.688636394
P10619;Q5JZH0;Q5JZG9	3;1;1	Lysosomal protective protein;Lysosomal protective prote	CTSA	7.7	54.465	480	1.4907	0.62039	3	-0.688752663
J3KPT4;Q9H4I3;Q9H4I3-2	3;3;2	TraB domain-containing protein	TRABD	9.5	42.692	379	1.3317	0.62023	3	-0.689124785
P50991;B7Z9L0;F5H5W3;B7	25;24;22;17	T-complex protein 1 subunit delta	CCT4	61.4	57.924	539	1.8865	0.61991	28	-0.689869318
P51511;Q9Y5R2	4;1	Matrix metalloproteinase-15	MMP15	6.9	75.806	669	1.4223	0.61973	2	-0.690288287
P40926;E9PDB2;G3XAL0	15;13;11	Malate dehydrogenase, mitochondrial;Malate dehydroge	MDH2	59.2	35.503	338	2.186	0.61901	22	-0.691965379
F8VXU5;H3BLV8;Q9UBQ0-2	2;2;2;2	Vacuolar protein sorting-associated protein 29	VPS29	10.7	23.98	214	1.629	0.6189	2	-0.692221773
Q6ZRP7;H0Y430	6;4	Sulfhydryl oxidase 2	QSOX2	10.3	77.528	698	1.1533	0.61881	4	-0.692431584
B0QZ18;Q99829;F2Z2V0;A6	5;5;5;4;3;2;2;2;2;2;2;	Copine-1	CPNE1	12	59.717	542	1.145	0.61843	4	-0.693317789
Q9NZL9;Q9NZL9-2;Q9NZL9	5;5;5;3;2;1;1;1	Methionine adenosyltransferase 2 subunit beta	MAT2B	20.1	37.551	334	1.1989	0.61806	2	-0.694181196
P38919;I3L3F6;I3L2Z3;I3L3H	17;6;5;4;2;1;1;1;1	Eukaryotic initiation factor 4A-III	EIF4A3	44.8	46.871	411	1.6664	0.61752	17	-0.695442232
J3QL51;P18621;J3KRX5;J3Q	6;6;6;6;5;5;4;3;2	60S ribosomal protein L17	RPL17	33.8	26.372	228	1.2858	0.6158	8	-0.699466228
Q9UNW1;Q9UNW1-4;Q9UI	4;3;1;1	Multiple inositol polyphosphate phosphatase 1	MINPP1	9.9	55.051	487	1.2003	0.61541	3	-0.700380208
O76003	2	Glutaredoxin-3	GLRX3	11.6	37.432	335	1.178	0.61464	2	-0.702186436
Q9NPD3;E9PI41;E9PPI9	3;2;1	Exosome complex component RRP41	EXOSC4	14.7	26.383	245	1.8475	0.61408	4	-0.703501478
P31939;E9PBU3;F5GWY2;H	17;16;13;5;5;4;4;2	Bifunctional purine biosynthesis protein PURH;Phosphor	ATIC	43.6	64.615	592	1.5192	0.61362	14	-0.704582589
Q9NR30;Q9NR30-2;Q9BQ3	9;9;1	Nucleolar RNA helicase 2	DDX21	14.6	87.343	783	1.8136	0.61293	7	-0.706205775
P07203;E9PAS1	8;2	Glutathione peroxidase 1	GPX1	56.7	22.106	203	1.5682	0.61219	4	-0.707948616
P07741;H3BQB1;H3BQF1;G	6;6;6;6;4;4	Adenine phosphoribosyltransferase	APRT	41.7	19.608	180	1.6957	0.61216	6	-0.708019316
O15067;F5GWT9;H0YGH1;J	18;10;5;4;4;2;1;1;1	Phosphoribosylformylglycinamidine synthase	PFAS	19	144.73	1338	1.6063	0.61112	8	-0.710472398
Q13085-4;Q13085;Q13085-	7;7;7;7;6;2;2;2;2;2;1;	Acetyl-CoA carboxylase 1;Biotin carboxylase	ACACA	2.8	270	2383	0.98492	0.61064	3	-0.711605998
Q13576;E7EWC2;F5H7S7;J3	56;50;37;37;37;30;21	Ras GTPase-activating-like protein IQGAP2	IQGAP2	44.1	180.58	1575	1.873	0.61059	55	-0.711724133
P62899;B7Z4C8;P62899-2;P	8;7;7;7;7;7;7;5	60S ribosomal protein L31	RPL31	50.4	14.463	125	1.732	0.61048	10	-0.711984063
P55084;B4E2W0;F5GZQ3;B5	27;27;24;18;7;7;4	Trifunctional enzyme subunit beta, mitochondrial;3-keto	HADHB	63.5	51.294	474	1.7404	0.60895	41	-0.715604319
P47897;B4DWJ2;C9J165;B7	27;25;11;10;10;9;4	Glutamine--tRNA ligase	QARS	46.7	87.798	775	1.8001	0.6082	28	-0.717382279
P15311;E7EQR4	20;19	Ezrin	EZR	33.8	69.412	586	1.6381	0.6082	25	-0.717382279
P56537;B7ZBG9;B7ZBH1;F8	7;5;3;1;1	Eukaryotic translation initiation factor 6	EIF6	47.3	26.599	245	1.9666	0.60818	8	-0.717429721
Q8N163;Q8N163-2;H0YB24	22;22;15;14;5;4;4;4;3	DBIRD complex subunit KIAA1967	KIAA1967	29.7	102.9	923	1.8397	0.60814	25	-0.71752461
O00505;H0Y4S9	6;1	Importin subunit alpha-3	KPNA3	12.9	57.81	521	1.3803	0.60787	2	-0.718165275
P49327;J3KTF0	127;1	Fatty acid synthase;[Acyl-carrier-protein] S-acetyltransfe	FASN	69.2	273.42	2511	1.5969	0.60724	253	-0.719661268
B4DXJ1;P55735;A8MV37;A8	7;7;6;5;2	Protein SEC13 homolog	SEC13	30.4	40.746	368	1.7302	0.60632	8	-0.721848683
Q92896-2;Q92896;Q92896-	40;40;39;25;7;3;3	Golgi apparatus protein 1	GLG1	39.9	137.22	1203	1.8653	0.60486	43	-0.725326838
P62701;A6NH36;C9JQ55	17;6;2	40S ribosomal protein S4, X isoform	RPS4X	55.9	29.597	263	1.6468	0.60449	25	-0.726209622
Q8N5K1;D6RCF4;I3L1N9	7;3;3	CDGSH iron-sulfur domain-containing protein 2	CISD2	48.1	15.278	135	1.6239	0.60253	8	-0.73089502
J3KQG6;P15170-2;P15170;H	8;8;7;6;4;1	Eukaryotic peptide chain release factor GTP-binding subu	GSPT1;GSPT2	16.3	68.699	637	1.893	0.60138	3	-0.733651206
Q13825;Q13825-2;B4DYI6	2;2;1	Methylglutaconyl-CoA hydratase, mitochondrial	AUH	5	35.608	339	0.87707	0.60122	2	-0.734035093
P61457	5	Pterin-4-alpha-carbinolamine dehydratase	PCBD1	51.9	11.999	104	1.5894	0.59976	4	-0.737542788
Q9H7Z7;H7C5L1;A6NHH0	17;12;11	Prostaglandin E synthase 2;Prostaglandin E synthase 2 tru	PTGES2	61.3	41.943	377	1.5824	0.59938	20	-0.73845715
B0QYN7;P63279;H3BQQ9;H	4;4;4;4	SUMO-conjugating enzyme UBC9	UBE2I	17.4	20.457	184	1.3176	0.59888	5	-0.739661142
O60493;O60493-4;O60493-	2;2;2	Sorting nexin-3	SNX3	15.4	18.762	162	1.324	0.59854	2	-0.74048043
Q8WTV0-2;B7ZKQ9;Q8WTV	8;8;8;7;7;7;7;6;6;2	Scavenger receptor class B member 1	SCARB1	15.7	56.973	509	2.1073	0.5959	7	-0.746857847
Q9BTT0;Q5TB20;E9PPH5;E9	5;5;4;2;2;1	Acidic leucine-rich nuclear phosphoprotein 32 family me	ANP32E	23.1	30.692	268	1.6442	0.5952	5	-0.748553568
O14929;O14929-2;F8W9G7	8;6;1;1	Histone acetyltransferase type B catalytic subunit	HAT1	27.4	49.512	419	1.3482	0.59421	9	-0.75095521
P54577	7	Tyrosine--tRNA ligase, cytoplasmic	YARS	14.6	59.143	528	1.1065	0.59291	4	-0.754114966
P06744-2;P06744;CON__Q3	9;9;2;2;2;1;1;1;1	Glucose-6-phosphate isomerase	GPI	18.1	64.324	569	1.4761	0.59138	8	-0.757842641
F5H098;P40925;P40925-2;B	12;12;9;9;7;7;4;3;1;1	Malate dehydrogenase;Malate dehydrogenase, cytoplasi	MDH1	45.2	38.627	352	1.7457	0.59097	14	-0.7588432
P39019	12	40S ribosomal protein S19	RPS19	57.2	16.06	145	1.3428	0.59038	17	-0.760284246
Q5JTH9;E9PCK7;Q5JTH9-2;I	8;8;8;7	RRP12-like protein	RRP12	8.5	143.7	1297	1.1386	0.58803	6	-0.766038335
Q9Y2Q3-2;Q9Y2Q3;Q9Y2Q3	11;11;10;8;8;6	Glutathione S-transferase kappa 1	GSTK1	51.8	31.566	282	1.7441	0.58612	30	-0.770732028
F5H6I0;P61769;H0YLF3	4;4;3	Beta-2-microglobulin;Beta-2-microglobulin form pI 5.3	B2M	36.9	13.972	122	1.6271	0.58459	7	-0.774502944
P04114;A8MUN2	213;39	Apolipoprotein B-100;Apolipoprotein B-48	APOB	56	515.6	4563	1.3847	0.58161	294	-0.78187602
Q96AC1-3;Q96AC1;H0YJ34;	4;4;4;4;1	Fermitin family homolog 2	FERMT2	8.6	78.675	687	1.0696	0.58155	3	-0.782024859
Q6YN16;Q6YN16-2;B4E136;	22;18;16;11	Hydroxysteroid dehydrogenase-like protein 2	HSDL2	53.3	45.394	418	1.785	0.58093	24	-0.78356376
E5KLJ5;O60313-2;E5KLJ6;O	22;22;22;22;21;21;4;2	Dynamin-like 120 kDa protein, mitochondrial;Dynamin-li	OPA1	28.3	117.74	1015	1.8038	0.579	22	-0.788364747
Q02318;C9J1K5;F8WD90	13;5;1	Sterol 26-hydroxylase, mitochondrial	CYP27A1	30.5	60.234	531	1.4588	0.57816	12	-0.790459295
P48634;F8VVM9;F8VVQ5;F	2;2;2;2;1;1;1;1;1;1;1;	Protein PRRC2A	PRRC2A	1.4	228.86	2157	1.2584	0.57779	2	-0.79138286
Q8TBA6;Q8TBA6-2;H0YK03	8;8;2	Golgin subfamily A member 5	GOLGA5	11.4	83.023	731	1.3339	0.57762	5	-0.791807399
P54707-2;P54707;REV__Q1	4;4;1;1	Potassium-transporting ATPase alpha chain 2	ATP12A	3.5	116.05	1045	1.7185	0.57761	2	-0.791832375
O95810	4	Serum deprivation-response protein	SDPR	12.5	47.173	425	0.99417	0.57705	5	-0.793231765
P00352;Q5SYQ8;Q5SYQ9;Q	28;12;12;10;2	Retinal dehydrogenase 1	ALDH1A1	77.8	54.861	501	1.6467	0.57664	30	-0.794257179
Q92499	19	ATP-dependent RNA helicase DDX1	DDX1	29.5	82.431	740	1.6435	0.57612	20	-0.795558753
Q9UL15-2;Q9UL15	2;2	BAG family molecular chaperone regulator 5	BAG5	3.9	56.026	488	1.1783	0.57543	2	-0.797287657
Q8NBJ5	15	Procollagen galactosyltransferase 1	GLT25D1	27	71.635	622	1.5582	0.57526	15	-0.797713937
Q7KZF4;H7C597	42;10	Staphylococcal nuclease domain-containing protein 1	SND1	64.9	102	910	1.8473	0.5749	48	-0.798617064
Q2NL82;I3L4E8	3;1	Pre-rRNA-processing protein TSR1 homolog	TSR1	5.2	91.809	804	1.1455	0.57479	2	-0.798893132
P54709;B7Z1N7;C9JA36;C9.	5;3;3;3;1;1;1	Sodium/potassium-transporting ATPase subunit beta-3	ATP1B3	21.9	31.512	279	1.2821	0.57476	4	-0.798968433
Q96C36;J3KR12	11;7	Pyrroline-5-carboxylate reductase 2	PYCR2	41.9	33.637	320	1.5413	0.57455	14	-0.799495647
Q93009;B7Z815;F5H8E5;H3	36;36;35;30;12;1;1;1;	Ubiquitin carboxyl-terminal hydrolase 7;Ubiquitin carbox	USP7	40.5	128.3	1102	1.5411	0.57387	39	-0.801204138
O43715	3	TP53-regulated inhibitor of apoptosis 1	TRIAP1	36.8	8.7858	76	1.3332	0.57378	2	-0.801430413
E9PEJ6;P98196;H0Y547	2;2;2	Probable phospholipid-transporting ATPase IH	ATP11A	1.4	135.85	1191	1.2645	0.57348	2	-0.802184921
O43396;K7ER96;G3V1K0;K7	4;3;2;1;1;1	Thioredoxin-like protein 1	TXNL1	19	32.251	289	1.3435	0.57341	4	-0.80236103
P39687;H0YN26;O43423;H7	8;8;2;2;1	Acidic leucine-rich nuclear phosphoprotein 32 family me	ANP32A	34.1	28.585	249	1.4093	0.57297	4	-0.803468492
H7C1E4;P61966;P61966-2	2;2;1	AP-1 complex subunit sigma-1A	AP1S1	12	22.065	191	1.3027	0.57161	2	-0.806896939
F6S0T5;O60341-2;O60341	15;15;15	Lysine-specific histone demethylase 1A	KDM1A	26.6	95.222	876	1.7246	0.57132	10	-0.80762906
P16403	6	Histone H1.2	HIST1H1C	23.5	21.364	213	1.5905	0.57115	15	-0.808058407
F5GY88;O75448;O75448-2;I	3;3;3;3;3;2;2;1	Mediator of RNA polymerase II transcription subunit 24	MED24	4.6	112.18	1008	1.1026	0.5701	2	-0.810713093
Q9HDC9;H0Y512;Q9HDC9-2	11;10;8	Adipocyte plasma membrane-associated protein	APMAP;C20orf3	35.1	46.48	416	1.2038	0.56796	8	-0.816138767
Q9H0A0;E7ESU4;E9PMU0;E	4;4;2;1	N-acetyltransferase 10	NAT10	4.9	115.73	1025	1.0963	0.56748	2	-0.817358547
P11177;P11177-2;P11177-3	14;14;12;10;8	Pyruvate dehydrogenase E1 component subunit beta, mi	PDHB	53.8	39.233	359	1.6425	0.56638	19	-0.820157773

Q16531;F5GY55;B4DG00;F5	34;30;15;8;8;6;6;5;5;5	DNA damage-binding protein 1	DDB1	33.9	126.97	1140	1.6487	0.56547	24	-0.822477608
B3KXW5;J3KQU9;O43747-2	13;13;13;13;4;3;3;2;2	AP-1 complex subunit gamma-1	AP1G1	19.8	94.139	845	1.5091	0.56533	14	-0.822834837
G5E9I4;H0YCF7;Q9HCU9;E9	2;2;2;2;1	Breast cancer metastasis-suppressor 1	BRMS1	7.2	33.715	290	1.2657	0.56424	2	-0.82561915
Q9Y266	9	Nuclear migration protein nudC	NUDC	31.1	38.242	331	1.5642	0.56088	4	-0.834235955
P42167;G5E972;P42167-2;H	12;11;10;6	Lamina-associated polypeptide 2, isoforms beta/gamma	TMPO	37.4	50.67	454	1.41	0.55852	21	-0.840319152
P61247;D6RG13;D6RAT0;H0	12;11;10;9;9;9;9;8;7;6	40S ribosomal protein S3a	RPS3A	48.5	29.945	264	1.6238	0.55785	22	-0.842050846
H3BRN4;P80404;H3BNQ7;H	18;18;17;3;2;1;1	4-aminobutyrate aminotransferase, mitochondrial	ABAT	50.1	58.14	515	1.0938	0.55411	4	-0.851755691
A2RRP1;A2RRP1-2;H0Y5G7	12;12;9;1	Neuroblastoma-amplified sequence	NBAS	6.3	268.57	2371	1.0836	0.55387	11	-0.852380697
P62917;E9PKU4;E9PIZ3;E9P	6;5;5;5;4;4	60S ribosomal protein L8	RPL8	22.2	28.024	257	1.6641	0.55268	7	-0.855483689
P62857	3	40S ribosomal protein S28	RPS28	46.4	7.8409	69	0.93068	0.552	8	-0.857259828
H0YF29;Q6P1X6;Q6P1X6-2	6;6;6	UPF0598 protein C8orf82	C8orf82	31	28.487	261	1.5628	0.55143	6	-0.858750337
P62829;J3KT29;C9JD32;B9Z	7;5;5;4;3;1	60S ribosomal protein L23	RPL23	52.1	14.865	140	1.2403	0.55079	7	-0.860425728
Q9UPT5;Q9UPT5-6;Q9UPT5	2;2;2;2;2;2	Exocyst complex component 7	EXOC7	3	83.381	735	1.1823	0.55062	2	-0.860871081
P62280	14	40S ribosomal protein S11	RPS11	63.3	18.431	158	1.4532	0.54869	14	-0.865936812
P62753;A2A3R5;A2A3R7	7;5;3	40S ribosomal protein S6	RPS6	34.1	28.68	249	1.4824	0.54808	8	-0.867541605
B1AVU8;P07602-3;C9JIZ6;P	5;5;5;5;5	Proactivator polypeptide;Saposin-A;Saposin-B-Val;Sapos	PSAP	9.5	61.692	559	0.88662	0.54764	6	-0.868700269
Q12904-2;Q12904;D6R937	8;8;2	Aminoacyl tRNA synthase complex-interacting multifunc	AIMP1	34.5	37.039	336	1.4422	0.54747	8	-0.869148184
P63241-2;P63241;I3L504;I3	8;8;7;7;6;3;3;3;3	Eukaryotic translation initiation factor 5A-1;Eukaryotic tra	EIF5A;EIF5AL1	43.5	20.17	184	1.2939	0.54742	12	-0.86927995
B1AKR6;Q9NP97;B4DFR2;H	5;5;4;2;2;1;1;1;1	Dynein light chain roadblock-type 1	DYNLRB1	54.1	16.253	148	0.97742	0.54622	3	-0.872445955
P20340-2;H0YGL6;F5H668;F	12;9;9;8;2;1		RAB6A	61.1	23.548	208	1.5786	0.54559	21	-0.874110893
P28482;A8CZ64;P27361;P27	9;7;2;2;2;2;2;2;1;1;	Mitogen-activated protein kinase 1	MAPK1	39.4	41.389	360	1.0578	0.54535	5	-0.874745661
Q3ZCM7;Q5SQY0;C9JAA5;F	11;10;10;10	Tubulin beta-8 chain	TUBB8;RP11-631N	24.5	49.775	444	1.6913	0.54432	3	-0.877473049
Q9NX58	3	Cell growth-regulating nucleolar protein	LYAR	10	43.614	379	1.0628	0.54139	2	-0.885259855
P27348;E9PG15	16;8	14-3-3 protein theta	YWHAQ	50.6	27.764	245	1.3686	0.54113	17	-0.885952869
P60866-2;P60866;E5RJX2;G	5;5;3;2;2	40S ribosomal protein S20	RPS20	24.6	16.005	142	1.6534	0.53775	13	-0.894992475
O60763-2;O60763;F5GYR8;	17;17;15;15;13;8;1;1;	General vesicular transport factor p115	USO1	21.2	109.19	973	1.4896	0.53751	17	-0.8956365
B8ZZN6;P63165;B9A032;B8	5;5;3;3;2;2	Small ubiquitin-related modifier 1	SUMO1	30.1	16.645	146	1.0191	0.53719	9	-0.896495646
Q9Y617;Q9Y617-2	14;13	Phosphoserine aminotransferase	PSAT1	46.8	40.422	370	1.437	0.5371	9	-0.896737373
Q8TB37;B4DWB0;Q8TB37-2	2;2;1;1;1;1	Iron-sulfur protein NUBPL	NUBPL	11.6	34.082	319	1.177	0.53345	2	-0.906575041
P36543;P36543-2;C9J8H1;P	5;5;4;3;2	V-type proton ATPase subunit E 1	ATP6V1E1	26.5	26.145	226	0.97505	0.53291	4	-0.90803619
P99999;C9JFR7;CON__P628	10;9;2	Cytochrome c	CYCS	66.7	11.749	105	1.4255	0.53214	30	-0.910122243
P62851	5	40S ribosomal protein S25	RPS25	29.6	13.742	125	1.2801	0.53155	8	-0.91172269
Q16576;E9PC52;Q16576-2;(	9;9;8;3;2;1	Histone-binding protein RBBP7	RBBP7	26.8	47.82	425	1.5509	0.53155	3	-0.91172269
Q9BZL1;K7EQ43	3;1	Ubiquitin-like protein 5	UBL5	41.1	8.5468	73	1.4816	0.53134	3	-0.91229277
P09622;E9PEX6;B4DT69;B4	11;10;9;9;2;2	Dihydrolipoyl dehydrogenase, mitochondrial;Dihydrolipo	DLD	25	54.177	509	1.51	0.5282	9	-0.920843793
Q00688;G3V5F2	7;1	Peptidyl-prolyl cis-trans isomerase FKBP3	FKBP3	33.9	25.177	224	1.3416	0.52786	8	-0.921772749
Q14980;Q14980-2;Q14980-	9;9;9;9;5;3;3;3;2;1;1;	Nuclear mitotic apparatus protein 1	NUMA1	5.5	238.26	2115	0.95665	0.52637	7	-0.925850829
O95793;O95793-3;O95793-	3;3;3;3;1;1;1;1;1;1;	Double-stranded RNA-binding protein Staufen homolog	STAU1	6.4	63.182	577	0.6042	0.52578	3	-0.92746883
P61011;P61011-2;G3V4F7	5;5;5	Signal recognition particle 54 kDa protein	SRP54	12.7	55.704	504	1.3823	0.5257	5	-0.92768836
Q13155;F8W950;A8MU58	7;6;6	Aminoacyl tRNA synthase complex-interacting multifunc	AIMP2	36.6	35.348	320	1.4309	0.52284	10	-0.935558576
P46782	14	40S ribosomal protein S5;40S ribosomal protein S5, N-ter	RPS5	68.6	22.876	204	1.4755	0.52217	28	-0.937408521
P33176;Q12840;J3KNA1;O6	24;8;8;7;7;4;1	Kinesin-1 heavy chain	KIF5B	31.8	109.68	963	1.4254	0.52181	9	-0.938403503
O43324;C9J1V9;D6RBD7;C9	10;7;7;7;6;2;1	Eukaryotic translation elongation factor 1 epsilon-1	EEF1E1	73	19.81	174	1.477	0.52104	13	-0.940533963
Q32Q12;P22392-2;P22392;J	15;15;12;11;9;7;6;5;3	Nucleoside diphosphate kinase;Nucleoside diphosphate	NME1-NME2;NME	67.8	32.642	292	1.1384	0.52056	21	-0.941863636
Q969Q0;J3KQN4;H0Y5B4;H	4;2;2;2;2	60S ribosomal protein L36a-like;60S ribosomal protein L3	RPL36AL;RPL36A	25.5	12.469	106	1.4151	0.51828	4	-0.948196373
B5MDF5;P62826;J3KQE5;F5	11;11;10;10;7;3	GTP-binding nuclear protein Ran	RAN	42.1	26.224	233	1.5921	0.51815	19	-0.948558289
Q8WWI5;Q8WWI5-2;Q8WV	3;3;3;1	Choline transporter-like protein 1	SLC44A1	6.2	73.301	657	1.3366	0.51667	2	-0.952684978
Q10570;E9PIM1	21;4	Cleavage and polyadenylation specificity factor subunit 1	CPSF1	17.4	160.88	1443	1.5482	0.51612	21	-0.954221558
P61513;C9J4Z3;E9PEL3;G5E	4;4;3;2	60S ribosomal protein L37a	RPL37A	42.4	10.275	92	0.87537	0.51334	4	-0.962013414
Q9UL46;H0YM70;H0YKU2	15;14;4	Proteasome activator complex subunit 2	PSME2	74.5	27.401	239	1.4977	0.51278	17	-0.963588101
P00390;P00390-3;P00390-2	14;14;14;11;11;6;4;2	Glutathione reductase, mitochondrial	GSR	42.9	56.256	522	1.3404	0.51235	5	-0.964798404
Q9BT22;B4DP08;K7EID2;K7	7;5;3;2;1	Chitobiosyldiphosphodolichol beta-mannosyltransferase	ALG1	21.1	52.518	464	1.2527	0.5117	4	-0.966629862
Q14690	5	Protein RRP5 homolog	PDCD11	2.6	208.7	1871	0.87196	0.51055	4	-0.969875839
P15531-2;P15531	10;10	Nucleoside diphosphate kinase A	NME1	56.5	19.653	177	1.2478	0.51005	2	-0.971289414
Q92688;Q92688-2;Q5T6W8	8;8;4	Acidic leucine-rich nuclear phosphoprotein 32 family me	ANP32B	34.7	28.787	251	1.4913	0.509	7	-0.974262439
B1AK40;Q6P587-2;Q6P587	6;6;6	Acylpyruvase FAHD1, mitochondrial	FAHD1	38.7	27.128	248	0.96996	0.50603	3	-0.982705177
Q8N1G4	2	Leucine-rich repeat-containing protein 47	LRRC47	4.5	63.472	583	0.92762	0.5049	2	-0.985930417
Q9NSD9;F5H6Y1	5;4	Phenylalanine--tRNA ligase beta subunit	FARSB	9.2	66.115	589	0.80703	0.50389	4	-0.988819269
Q53S33	4	BolA-like protein 3	BOLA3	39.3	12.114	107	0.8509	0.50358	2	-0.989707108
P30041	15	Peroxiredoxin-6	PRDX6	75.9	25.035	224	1.3673	0.50311	20	-0.991054229
O43776;B4DN60;K7EQ35;K	6;5;1;1;1	Asparagine--tRNA ligase, cytoplasmic	NARS	13	62.942	548	1.3368	0.50157	4	-0.995477035
E7EPA7;P29401;B4E022;E9	21;21;21;10;3	Transketolase	TKT	46	68.813	631	1.2975	0.50012	14	-0.999653795
P61254;J3QRC4;J3QQV1;J3	11;10;10;10;10;9;8;5;	60S ribosomal protein L26	RPL26;RPL26L1	46.2	17.258	145	1.5992	0.49967	13	-1.000952493
E7EVA0;P27816;P27816-6;F	6;6;6;4;4;4;3;3;3;3;2;	Microtubule-associated protein;Microtubule-associated	MAP4	3.5	245.44	2297	1.4389	0.49914	2	-1.002483572
Q9NR28;Q9NR28-2;Q9NR2	11;11;9;8;7;7;6;5;4;3	Diablo homolog, mitochondrial	DIABLO	62.3	27.131	239	1.5748	0.49714	18	-1.008275907
P23396;J3KN86;E9PL09;E9F	21;19;18;14;14;13;12	40S ribosomal protein S3	RPS3	79	26.688	243	1.4765	0.49707	23	-1.008479061
P11413-2;P11413-3;P11413	31;31;31;20;19;19	Glucose-6-phosphate 1-dehydrogenase	G6PD	63.5	63.826	561	1.345	0.49646	15	-1.010250611
O95163;F5H2T0;H0YDF3	16;15;2	Elongator complex protein 1	IKBKAP	15.4	150.25	1332	1.0952	0.49533	7	-1.013538093
P52209;B4DQJ8;F5H7U0;K7	12;12;11;6;6;5;3	6-phosphogluconate dehydrogenase, decarboxylating	PGD	31.1	53.139	483	1.2596	0.4932	7	-1.019755295
Q93096;Q12974;E9PGJ6;Q5	3;3;3;2;1;1;1;1;1	Protein tyrosine phosphatase type IVA 1;Protein tyrosine	PTP4A1;PTP4A2	17.3	19.815	173	0.97485	0.49268	3	-1.021277187
O43390;O43390-2;Q2L7G6;	19;18;18;11	Heterogeneous nuclear ribonucleoprotein R	HNRNPR	34	70.942	633	1.653	0.49233	31	-1.022302442
P11498;B4DN00;E9PRE7;E9	28;14;13;8	Pyruvate carboxylase, mitochondrial	PC	36.8	129.63	1178	1.3084	0.49223	27	-1.022595506
E7EPK6;P62847;P62847-3;E	5;5;5;5;5	40S ribosomal protein S24	RPS24	16.3	32.43	289	1.3847	0.4916	8	-1.024443179
P56937;P56937-2;P56937-3	7;7;7;3	3-keto-steroid reductase	HSD17B7	27.6	38.206	341	1.3756	0.48863	7	-1.033185653
Q9NRV9;F5GWX2;H0YG71	4;3;1	Heme-binding protein 1	HEBP1	30.7	21.097	189	1.0437	0.4879	4	-1.035342612
O43175;Q5SZU1	14;13	D-3-phosphoglycerate dehydrogenase	PHGDH	33.8	56.65	533	1.4276	0.48742	14	-1.036762645
Q9NPJ3;F5H2L4	6;4	Acyl-coenzyme A thioesterase 13	ACOT13	44.3	14.96	140	1.1127	0.48489	15	-1.044270594
P40939;H0YFD6;B4DYP2	33;10;9	Trifunctional enzyme subunit alpha, mitochondrial;Long-	HADHA	61.9	82.999	763	1.7873	0.48416	63	-1.046444202
P51687	3	Sulfite oxidase, mitochondrial	SUOX	6.1	60.282	545	1.3459	0.48184	3	-1.053373931
P83436	2	Conserved oligomeric Golgi complex subunit 7	COG7	4.2	86.343	770	0.97255	0.48166	2	-1.053912976
P09543;P09543-2;B4DFN6;I	18;18;7;7;6;6;5	2,3-cyclic-nucleotide 3-phosphodiesterase	CNP	46.1	47.578	421	1.335	0.47902	19	-1.061842202
H3BND4;Q6P996;J3KNK7;H	2;2;2;2;2;2;1;1;1	Pyridoxal-dependent decarboxylase domain-containing	PDXDC1;PDXDC2F	3.3	88.761	806	0.78371	0.47744	3	-1.066608654
P49588;H3BPK7	11;4	Alanine--tRNA ligase, cytoplasmic	AARS	16.4	106.81	968	1.1225	0.47724	4	-1.067213127
P60981;F6RFD5;P60981-2	5;5;4	Destrin	DSTN	38.8	18.506	165	1.2252	0.4769	3	-1.068241312
Q9HAV4;H0Y9I3;E2QRM3	10;2;1	Exportin-5	XPO5	11.2	136.31	1204	1.1011	0.47685	10	-1.068392578
P04406;E7EUT4;E7EUT5;O1	17;15;13;1	Glyceraldehyde-3-phosphate dehydrogenase	GAPDH	73.4	36.053	335	1.407	0.47608	91	-1.070724072
Q96PE7;H7BZS7;F5GZ54	5;2;2	Methylmalonyl-CoA epimerase, mitochondrial	MCEE	39.2	18.749	176	0.69317	0.47452	2	-1.0754592
P55145;H7C2D6	14;3	Mesencephalic astrocyte-derived neurotrophic factor	MANF	51.6	20.7	182	1.1061	0.47442	15	-1.075763264
O60487	2	Myelin protein zero-like protein 2	MPZL2	10.2	24.484	215	1.3081	0.47331	2	-1.079142692
P25398	8	40S ribosomal protein S12	RPS12	70.5	14.515	132	1.4785	0.4721	25	-1.082835612
Q9BUT1;Q9BUT1-2;D6RIR6	3;2;2;2;2;1;1;1	3-hydroxybutyrate dehydrogenase type 2	BDH2	13.5	26.724	245	0.88547	0.47055	3	-1.087580065
P05165;P05165-2;P05165-3	13;13;12;2;1;1	Propionyl-CoA carboxylase alpha chain, mitochondrial	PCCA	22.4	80.058	728	1.1159	0.47031	3	-1.088316087
P62277;J3KMX5;E9PS50	8;7;3	40S ribosomal protein S13	RPS13	44.4	17.222	151	1.1291	0.46933	12	-1.091325413
Q96CN7;E7ENF9;D6RE73;D	8;6;5;5	Isochorismatase domain-containing protein 1	ISOC1	40.9	32.236	298	1.4342	0.46899	10	-1.092370934
O14980;C9JKM9;C9J673;C9	42;9;7;5;5;5;4;3;3;3	Exportin-1	XPO1	53.6	123.38	1071	1.4415	0.46764	34	-1.096529758
Q9Y281;G3V5P4;F8WDN3;(	9;8;3;1	Cofilin-2	CFL2	57.8	18.736	166	1.0512	0.46663	4	-1.099649033
P84098;J3KTE4;J3QR09;J3Q	5;5;5;2	60S ribosomal protein L19	RPL19	25.5	23.466	196	1.2466	0.46088	9	-1.117536932
Q15843;E9PL57;E9PS38;F8V	4;3;3;3	NEDD8	NEDD8;NEDD8-M	45.7	9.0714	81	0.62814	0.46078	7	-1.117849996
P37059;H3BS44;H3BNN1;H	6;5;1;1	Estradiol 17-beta-dehydrogenase 2	HSD17B2	14.2	42.785	387	1.1691	0.45839	7	-1.125352524
Q99523;B4DWI3	6;5	Sortilin	SORT1	11.4	92.067	831	1.2337	0.45692	2	-1.129986502
P30405;H0Y548;Q2YDB7	6;2;2	Peptidyl-prolyl cis-trans isomerase F, mitochondrial	PPIF	44	22.04	207	1.0846	0.4566	7	-1.130997235
P14868;Q68CR9;H7BZ35;C9	22;19;9;6;6;4;3	Aspartate--tRNA ligase, cytoplasmic	DARS;DKFZp781B	51.1	57.136	501	1.3755	0.45575	20	-1.133685439
Q08380;K7EP36;K7EKQ5;K7	8;2;2;2	Galectin-3-binding protein	LGALS3BP	18.5	65.33	585	1.1334	0.4532	10	-1.141780234
P54819;F8W1A4;P54819-2;	16;16;16;14;13;13;12	Adenylate kinase 2, mitochondrial	AK2	74.9	26.477	239	1.0979	0.45277	62	-1.143149725
Q9BY77;Q9BY77-2;F8WCX5	3;3;2;2;1;1	Polymerase delta-interacting protein 3	POLDIP3	8.6	46.089	421	0.95112	0.45263	4	-1.143595886
P45954;B4DQ51;H0Y6T4	14;13;2	Short/branched chain specific acyl-CoA dehydrogenase,	ACADSB	43.8	47.485	432	1.2913	0.45101	9	-1.148768673
P61221;D6R9I9;D6RGF4;H0	5;5;2;1	ATP-binding cassette sub-family E member 1	ABCE1	12.5	67.314	599	0.83468	0.44936	3	-1.154056387
P49326;C9JJD1;P49326-2;E	3;3;2;2;2;2	Dimethylaniline monooxygenase [N-oxide-forming] 5	FMO5	7.3	60.22	533	1.1153	0.44623	3	-1.164140586
P55060;P55060-3;F8W904;	38;38;33;29;8	Exportin-2	CSE1L	53.5	110.42	971	1.2479	0.44582	31	-1.165466756
Q9NQT5;Q9NQT5-2	2;1	Exosome complex component RRP40	EXOSC3	8	29.572	275	1.1794	0.44375	2	-1.172180975
P62269;Q5GGW2;J3JS69	14;4;3	40S ribosomal protein S18	RPS18	63.2	17.718	152	1.2653	0.44202	23	-1.177816446
P83731;C9JNW5;C9JXB8	4;4;4	60S ribosomal protein L24	RPL24	24.2	17.779	157	1.297	0.44131	7	-1.180135656
P60891;P21108;B4DNL6;B1	7;5;5;5;3;3;2	Ribose-phosphate pyrophosphokinase 1;Ribose-phospha	PRPS1;PRPS1L1	28.6	34.834	318	0.8497	0.43878	8	-1.188430327
P18669;Q8N0Y7;P15259	13;4;4	Phosphoglycerate mutase 1	PGAM1	70.9	28.804	254	1.0323	0.43857	17	-1.189120966
O96019;H7C5S0;O96019-2;	4;3;2;1;1	Actin-like protein 6A	ACTL6A	11	47.46	429	1.0583	0.43695	2	-1.194459893
Q9UQ80;F8VTY8;F8VRZ3;F	12;11;8;7;5;5;2	Proliferation-associated protein 2G4	PA2G4	35.3	43.786	394	1.1382	0.43665	11	-1.195450755

Q06323;A6NJG9;H0YNE3;H	15;14;14;5;4	Proteasome activator complex subunit 1	PSME1	60.2	28.723	249	1.2747	0.43619	12	-1.196971399
Q13011	17	Delta(3,5)-Delta(2,4)-dienoyl-CoA isomerase, mitochond	ECH1	64.3	35.816	328	1.2228	0.43378	29	-1.204964558
F5H5D3;Q9BQE3;F8VVB9;F	27;27;12;12;7;6;5;5;4	Tubulin alpha-1C chain	TUBA1C	65.7	57.73	519	1.2083	0.43255	77	-1.209061186
Q00796;H0YLA4;H0YKB3;B7	8;6;2;2	Sorbitol dehydrogenase	SORD	32.5	38.324	357	1.0976	0.43248	6	-1.209294678
P00338-3;P00338;F5H308;P	25;25;21;21;16;16;12	L-lactate dehydrogenase A chain;L-lactate dehydrogenas	LDHA	74	39.837	361	1.1343	0.43073	34	-1.215144285
Q4VC31;H7C525;C9JQ41;C9	6;5;5;1	Coiled-coil domain-containing protein 58	CCDC58	59	16.62	144	1.2415	0.42815	12	-1.223811769
A1L0T0;E9PJS0;E9PL44;E9P	19;8;5;1	Acetolactate synthase-like protein	ILVBL	57.1	67.867	632	1.1965	0.42811	22	-1.22394656
O75348;C9JRT2;O95670;F2	4;1;1;1;1;1	V-type proton ATPase subunit G 1	ATP6V1G1	41.5	13.757	118	1.2003	0.42788	4	-1.224721849
Q6UWM9;F5GY78;D6RBL8	6;2;2	UDP-glucuronosyltransferase 2A3	UGT2A3	12.5	60.254	527	1.0757	0.42765	7	-1.225497555
P36578;H3BM89;H3BU31;H	14;9;6;6	60S ribosomal protein L4	RPL4	38.9	47.697	427	1.1369	0.42694	16	-1.22789476
P62244;I3L3P7;H3BVC7	7;6;2	40S ribosomal protein S15a	RPS15A	59.2	14.839	130	1.3672	0.42565	5	-1.232260464
P07437;F8VW92;F8VYX6;Q	25;25;25;25;22;22;19	Tubulin beta chain	TUBB	62.8	49.67	444	1.3657	0.42325	90	-1.240418027
Q15043;Q15043-3;Q15043-	4;4;4;2;2;2;2;1	Zinc transporter ZIP14	SLC39A14	11	54.212	492	1.1959	0.42224	7	-1.243864839
B5MBZ0;Q9HC35;B5MCW9	23;23;21;3	Echinoderm microtubule-associated protein-like 4	EML4	28.3	110.18	992	1.2935	0.422	14	-1.244685096
Q15046-2;Q15046;H3BVA8;	15;15;5;2;1	Lysine--tRNA ligase	KARS	24.6	71.496	625	1.2388	0.42103	18	-1.24800506
P54136;P54136-2;F5H3T8;E	17;16;12;2;2	Arginine--tRNA ligase, cytoplasmic	RARS	31.8	75.378	660	1.0566	0.41979	20	-1.252260295
Q9H8H3;H0YI09;F8VQX6	10;9;7	Methyltransferase-like protein 7A	METTL7A	38.9	28.319	244	1.2757	0.41969	12	-1.252604007
P34913;E5RFU2;J3KPH7;G3	10;10;8;8;5;5;4	Epoxide hydrolase 2	EPHX2	26.1	62.615	555	1.0323	0.41946	10	-1.253394854
P62266;D6RD47;D6R9I7;D6	4;3;2;2;2	40S ribosomal protein S23	RPS23	29.4	15.807	143	1.0302	0.41913	5	-1.254530306
Q13724;F5H6D0;C9J8D4;C9	22;20;14;5;3;2	Mannosyl-oligosaccharide glucosidase	MOGS	39.4	91.916	837	1.3454	0.41912	26	-1.254564728
P62249;Q6IPX4	12;5	40S ribosomal protein S16	RPS16	60.3	16.445	146	1.3669	0.41655	15	-1.263438417
P47914	2	60S ribosomal protein L29	RPL29	14.5	17.752	159	1.0746	0.41638	2	-1.264027322
F8W8S0;Q13409;Q13409-5;	2;2;2;2;2;2;2	Cytoplasmic dynein 1 intermediate chain 2	DYNC1I2	3	71.349	638	1.2239	0.41637	2	-1.264061971
Q96C01;E7EQY1;C9JF51;C9	11;9;3;2	Protein FAM136A	FAM136A	71	15.641	138	1.2032	0.41393	25	-1.272541282
Q9NVS9;B4E1D7;B4E152;B4	5;4;4;4;3	Pyridoxine-5-phosphate oxidase	PNPO	26.4	29.988	261	0.91188	0.41362	2	-1.273622148
P62888;E5RI99;E5RJH3	7;7;3	60S ribosomal protein L30	RPL30	59.1	12.784	115	1.0418	0.41257	14	-1.277289175
O75116;E9PF63;D6REE7;C9	20;16;9;5;2	Rho-associated protein kinase 2	ROCK2	16.1	160.9	1388	0.95521	0.41236	8	-1.278023701
D6RFN0;Q9BT78;D6RAX7;B	3;3;2;2;1	COP9 signalosome complex subunit 4	COPS4	9.4	49.684	438	0.81152	0.40563	2	-1.301763738
J3KQ32;Q9NTK5;Q9NTK5-2	6;6;4;2;2	Obg-like ATPase 1	OLA1	17.1	46.937	416	1.0345	0.40538	3	-1.302653182
C9JGV6;P43487;C9JXG8;B3	3;3;3;2;2;2;1;1	Ran-specific GTPase-activating protein	RANBP1	21	23.33	205	1.0495	0.40537	2	-1.302688771
F8W727;P62910;D3YTB1;D3	4;4;4;1	60S ribosomal protein L32	RPL32	26.1	17.962	153	0.88377	0.40421	6	-1.306823081
P40616;B4DWW1;F8VYN9;	8;8;7;6;1;1;1	ADP-ribosylation factor-like protein 1	ARL1	49.7	20.417	181	0.99271	0.40287	13	-1.311613717
O43837;O43837-2;O43837-	5;5;2	Isocitrate dehydrogenase [NAD] subunit beta, mitochon	IDH3B	14.3	42.183	385	1.0161	0.40057	5	-1.319873718
Q9UL12;B4DPI2;Q5SYV1;Q	4;4;3;2	Sarcosine dehydrogenase, mitochondrial	SARDH	4.7	101.04	918	1.0146	0.39981	3	-1.322613538
P00441;H7BYH4	2;1	Superoxide dismutase [Cu-Zn]	SOD1	32.5	15.936	154	0.90525	0.39727	2	-1.331808243
O60884;H3BMW5	6;2	DnaJ homolog subfamily A member 2	DNAJA2	20.9	45.745	412	1.0112	0.39593	5	-1.336682709
Q6EMK4	2	Vasorin	VASN	3.6	71.712	673	1.0134	0.39461	2	-1.341500578
P50895;K7ENU8;K7ERB7	20;12;2	Basal cell adhesion molecule	BCAM	38.2	67.404	628	1.3038	0.39426	24	-1.342780747
Q9C0H2-4;Q9C0H2;Q9C0H2	2;2;2;1	Protein tweety homolog 3	TTYH3	5.7	57.982	526	0.73438	0.39333	2	-1.346187867
O76021;J3QSV6;I3L3U9;I3L	2;2;1;1;1	Ribosomal L1 domain-containing protein 1	RSL1D1	4.7	54.972	490	0.96306	0.39295	2	-1.347582343
O75521;O75521-2;C9J000;F	21;21;11;10;9;9;5	Enoyl-CoA delta isomerase 2, mitochondrial	ECI2	55.3	43.585	394	1.0741	0.39091	37	-1.355091604
P00367;B4DGN5;B3KV55;P	34;27;25;22	Glutamate dehydrogenase 1, mitochondrial;Glutamate d	GLUD1;GLUD2	70.8	61.397	558	1.2104	0.39009	84	-1.35812108
P06733;P06733-2;K7EM90;I	21;14;10;2;2;2;2;2;2;	Alpha-enolase	ENO1	64.1	47.168	434	1.2157	0.38928	29	-1.361119869
O94876;E9PC87;B4DE04;Q6	2;2;2;2;1	Transmembrane and coiled-coil domains protein 1	TMCC1;DKFZp686	3.1	72.082	653	1.1512	0.38657	2	-1.371198414
Q15436;F5H365;F5H6C4;B3	21;21;15;15;9;5;5;5;5	Protein transport protein Sec23A	SEC23A	39.2	86.16	765	1.1656	0.38526	28	-1.37609569
H7BZT4;P61956;P61956-2;A	2;2;2;1;1;1;1;1	Small ubiquitin-related modifier 2;Small ubiquitin-relate	SUMO2;SUMO3;S	23.2	10.839	95	1.2055	0.38517	6	-1.376432756
P18085;C9JAK5;C9JPM4;F8	10;8;8;1;1;1;1	ADP-ribosylation factor 4	ARF4	64.4	20.511	180	1.0823	0.38371	20	-1.381911731
Q86U38;A8MY76	3;2	Pumilio domain-containing protein C14orf21	C14orf21	6.4	69.437	636	0.73072	0.38265	3	-1.385902695
P27635;F8W7C6;Q96L21;H7	8;7;6;5;5;3;3;1	60S ribosomal protein L10;60S ribosomal protein L10-like	RPL10;RPL10L	45.3	24.604	214	1.1823	0.38264	12	-1.385940398
P32119;A6NIW5;P32119-2	14;9;3	Peroxiredoxin-2	PRDX2	83.3	21.892	198	0.98709	0.38084	20	-1.392743081
Q8N2U9;G5E989;B3KW14;C	2;2;2;2;1;1;1	PQ-loop repeat-containing protein 1	PQLC1	8.9	30.477	271	0.89626	0.38049	2	-1.394069557
Q15800;Q15800-2;D6R952;	2;2;1;1	Methylsterol monooxygenase 1	MSMO1	7.2	35.215	293	0.8684	0.37998	2	-1.39600461
P32969;H0Y9V9;D6RAN4;E7	9;8;8;6;4	60S ribosomal protein L9	RPL9	65.1	21.863	192	0.93699	0.37986	13	-1.396460293
K7EM18;P41567;O60739;K7	8;8;4;1	Eukaryotic translation initiation factor 1;Eukaryotic transl	EIF1;EIF1B	71.9	13.607	121	0.75258	0.37711	8	-1.406942687
Q9BPW8;H7C2U6;C9JDV8;F	15;10;5;3	Protein NipSnap homolog 1	NIPSNAP1	65.5	33.31	284	0.99646	0.37677	37	-1.408243999
O14602;P47813;A6NJH9	3;3;2	Eukaryotic translation initiation factor 1A, Y-chromosoma	EIF1AY;EIF1AX	24.3	16.442	144	0.61105	0.37456	4	-1.416731255
P62241;Q5JR95	11;10	40S ribosomal protein S8	RPS8	55.3	24.205	208	1.2079	0.37225	17	-1.425656246
P63244;J3KPE3;D6RAC2;D6	19;16;15;14;13;13;12	Guanine nucleotide-binding protein subunit beta-2-like	GNB2L1	72.2	35.076	317	1.0607	0.37201	20	-1.426586692
P61313;E7EQV9;E7ENU7;E7	6;5;4;4;3;1	60S ribosomal protein L15;Ribosomal protein L15	RPL15	32.4	24.146	204	0.85132	0.37138	12	-1.429031971
Q8WVC6;K7ESP4;Q8WVC6	6;6;2	Dephospho-CoA kinase domain-containing protein	DCAKD	30.3	26.55	231	0.89991	0.36557	5	-1.451780412
O94855-2;O94855;E9PDM8	7;7;5;5	Protein transport protein Sec24D	SEC24D	9.3	113.08	1033	0.82401	0.36462	2	-1.455534397
Q13308-6;Q13308;Q13308-	26;26;25;25;21;15;12	Inactive tyrosine-protein kinase 7	PTK7	32.3	119.2	1078	1.1114	0.36237	40	-1.464464573
P04156;A2A2V1;P04156-2	2;2;2	Major prion protein	PRNP	7.9	27.661	253	0.91522	0.3595	2	-1.475936324
P62424;Q5T8U3;Q5T8U2	9;6;4	60S ribosomal protein L7a	RPL7A	36.8	29.995	266	1.0144	0.35891	10	-1.478305975
Q16658;J3KNT0;H7BYX5;C9	12;12;2;2;1	Fascin	FSCN1	27.2	54.529	493	0.87325	0.35806	3	-1.481726735
P39023;G5E9G0;H7C422;B5	6;4;4;4;2;2;1	60S ribosomal protein L3	RPL3	15.6	46.108	403	0.99006	0.3549	9	-1.49451552
A8MXP9;P43243;D6REM6;D	21;21;18;15;9;9;9;3;3	Matrin-3	MATR3	32.3	99.966	895	1.091	0.35324	27	-1.501279376
Q7Z7K6;Q7Z7K6-3;Q7Z7K6-	6;6;3	Centromere protein V	CENPV	30.5	29.946	275	0.96143	0.35221	8	-1.505492224
P11166;C9JIM8	3;1	Solute carrier family 2, facilitated glucose transporter me	SLC2A1	8.5	54.083	492	0.74774	0.35114	4	-1.509881745
Q86SX6	3	Glutaredoxin-related protein 5, mitochondrial	GLRX5	26.1	16.628	157	1.0495	0.35064	4	-1.511937511
P62854;F8VZW7;Q5JNZ5	3;2;2	40S ribosomal protein S26;Putative 40S ribosomal protei	RPS26;RPS26P11	31.3	13.015	115	1.0531	0.35022	7	-1.513666621
Q9NUV7;C9JC36;B1AKS3;B	2;2;1;1	Serine palmitoyltransferase 3	SPTLC3;SPTLC2L	6.2	62.049	552	0.73914	0.34788	2	-1.523338356
P53985;Q5T8R5;Q49A45;Q	4;3;3;2	Monocarboxylate transporter 1	SLC16A1	13.2	53.944	500	0.85877	0.34575	5	-1.532198844
O14745;J3QRP6;B3KY21	7;7;1	Na(+)/H(+) exchange regulatory cofactor NHE-RF1	SLC9A3R1	27.4	38.868	358	0.92489	0.34477	9	-1.53629385
P05386	5	60S acidic ribosomal protein P1	RPLP1	94.7	11.514	114	0.76291	0.34442	6	-1.537759174
P35613;P35613-2;P35613-4	8;8;7;7;6;4	Basigin	BSG	25.7	42.2	385	0.95079	0.34094	14	-1.552410225
Q99956;H0Y7W4	3;2	Dual specificity protein phosphatase 9;Dual specificity pr	DUSP9	11.2	41.867	384	0.50559	0.34046	2	-1.554442786
P46776;E9PLL6;E9PJD9;E9P	4;4;3;1	60S ribosomal protein L27a	RPL27A	27.7	16.561	148	1.1557	0.33916	5	-1.559962064
O60220	5	Mitochondrial import inner membrane translocase subu	TIMM8A	79.4	10.998	97	1.082	0.33887	4	-1.561196174
P08107;F8VZJ4;P08107-2;E	30;28;25;24;20;10	Heat shock 70 kDa protein 1A/1B	HSPA1A;HSPA1B	64.9	70.051	641	0.99839	0.33709	57	-1.568794265
P41252;J3KR24;Q5TCD1;Q5	49;45;12;10;8;6;4;1	Isoleucine--tRNA ligase, cytoplasmic	IARS	46.3	144.5	1262	1.1161	0.33617	52	-1.572737111
Q14558-2;Q14558;B4DP31;	6;6;4;3;2;1;1;1;1;1;	Phosphoribosyl pyrophosphate synthase-associated prot	PRPSAP1	23.1	42.467	385	0.78942	0.33364	2	-1.583635831
Q9UII2	4	ATPase inhibitor, mitochondrial	ATPIF1	17.9	12.249	106	0.5441	0.33218	3	-1.589962881
Q16822;B4DW73;H0YM31;	19;14;13;12;11;4;3;2;	Phosphoenolpyruvate carboxykinase [GTP], mitochondri	PCK2	36.6	70.729	640	0.68266	0.329	2	-1.603840511
P46778;G3V1B3	5;3	60S ribosomal protein L21	RPL21	30	18.565	160	0.73799	0.32751	5	-1.610389137
Q02878;F8W181;F8VZ45;F8	12;12;3;2;1;1	60S ribosomal protein L6	RPL6	42.4	32.728	288	0.75541	0.32624	17	-1.615994414
P56277	3	Mature T-cell proliferation 1 neighbor protein	MTCP1NB	38.2	7.7469	68	0.96754	0.32599	3	-1.617100386
P08559-4;P08559-2;P08559	8;8;8;8;3;2;2;2;1;1	Pyruvate dehydrogenase E1 component subunit alpha, s	PDHA1	18.5	47.579	428	0.91805	0.32584	4	-1.617764375
P14174	2	Macrophage migration inhibitory factor	MIF	17.4	12.476	115	1.0146	0.32538	4	-1.619802519
O43464;O43464-3;O43464-	13;11;9;7;1;1	Serine protease HTRA2, mitochondrial	HTRA2	48.9	48.84	458	0.74879	0.32486	19	-1.622109979
P00558;B7Z7A9;E7ERH5;P0	20;18;11;4	Phosphoglycerate kinase 1;Phosphoglycerate kinase	PGK1	61.6	44.614	417	0.85327	0.324	15	-1.625934282
P62760;E7ER47;E9PE24	10;6;5	Visinin-like protein 1	VSNL1	61.8	22.142	191	0.84431	0.32353	11	-1.6280286
P62495;B7Z7P8;Q96CG1;I3	7;7;7;2;2;1	Eukaryotic peptide chain release factor subunit 1	ETF1	22.2	49.03	437	0.84283	0.32316	4	-1.629679459
Q01469;I6L8B7	5;3	Fatty acid-binding protein, epidermal	FABP5	28.9	15.164	135	0.6206	0.32296	5	-1.630572603
P62906	14	60S ribosomal protein L10a	RPL10A	54.4	24.831	217	0.77261	0.32255	15	-1.632405278
P21549	10	Serine--pyruvate aminotransferase	AGXT	36.5	43.009	392	0.89968	0.32204	9	-1.634688201
P49207	4	60S ribosomal protein L34	RPL34	23.1	13.293	117	0.63892	0.32198	6	-1.634957018
J3QQ67;Q07020;H0YHA7;G	8;8;7;7;7;6;5;1	60S ribosomal protein L18	RPL18	42.1	21.728	190	0.73355	0.31989	12	-1.644352201
Q9HA77;H0YFF0;F5H579;F5	12;6;1;1;1	Probable cysteine--tRNA ligase, mitochondrial	CARS2	28.2	62.223	564	0.89183	0.31855	9	-1.650408257
P78504;B4DYR1	12;11	Protein jagged-1	JAG1	12.6	133.8	1218	0.64774	0.31715	6	-1.656762753
Q01130;J3KP15;B4DN89;J3	5;5;4;4;2;2	Serine/arginine-rich splicing factor 2	SRSF2;SFRS2	25.3	25.476	221	0.59238	0.3099	12	-1.69012534
Q9BWD1;B7Z233	9;8	Acetyl-CoA acetyltransferase, cytosolic	ACAT2	43.3	41.35	397	0.82332	0.30949	8	-1.6920353
Q96I25;Q5W010;Q5W011;C	4;2;2;1	Splicing factor 45	RBM17	11.2	44.961	401	0.36678	0.30892	3	-1.694694818
Q7Z4W1;J3KS22;J3QS36;J3	10;10;9;7;6;6;5;1	L-xylulose reductase	DCXR	57	25.913	244	0.78115	0.30664	8	-1.705382192
Q9H3K6;H3BVE0;H3BTW0;	5;3;3;3;3	BolA-like protein 2	BOLA2;BOLA2B	72.1	10.116	86	0.72152	0.30571	3	-1.70976435
Q92522	3	Histone H1x	H1FX	17.8	22.487	213	0.90499	0.30548	3	-1.710850166
G5E9Q6;P35080-2;C9J0J7;C	5;5;5;3;2	Profilin	PFN2	24.5	20.787	188	0.63947	0.3019	6	-1.727857338
P36957;B7Z5W8;Q86SW4;C	7;6;4;1;1;1	Dihydrolipoyllysine-residue succinyltransferase compon	DLST	16.3	48.755	453	0.87577	0.30096	6	-1.732356341
Q9Y6X4;D6RB01;D6R953;D	6;6;1;1	Protein FAM169A	FAM169A	10.9	74.954	670	0.82971	0.30007	2	-1.736629005
Q92820;E5RGF0	12;6	Gamma-glutamyl hydrolase	GGH	44.3	35.964	318	0.84072	0.29845	13	-1.744438841
P68371	25	Tubulin beta-4B chain	TUBB4B	63.1	49.83	445	0.96149	0.29838	11	-1.744777258
H0YMV8;Q71UM5;C9JLI6	5;5;4	40S ribosomal protein S27;40S ribosomal protein S27-like	RPS27L	34	11.345	100	0.57519	0.296	4	-1.756330919
H0YKD8;E9PB24;P46779-2;	6;6;6;6;5;5;5;5	60S ribosomal protein L28	RPL28	28.2	19.072	170	0.91538	0.29572	7	-1.757696276
Q02543;B4DM74	10;9	60S ribosomal protein L18a	RPL18A	49.4	20.762	176	0.66532	0.2957	11	-1.757793851
P36551;H0YA22;D6RER6	19;3;2	Coproporphyrinogen-III oxidase, mitochondrial	CPOX	50	50.151	454	0.84499	0.29477	25	-1.762338392
P26373;J3QSB4;F5H1S2;H3	6;5;4;1	60S ribosomal protein L13	RPL13	29.4	24.261	211	0.60427	0.29395	14	-1.766357317
O15427;J3QSC3;J3QQV2;J3	10;6;6;5;4;4;4;3;2;2;1	Monocarboxylate transporter 4	SLC16A3	28	49.469	465	0.67375	0.29045	9	-1.783638265
Q9P2E9;Q9P2E9-2;Q9P2E9-	38;38;37;31;4;1;1;1;1	Ribosome-binding protein 1	RRBP1	33.8	152.47	1410	0.91075	0.28649	53	-1.803443313
Q9BY49;Q9BY49-2;B4DJS2	17;9;7	Peroxisomal trans-2-enoyl-CoA reductase	PECR	81.8	32.544	303	0.89329	0.28576	27	-1.807124109
J3QQX2;P52565;J3KTF8;J3K	5;5;5;3;3;3;2	Rho GDP-dissociation inhibitor 1	ARHGDIA	36.2	25.831	235	0.74426	0.28291	3	-1.821584922
Q08426;Q08426-2;C9JJE0	33;26;5	Peroxisomal bifunctional enzyme;Enoyl-CoA hydratase/3	EHHADH	56.4	79.494	723	0.91048	0.27629	33	-1.855744748
P61353;K7ELC7;K7EQQ9;K7	6;5;4;1	60S ribosomal protein L27	RPL27	43.4	15.798	136	0.8116	0.27536	6	-1.860609092
P02751;P02751-3;E9PE77;P	24;24;24;24;24;24;24	Fibronectin;Anastellin;Ugl-Y1;Ugl-Y2;Ugl-Y3	FN1	14.3	262.62	2386	0.57061	0.27535	23	-1.860661486
P18077;C9K025;F8WB72;F8	6;4;4;4	60S ribosomal protein L35a	RPL35A	29.1	12.538	110	0.79617	0.27267	8	-1.874772116
Q99424;C9J0G0;C9JY29	2;2;1	Peroxisomal acyl-coenzyme A oxidase 2;Acyl-coenzyme A	ACOX2	4.3	76.826	681	0.42678	0.2719	2	-1.878851944
P27144;D3DQ64	11;9	Adenylate kinase isoenzyme 4, mitochondrial	AK4	66.8	25.268	223	0.74103	0.27173	11	-1.879754242
P18124;A8MUD9;C9JIJ5;C9	12;11;3;2	60S ribosomal protein L7	RPL7	48.4	29.225	248	0.72122	0.2701	12	-1.888434455
Q9Y3U8;J3QSB5;J3KTD3	4;2;1	60S ribosomal protein L36	RPL36	30.5	12.254	105	0.74477	0.26971	7	-1.890519082
F5H2F4;G3V2B8;P11586;G3	20;20;20;12	C-1-tetrahydrofolate synthase, cytoplasmic;Methylenete	MTHFD1	22.7	110.61	1020	0.76495	0.26539	12	-1.913814084
Q8IWU2	2	Serine/threonine-protein kinase LMTK2	LMTK2	1.9	164.9	1503	0.61328	0.26525	2	-1.914575344
Q9NXV6;D6RGD2;J3KNE1	5;3;3	CDKN2A-interacting protein	CDKN2AIP	9.7	61.124	580	0.57144	0.26431	4	-1.919697085
Q14331;E9PRR7;Q9BZ01;F5	2;2;1;1;1;1	Protein FRG1;Protein FRG1B	FRG1;FRG1B	8.9	29.172	258	0.48098	0.26322	2	-1.925658983

P14618;P14618-2;P14618-3	35;31;30;26;19;18;12	Pyruvate kinase isozymes M1/M2;Pyruvate kinase	PKM2	74.6	57.936	531	0.77566	0.2615	53	-1.935117148
P21860;P21860-4;P21860-5	6;6;5;4;4;2;2;1	Receptor tyrosine-protein kinase erbB-3	ERBB3	6.1	148.1	1342	0.56362	0.25989	6	-1.944026972
Q96RS6;Q96RS6-2;Q96RS6-	2;2;2	NudC domain-containing protein 1	NUDCD1	4.3	66.755	583	0.73821	0.25946	2	-1.946415954
P01023;P20742-2;H0YGH6;I	40;5;5;3;2	Alpha-2-macroglobulin	A2M	36.4	163.29	1474	0.78971	0.25878	45	-1.950201973
Q06830	21	Peroxiredoxin-1	PRDX1	89.4	22.11	199	0.63026	0.25693	46	-1.960552741
P30086	11	Phosphatidylethanolamine-binding protein 1;Hippocamp	PEBP1	79.7	21.057	187	0.74379	0.25535	14	-1.969452036
Q6NUQ4;Q6NUQ4-2;H7C0(	5;5;2;2;1	Transmembrane protein 214	TMEM214	7.8	77.15	689	0.48784	0.25511	4	-1.970808643
Q01814;Q01814-5;Q01814-	12;12;12;12;12;11;11	Plasma membrane calcium-transporting ATPase 2	ATP2B2	10	136.87	1243	0.52924	0.25376	2	-1.978463419
P38159;P38159-2;H3BT71;H	13;12;10;10;9;7;7;4;4	RNA-binding motif protein, X chromosome;RNA-binding	RBMX;RBMXL1	33	42.331	391	0.43352	0.24862	20	-2.007985738
Q96LJ7;H0YNC2	7;4	Dehydrogenase/reductase SDR family member 1	DHRS1	28.8	33.909	313	0.6172	0.24533	6	-2.027204431
P51659;F5HE57;E9PB82;E7E	46;43;43;43;34;34;30	Peroxisomal multifunctional enzyme type 2;(3R)-hydroxy	HSD17B4	70	79.685	736	0.74214	0.24428	129	-2.033392345
P05387;H0YDD8;A6NIB2	7;3;1	60S acidic ribosomal protein P2	RPLP2	85.2	11.665	115	0.63913	0.24005	12	-2.058593159
Q15067-2;F5GYQ8;F5H0M0	32;29;27;7;6;2	Acyl-coenzyme A oxidase	ACOX1	70.8	74.667	660	0.71827	0.23987	60	-2.059675361
P40429;Q6NVV1	7;2	60S ribosomal protein L13a	RPL13A	32.5	23.577	203	0.65504	0.23982	9	-2.059976116
P30050;P30050-2	6;4	60S ribosomal protein L12	RPL12	54.5	17.818	165	0.72465	0.23604	7	-2.082896731
O75874;C9J4N6;C9JLU6;C9J	19;9;3;3	Isocitrate dehydrogenase [NADP] cytoplasmic	IDH1	55.3	46.659	414	0.64327	0.23399	14	-2.09548122
Q15067;I3L0T4;K7ELT1;I3L4	32;6;6;3	Peroxisomal acyl-coenzyme A oxidase 1	ACOX1	70.2	74.423	660	0.63435	0.23271	3	-2.103394887
Q3SXM5;Q3SXM5-2;H3BT5	5;5;3;3;1	Inactive hydroxysteroid dehydrogenase-like protein 1	HSDL1	17	37.001	330	0.67689	0.23153	5	-2.110728955
F8W9A1;O95070;A6NGW1;	3;3;3;2;2;1	Protein YIF1A	YIF1A	13.1	32.482	297	0.60941	0.22124	2	-2.176315848
Q86UE4;E5RJU9;H0YB56	7;5;2	Protein LYRIC	MTDH	13.9	63.836	582	0.44066	0.2212	11	-2.176576709
P05388;F8VWS0;Q3B7A4;F	11;10;10;9;8;8;7;7;7;6	60S acidic ribosomal protein P0;60S acidic ribosomal prot	RPLP0;RPLP0P6	53.6	34.273	317	0.68356	0.22117	11	-2.176772386
P62942;Q5W0X3;Q1JUQ5	3;2;1	Peptidyl-prolyl cis-trans isomerase FKBP1A;Peptidyl-pro	FKBP1A	41.7	11.951	108	0.62942	0.20933	3	-2.25614901
P63220;Q8WVC2;Q9BYK1;(	6;6;4;1	40S ribosomal protein S21	RPS21	63.9	9.1113	83	0.46958	0.20831	11	-2.263195997
P07305;P07305-2	5;4	Histone H1.0	H1F0	27.3	20.863	194	0.49588	0.20214	5	-2.306573261
P04075;J3KPS3;H3BQN4;H3	29;27;27;19;14;14;13	Fructose-bisphosphate aldolase A;Fructose-bisphosphat	ALDOA	88.7	39.42	364	0.57511	0.20186	54	-2.308573036
Q5UIP0;Q5UIP0-2;H7BZN3;	2;2;2;1	Telomere-associated protein RIF1	RIF1	1.2	274.46	2472	0.33661	0.19693	2	-2.344245189
P10515;H0YDD4;F5H7M3;E9	6;6;6;4	Dihydrolipoyllysine-residue acetyltransferase componer	DLAT	10.5	68.996	647	0.4736	0.19477	6	-2.360156616
Q06210;Q06210-2;E5RJP4	12;12;1	Glucosamine--fructose-6-phosphate aminotransferase [i	GFPT1	23.3	78.806	699	0.45175	0.19282	5	-2.374673394
Q14118	6	Dystroglycan;Alpha-dystroglycan;Beta-dystroglycan	DAG1	10.4	97.44	895	0.55165	0.19069	4	-2.390698906
Q9NZ45	4	CDGSH iron-sulfur domain-containing protein 1	CISD1	42.6	12.199	108	0.33549	0.1895	5	-2.399730246
P08865;C9J9K3;A6NE09;C9	12;12;10;4	40S ribosomal protein SA	RPSA;RPSAP58	52.5	32.854	295	0.51207	0.185	17	-2.434402824
J3KNC3;A6NFE2	3;2	Uncharacterized protein C12orf70	C12orf70	8.5	33.803	293	0.48036	0.17573	2	-2.508567591
P49756;P49756-3;P49756-2	19;8;8;6;2;2;1	RNA-binding protein 25	RBM25	30.8	100.18	843	0.33516	0.17555	20	-2.510046099
P07148;A8MW49;Q9NQV6	6;4;3	Fatty acid-binding protein, liver	FABP1	53.5	14.208	127	0.38488	0.1751	19	-2.513749011
P09972;A8MVZ9;J3QKP5;J3	5;2;1;1;1;1	Fructose-bisphosphate aldolase C	ALDOC	17.9	39.455	364	0.46484	0.16926	2	-2.562687023
P22090;C9JEH7	12;12	40S ribosomal protein S4, Y isoform 1	RPS4Y1	43	29.455	263	0.2508	0.16886	3	-2.566100476
O43813;E9PHS0;F8WDS9	4;4;1	LanC-like protein 1	LANCL1	15.8	45.283	399	0.4691	0.16853	2	-2.568922667
P49773;D6RD60;D6RE99;D6	6;3;3;3;2	Histidine triad nucleotide-binding protein 1	HINT1	74.6	13.802	126	0.43391	0.16698	8	-2.58225278
Q92673;E9PPB3;E9PP43;E9	8;2;2;2;2	Sortilin-related receptor	SORL1	5.3	248.42	2214	0.34838	0.16588	7	-2.591788143
Q8N3C0;Q8N3C0-4	3;1	Activating signal cointegrator 1 complex subunit 3	ASCC3	1.4	251.46	2202	0.37791	0.16578	2	-2.592658127
P10599;B1ALW1	7;5	Thioredoxin	TXN	51.4	11.737	105	0.41353	0.16458	7	-2.603139067
K7EQG4;O00168	1;1	Phospholemman	FXYD1	11.2	12.05	107	0.38479	0.16407	2	-2.607616627
A0AVT1;A0AVT1-2;H0Y8S8	5;5;3	Ubiquitin-like modifier-activating enzyme 6	UBA6	6.1	117.97	1052	0.35132	0.16346	2	-2.612990455
E7EU23;P50395;P50395-2;C	16;16;13;10;7;7;5;5;3	Rab GDP dissociation inhibitor beta	GDI2	48.3	51.18	449	0.41451	0.16324	12	-2.614933479
P07858;E9PQM1;E9PSG5;E9	10;2;2;2;2;2;2;2;2;2;2	Cathepsin B;Cathepsin B light chain;Cathepsin B heavy ch	CTSB	44	37.821	339	0.39975	0.15922	16	-2.650906527
B4DHX5;E7EQC1;P22570;B7	16;16;16;16;15;15;15	NADPH:adrenodoxin oxidoreductase, mitochondrial	FDXR	35.2	58.303	534	0.3388	0.15669	11	-2.674014985
P24666;B5MCC7;G5E9R5;P2	7;5;5;4;4;3;2	Low molecular weight phosphotyrosine protein phospha	ACP1	60.8	18.042	158	0.46112	0.15322	6	-2.706323469
J3KMX3;P02771	8;8	Alpha-fetoprotein	AFP	18.5	70.449	622	0.29668	0.14518	8	-2.784085374
Q5SRN5;P04439;Q5SRN7;P	12;12;12;10;10;10;10	HLA class I histocompatibility antigen, A-3 alpha chain;HL	HLA-A	37.5	41.368	371	0.33739	0.14346	2	-2.801279559
Q9BRF8;Q9BRF8-2;B4DQ68	9;7;4	Calcineurin-like phosphoesterase domain-containing pro	CPPED1	42.7	35.548	314	0.37927	0.14309	10	-2.805005244
Q06520	5	Bile salt sulfotransferase	SULT2A1	18.6	33.779	285	0.36135	0.13564	4	-2.882145405
P0C024;B4DLE5;H3BUB8;H3	2;2;1;1	Peroxisomal coenzyme A diphosphatase NUDT7	NUDT7	12.6	26.942	238	0.22446	0.13336	2	-2.906602085
Q08257;A6NN60;A6NP24;C	19;16;13;11;11	Quinone oxidoreductase	CRYZ	79	35.206	329	0.35396	0.13019	13	-2.941309457
P35579;P35579-2	22;18	Myosin-9	MYH9	13.2	226.53	1960	0.23783	0.12642	2	-2.983703375
P23141-2;P23141;I3L0R9	33;33;26	Liver carboxylesterase 1	CES1	65.7	62.591	568	0.23654	0.12395	2	-3.012169823
Q96EK6;G3V5E4;G3V4W4	5;4;3	Glucosamine 6-phosphate N-acetyltransferase	GNPNAT1	36.4	20.749	184	0.28191	0.12369	5	-3.015199228
O60701;O60701-2;B4DN25;	35;31;29;7;7;7;4;2;2	UDP-glucose 6-dehydrogenase	UGDH	79.4	55.023	494	0.38215	0.12348	47	-3.017650707
Q9Y5X3	2	Sorting nexin-5	SNX5	6.4	46.816	404	0.14457	0.12119	2	-3.044657435
O00748;O00748-2;J3QSC0;J	11;10;4;2	Cocaine esterase	CES2	30.4	61.806	559	0.31303	0.1183	4	-3.079478021
A2BHY4;B0UZ83;B0V2C8;P(	17;17;17;17;17;17;17	Complement C4-A;Complement C4 beta chain;Complem	C4B-1;C4A;C4B	12.7	192.8	1744	0.27199	0.11781	8	-3.085466091
Q9UKM9;Q9UKM9-2;Q5QP	10;10;10;6;6;5	RNA-binding protein Raly	RALY	34.3	32.463	306	0.24182	0.1128	20	-3.148161027
P10646;C9JBB3;P10646-2;C	1;1;1;1;1;1	Tissue factor pathway inhibitor	TFPI	2.6	35.015	304	0.20859	0.11279	3	-3.148288931
P11532;E9PDN5;P11532-4;I	5;5;5;4;4;2;2;2;2;2;2;	Dystrophin	DMD	1.4	426.74	3685	0.2352	0.11214	3	-3.15662712
P50225;H3BRY5;P50226;P5(	12;10;9;7;6;6;5;3	Sulfotransferase 1A1;Sulfotransferase 1A2	SULT1A1;SULT1A2	54.6	34.165	295	0.24564	0.11158	9	-3.163849638
Q9NRW3	2	Probable DNA dC->dU-editing enzyme APOBEC-3C	APOBEC3C	13.2	22.826	190	0.16018	0.10973	2	-3.187970085
P14209;A6NIW1	2;2	CD99 antigen	CD99	13	18.848	185	0.35539	0.10904	3	-3.197070628
B4DMU0;P32322;E2QRB3;P	14;14;13;12;9;7;7;6;6	Pyrroline-5-carboxylate reductase;Pyrroline-5-carboxyla	PYCR1	53.8	35.98	346	0.24504	0.1088	30	-3.200249538
P50914;E7EPB3	4;4	60S ribosomal protein L14	RPL14	20.9	23.432	215	0.28296	0.10644	5	-3.231887679
Q9H773;H3BSA6;H3BPN2	6;3;1	dCTP pyrophosphatase 1	DCTPP1	38.8	18.681	170	0.19658	0.10528	3	-3.247696701
Q14978-2;Q14978-3;Q1497	6;6;6	Nucleolar and coiled-body phosphoprotein 1	NOLC1	8.9	74.746	709	0.19196	0.1017	11	-3.297608416
J3KQL8;Q9BQE5;E9PM95	4;4;1	Apolipoprotein L2	APOL2	9.8	48.929	449	0.21677	0.098583	4	-3.342517305
P37268;E9PNM1;B7Z1J3;B4	10;9;8;8;8;7;4;3	Squalene synthase	FDFT1	29.5	48.115	417	0.16702	0.095353	13	-3.39057786
Q3LXA3;H0YCY6;I3L252;E9F	16;13;6;5;1	Bifunctional ATP-dependent dihydroxyacetone kinase/F	DAK	43.1	58.946	575	0.18421	0.091045	14	-3.4572764
P61626;F8VV32	5;4	Lysozyme C	LYZ	46.6	16.537	148	0.23333	0.087968	7	-3.506877378
Q9BTZ2;Q9BTZ2-4;E2QRL8;	7;7;7;4;4;3;3;3;3;3;3;	Dehydrogenase/reductase SDR family member 4	DHRS4	24.1	29.537	278	0.18633	0.086493	8	-3.531272812
P52895;B4DK69;P52895-2	16;14;2	Aldo-keto reductase family 1 member C2	AKR1C2	61.6	36.735	323	0.18841	0.083292	5	-3.585678255
H7BY58;J3KP72;P22061-2;P	15;15;15;15;12;4;4;4;	Protein-L-isoaspartate O-methyltransferase;Protein-L-is	PCMT1	56.3	30.358	286	0.19381	0.083269	31	-3.586076691
Q1ET66;P50224;P50224-2;H	11;11;6;5;2;1	Sulfotransferase 1A3/1A4	ST1A5;SULT1A3	46.2	34.779	301	0.18186	0.081797	2	-3.611808258
P51812;B7ZB17;B4DG22;F5	6;6;6;6;3;3;3;3;3;3;3;	Ribosomal protein S6 kinase alpha-3;Ribosomal protein S	RPS6KA3;RPS6KA	9.9	83.735	740	0.12364	0.073313	5	-3.769787147
Q4G0P3;F8WD23;F8W6C8;	2;2;1;1;1;1;1;1;1;1;1;	Hydrocephalus-inducing protein homolog	HYDIN	0.4	575.89	5121	0.14139	0.059592	2	-4.068737522
P51857;P51857-2	1;1	3-oxo-5-beta-steroid 4-dehydrogenase	AKR1D1	2.8	37.376	326	0.11145	0.051412	2	-4.281751054
Q8N6C5-4;Q8N6C5;Q8N6C	13;13;13	Immunoglobulin superfamily member 1	IGSF1	11.5	149.42	1341	0.10365	0.047248	18	-4.403602928
B7ZAC3;Q8TDB8;Q8TDB8-2	5;5;5;5;5;2	Solute carrier family 2, facilitated glucose transporter me	SLC2A14;SLC2A3	8.8	58.249	535	0.12632	0.046057	4	-4.440435748
Q9P0Z9;K7EJU8;K7EK30	15;7;6	Peroxisomal sarcosine oxidase	PIPOX	48.7	44.065	390	0.087356	0.04127	15	-4.598762751
Q5T7Y6;P23297;Q5T7Y4;Q5	2;2;1;1	Protein S100-A1	S100A1	24.5	15.895	147	0.081601	0.039298	5	-4.669400299
D3DS54;Q13268;C9JZP6;Q1	16;15;14;13;11;10	Dehydrogenase/reductase SDR family member 2	DHRS2	68.2	29.926	280	0.071773	0.036877	70	-4.761134895
P15559;P15559-2;P15559-3	13;13;11;11;10;10	NAD(P)H dehydrogenase [quinone] 1	NQO1	47.8	30.867	274	0.080089	0.033617	8	-4.894665206
P25815	4	Protein S100-P	S100P	42.1	10.4	95	0.083431	0.031563	3	-4.985621853
O60218;C9JRZ8-2;C9JRZ8	15;8;7	Aldo-keto reductase family 1 member B10	AKR1B10	64.6	36.019	316	0.060455	0.024001	14	-5.380761673
Q04828;H0Y804;P17516;A6	17;12;6;6	Aldo-keto reductase family 1 member C1	AKR1C1	66.9	36.788	323	0.042853	0.02033	26	-5.620245975
Q9UKD1;Q5JTV1	1;1	Glucocorticoid modulatory element-binding protein 2	GMEB2	2.1	56.421	530	0.0052134	0.0057754	2	-7.435863415

Supplementary Table 1: List of differentially regulated proteins with their corresponding normalized SILAC ratios

Supplementary Table 2

Input IDs	Protein name	Gene Symbol
P21589	5'-NTD	NT5E
Q9NRK6	ABCB10	ABCB10
O00116	ADAS	AGPS
Q6DD88	ATL3	ATL3
Q9H7F0	ATP13A3	ATP13A3
P36542	ATP5C	ATP5C1
P56381	ATP5E	ATP5E
P24539	ATP5F1	ATP5F1
O75947	ATP5H	ATP5H
P56385	ATP5I	ATP5I
J3KQ83	ATP5J	ATP5J
O75964	ATP5L	ATP5L
Q8WWM7	ATXN2L	ATXN2L
O00400	Acetyl-coenzyme A tra	SLC33A1
O00468	Agrin	AGRN
P35221	Alpha-1 catenin	CTNNA1
Q13813	Alpha-fodrin	SPTAN1
Q12955	Ankyrin-G	ANK3
P07355	Annexin II	ANXA2
Q9BXK5	BCL2L13	BCL2L13
O95563	BRP44	MPC2
P61165	C11orf10	TMEM258
Q9UKR5	C14orf1	C14orf1
Q9NPL8	C3orf1	TIMMDC1
Q8N5G0	C4orf52	SMIM20
Q96A33	CCDC47	CCDC47
O43490	CD133	PROM1
Q14108	CD36L2	SCARB2
P13073	COX IV-1	COX4I1
P20674	COX Va	COX5A
Q9UBT7	CTNNAL1	CTNNAL1
O14967	Calmegin	CLGN
P10909	Clusterin	CLU
O75911	DHRS3	DHRS3
Q9P2X0	DPM3 (reg)	DPM3
P27487	DPP4	DPP4
Q9BUN8	Derlin1	DERL1
P00533	EGFR	EGFR
Q9BW60	ELOVL1	ELOVL1
Q9NXB9	ELOVL2	ELOVL2
O75616	ERAL1	ERAL1
Q96RT1	ERBIN	ERBB2IP
P58511	FAM165B	SMIM11
Q01740	FMO1	FMO1
Q14315	Filamin C	FLNC
Q9UBI6	G-protein gamma 12	GNG12
Q10472	GALNT1	GALNT1
Q10471	GALNT2	GALNT2
O00461	GPP130	GOLIM4
C9JLE3	Glypican-3	GPC3
Q5VW38	Gpr107	GPR107
P53701	HCCS	HCCS
P07686	HEXB	HEXB
Q96A26	HGTD-P	FAM162A
Q9BW72	HIGD2A	HIGD2A
P01891	HLA-A11	HLA-A
P01891	HLA-A2	HLA-A
P01891	HLA-A3	HLA-A
P01891	HLA-A68	HLA-A
Q01628	IFITM3	IFITM3
Q14573	IP3R3	ITPR3
P08648	ITGA5	ITGA5
P55285	K-cadherin (CDH6)	CDH6
P33947	KDELR2	KDELR2
O43731	KDELR3	KDELR3
Q9HA82	LAG1 longevity assurar	CERS4
Q32M63	LAG1 longevity assurar	CERS6
Q96G23	LASS2	CERS2
Q9HA82	LASS4	CERS4
Q32M63	LASS6	CERS6
O95202	LETM1	LETM1
Q92604	LPGAT1	LPGAT1
Q9BTT6	LRRC1	LRRC1
O95202	Leucine zipper-EF-han	LETM1
P21397	MAOA	MAOA
P49006	MLP	MARCKSL1
Q9UHA4	MP1	LAMTOR3
O75352	MPDU1	MPDU1
Q86Y39	NDUFA11	NDUFA11
Q16718	NDUFA5	NDUFA5
O96000	NDUFB10	NDUFB10
Q9NX14	NDUFB11	NDUFB11
O43676	NDUFB3	NDUFB3
P55011	NKCC1	SLC12A2
Q9UHQ9	NQO3A2	CYB5R1
P49790	NUP153	NUP153
Q969N2	PIGT	PIGT
O15162	PL scramblase 1	PLSCR1
Q9NRZ7	PLCC	AGPAT3
Q13393	PLD1	PLD1
Q8IV08	Pld3	PLD3
P49768	Presenilin 1	PSEN1
Q9H7F0	Probable cation-transp	ATP13A3
P58511	Protein C21orf51	SMIM11
Q96Q06	Protein KIAA1881	PLIN4
P60602	ROMO1	ROMO1
Q9H0U4	Rab-1B	RAB1B
P20336	Rab-3A	RAB3A
P20337	Rab-3B	RAB3B
Q92930	Rab8B	RAB8B
Q16799	Reticulon 1	RTN1
O95197	Reticulon 3	RTN3
Q96Q06	S3-12	PLIN4
Q14160	SCRIB	SCRIB
Q99643	SDHC	SDHC
O14521	SDHD	SDHD
Q99442	SEC62	SEC62
Q9UGP8	SEC63	SEC63
Q9H9B4	SFXN1	SFXN1
Q9Y666	SLC12A7	SLC12A7
Q02978	SLC25A11	SLC25A11
Q99624	SN1	SLC38A3
Q9Y6A9	SPC12	SPCS1
Q13445	ST2L	TMED1
Q8NHG7	SVIP	SVIP
Q92797	Symplekin	SYMPK
Q96DA6	TIM14	DNAJC19
Q99805	TM9SF2	TM9SF2
Q9HD45	TM9SF3	TM9SF3
Q7Z7H5	TMED4	TMED4
Q5BJD5	TMEM41B	TMEM41B
Q15388	TOM20	TOMM20
Q9NS69	TOM22	TOMM22
P12270	TPR	TPR
Q9Y320	TXNDC14	TMX2
Q9P0S9	Tmem14c	TMEM14C
Q6UW68	Tmem205	TMEM205
Q9HD45	Transmembrane 9 supe	TM9SF3
Q8NBM4	UBAC2	UBAC2
Q96IX5	USMG5	USMG5
Q9NZW5	VAM-1	MPP6
P50552	VASP	VASP
P45880	VDAC 2	VDAC2
F5H301	ZO-2	TJP2
Q8IV08	phospholipase D famil	PLD3
O75616		ERAL1

Supplementary Table 2: List of membrane proteins up-regulated in Hep3B cell line identified by GeneGO cellular component analysis

Supplementary Table 3

Patient ID (Plasma samples)	Sample number	Stage	Agrin concentration (ng/ml)
264	H1	Stage II	57.04022989
265	H2	Stage I	414.3199234
266	H3	Stage IIIB	604.9329502
267	H4	Stage I	undetected
268	H5	Stage IIIB	140.9
269	H6	No information on HCC	59.9137931
276	H7	No information on HCC	405.2203065
278	H8	Stage I or II (no Histo data)	77.15517241
SGH 40	H9	Stage 4<br>Multifocal hcc with mets to lymph nodes, bone and adrenal	547.940613
SGH41	H10	Mixed HCC/cholangiocarcinoma T1	91.52298851

HCC tissues (Western Blot analysis)

Patient ID	Vascular Invasion	Alphafetoprotein (AFP)	BCLCstage	Stage	TumorSize	RecurrenceTime	PathologicalDifferentiat	LiverCirrhosis	HBsAg
1	1	90944	A	2	8	11-Dec-12	3	1	1
2	1	23.81	A	2	6	10-Aug-12	2	1	1
3	0	415.4	A	1	3.5	7-Aug-12	2	1	1
4	0	13.29	A	1	2	10-Aug-12	2	0	1
5	0	3.69	A	1	8	7-Sep-10	3	1	0
6	0	3.17	A	1	8	30-Jun-11	3	0	0
7	0	85332	A	1	4.5	#NULL!	2	1	1
8	1	2794	A	2	14	13-Aug-12	2	1	1
9	1	865	B	2	4	19-May-11	2	0	0
10	0	5.84	C	1	13	15-Feb-11	2	0	0
11	0	64734	A	1	21	10-May-10	3	0	0

Patient information of HCC tissue microarray

Sex	Age	Organ	Pathology	Grade	Stage	TNM	Type
M	42	Liver	Hepatocellular carcinoma	1	III	T3N0M0	Malignant
M	65	Liver	Hepatocellular carcinoma	1	I	T1N0M0	Malignant
M	50	Liver	Hepatocellular carcinoma	1	III	T3N0M0	Malignant
M	55	Liver	Hepatocellular carcinoma	1	II	T2N0M0	Malignant
M	40	Liver	Hepatocellular carcinoma	1	II	T2N0M0	Malignant
M	52	Liver	Degenerative hepatocellular carcinoma tissue (sparse)	-	II	T2N0M0	Malignant
M	42	Liver	Hepatocellular carcinoma	1	III	T3N0M0	Malignant
M	40	Liver	Hepatocellular carcinoma	1	II	T2N0M0	Malignant
M	51	Liver	Hepatocellular carcinoma	1	I	T1N0M0	Malignant
M	49	Liver	Hepatocellular carcinoma	1	II	T2N0M0	Malignant
M	38	Liver	Hepatocellular carcinoma	1~2	III	T3N0M0	Malignant
F	46	Liver	Hepatocellular carcinoma	1~2	III	T3N0M0	Malignant
M	48	Liver	Hepatocellular carcinoma	2	III	T3N0M0	Malignant
F	41	Liver	Hepatocellular carcinoma	2	III	T3N0M0	Malignant
M	37	Liver	Hepatocellular carcinoma	2	III	T3N0M0	Malignant
M	59	Liver	Hepatocellular carcinoma	2	III	T3N0M0	Malignant
M	43	Liver	Hepatocellular carcinoma	2	III	T3N0M0	Malignant
F	60	Liver	Hepatocellular carcinoma	2	III	T3N0M0	Malignant
M	50	Liver	Hepatocellular carcinoma	2	III	T2N0M0	Malignant
F	48	Liver	Hepatocellular carcinoma	2	I	T1N0M0	Malignant
M	44	Liver	Hepatocellular carcinoma	2	II	T2N0M0	Malignant
M	47	Liver	Hepatocellular carcinoma with necrosis	2	II	T2N0M0	Malignant
M	63	Liver	Hepatocellular carcinoma	1	II	T2N0M0	Malignant
F	27	Liver	Hepatocellular carcinoma	2	II	T2N0M0	Malignant
F	63	Liver	Hepatocellular carcinoma	2	III	T3N0M0	Malignant
F	48	Liver	Hepatocellular carcinoma	2	III	T3N0M0	Malignant
M	53	Liver	Hepatocellular carcinoma	2	III	T3N0M0	Malignant
M	42	Liver	Hepatocellular carcinoma (fibrous tissue and necrosis)	-	II	T2N0M0	Malignant
M	62	Liver	Hepatocellular carcinoma	2	III	T3N0M0	Malignant
M	54	Liver	Hepatocellular carcinoma	2	III	T3N0M0	Malignant
M	53	Liver	Hepatocellular carcinoma	2	III	T3N0M0	Malignant
M	57	Liver	Hepatocellular carcinoma	2	III	T3N0M0	Malignant
M	37	Liver	Hepatocellular carcinoma	2	III	T3N0M0	Malignant
M	70	Liver	Hepatocellular carcinoma	2	II	T2N0M0	Malignant
M	53	Liver	Hepatocellular carcinoma	3	II	T2N0M0	Malignant
M	34	Liver	Hepatocellular carcinoma	3	II	T2N0M0	Malignant
M	66	Liver	Hepatocellular carcinoma	3	I	T1N0M0	Malignant
M	34	Liver	Hepatocellular carcinoma	2~3	III	T3N0M0	Malignant
F	14	Liver	Normal hepatic tissue	-	-	-	Normal
F	2	Liver	Normal hepatic tissue with focal mild fatty degeneration of hepatocyte	-	-	-	Normal
M	56	Liver	Normal hepatic tissue	-	-	-	Normal
M	47	Liver	Normal hepatic tissue	-	-	-	Normal
F	50	Liver	Normal hepatic tissue with focal mild fatty degeneration of hepatocyte	-	-	-	Normal
F	35	Liver	Normal hepatic tissue with focal mild fatty degeneration of hepatocyte	-	-	-	Normal
M	35	Liver	Normal hepatic tissue	-	-	-	Normal
M	35	Liver	Normal hepatic tissue	-	-	-	Normal
M	40	Liver	Normal hepatic tissue	-	-	-	Normal
M	40	Liver	Normal hepatic tissue	-	-	-	Normal
M	38	Liver	Normal hepatic tissue	-	-	-	Normal
M	45	Liver	Normal hepatic tissue	-	-	-	Normal
M	47	Liver	Normal hepatic tissue	-	-	-	Normal
M	16	Liver	Normal hepatic tissue	-	-	-	Normal
F	18	Liver	Normal hepatic tissue	-	-	-	Normal
F	21	Liver	Normal hepatic tissue	-	-	-	Normal
M	43	Liver	Normal hepatic tissue	-	-	-	Normal

Supplementary Table 3: Disease information and details of HCC patient cohorts used for Agrin expression analysis
